# Undernutrition as a risk factor for tuberculosis disease

**DOI:** 10.1002/14651858.CD015890.pub2

**Published:** 2024-06-11

**Authors:** Juan VA Franco, Brenda Bongaerts, Maria-Inti Metzendorf, Agostina Risso, Yang Guo, Laura Peña Silva, Melanie Boeckmann, Sabrina Schlesinger, Johanna AAG Damen, Bernd Richter, Annabel Baddeley, Mathieu Bastard, Anna Carlqvist, Maria Nieves Garcia-Casal, Bianca Hemmingsen, Farai Mavhunga, Jennifer Manne-Goehler, Kerri Viney

**Affiliations:** Institute of General PracticeMedical Faculty of the Heinrich-Heine-University DüsseldorfDüsseldorfGermany; Family and Community Medicine DivisionHospital Italiano de Buenos AiresBuenos AiresArgentina; Faculty of Human and Health SciencesUniversity of BremenBremenGermany; Institute for Biometrics and EpidemiologyGerman Diabetes Center (Deutsches Diabetes-Zentrum/DDZ), Leibniz Center for Diabetes Research at Heinrich Heine University DüsseldorfDüsseldorfGermany; Cochrane NetherlandsJulius Center for Health Sciences and Primary Care, University Medical Center Utrecht, Utrecht UniversityUtrechtNetherlands; Global Tuberculosis ProgrammeWorld Health OrganizationGenevaSwitzerland; Department of Nutrition and Food SafetyWorld Health OrganizationGenevaSwitzerland; Department of Noncommunicable DiseasesWorld Health OrganizationGenevaSwitzerland; Brigham and Women's HospitalHarvard Medical SchoolBostonMAUSA

## Abstract

**Background:**

Tuberculosis (TB) is a leading cause of mortality due to an infectious disease, with an estimated 1.6 million deaths due to TB in 2022. Approximately 25% of the global population has TB infection, giving rise to 10.6 million episodes of TB disease in 2022. Undernutrition is a key risk factor for TB and was linked to an estimated 2.2 million TB episodes in 2022, as outlined in the World Health Organization (WHO) Global Tuberculosis Report.

**Objectives:**

To determine the prognostic value of undernutrition in the general population of adults, adolescents, and children for predicting tuberculosis disease over any time period.

**Search methods:**

We searched the literature databases MEDLINE (via PubMed) and WHO Global Index Medicus, as well as the WHO International Clinical Trials Registry Platform (ICTRP) on 3 May 2023 (date of last search for all databases). We placed no restrictions on the language of publication.

**Selection criteria:**

We included retrospective and prospective cohort studies, irrespective of publication status or language. The target population comprised adults, adolescents, and children from diverse settings, encompassing outpatient and inpatient cohorts, with varying comorbidities and risk of exposure to tuberculosis.

**Data collection and analysis:**

We used standard Cochrane methodology and the Quality In Prognosis Studies (QUIPS) tool to assess the risk of bias of the studies. Prognostic factors included undernutrition, defined as wasting, stunting, and underweight, with specific measures such as body mass index (BMI) less than two standard deviations below the median for children and adolescents and low BMI scores (< 18.5) for adults and adolescents. Prognostication occurred at enrolment/baseline. The primary outcome was the incidence of TB disease. The secondary outcome was recurrent TB disease. We performed a random‐effects meta‐analysis for the adjusted hazard ratios (HR), risk ratios (RR), or odds ratios (OR), employing the restricted maximum likelihood estimation. We rated the certainty of the evidence using the GRADE approach.

**Main results:**

We included 51 cohort studies with over 27 million participants from the six WHO regions. Sixteen large population‐based studies were conducted in China, Singapore, South Korea, and the USA, and 25 studies focused on people living with HIV, which were mainly conducted in the African region. Most studies were in adults, four in children, and three in children and adults. Undernutrition as an exposure was usually defined according to standard criteria; however, the diagnosis of TB did not include a confirmatory culture or molecular diagnosis using a WHO‐approved rapid diagnostic test in eight studies. The median follow‐up time was 3.5 years, and the studies primarily reported an adjusted hazard ratio from a multivariable Cox‐proportional hazard model.

**Hazard ratios (HR)**

The HR estimates represent the highest certainty of the evidence, explored through sensitivity analyses and excluding studies at high risk of bias. We present 95% confidence intervals (CI) and prediction intervals, which present between‐study heterogeneity represented in a measurement of the variability of effect sizes (i.e. the interval within which the effect size of a new study would fall considering the same population of studies included in the meta‐analysis).

Undernutrition may increase the risk of TB disease (HR 2.23, 95% CI 1.83 to 2.72; prediction interval 0.98 to 5.05; 23 studies; 2,883,266 participants). The certainty of the evidence is low due to a moderate risk of bias across studies and inconsistency.

When stratified by follow‐up time, the results are more consistent across < 10 years follow‐up (HR 2.02, 95% CI 1.74 to 2.34; prediction interval 1.20 to 3.39; 22 studies; 2,869,077 participants). This results in a moderate certainty of evidence due to a moderate risk of bias across studies.

However, at 10 or more years of follow‐up, we found only one study with a wider CI and higher HR (HR 12.43, 95% CI 5.74 to 26.91; 14,189 participants). The certainty of the evidence is low due to the moderate risk of bias and indirectness.

**Odds ratio (OR)**

Undernutrition may increase the odds of TB disease, but the results are uncertain (OR 1.56, 95% CI 1.13 to 2.17; prediction interval 0.61 to 3.99; 8 studies; 173,497 participants). Stratification by follow‐up was not possible as all studies had a follow‐up of < 10 years. The certainty of the evidence is very low due to the high risk of bias and inconsistency. Contour‐enhanced funnel plots were not reported due to the few studies included.

**Risk ratio (RR)**

Undernutrition may increase the risk of TB disease (RR 1.96, 95% CI 1.73 to 2.21; prediction interval 1.50 to 2.56; 4 studies; 1,475,867 participants). Stratification by follow‐up was not possible as all studies had a follow‐up of < 10 years. The certainty of the evidence is low due to the high risk of bias. Contour‐enhanced funnel plots were not reported due to the few studies included.

**Authors' conclusions:**

Undernutrition probably increases the risk of TB two‐fold in the short term (< 10 years) and may also increase the risk in the long term (> 10 years). Policies targeted towards the reduction of the burden of undernutrition are not only needed to alleviate human suffering due to undernutrition and its many adverse consequences, but are also an important part of the critical measures for ending the TB epidemic by 2030. Large population‐based cohorts, including those derived from high‐quality national registries of exposures (undernutrition) and outcomes (TB disease), are needed to provide high‐certainty estimates of this risk across different settings and populations, including low and middle‐income countries from different WHO regions. Moreover, studies including children and adolescents and state‐of‐the‐art methods for diagnosing TB would provide more up‐to‐date information relevant to practice and policy.

**Funding:**

World Health Organization (203256442).

**Registration:**

**PROSPERO registration:** CRD42023408807

**Protocol:**https://doi.org/10.1002/14651858.CD015890

## Plain language summary

**Does undernutrition increase the risk of tuberculosis?**

**Key messages**

- Having undernutrition doubles the risk of getting tuberculosis.
- These results apply to the general adult population; the risk for other groups, such as adolescents and children, is uncertain.

**What is tuberculosis?**

Tuberculosis (TB) is an infection caused by a bacteria (*Mycobacterium tuberculosis*). It primarily affects the lungs but can also affect other sites in the body. TB spreads through the air when an infected person coughs or sneezes. Some people do not develop symptoms after infection, while others progress with the disease and develop symptoms, such as persistent cough, which may be bloody, weight loss, fever, and night sweats. After a TB diagnosis, this is called **TB disease**.

The diagnosis of TB disease usually involves a molecular test or culture that detects the bacteria in a bodily secretion (such as sputum) after consultation with a doctor.

TB is a major health concern globally; over 10 million people develop TB, and over 1.5 million die from the disease every year.

**What is undernutition?**

Undernutrition is a condition where the body does not get enough essential nutrients, affecting a person's health. Body mass index (BMI) is a measure that takes into account both weight and height. It helps identify if someone is underweight, which is one of the definitions of undernutrition. For children, we use "weight‐for‐height" to assess growth. It compares a child's weight to what is typical for their height, helping to gauge if they are thriving or facing nutritional challenges.

**What did we want to find out?**

We wanted to estimate the risk of getting TB disease for people with undernutrition compared to those without undernutrition.

**What did we do?**

We looked at studies that included people with and without undernutrition over time and compared how frequently each group developed tuberculosis.

**What did we find?**

We included 51 studies with over 27 million participants from the six World Health Organization (WHO) regions. Sixteen large population‐based studies were conducted in China, Singapore, South Korea, and the USA, and 25 studies focused on people living with HIV, which were mainly conducted in the African region. Most studies were in adults, four in children, and three in children and adults combined. On average, the studies followed people for 3.5 years.

We found that having undernutrition doubled the risk of getting sick due to tuberculosis compared to those who had not.

**What are the limitations of the evidence?**

Many of the studies had some limitations. One problem was that many of them used sputum microscopy to diagnose TB in people with symptoms, which might have missed some diagnoses. More accurate methods, such as culture or rapid diagnostic tests, currently exist and would miss less TB.

**How up‐to‐date is this review?**

This evidence is up‐to‐date as of 3 May 2023.

## Summary of findings

**Summary of findings 1:** Undernutrition as a risk factor for tuberculosis disease

Outcomes	Follow‐up	Relative effects (95% CI)	Number of participants (studies)	Certainty of the evidence (GRADE)	Anticipated absolute effects (95% CI)	
					Risk of TB disease in people without undernutrition*	Risk of TB in people with undernutrition*
**Patient or population:** general population **Setting:** outpatient/community **Risk factor:** undernutrition **Comparison:** no undernutrition						
**TB disease** *Assessed as: clinical diagnosis of TB disease, with microbiological confirmation (smear or culture)*	Overall	**HR 2.23** (1.83 to 2.72)	2,883,266 (23 CS)	⊕⊕⊝⊝ LOW^a,b^	110 cases per 100,000 population	**120 more per 100,000** (83 more to 163 more)
	< 10 years follow‐up	**HR 2.02** (1.74 to 2.34)	2,869,077 (22 CS)	⊕⊕⊕⊝ MODERATE^a^		**101 more per 100,000** (74 more to 130 more)
	≥ 10 years follow‐up	**HR 12.43** (5.74 to 26.91)	14,189 (1 CS)	⊕⊕⊝⊝ LOW^a,c^		**657 more per 100,000** (380 more to 847 more)
	< 10 years follow‐up	**OR 1.56** (1.13 to 2.17)	173,497 (8 CS)	⊕⊕⊝⊝ VERY LOW^b,d^		**52 more per 100,000** (13 more to 102 more)
	≥ 10 years	*Not reported (OR) in the included studies*				‐
	< 10 years follow‐up	**RR 1.96** (1.73 to 2.21)	1,475,867 (4 CS)	⊕⊕⊝⊝ LOW^d^		**105 more per 100,000** (80 more to 133 more)
	≥ 10 years follow‐up	*Not reported (RR) in the included studies*				‐
*The risk in the exposed (undernutrition) group (and its 95% confidence interval) is based on the assumed risk in the comparison group and the relative effect of the intervention (and its 95% CI). The baseline risk was taken from the WHO Global TB Report [1], which was 133 cases per 100,000 population, of which 17% was attributable to undernutrition; therefore, an approximation of the incidence in people without undernutrition would be 111 cases per 100,000 population. **CI:** confidence interval; **CS:** cohort studies; **HR:** hazard ratio; **OR:** odds ratio; **QUIPS:** Quality In Prognosis Studies; **RR:** risk ratio; **TB:** tuberculosis.						
**GRADE Working Group grades of evidence** **High certainty:** We are very confident that the true effect lies close to that of the estimate of the effect. **Moderate certainty:** We are moderately confident in the effect estimate: The true effect is likely to be close to the estimate of the effect, but there is a possibility that it is substantially different. **Low certainty:** Our confidence in the effect estimate is limited: The true effect may be substantially different from the estimate of the effect. **Very low certainty:** We have very little confidence in the effect estimate: The true effect is likely to be substantially different from the estimate of effect.						

^a^Downgraded one level due to risk of bias: the study or most of the studies had a low to moderate risk of bias across domains of the QUIPS tool. ^b^Downgraded one level due to inconsistency: prediction interval crossed the line of no effect. ^c^Downgraded one level due to indirectness: the estimates are based on a single study that covered a period from 1971 to 1992, which may not represent the current population at risk for TB. ^d^Downgraded two levels due to risk of bias: the included studies had moderate to high risk of bias across domains of the QUIPS tool.

## Background

### Description of the health condition and context

Tuberculosis (TB) is one of the leading causes of death worldwide and the second‐leading cause of death for a single infectious agent after COVID‐19 [1]. The estimated number of deaths from TB increased between 2019 and 2021, in contrast with the previous trend of a continuing decline [1]. The burden of TB has a widely inequitable distribution across the globe. More than two‐thirds of the estimated 10.6 million TB episodes are detected in the World Health Organization (WHO) Southeast Asia and Africa Regions, with a lower proportion found in the Western Pacific, the Americas, the Eastern Mediterranean, and Europe. Due to the COVID‐19 pandemic, there was an 18% decline in TB notifications between 2019 and 2020, although there has since been some recovery. Fluctuations in both TB notifications and deaths have impacted the lives of individuals and have negatively affected global efforts to end TB as a public health problem, aligned with the WHO 'End TB Strategy' and the UN 'Sustainable Development Goals' [1].

An estimated 2 billion people, equal to 25% of the global population, have been infected with the TB bacteria [1]. Approximately 5% to 10% of infected individuals will develop TB disease at some point in their lives, but some people, especially those with immunosuppressive conditions such as HIV infection, are at a higher risk of falling ill [2]. The WHO estimated that in 2022, about 10.6 million people fell ill with TB, and 1.3 million (including 167,000 people with HIV) died from the disease [1].

Efforts to prevent the spread of TB include the early detection and effective treatment of TB and the provision of TB preventive treatment to people who are at highest risk of progressing from TB infection to TB disease, infection prevention and control measures, Bacillus Calmette‐Guérin (BCG) vaccination, and the detection and control of the underlying health‐related risk factors that increase the risk of TB, such as HIV infection [3]. Tackling the social determinants of health that lead to higher transmission, morbidity, and death rates among more vulnerable populations is also a critical component of preventive efforts [4]. This includes, for example, protecting and promoting the health of people in prisons and other closed settings, migrants, other socially disadvantaged groups, people living with HIV, diabetes, or other immunosuppressive disorders, and children [5].

### Description of the prognostic factor

In their annual Global Tuberculosis Report, the WHO Global Tuberculosis Programme estimated the number of TB episodes attributable to the following five health‐related risk factors for TB: alcohol use disorders, diabetes, HIV, tobacco smoking, and undernourishment (see Table). These five aspects have been identified as risk factors in previous systematic reviews informing the programme [6, 7, 8, 9]. Moreover, socioeconomic status, sex, and age have been reported as important effect modifiers for the risk of TB disease (10).

**1:** Overview of included studies

Study ID	Dates	Country	n	Definition of malnutrition	Follow‐up (years)	Population	Age group	Effect measure in SoF
Ahmed 2018	2010‐2015	Ethiopia	503	BMI < 18.5 kg/m^2^	3.9	PLHIV	Adults	HR
Aibana 2016	2009‐2012	Peru	12,430	BMI < 18.5 kg/m^2^ or < 2 SD for children	1	Household contacts	Both	HR
Alemu 2020	2013‐2018	Ethiopia	566	BMI < 18.5 kg/m^2^	10	PLHIV	Adults	HR
Anaam 2020	2007‐2014	Yemen	815	BMI < 18.5 kg/m^2^	5	Treated tuberculosis	Adults	OR (recurrence)
Ayana 2021	2016‐2019	Ethiopia	471	BMI < 18.5 kg/m^2^	3.66	PLHIV	Adults	‐
Baker 2012	2001‐2004	Taiwan, China	17,715	Not reported	4	General population	Adults	HR
Batista 2013	2007‐2010	Brazil	2362	BMI < 18.5 kg/m^2^	5	PLHIV	Adults	HR
Benjumea‐Bedoya 2019	2005‐2009	Colombia	1040	< ‐1 Z score in weight and height	2	Household contacts	Children	‐
Beshir 2019	2013‐2017	Ethiopia	428	< 2 SD height/weight/height and weight	5	PLHIV	Children	HR
Cegielski 2012	1971‐1992	USA	14,189	BMI < 18.5 kg/m^2^	15.8	General population	Adults	HR
Chan‐Yeung 2007	2000‐2005	China	4212	BMI < 18.5 kg/m^2^	2.5	TB screening programme	Adults	OR
Chang 2015	2005‐2012	Nigeria	32,611	BMI < 18.5 kg/m^2^	2.43	PLHIV	Adults	‐
Chen 2022	2013‐2015	China	26,022	BMI < 18.5 kg/m^2^	2.25	General population	Adults	HR
Cheng 2020	2013‐2015	China	34,076	BMI < 18.5 kg/m^2^	2	General population	Adults	‐
Cho 2022	2010‐2017	South Korea	11,135,332	BMI < 18.5 kg/m^2^	7	General population	Adults	‐
Choi 2021	2011‐2014	South Korea	10,087,903	BMI < 18.5 kg/m^2^	7	General population	Adults	‐
Choun 2013	2003‐2010	Cambodia	2984	BMI < 18.5 kg/m^2^	5	PLHIV	Adults	HR
Dembélé 2010	1998‐2005	Burkina Faso	2383	BMI < 18.5 kg/m^2^	2.29	PLHIV	Adults	‐
Ganesan 2023	2013‐2021	Uganda, Kenya, Tanzania and Nigeria	3171	BMI < 18.5 kg/m^2^	2.98	PLHIV	Adults	HR
Gatechompol 2022	1996‐2020	Thailand	2849	BMI < 18.5 kg/m^2^	7.6	PLHIV	Adults	HR
Gedfew 2020	2013‐2017	Ethiopia	433	BMI < 18.5 kg/m^2^	2.5	Diabetes	Adults	HR
Getu 2022	2016‐2020	Ethiopia	529	BMI < 18.5 kg/m^2^	5	PLHIV	Adults	HR
Hanrahan 2010	2003‐2008	South Africa	3456	BMI < 18.5 kg/m^2^	5	PLHIV	Adults	HR
Jung 2016	2007‐2009	South Korea	1776	BMI < 18.5 kg/m^2^	4	Gastrectomy	Adults	OR
Kim 2018	2002‐2006	South Korea	301,081	BMI < 18.5 kg/m^2^	7	General population	Adults	HR
Kyaw 2022	2011‐2017	Myanmar	20,865	BMI < 18.5 kg/m^2^	2.2	PLHIV	Adults	‐
Leung 2007	2000‐2005	China	42,659	BMI < 18.5 kg/m^2^	2.41	General population	Adults	HR
Li 2013	2004‐2011	Tanzania	6579	< 2 Z‐score weight for length or BMI	0.8	PLHIV	Children	HR
Lin 2018 ‐ NIHS cohort	2001‐2013	Taiwan, China	48,713	BMI < 18.5 kg/m^2^	8.3	General population	Adults	‐
Lin 2018 ‐ NTC cohort	2005‐2013	Taiwan, China	119,340	BMI < 18.5 kg/m^2^	7.3	General population	Adults	OR
Liu 2015	2004‐2012	Tanzania	83,251	BMI 17.0 to 18.5 kg/m^2^	2	PLHIV	Adults	‐
Long 2020	2012‐2018	China	349	BMI < 18.5 kg/m^2^	2	Rheumatological diseases	Adults	OR
Maro 2010	2001‐2005	Tanzania	979	BMI < 18 and BMI < 17 kg/m^2^	3.2	PLHIV	Adults	HR
Moore 2007	2003‐2005	Uganda	1044	BMI < 18 kg/m^2^	1.4	PLHIV	Adults	HR
Morán‐Mendoza 2010	1990‐2000	Canada	42,593	Code in the clinical record	6	Household contacts	Both	‐
Nicholas 2011	2006‐2008	Subsaharan Africa	30,134	BMI < 18.5 kg/m^2^	0.8	PLHIV	Adults	RR
Okwara 2017	2011‐2013	Kenya	428	Weight for age < 80% or stunting	1	Household contacts	Children	OR
Paradkar 2020	2014‐2017	India	997	BMI < 18.5 or < 2 SD for children	0.46	Household contacts	Both	RR
Park 2022	2009‐2017	South Korea	2,396,434	BMI < 18.5 kg/m^2^	7.27	General population	Adults	HR
Park 2023	2002‐2017	South Korea	288,955	BMI < 18.5 kg/m^2^	7.45	Gastrectomy	Adults	‐
Pealing 2015	1990‐2012	UK	1,441,347	BMI < 20 kg/m^2^	4.4	General population	Adults	RR
Sabasaba 2019	2011‐2014	Tanzania	68,378	BMI < 18.5 kg/m^2^	3.4	PLHIV	Adults	‐
Soh 2019	1993‐2014	Singapore	50,398	BMI < 18.5 kg/m^2^	16.9	General population	Adults	‐
Sudfeld 2013	2006‐2009	Tanzania	3389	BMI < 18.5 kg/m^2^	1.64	PLHIV	Adults	‐
Tchakounte Youngui 2020	2010‐2016	9 West African countries	6938	BMI 16 to 21 kg/m^2^	2	PLHIV	Adults	RR
Tiruneh 2019	200‐2012	Ethiopia	600	BMI < 18.5 kg/m^2^	2.16	PLHIV	Adults	HR
Van Rie 2011	2004‐2008	South Africa	7536	BMI < 18.5 kg/m^2^	1.78	PLHIV	Adults	‐
Were 2009	2003‐2005	Uganda	1015	BMI < 18.5 kg/m^2^	1.4	PLHIV	Adults	OR
Worodria 2011	N/A	Uganda	225	BMI < 18.5 kg/m^2^	1	PLHIV	Adults	HR
Yen 2017	2001‐2013	Taiwan, China	46,028	BMI < 18.5 kg/m^2^	13	General population	Adults	‐
Yoo 2021a	2009‐2014	South Korea	1,245,640	BMI < 18.5 kg/m^2^	6.4	General population	Adults	HR
Youn 2022	2002‐2013	South Korea	2226	BMI < 18.5 kg/m^2^	5	General population	Adults	HR (recurrence)

**BMI:** body mass index; **HR:** hazard ratio; **N/A:** not applicable; **NIHS**: National Health Interview Surveys; **NTC**: New Taipei City; **OR:** odds ratio; **PLHIV:** people living with HIV (human immunodeficiency virus); **RR:** risk ratio; **SD:** standard deviation; **SoF:** summary of findings table.

Table. Global estimates of tuberculosis episodes in 2022 attributable to selected risk factors.

**Table:** 

Risk factor	Risk ratio (uncertainty interval)		Number of people with the risk factor (millions)	Attributable TB episodes (millions, uncertainty interval)	
Alcohol use disorders	3.3	(2.1 to 5.2)	297	0.73	(0.52 to 0.99)
Diabetes	1.5	(1.3 to 1.8)	509	0.37	(0.27 to 0.48)
HIV infection	14	(12 to 16)	39	0.89	(0.73 to 1.1)
Smoking	1.6	(1.2 to 2.1)	998	0.70	(0.50 to 0.95)
Undernourishment	3.2	(3.1 to 3.3)	711	2.2	(2.0 to 2.4)

Undernourishment, also referred to as undernutrition, is the deficient intake of essential nutrients resulting from either a lack of food or an imbalanced diet that does not provide adequate quantities of the necessary calories, macronutrients, or micronutrients [11]. Undernutrition can be measured using body mass index and affects every country in the world; it particularly poses a risk for children, who are more vulnerable to TB disease and death. The WHO estimated that in 2022, one in five (148 million) children younger than five years of age across the globe were too short for their age (stunted); 45 million were too thin for their height (wasted) [12]. Furthermore, for adults, the latest available age‐standardised estimates highlight that in 2016, about 9% of people aged 20 years and older were underweight, having a body mass index (BMI) below 18.5 kg/m^2^ [13]. The State of Food Security and Nutrition in the World Report 2023 estimated that between 691 and 783 million people suffered hunger in 2022, with protracted conflict, climate disasters, economic shocks, and the prolonged financial aftermath of the pandemic continuing to drive up needs in recent years (14).

### Health outcomes

Tuberculosis starts with tuberculosis infection and, in subsets of individuals, progresses to tuberculosis disease (TB disease), depending on the ability of the host to control the infection. Individuals with tuberculosis infection are infected with *Mycobacterium tuberculosis* but do not have TB disease (sometimes called active TB) and cannot spread the infection to others. In some people, *M tuberculosis* overcomes the immune system and multiplies, progressing from TB infection to TB disease [2]. Half of the people who progress to TB disease do so within the first year after infection, whilst the incidence of progression decreases beyond five years [15].

TB disease can occur in pulmonary (lungs) and extrapulmonary sites (elsewhere in the body). Pulmonary TB disease is the most common form, and people with this form of TB usually present with a cough and an abnormal chest radiograph. Extrapulmonary TB can involve any other organ, most commonly affecting the larynx, lymph nodes, pleura, brain, kidneys, bones, and joints; extrapulmonary TB is usually not infectious, although laryngeal TB is an exception [2].

The detection of TB commonly involves clinical evaluation, imaging studies, and laboratory microbiology tests [16]. A clinical evaluation involves the assessment of disease symptoms, medical history (including a history of contact with someone with TB), and a physical examination. Imaging diagnostics, including a chest X‐ray or computed tomography (CT) scan, can indicate characteristic patterns associated with pulmonary TB. Rapid molecular tests, which detect the genetic material of the bacteria, are recommended by WHO as the initial diagnostic test for people presenting with signs and symptoms of TB [17]. Culture‐based techniques are also used as they have increased sensitivity for detecting *M tuberculosis*. Other diagnostic tests include sputum smear microscopy and lateral flow urine lipoarabinomannan assay (LF‐LAM) for people living with HIV. However, these tests have less sensitivity than the other methods and sputum smear microscopy is no longer recommended as the primary diagnostic test. The diagnostic approaches used in the included studies vary depending on local resources. In resource‐limited settings or those with limited access to diagnostics, diagnosis based on clinical and radiological findings may be relied upon for prompt treatment initiation. However, molecular tests or culture confirmation is always encouraged [16]. TB in children is typically characterised by paucibacillary disease and has a greater reliance on clinical judgement in the absence of microbiological confirmation, which highlights the need for confirmation through culture‐based techniques and rapid molecular tests [18].

TB can also occur in individuals following successfully completed treatment for TB, also known as recurrent TB, which encompasses both relapse and reinfection [19]. Relapse refers to the reactivation of the same strain of *M tuberculosis* that caused the initial infection, whereas reinfection involves acquiring a new strain of *M tuberculosis* following a previous TB infection. For people with recurrent TB disease, it can be challenging to differentiate between relapse and reinfection due to a similar clinical presentation and an inability to know whether the same strain caused the subsequent infection. However, a large proportion of recurrent TB is related to relapse [19].

Undernutrition may impact both the risk of TB infection and TB disease. While a distinction between these two outcomes may be desirable, it is challenging to delineate in empirical studies since the progression from TB infection to TB disease is commonly not tracked in individuals at risk due to the lack of systematic identification of TB infection or a lack of knowledge about who is infected prior to developing TB disease. The WHO has issued a conditional recommendation to screen people with clinical risk factors as well as the general population in high‐prevalence areas for TB disease. WHO also has separate recommendations on the provision of nutritional interventions, and these are also mostly focused on people with TB disease (20). Therefore, the focus of this review is on undernutrition and TB disease.

### Why it is important to do this review

Previous systematic reviews have provided estimates for the multivariable‐adjusted risk of TB attributable to alcohol (2017), diabetes (2018), tobacco (2010), HIV (annual data from UNAIDS), and undernutrition (2010) [6, 7, 8, 9]. The WHO has commissioned updated reviews to assess the current prognostic value of some of the key health‐related risk factors for TB to update the Population Attributable Factor (PAF) estimates (which are included in the Global TB Report). PAFs inform policy and can help plan and prioritise prevention strategies. Two of these risk factors (diabetes and undernutrition) were prioritised to inform the WHO Global Tuberculosis Report. This review reports on undernutrition and TB disease.

## Objectives

To determine the prognostic value of undernutrition in the general population of adults, adolescents, and children for predicting tuberculosis disease over any time period.

**Table:** 

**Population**	Adults, children, and adolescents from the general population
**Index prognostic factors**	Undernutrition (wasting, stunting, underweight)
**Outcomes**	Incidence of tuberculosis (adjusted risk)
**Timing**	Prognostication: baseline/enrolment. Follow‐up overall, < 10, and ≥ 10 years.
**Setting**	General population/outpatients
**Study design**	Cohort studies

## Methods

We followed the Methodological Expectations for Cochrane Intervention Reviews when conducting the review and the Preferred Reporting Items of Systematic Reviews and Meta‐Analyses (PRISMA) 2020 for the reporting [21].

### Criteria for considering studies for this review

#### Types of studies

We included retrospective and prospective cohort studies. We included studies regardless of their publication status or language.

#### Targeted population

We included adults, adolescents, and children from the general population. These may include individuals from different settings of outpatient care (primary care and hospital care) with variable distribution of comorbidities and risk exposure to tuberculosis (TB). We included studies across all regions of the world regardless of TB infection status.

#### Type of prognostic factor

The parameters used to define undernutrition for the purpose of this review included the following measures [22]:

- Children under five years of age: underweight (low weight‐for‐age), wasting (low weight‐for‐height), stunting (low height‐for‐age).
- Children and adolescents (5 to 19 years) with low BMI, under two standard deviations below the median.
- Adults (> 19 years) with BMI < 18.5 kg/m^2^.

The timing of prognostication was determined upon enrolment/baseline.

#### Types of outcomes to be predicted

##### Primary outcome

Incidence of TB disease, including pulmonary (lungs) and extrapulmonary sites (elsewhere in the body). The diversity of diagnostic approaches, including clinical evaluation, imaging studies, and microbiology, inherently contributed to clinical heterogeneity across the included studies.

##### Secondary outcomes

Incidence of recurrent TB disease, including due to relapse, reinfection, or non‐specified.

##### Time points for outcome assessments

We initially considered other thresholds (1 and 5 years) in our protocol, but as the data were consistent across different time frames, we presented data at medium‐term (< 10 years) and long‐term (≥ 10 years) follow‐up. This change was explored through sensitivity analyses (see below).

###### Exclusion criteria for outcomes

We excluded studies that reported overall incident rates for groups of people with and without undernutrition or unadjusted estimates (risk ratios, odds ratios, or hazard ratios) of the risk estimates. We also excluded studies that modelled the relationship of BMI and weight across all BMI categories (including 'normal' weight and 'obesity') or studies including definitions of 'low BMI' within the range of 'normal weight' (e.g. BMI < 20, 21, 22, 23). These specifications were added after the publication of our protocol (see Discussion, 'Potential bias in the review process').

### Search methods for identification of studies

#### Electronic searches

We conducted a unified search for two risk factors, diabetes and undernutrition, informing two Cochrane reviews of each risk factor independently due to the expected overlap in studies assessing multiple risk factors simultaneously. To identify relevant studies, we developed an empirically derived search strategy for MEDLINE. First, we sourced relevant cohort studies from five known systematic reviews on 'tuberculosis and diabetes' (= 4) [6, 23, 24, 25] and 'tuberculosis and undernutrition' (= 1) [8]. This yielded a set of 25 relevant references as a study pool for diabetes and five relevant studies for undernutrition. We complemented the undernutrition topic by carrying out exploratory searches, resulting in an expanded study pool for undernutrition of 20 studies. After removing duplicates, the final study pool for both topics combined resulted in 43 relevant references for cohort studies (year range 1957 to 2022), which were all indexed in MEDLINE.

We conducted a text analysis of these studies using the tools PubReMiner (hgserver2.amc.nl/cgi‐bin/miner/miner2.cgi) and Yale MeSH Analyzer (mesh.med.yale.edu/) [26] to inform the design of the search strategy. Our final search strategy retrieved 39 of 43 relevant references from MEDLINE. Two older references (from 1957 and 1971) could not be retrieved because they had no abstract; nonetheless, they used a different definition of undernutrition (5% or 10% deviation from normal weight), so they would not have been eligible for this review (27; 28). Two further references could not be retrieved because they focused on post‐transplant patients, and the abstracts did not mention 'body mass index' or 'underweight' as a risk factor; nonetheless, the full text did not provide estimates for undernutrition as a risk factor for TB (29; 30).

Using this strategy (see Supplementary material 1), we conducted a comprehensive search with no restrictions on language of publication, publication status, or study design filter in the following sources from inception of each database to 3 May 2023 (date of last search for all databases):

- MEDLINE (via PubMed).
- WHO Global Index Medicus, comprising five information sources:
  - AIM (African Index Medicus);
  - LILACS (Latin American and Caribbean Health Science Information Database);
  - IMEMR (Index Medicus for the Eastern Mediterranean Region);
  - IMSEAR (Index Medicus for South‐East Asia Region);
  - WPRIM (Western Pacific Region Index Medicus).
- World Health Organization International Clinical Trials Registry Platform (WHO ICTRP) (trialsearch.who.int/Default.aspx).

#### Searching other resources

We screened the reference lists of the 43 relevant studies identified by extracting studies from known systematic reviews and exploratory searches described in the previous section. We also screened the reference lists of other potentially eligible studies or ancillary publications identified by our search. Furthermore, we contacted the study authors of the included studies to identify any articles that we may have missed.

### Data collection and analysis

#### Selection of studies

We used Covidence to identify and remove duplicate records [31]. Two pairs of review authors (BB + AR and MB + SS) independently scanned abstracts and titles to determine which studies should be assessed further following an initial pilot test of the inclusion criteria. Two review authors categorised all potentially relevant records as full‐text or mapped records to studies and classified them as included studies, excluded studies, studies awaiting classification, or ongoing studies, according to the criteria in the *Cochrane Handbook for Systematic Reviews of Interventions* [32]. Any disagreements between the two review authors were resolved through consensus or by recourse to a third review author (JVAF). If a resolution was not possible, we designated the corresponding study as 'awaiting classification'. We contacted the study authors as needed for clarification to determine the health status or diagnostic criteria of included participants. If we received no response, clinical experts in our review group classified the study, or studies were listed as 'awaiting classification'. In Supplementary material 3 we documented the reasons for excluding studies. We present a PRISMA flow diagram showing the study selection process [21].

#### Data extraction and management

We developed a dedicated data extraction form based on the Critical Appraisal and Data Extraction for Systematic Reviews of Prediction Modelling Studies (CHARMS) checklist adapted for prognostic factor studies [33, 34]. We pilot‐tested the data extraction form ahead of time. Two review authors (BB, AR, YG, LPS, MB, SS) independently abstracted the following information from included studies, which we presented in the table 'Characteristics of included studies'.

- Study design (e.g. prospective or retrospective cohort) and sources (surveys, laboratory data, clinical health records, claims data, etc.).
- Study dates, settings and country, inclusion/exclusion criteria.
- Participants: eligibility and recruitment method, characteristics at baseline, including data on other potential risk factors, including some of the following: diabetes, undernutrition, HIV infection, recent TB infection, history of untreated or inadequately treated TB disease, immunosuppressive therapy, cigarette smokers, drug or alcohol use disorders, and socioeconomic status, amongst others.
- Sample size: calculation, number of participants, outcomes used to define sample size, and number of events.
- Prognostic factors: definition and method of measurement of undernutrition, including timing and handling of prognostic factors in the statistical modelling.
- Outcome to be predicted: definition (TB type (pulmonary or extrapulmonary)) and method for measurement of outcome(s), type of outcome(s), time of outcome(s), occurrence, or summary of duration of follow‐up.
- Missing data: number of participants with any missing value, details of attrition (loss to follow‐up); for time‐to‐event outcomes: number of censored observations and handling of missing data.
- Analysis: modelling method, assumptions, unadjusted and adjusted prognostic effect estimates, and adjustment factors used.
- Results: interpretation of presented results, comparison with other studies, discussion of generalisability, strengths, and limitations.
- Study funding sources.
- Declarations of interest by primary investigators.

We resolved any disagreements by discussion or, if required, by consultation with a third review author (BR).

We provided information about potentially relevant ongoing studies in Supplementary material 5 including study identifiers.

We contacted the authors of the included studies to obtain key missing data as needed (in Supplementary material 2 we documented the contact with each author and their responses, when available).

##### Dealing with duplicate and companion publications

In the case of duplicate publications, companion documents, or multiple reports relating to a primary study, we maximised the yield of information by mapping all publications to unique studies and collating all available data. We used the most complete data set aggregated across all known publications. In case of doubt, we prioritised publications reporting the most extended follow‐up times associated with our primary or secondary outcomes.

#### Assessment of risk of bias in included studies

We used the Quality In Prognosis Studies (QUIPS) tool to assess the risk of bias in included studies [35], including the following specifications on using this tool for our review question.

- Study participation
  - Source population (e.g. general population, hospitalised people, or chosen by a diagnosis or location)
  - Sampling frame, recruitment methods, and study participation
  - Period and place of recruitment
  - Inclusion and exclusion criteria (e.g. age cut‐offs or explicit diagnostic criteria)
  - Baseline characteristics: diabetes, undernutrition, sex and age, history of TB, immunosuppressive therapy or status including HIV infection, alcohol use disorder, tobacco use, and socioeconomic status. In studies focused on children, some of these might not be relevant (diabetes, tobacco, and alcohol use).
- Study attrition
  - Proportion available for analysis
  - Attempts to collect data on the characteristics of participants who dropped out, including reasons, outcome, and prognostic factor information and differences from those with complete data
- Prognostic factor measurement
  - Clear, valid, and reliable definition and measurement of undernutrition (weight and height) for all participants
  - Adequate categorisation of the continuous variables for BMI and weight‐for‐age for undernutrition
  - Proportion and handling of missing data on prognostic factors
- Outcome measurement
  - Clear, valid, and reliable definition and measurement of TB disease (microbiological, clinical, radiological, or obtained through pharmacy or clinical health records) for all participants
- Adjustment for other prognostic factors
  - Clear, valid, and reliable definitional measurement of relevant other prognostic factors for all participants
  - Methods used to handle missing data on other prognostic factors
  - Important other prognostic factors are accounted for in the study design or analysis (matching, stratification, restriction, or adjustment)
    - For adults, important other prognostic factors include sex, age, diabetes, immunosuppression including HIV, alcohol intake, tobacco use, or socioeconomic status
    - For children, important other prognostic factors include at least two of the following: sex, age, immunosuppression including HIV, history of TB, or socioeconomic status
- Statistical analysis and reporting: presentation of the analytical strategy, model development, and reporting of results

Two review authors (YG and BB) judged each domain at low, moderate, high, or uncertain risk of bias according to the criteria set out in the tool, considering specificities of rating for observational studies, following an initial pilot test of the rating criteria. Given the expected inconsistent reporting of the prognostic studies, we used an 'unclear' risk option for judging the domains. We also reached an overall judgement of low risk of bias for studies rated as low risk of bias in the domains of adjustment, statistical analysis, and reporting. We also explored this low risk of bias definition in a sensitivity analysis.

We resolved any disagreements by discussion or, if required, by consultation with a third review author (JVAF).

#### Measures of association to be extracted

We only included association measures adjusted for prognostic factors (e.g. diabetes, sex, age, history of TB, immunosuppressive therapy or status such as HIV, alcohol use disorder, tobacco use, and socioeconomic status). We initially planned to extract unadjusted estimates, but we restricted our review to adjusted estimates (see 'Potential biases in the review process'). The type of effect measure was considered as a source of heterogeneity.

#### Dealing with missing data

We contacted study authors for additional or updated information that was not available from the published articles. If measures of the association had not been reported, or details on precision were missing, we estimated these from other information, including sample size, number of events, log‐rank P values and confidence intervals (CIs), and Kaplan‐Meier curves [36, 37, 38].

#### Assessment of heterogeneity

Between‐study heterogeneity concerning the role of undernutrition as a risk factor for TB can be attributed to three crucial aspects:

- Clinical heterogeneity: this encompasses differences in study populations (e.g. age and comorbidities), variations in the definition of the exposure (undernutrition), and co‐interventions (e.g. use of antiretrovirals) within study cohorts. Additionally, differences in outcome measurement, based on culture, clinical assessment and imaging, or indirect assessments (e.g. codes in electronic health records or prescriptions of antituberculosis medication).
- Methodological heterogeneity: this refers to the diversity of methods employed and the extent to which studies were robustly conducted, considering the risk of bias. Variations in the approach to analysis also contribute to methodological heterogeneity. Sample size and potential covariates in regression models can affect the reliability and generalisability of the findings.
- Statistical heterogeneity: we quantified this using tau². We also assessed between‐study heterogeneity using the I² statistic following the guidance in the *Cochrane Handbook* [32], considering:
  - 0% to 40%: might not be important;
  - 30% to 60%: may represent moderate heterogeneity;
  - 50% to 90%: may represent substantial heterogeneity;
  - 75% to 100%: considerable heterogeneity.

#### Assessment of reporting bias

Reporting bias is likely an important problem in prognostic factor research. In addition, the prospective registration of prognostic factor studies is not common, making it impossible to check whether there were reporting deficiencies for all included studies [39].

We explored small‐study effects by creating a funnel plot with the measure of association and its standard error. We tested funnel plot asymmetry using the 'meta' package in STATA by producing contour‐enhanced funnel plots [40]. We interpreted the results cautiously, as these tests often have limited power to detect asymmetry, and many tests yield inadequate type I error rates [41].

#### Data synthesis and meta‐analysis approach

We performed meta‐analyses for the adjusted hazard ratios, risk ratios, or odds ratios for the incidence of TB disease. We presented the results of different effect measures separately and stratified our analyses by time points. We only conducted meta‐analyses when studies were sufficiently similar, and other covariates in the models were similar. If there had been sufficient studies at low risk of bias (e.g. 10 or more), we would have restricted our main meta‐analyses to this subgroup. We conducted our main meta‐analyses on studies that adjusted the analysis, if possible, for the following risk factors [2].

- For adults: sex, age, diabetes, immunosuppression, alcohol intake, and tobacco use
- For children: at least two of the following: sex, age, immunosuppression, history of TB, or socioeconomic status

We used a random‐effects approach for meta‐analyses, employing the restricted maximum likelihood estimation in the 'meta' package for STATA [40, 42]. Using the same software package, we calculated 95% (approximate) prediction intervals alongside our 95% confidence intervals [42].

Where meta‐analysis had not been possible, we would have conducted alternative forms of synthesis, including the summary of effect estimates [43].

#### Subgroup analysis and investigation of heterogeneity

As we did not have sufficient studies per variable, we were unable to conduct meta‐regression analyses, as this method requires sufficient data to yield informative results. Therefore, we explored, when possible, heterogeneity through subgroup analyses, considering the following factors.

- Age and sex
- HIV status or immunosuppression status
- Socioeconomic status
- Local/geographical TB incidence
- Definition and degree of undernutrition (mild, moderate, and severe undernutrition, e.g. marasmus, Kwashiorkor, or BMI < 16 kg/m^2^)
- Post hoc: exploration of the effect of the comparison group (normal weight versus 'no undernutrition')

We interpreted the results of subgroups with caution, considering formal statistical testing as indicated in Section 10.11.3.1 of the *Cochrane Handbook* [32]. If none of these approaches were possible, we provided a narrative discussion of sources of clinical heterogeneity.

#### Sensitivity analysis

We estimated the robustness of our analysis by performing the following sensitivity analyses.

- Risk of bias: restricting our analysis to studies at low risk of bias for the domains of adjustment and statistical analysis and reporting or all domains.
- Complete information: restricting the analysis to studies that reported complete information on risk estimates and precision versus those for which we had to calculate this, based on other reported information as described above in the section 'Dealing with missing data'.
- Adjustment for risk factors: including studies with fewer risk factors in their analysis.

#### Summary of findings table

We presented the absolute risks in a summary of findings table by each defined outcome (incidence of TB disease). We used the GRADE approach for prognostic reviews to assess the quality of evidence of the listed outcomes [44, 45]. The GRADE system classifies the certainty of evidence into one of four grades, as follows:

- High: we are very confident that the variation in risk associated with the prognostic factor (probability of future events in those with/without the prognostic factor) lies close to that of the estimate.
- Moderate: we are moderately confident that the variation in risk associated with the prognostic factor (probability of future events in those with/without the prognostic factor) is likely to be close to the estimate, but there is a possibility that it is substantially different.
- Low: our certainty in the estimate is limited: the variation in risk associated with the prognostic factor (probability of future events in those with/without the prognostic factor) may differ substantially from the estimate.
- Very low: we have very little certainty in the estimate: the variation in risk associated with the prognostic factor (probability of future events in those with/without the prognostic factor) is likely to differ substantially from the estimate.

The certainty of evidence can be downgraded by one (serious concern) or two levels (very serious concern) for the following reasons: risk of bias, inconsistency, indirectness, imprecision, and publication bias. Conversely, the certainty of the evidence can also be upgraded by one level due to a large summary effect.

## Results

### Results of the search

The search identified 5328 records, of which 5084 remained after deduplication, which we screened on title and abstract, with 4864 classified as irrelevant. Then we screened 220 references at the full‐text level, of which we excluded 160 studies (161 references) for various reasons (Supplementary material 3), four were not retrievable (Supplementary material 4), and two were identified as ongoing studies (Supplementary material 5). Finally, we included 51 studies (53 references) reporting on 52 cohorts in this review (Supplementary material 2). The details are provided in the PRISMA flow diagram (Figure).

**1:**
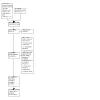


### Characteristics of included studies

An overview of study characteristics is provided in Table.

#### Study design

We included 51 prospective or retrospective cohort studies with 27,646,377 participants. The median sample size was 5396 (interquartile range 1010 to 42,501, range 225 to 11,135,332) participants. The median follow‐up was 3.5 years (interquartile range 2 to 6.2, range 0.5 to 16.9).

Six cohorts recruited participants between the 1970s and 1990s (Cegielski 2012 [46]; Dembélé 2010 [47]; Gatechompol 2022 [48]; Morán‐Mendoza 2010 [49]; Pealing 2015 [50]; Soh 2019 [51]), 28 in the 2000s (Baker 2012 [52]; Chan‐Yeung 2007 [53]; Leung 2007 [54]; Lin 2018 [55]; Maro 2010 [56]; Tiruneh 2019 [57] ‐ National Health Interview Surveys (NHIS) cohort; Benjumea‐Bedoya 2019 [58]; Chang 2015 [59]; Choun 2013 [60]; Hanrahan 2010 [61]; Kim 2018 [62]; Li 2013 [63]; Lin 2018; Liu 2015 [64]; Moore 2007 [65]; Park 2023 [66]; Van Rie 2011 [67]; Were 2009 [68]; Yen 2017 [69]; Youn 2022 [70] ‐ New Taipei City (NTC) cohort; Aibana 2016 [71]; Anaam 2020 [72]; Batista 2013 [73]; Jung 2016 [74]; Nicholas 2011 [75]; Park 2022 [76]; Sudfeld 2013 [77]; Yoo 2021a [78]), and 18 from the 2010s onwards (Ahmed 2018 [79]; Alemu 2020 [80]; Ayana 2021 [81]; Beshir 2019 [82]; Chen 2022 [83]; Cheng 2020 [84, 85]; Cho 2022 [86]; Choi 2021 [87]; Ganesan 2023 [88]; Gedfew 2020 [89, 90]; Getu 2022 [91]; Kyaw 2022 [92]; Long 2020 [93]; Okwara 2017 [94]; Paradkar 2020 [95]; Sabasaba 2019 [96]; Tchakounte Youngui 2020 [97]; Worodria 2011 [98]).

Most studies consisted of clinical cohorts following a group of participants (primarily people living with HIV) and collecting data from clinical health records. However, 16 studies were based on national databases or population‐based cohorts (Cho 2022; Choi 2021; Kim 2018; Lin 2018; Youn 2022 ‐ NIHS cohort; Baker 2012; Cegielski 2012; Chen 2022; Cheng 2020; Lin 2018; Park 2022; Park 2023; Pealing 2015; Yen 2017; Yoo 2021a ‐ NTC cohort; Soh 2019). Finally, six studies focused on the follow‐up of close contacts of people with TB in the context of TB programmes (Aibana 2016; Benjumea‐Bedoya 2019; Chan‐Yeung 2007; Morán‐Mendoza 2010; Okwara 2017; Paradkar 2020). We checked for the overlap of study populations of each study to avoid double‐counting in meta‐analyses (the details are presented in Supplementary material 7).

The studies were conducted in the following WHO Regions: five from the Americas (Brazil, Colombia, Peru, Canada, and the USA), two from Europe (the United Kingdom), 23 from Africa (Burkina Faso, Ethiopia, Kenya, Nigeria, South Africa, Tanzania, Uganda, and some in more than one country), one from the Eastern Mediterranean Region (Yemen), 11 from the Western Pacific Region (Cambodia, China, and Singapore) and 11 from the South‐East Asia Region (India, Myanmar, South Korea, and Thailand). The 16 large population‐based studies were conducted in China, Singapore, South Korea, and the USA, and 22 out of the 23 studies conducted in the African region were on people living with HIV.

#### Study populations

Almost half of the studies only included people living with HIV (25 studies), which were reported in clinical cohorts within programmes or trials of people under antiretroviral therapy (Ahmed 2018; Alemu 2020; Ayana 2021; Batista 2013; Beshir 2019; Chang 2015; Choun 2013; Dembélé 2010; Ganesan 2023; Gatechompol 2022; Getu 2022; Hanrahan 2010; Kyaw 2022; Li 2013; Liu 2015; Maro 2010; Moore 2007; Nicholas 2011; Sabasaba 2019; Sudfeld 2013; Tchakounte Youngui 2020; Tiruneh 2019; Van Rie 2011; Were 2009; Worodria 2011). Sixteen studies were conducted in the general population, usually as large population‐based cohorts or national databases with data linkage from clinical records and other registries (e.g. TB notification systems) (Baker 2012; Cegielski 2012; Leung 2007; Lin 2018; Pealing 2015; Soh 2019 ‐ NIHS cohort; Kim 2018; Lin 2018; Yen 2017; Youn 2022 ‐ NTC cohort; Chen 2022; Cheng 2020; Cho 2022; Choi 2021; Park 2022; Yoo 2021a). These studies usually did not identify HIV infection as an exposure variable due to the low population prevalence (only Cegielski 2012 reported 22 cases, Leung 2007 one case, and Chen 2022 0.1% prevalence). Five studies reported the follow‐up of close contacts of people with TB, i.e. within national TB programmes (Aibana 2016; Benjumea‐Bedoya 2019; Morán‐Mendoza 2010; Okwara 2017; Paradkar 2020). Finally, five studies reported cohorts of participants with specific conditions or diagnoses such as diabetes, gastrectomy, rheumatic diseases, or recent TB (Anaam 2020; Gedfew 2020; Jung 2016; Long 2020; Park 2023).

Most of the studies were in adults; four were in children or adolescents (Benjumea‐Bedoya 2019; Beshir 2019; Li 2013; Okwara 2017). Benjumea‐Bedoya 2019 primarily included children aged 1 to 4 (28.6%), 5 to 9 (21.8%), and 10 to 14 years (34.2%), Beshir 2019 included children ≤ 5 (42.8%), 6 to 10 (35.7%), and ≥ 11 years (21.5%), Li 2013 median age of 5 years (interquartile range 1 to 9), and Okwara 2017 included younger children (mean age 31.4 months, standard deviation 5.6). Additionally, three other studies included a proportion of children and adolescents in their population (43.1% ≤ 19 years Aibana 2016; 8.2% ≤ 10 years Morán‐Mendoza 2010; 31% ≤ 17 years Paradkar 2020).

#### Definition of undernutrition

Most studies in adults included a definition for undernutrition of a BMI < 18.5 kg/m^2^. Two studies used a cut‐off point of 20 or 21 (Pealing 2015; Tchakounte Youngui 2020), but we obtained the estimates for a BMI < 18.5 kg/m^2^ for one of them from the authors (Tchakounte Youngui 2020). Two studies additional provided no definitions beyond "malnutrition/undernutrition" (Baker 2012; Morán‐Mendoza 2010), and the studies in children and adolescents used different measures of undernutrition or low height‐for‐age (stunting) or weight‐for‐height (wasting), usually below two standard deviations below the mean (Aibana 2016; Benjumea‐Bedoya 2019; Beshir 2019; Li 2013; Okwara 2017; Paradkar 2020).

#### Outcomes

Almost all studies reported the primary outcome of the incidence of TB disease. In studies based on national databases or population‐based cohorts, the incidence of TB disease was collected or linked from registries where TB was notified, which is mandatory in most countries. In other studies, the data were collected from clinical records or TB programmes. In most studies, the diagnosis was reached through clinical assessment or active surveillance and a positive acid‐fast smear sputum sample. Few studies included molecular diagnosis or culture in their algorithm, contributing to the risk of bias in the outcome assessment (see below).

Two studies reported the incidence of recurrent TB disease (relapse or reinfection) (Anaam 2020; Youn 2022).

#### Analysis

The studies presented different types of analyses and adjustment methods. Most provided adjusted estimates for a fixed set of selected variables, but most frequently by selecting statistically significant variables in bivariate analysis and then incorporating them in multivariable analysis. Only three studies provided an adjusted estimate for all pre‐defined variables of interest (age, sex, diabetes, alcohol intake, tobacco use, immunosuppression, socioeconomic status, and previous history of TB) (Aibana 2016; Beshir 2019; Li 2013; Li 2013). Whereas most studies reported a hazard ratio adjusted using a Cox‐proportional hazard model, some studies also reported estimates as risk ratios and odds ratios using logistic regression models (which were analysed separately).

### Risk of bias assessment

Here, we summarise the findings per domain of the QUIPS tool. See Figure for a summary of the risk of bias assessment and Supplementary material 2 for detailed support for judgement.

**2:**
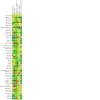
Green indicates low risk of bias; yellow indicates moderate risk of bias; red indicates high risk of bias.

- Study participation: we rated 37 studies as having an 'unclear' risk of bias, as the selected study population (household contacts, people living with HIV or other comorbidities) may have a different risk for TB due to undernutrition than the general population. We rated population‐based studies and national databases with adequate participation as having a 'low' risk of bias.
- Study attrition: we rated 26 studies as 'low' risk of bias due to minimal or no attrition, whereas we rated the rest as moderate or high risk of bias depending on the magnitude and details of how the authors described missing outcome data at follow‐up.
- Prognostic factor measurement: we judged 42 studies to be at a 'low' risk of bias, considering that BMI was a standardised, clinically assessed measure for undernutrition. We rated nine studies as 'moderate' or 'high' due to uncertainties in the definition or cut‐off points used to measure the outcome.
- Outcome measurement: we only classified eight studies as having 'low' risk of bias because they had indicated that culture or similar methods (Xpert) confirmed the diagnosis; 39 studies indicated that diagnosis was based on clinical findings and microbiological acid‐fast sputum smear. Five studies were primarily based on clinical findings with no microbiological confirmation and we therefore classified them as having a 'high' risk of bias.
- Adjustment for other prognostic factors: as we excluded studies without adjustment (i.e. only bivariate estimates), we followed our pre‐defined criteria to judge whether studies adjusted for sufficient variables. Only three studies provided adjusted estimates for all our variables. Moreover, two studies had minimal adjustment based on the limited measurement of characteristics of participants (e.g. only by family type or HIV status).
- Statistical analysis and reporting: considering that many features of the analysis (missing data, selection, and adjustment of variables) are covered in other domains, 18 studies had an incomplete reporting of the selection of variables for adjustments or did not perform additional sensitivity analysis and we categorised them as 'moderate' risk of bias, while we categorised the rest of the studies as 'low' risk of bias.

### Main findings

#### Incident tuberculosis disease

See a detailed description of the findings in Table.

**Hazard ratios (HR)**

These results represent the highest certainty of evidence, explored through sensitivity analyses (see below). We present 95% CI and prediction intervals, which present between‐study heterogeneity represented in a measurement of the variability of effect sizes (i.e. the interval within which the effect size of a new study would fall considering the same population of studies included in the meta‐analysis).

Undernutrition may increase the risk of TB disease (HR 2.23, 95% CI 1.83 to 2.72; prediction interval 0.98 to 5.05; 23 studies; 2,883,266 participants). The certainty of the evidence is low due to a moderate risk of bias across studies and inconsistency.

When stratified by follow‐up time, the results are more consistent across < 10 years follow‐up (HR 2.02, 95% CI 1.74 to 2.34; prediction interval 1.20 to 3.39; 22 studies; 2,869,077 participants). This results in a moderate certainty of the evidence due to a moderate risk of bias across studies.

However, at 10 or more years of follow‐up, we found only one study with a wider CI and higher HR (HR 12.43, 95% CI 5.74 to 26.91; 14,189 participants). The certainty of the evidence is low due to the moderate risk of bias and indirectness.

See Figure for full details of the included studies, their individual effect measures, overall meta‐analysis, and estimates by follow‐up strata. See Figure for the contour‐enhanced funnel plot for assessing publication bias, where we observed no important asymmetry (Begg's test P value = 0.07).

**3:**
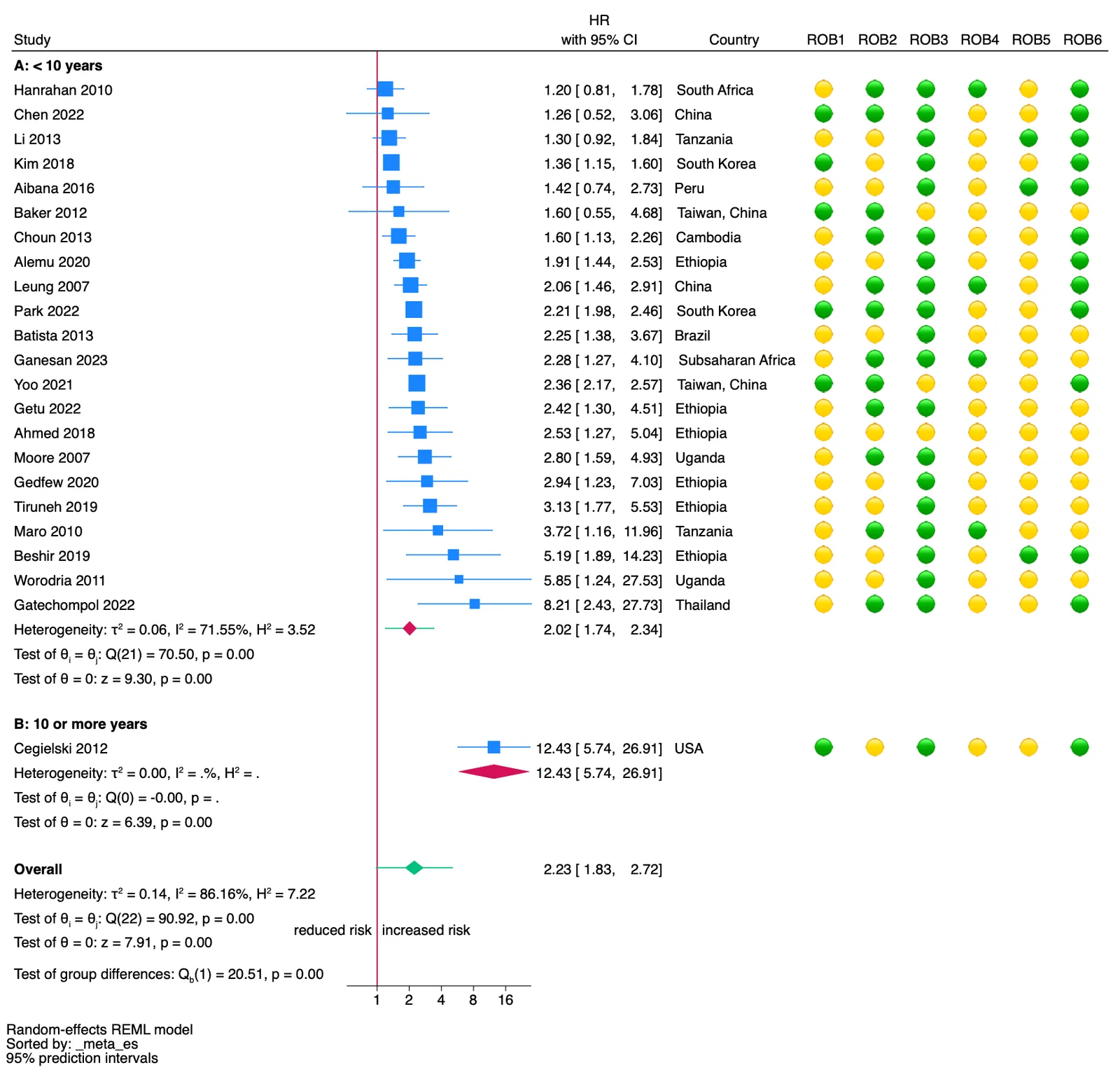
**Hazard ratio of the risk of incident TB disease due to undernutrition.** The blue squares represent the hazard ratios of the individual studies. The red diamond represents the meta‐analysis for each subgroup, the green diamond represents the overall meta‐analysis, and their width represents the confidence interval. The green horizontal line emanating from the diamond represents the prediction interval (which can be found reported in the main text). ROB1: Study Participation; ROB2: Study Attrition; ROB3: Prognostic Factor Measurement; ROB4: Outcome Measurement; ROB5: Study Confounding; ROB6: Statistical Analysis and Reporting. The green, yellow, and red circles in each ROB column represent low, moderate, and high risk of bias, respectively.

**4:**
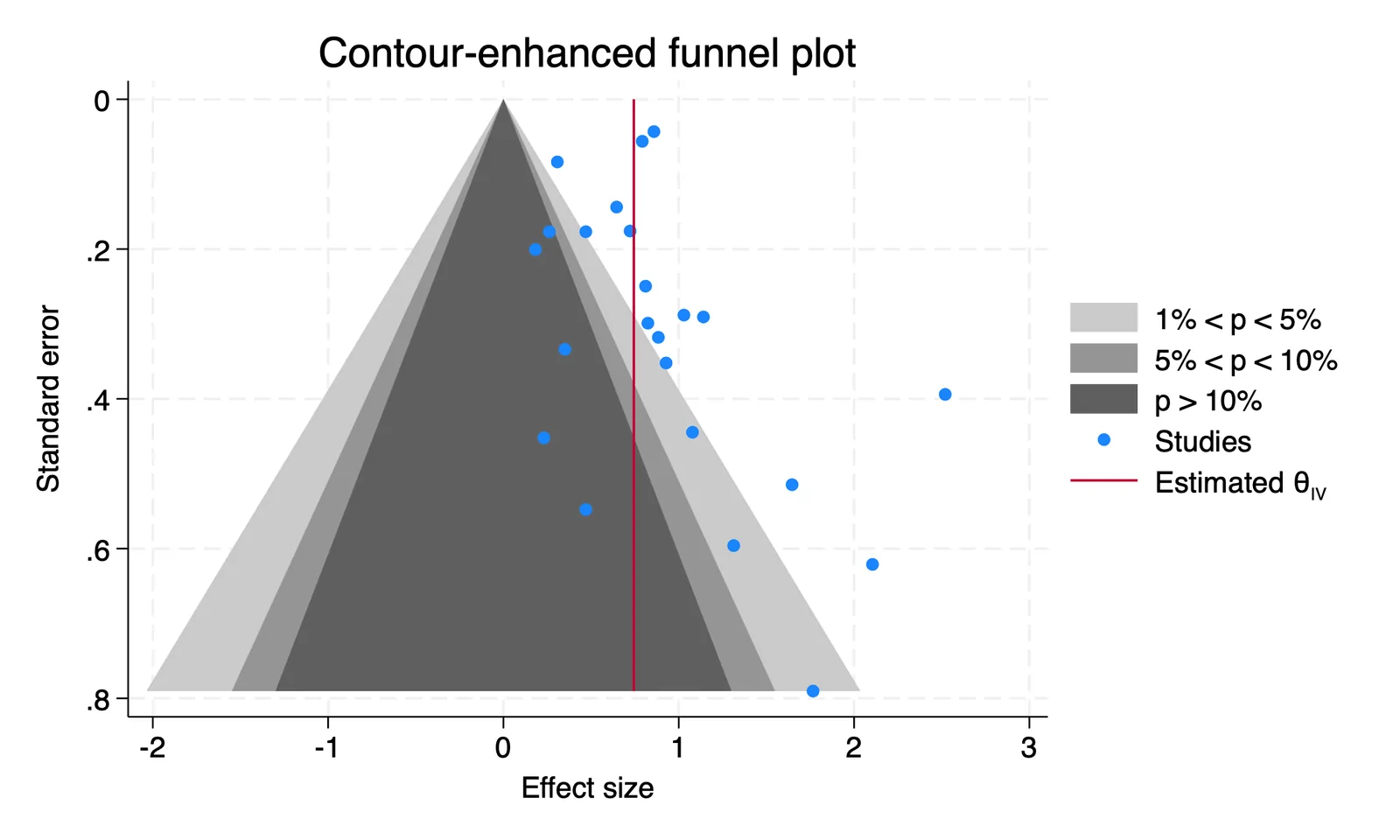
**Contour‐enhanced funnel plot for the overall hazard ratio for the risk of incident TB disease due to undernutrition**. The Y axis represents the standard error, and the X axis represents the log scale of the hazard ratios. The grey areas indicate the regions in which the different P values of the included studies map—Begg's test for asymmetry P value = 0.07.

**Odds ratio (OR)**

Undernutrition may increase the odds of TB disease, but the results are uncertain (OR 1.56, 95% CI 1.13 to 2.17; prediction interval 0.61 to 3.99; 8 studies; 173,497 participants; Figure). Stratification by follow‐up was not possible as all studies had a follow‐up of < 10 years. The certainty of the evidence is very low due to the high risk of bias and inconsistency. Contour‐enhanced funnel plots were not reported due to the few studies included.

**5:**
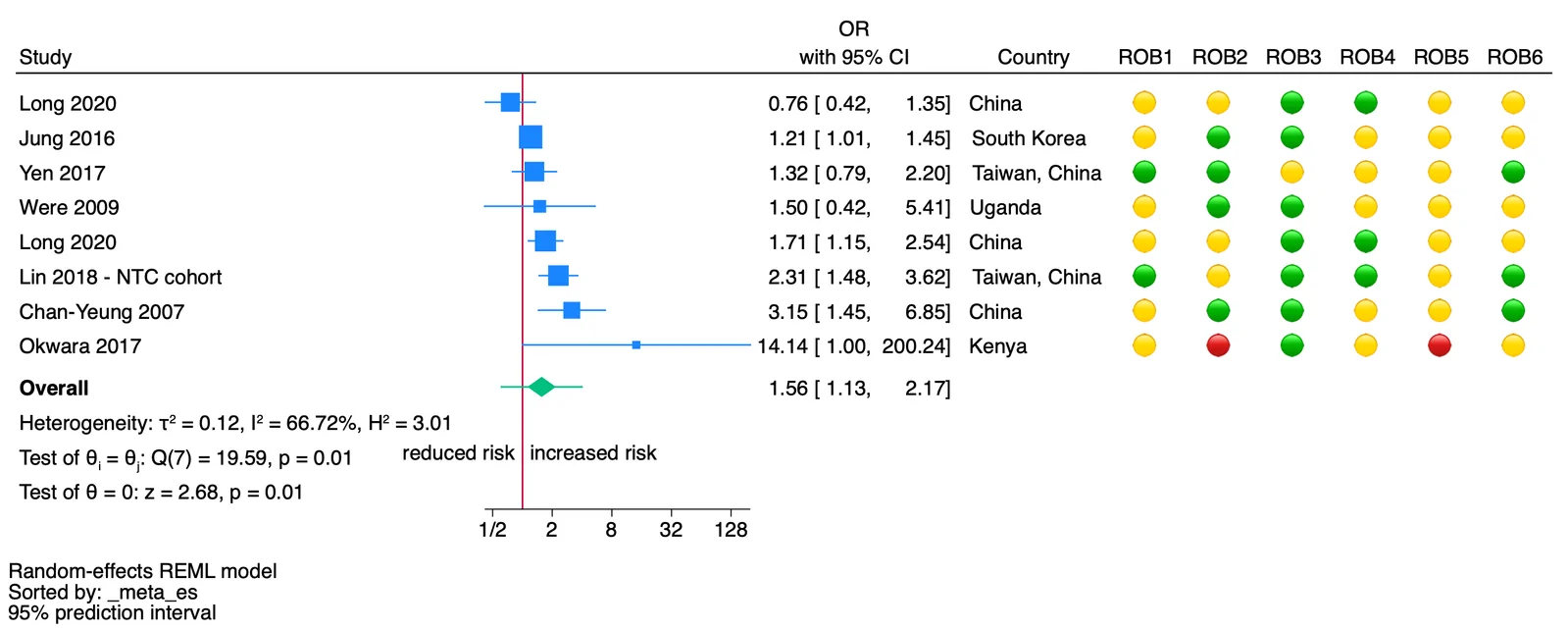
**Odds ratio estimations for incident TB disease due to undernutrition**. The blue squares represent the odds ratios of the individual studies. The green diamond represents the meta‐analysis, and its width represents the confidence interval. The green horizontal line emanating from the diamond represents the prediction interval (which can be found reported in the main text). ROB1: Study Participation; ROB2: Study Attrition; ROB3: Prognostic Factor Measurement; ROB4: Outcome Measurement; ROB5: Study Confounding; ROB6: Statistical Analysis and Reporting. The green, yellow, and red circles in each ROB column represent low, moderate, and high risk of bias, respectively.

**Risk ratio (RR)**

Undernutrition may increase the risk of TB disease (RR 1.96, 95% CI 1.73 to 2.21; prediction interval 1.50 to 2.56; 4 studies; 1,475,867 participants; Figure). Stratification by follow‐up was not possible as all studies had a follow‐up of < 10 years. The certainty of the evidence is low due to the high risk of bias. Contour‐enhanced funnel plots were not reported due to the few studies included.

**6:**
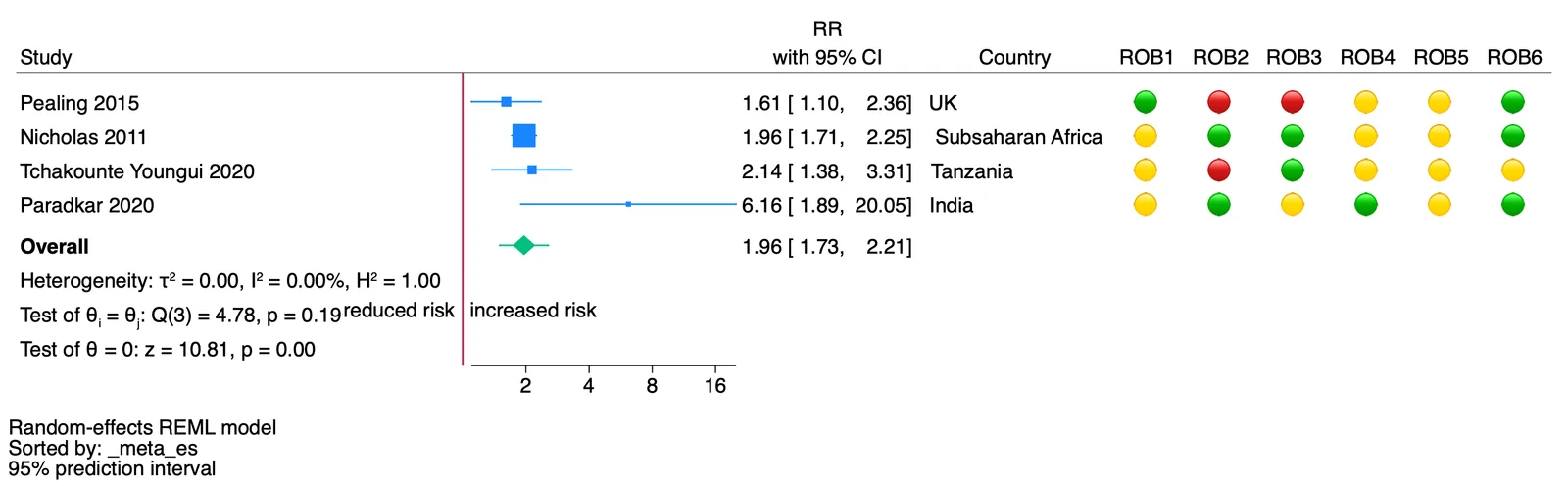
**Risk ratios for the risk of incident TB disease due to undernutrition**. The blue squares represent the risk ratios of the individual studies. The green diamond represents the meta‐analysis, and its width represents the confidence interval. The green horizontal line emanating from the diamond represents the prediction interval (which can be found reported in the main text). ROB1: Study Participation; ROB2: Study Attrition; ROB3: Prognostic Factor Measurement; ROB4: Outcome Measurement; ROB5: Study Confounding; ROB6: Statistical Analysis and Reporting. The green, yellow, and red circles in each ROB column represent low, moderate, and high risk of bias, respectively.

##### Subgroup analyses

Although we decided not to downgrade for inconsistency due to the distribution of effects, we found high statistical heterogeneity. Therefore, we conducted the pre‐specified subgroup analyses using the entire body of evidence, including studies on subpopulations for the outcomes reported as HR, as they provided enough studies to conduct such analyses. Table already presents analysis by type of outcome measures (HR, RR, OR).

###### Age and sex

*Across studies*

As no studies were conducted in only one sex, we could not analyse study‐level differences according to sex. Nonetheless, we performed a subgroup analysis based on whether the studies were conducted in adults, children or both, and we could not identify differences across estimates (P value = 0.39, see Figure).

**7:**
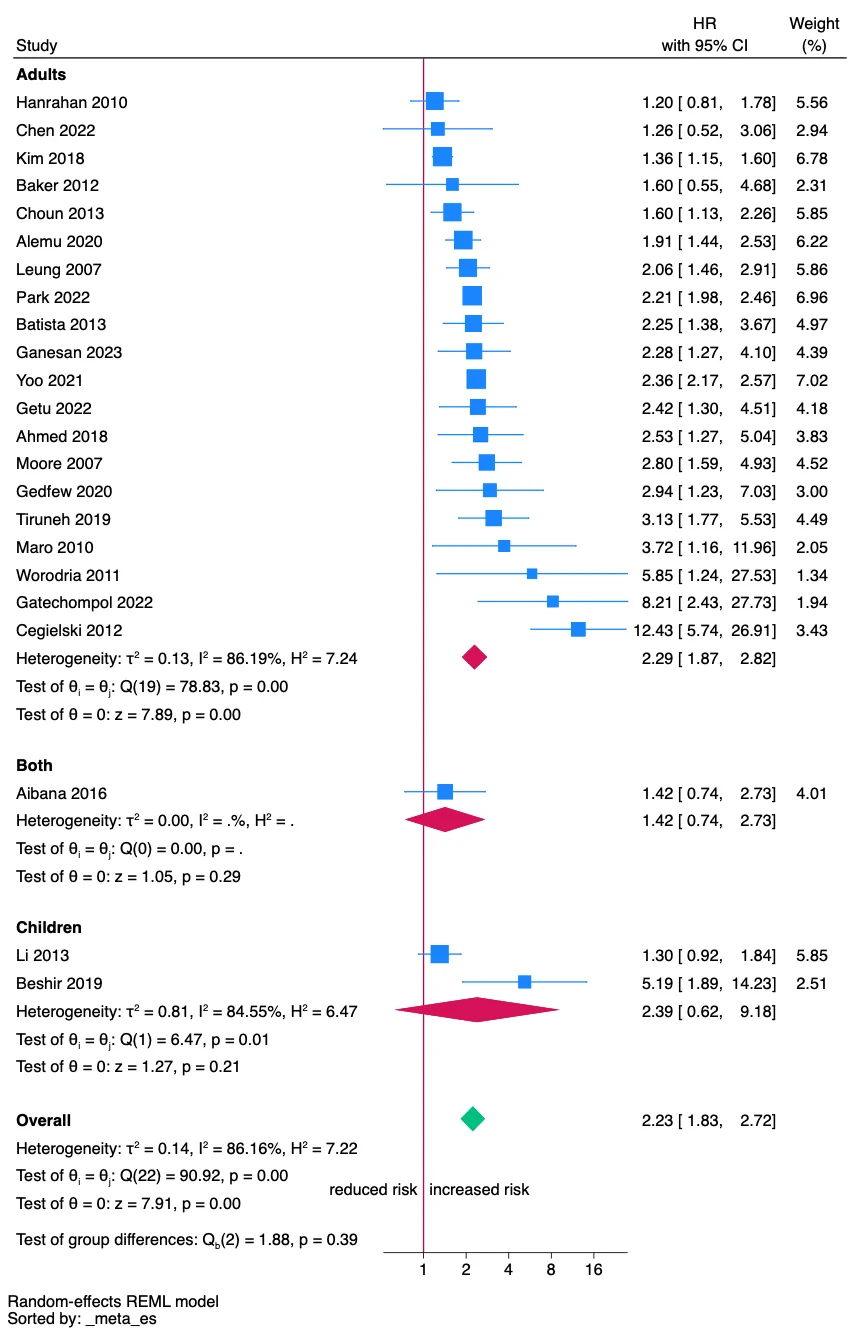
**Subgroup analysis by age groups for the risk of incident TB disease due to undernutrition.** The blue squares represent the hazard ratios of the individual studies. The red diamond represents the meta‐analysis for each subgroup, the green diamond represents the overall meta‐analysis, and their width represents the confidence interval.

*Within studies*

We extracted subgroup data on age and sex for the included studies. We could not conduct subgroup analyses according to age due to the different effect measures and age ranges in each category. Nonetheless, the within‐study analysis indicated that most studies did not find differences across ages, except Park 2022, which identified a gradient of increased risk across age groups (P value = 0.01, see Figure).

**8:**
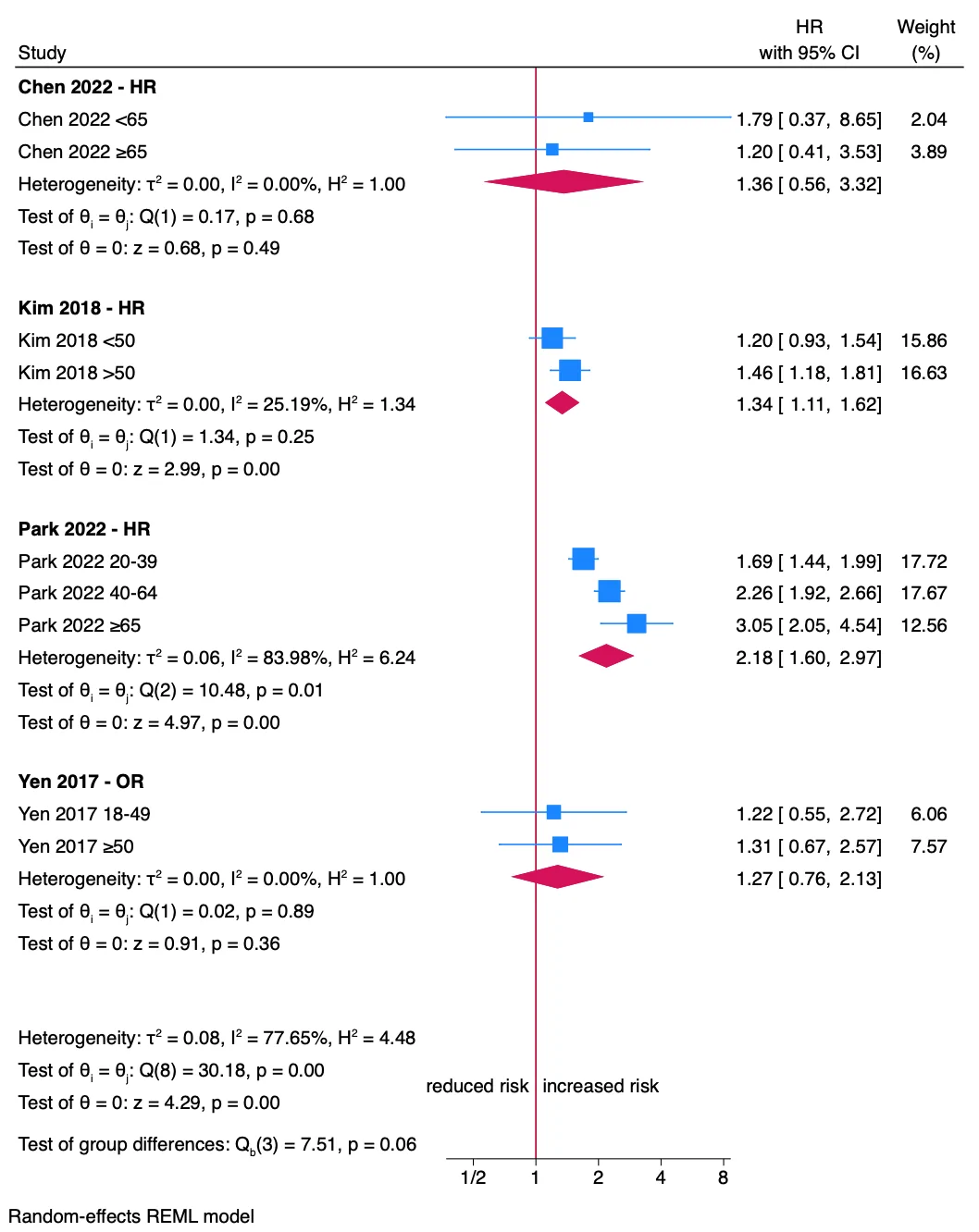
**These are the subgroups according to age reported in each individual study for the risk of incident TB disease due to undernutrition.** As the studies had different definitions for age groups, these subgroups could not be pooled and were reported separately. Only one study indicated subgroup differences across the three age categories (Park 2022). The blue squares represent the hazard ratios of the individual studies. The red diamond represents the meta‐analysis for each subgroup, and their width represents the confidence interval.

As for sex, although the effect measure for men was higher than for women (HR 1.96, 95% CI 1.31 to 2.93 for men, HR 1.29, 95% CI 1.02 to 1.64 for women), we could not find differences across subgroups (P value = 0.08, see Figure).

**9:**
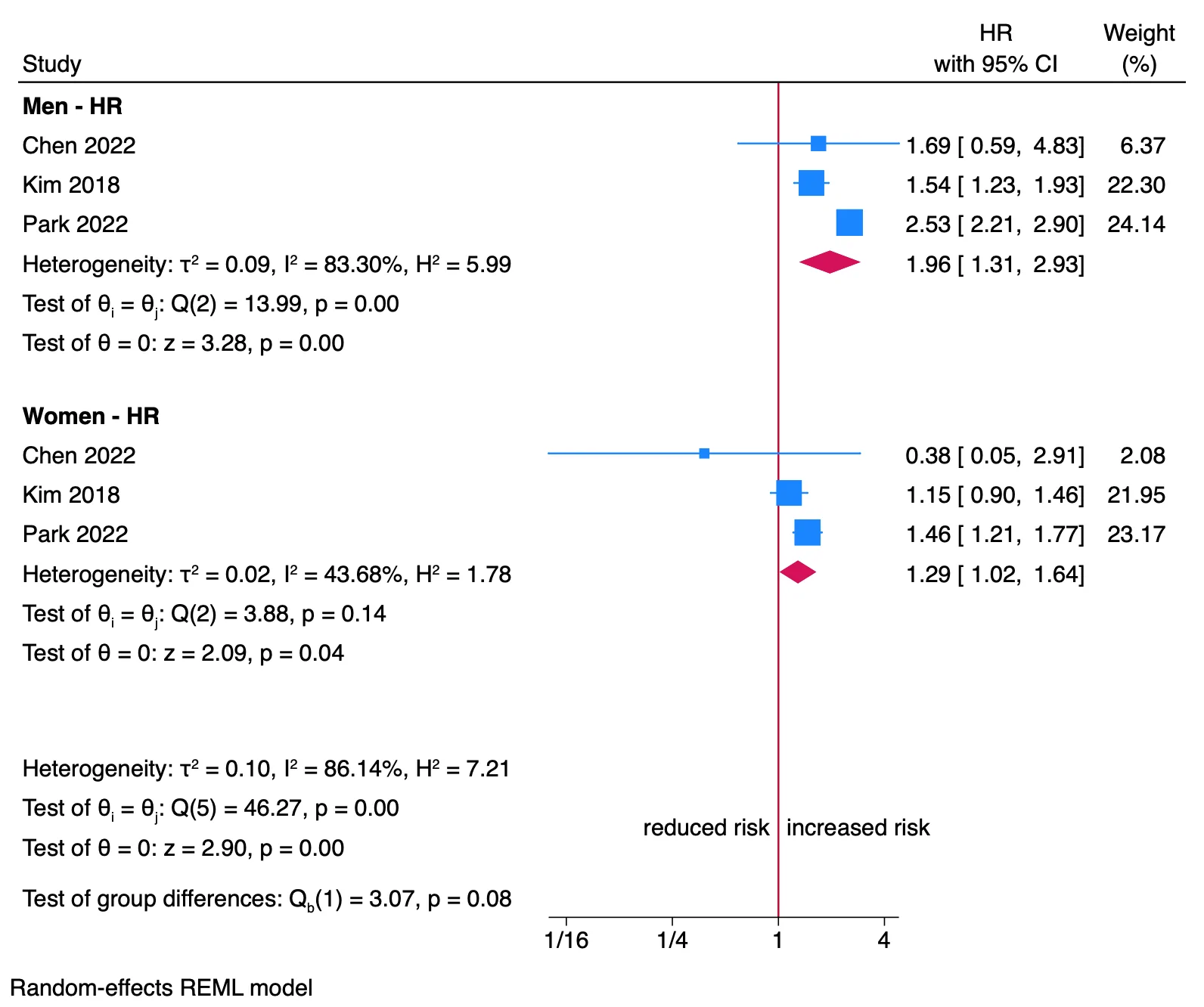
**Subgroup analysis by sex for the risk of incident TB disease due to undernutrition.** The blue squares represent the hazard ratios of the individual studies. The red diamond represents the meta‐analysis for each subgroup, and their width represents the confidence interval.

One study reported subgroup analysis for sex and age but for recurrent TB (Youn 2022) (Figure). Numerically, adjusted estimates were reported for men (HR 2.34, 95% CI 1.30 to 4.24), individuals younger than 40 years old (HR 2.52, 95% CI 1.15 to 5.51), and older than 60 years old (HR 3.25, 95% CI 1.28 to 8.25). No P values are reported, but confidence intervals across subgroups overlap, and no differences could be detected.

**10:**
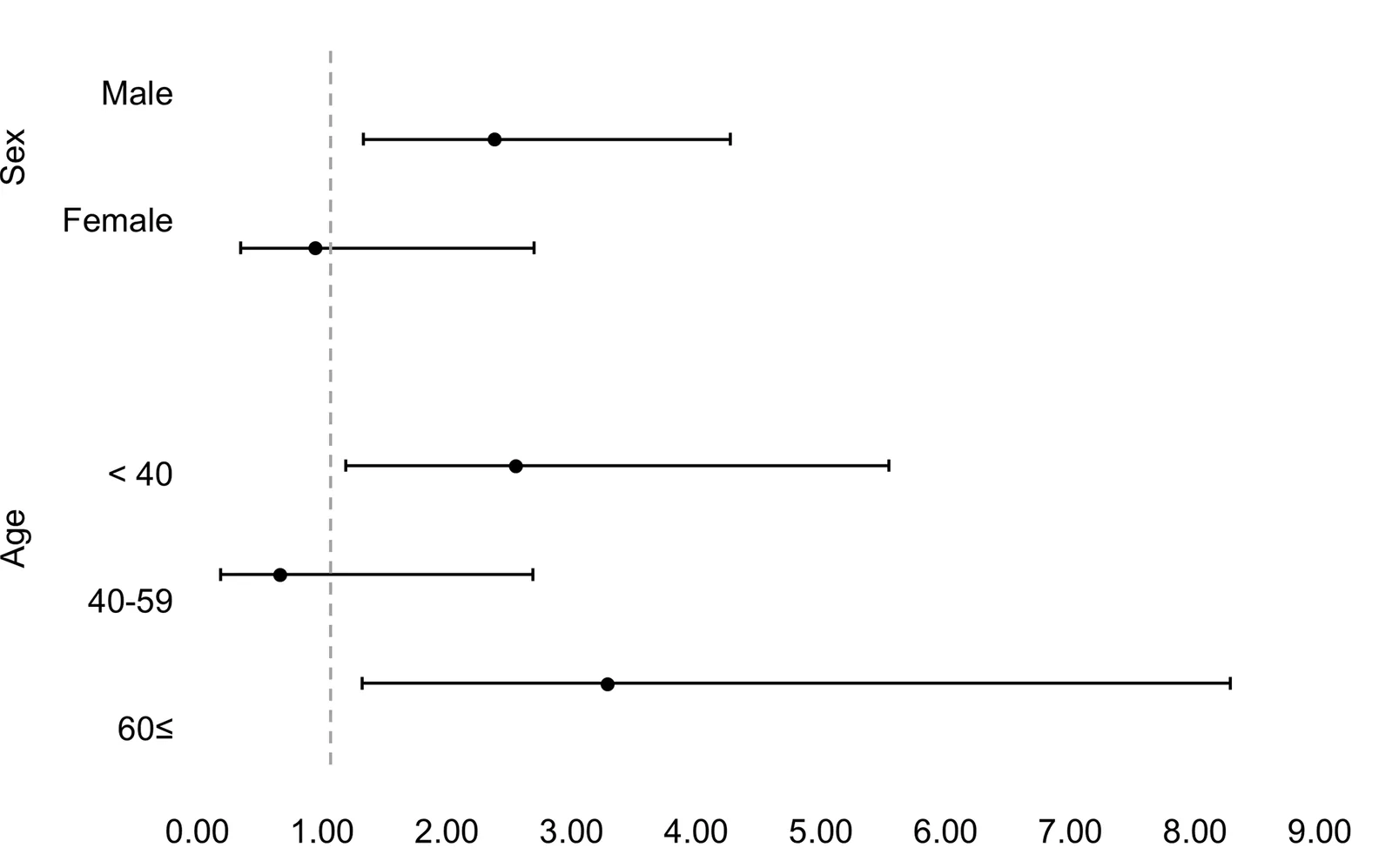
**Subgroup analysis for the recurrence of TB.** Figure from Youn 2022 (Creative Commons Attribution Licence). The round dot represents the point estimate and the horizontal lines the confidence intervals. The dotted vertical line, the line of no effect (RR = 1).

###### HIV status or immunosuppression status

We conducted a subgroup analysis across studies focused on participants with HIV (HR 2.21, 95% CI 1.75 to 2.78; I^2^ = 59.16%) versus those studies on the general population (HR 2.21, 95% CI 1.48 to 3.31; I^2^ = 96.03%), and we could not identify differences across estimates (P value = 0.99, see Figure).

**11:**
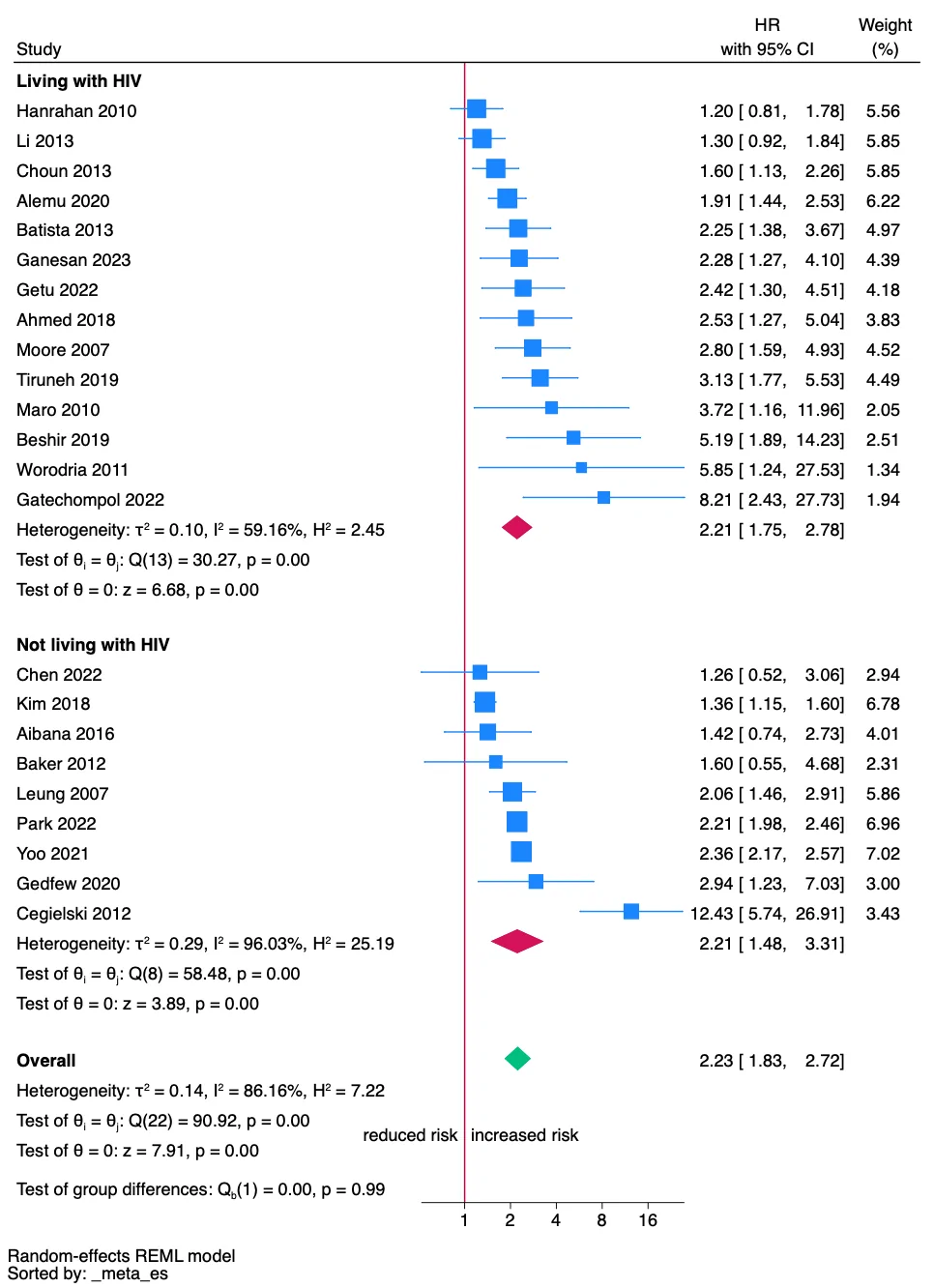
**Subgroup analysis by HIV status for the risk of incident TB disease due to undernutrition.** The blue squares represent the hazard ratios of the individual studies. The red diamond represents the meta‐analysis for each subgroup, the green diamond represents the overall meta‐analysis, and their width represents the confidence interval.

###### Socioeconomic status

There were insufficient data to conduct this subgroup analysis.

###### Local/geographical TB incidence disease

There were insufficient data for this subgroup analysis based on the local TB incidence (high versus low incidence).

###### Definition and degree of undernutrition

We conducted a subgroup analysis across studies focused on participants with severe malnutrition versus moderate malnutrition, and we could not identify differences across estimates (P value = 0.26, see Figure). Severe malnutrition was defined in the studies as BMI < 16 (Chang 2015), < 17 (Liu 2015), or weight‐for‐height < ‐3 SD (Beshir 2019; Li 2013).

**12:**
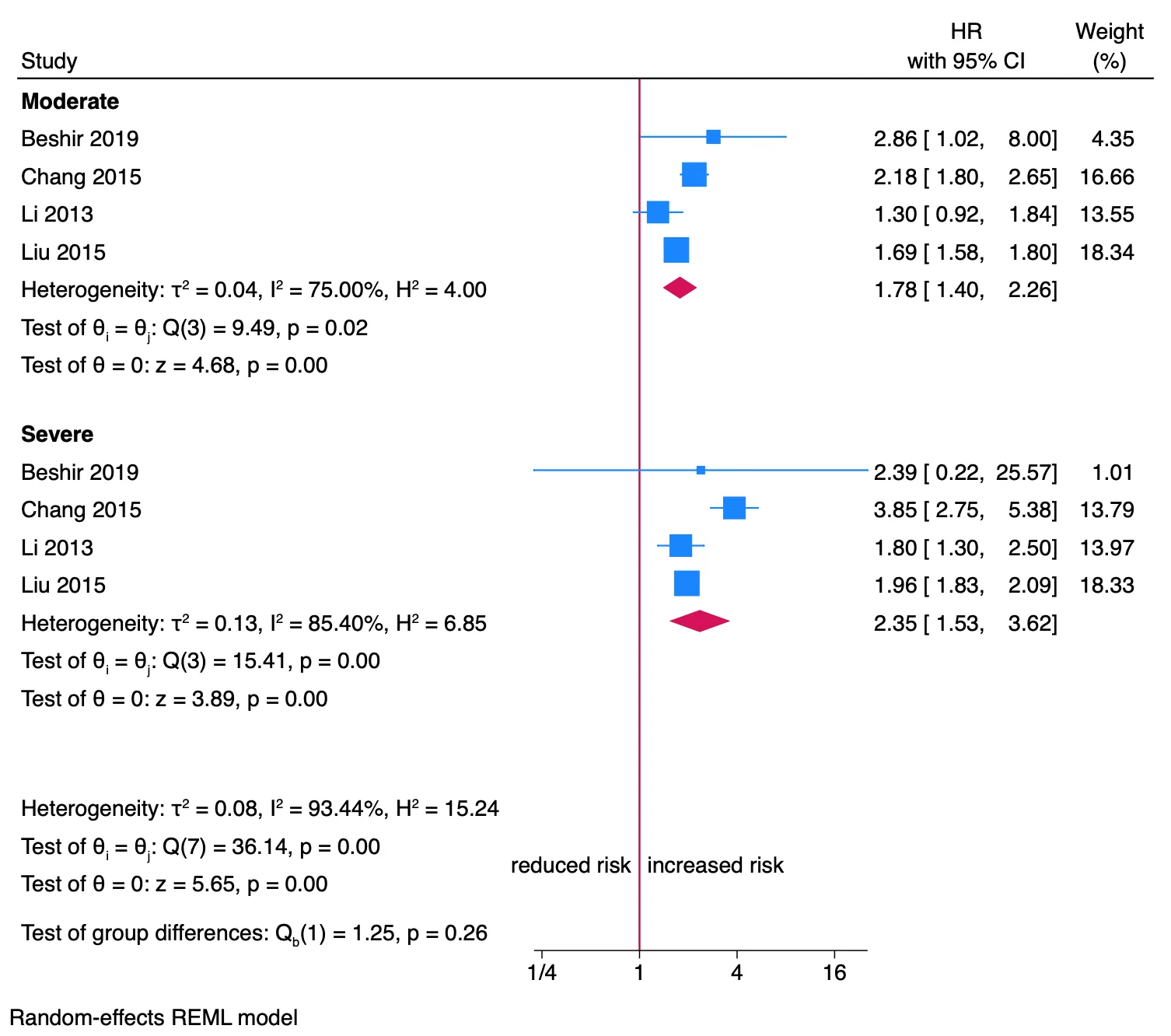
**Subgroup analysis by degree of undernutrition for the risk of incident TB disease due to undernutrition**. Severe malnutrition was defined in the studies as BMI < 16 (Chang 2015), < 17 (Liu 2015), or weight‐for‐height < ‐3 SD (Beshir 2019; Li 2013). The blue squares represent the hazard ratios of the individual studies, the red diamond represents the meta‐analysis for each subgroup, and their width represents the confidence interval.

As the BMI range of the comparison group with which the people with undernutrition were compared varied considerably between studies, we conducted a post hoc subgroup analysis. We analysed the subgroup of studies that included people with normal weight (i.e. BMI between 18.5 and 25 kg/m^2^) as a reference group and those that had a comparison group of "no undernutrition" (i.e. BMI > 18.5 kg/m^2^, which also includes people with overweight and obesity) as a reference group. We could not identify differences across estimates (P value = 0.26; see Figure).

**13:**
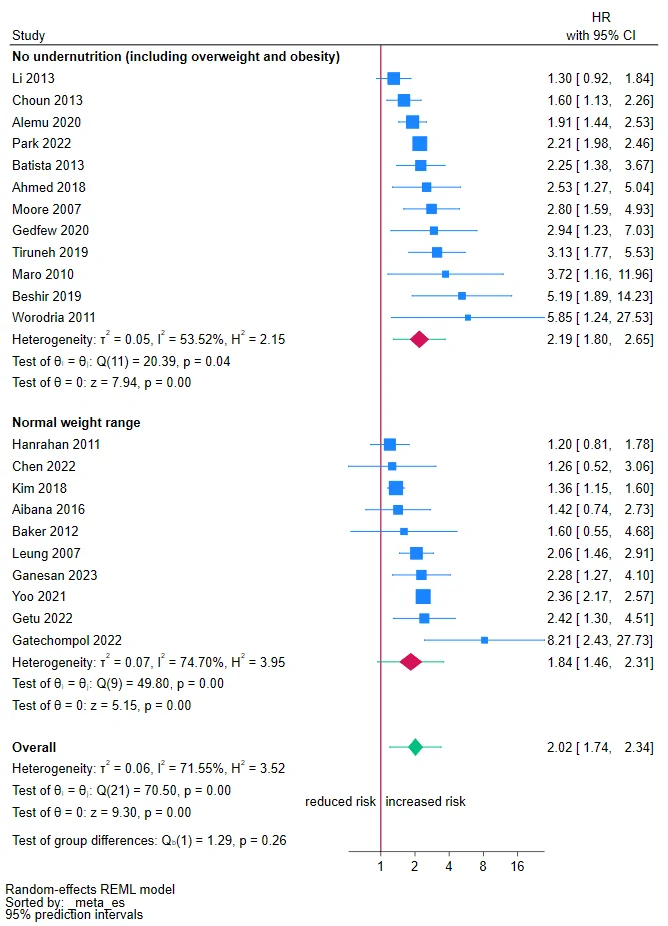
**Subgroup analysis for the risk of incident TB disease due to undernutrition based on comparison/reference group:** Studies comparing underweight vs no undernutrition as a reference group (usually BMI > 18.5 kg/m^2^, including overweight and obesity) and studies comparing underweight with a normal weight range as a reference group (usually BMI 18.5 to 25 kg/m^2^). The blue squares represent the hazard ratios of the individual studies, the red diamond represents the meta‐analysis for each subgroup, the green diamond represents the overall meta‐analysis, and their width represents the confidence interval.

###### Sensitivity analyses

In our protocol, we defined that our primary analyses would be based on studies with a low risk of bias in some domains of the risk of bias tool QUIPS and that further sensitivity analyses on other domains would be explored subsequently. Supplementary material 8 describes the different sensitivity analyses for HR, including 95% CIs and prediction intervals. Estimates were robust for all 'overall' and '< 10 years' estimates. The long‐term estimates were sensitive to bias, i.e. depending on the domain of the QUIPS tool that was selected as 'low risk of bias', the results varied substantially. Each analysis restricting studies to a low risk of bias in one domain included studies with a high risk of bias in another domain. Therefore, the certainty of the evidence would have been lower, given the need to downgrade two levels due to the risk of bias. We chose, therefore, an alternative approach by excluding studies at high risk of bias, retaining precision and downgrading by one level the certainty of the evidence due to moderate risk of bias for the 'overall' and '< 10 years' estimates. Sensitivity analysis for RR and OR was not possible due to the few studies reporting on these estimates.

We also explored the effect of the decision to report a single estimate < 10 years follow‐up by stratifying this estimate into three categories using two and five years as cut‐off points. This stratification yielded consistent results across these three categories (See Figure).

**14:**
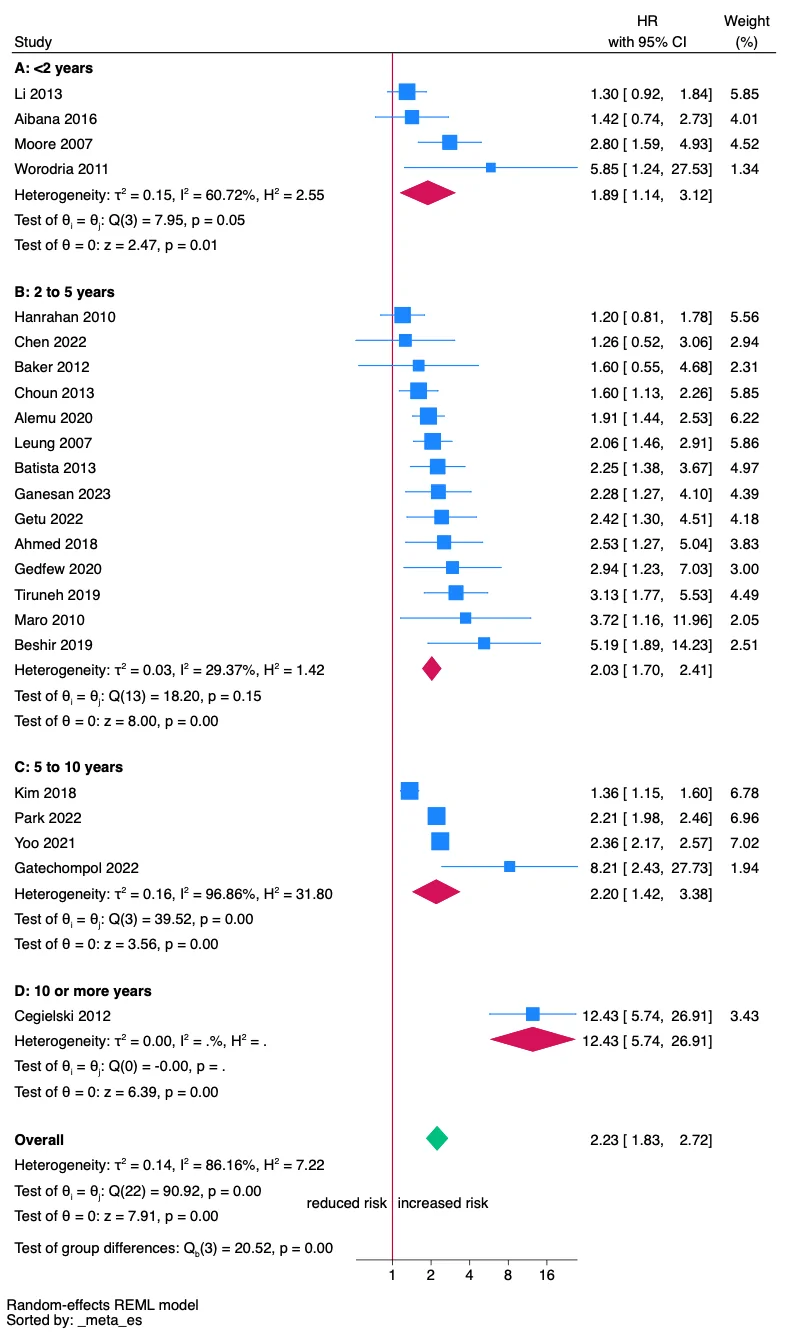
**Subgroup analysis stratified by different time point categories for the risk of incident TB disease due to undernutrition**: < 2 years, 2 to 5 years, 5 to 10 years, and 10 or more years. The blue squares represent the hazard ratios of the individual studies, the red diamond represents the meta‐analysis for each subgroup, the green diamond represents the overall meta‐analysis, and their width represents the confidence interval.

#### Recurrent tuberculosis

Only two studies with 3041 participants reported this outcome (Anaam 2020; Youn 2022). Undernutrition may result in a higher risk of recurrence (HR 1.92, 95% CI 1.15 to 3.20 and OR 1.66, 95% CI 0.86 to 3.20, see Figure). Contour‐enhanced funnel plots were not reported due to the few studies included.

**15:**
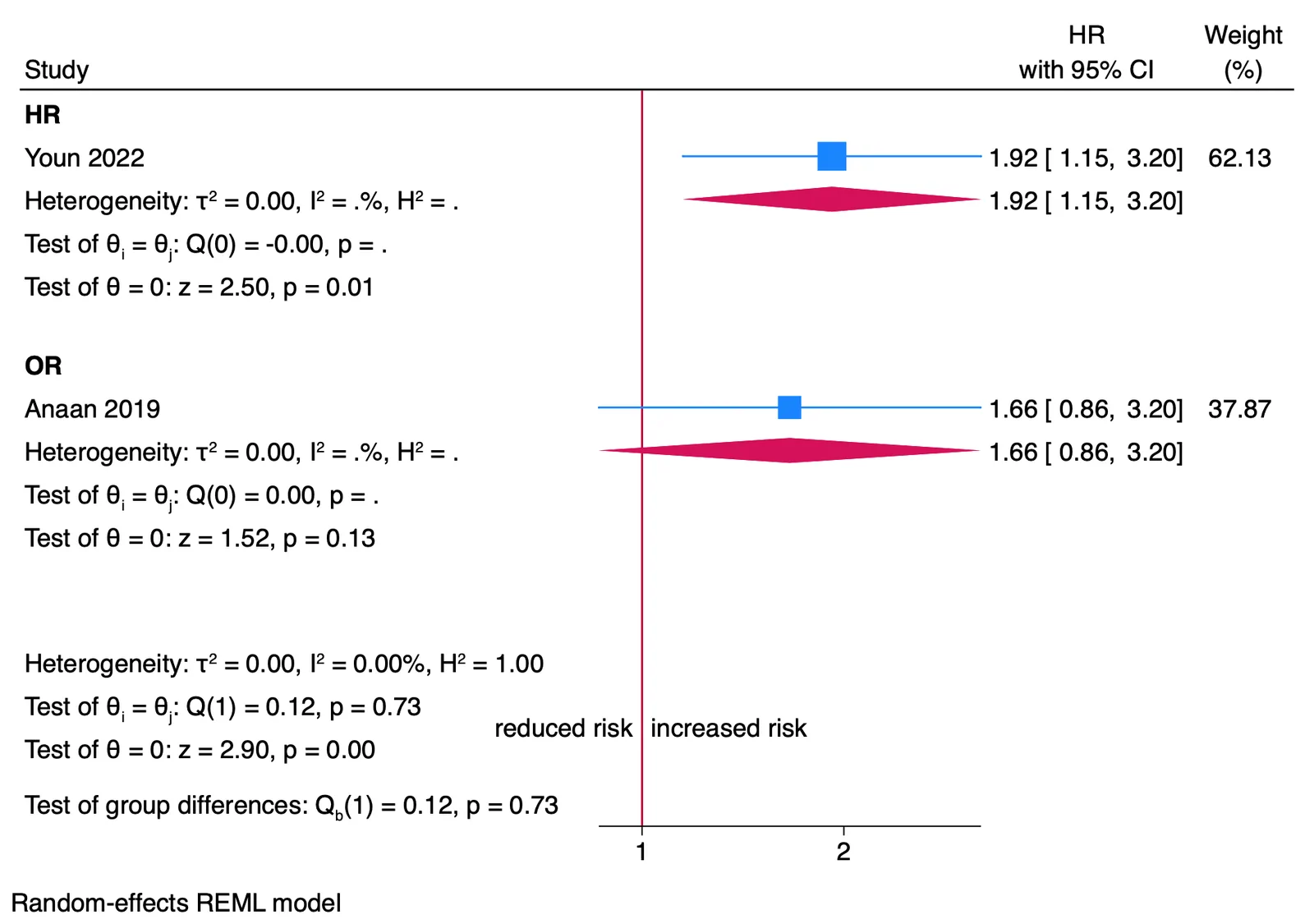
**Risk of recurrent TB disease due to undernutrition** (**HR:** hazard ratio; **RR:** risk ratio). The blue squares represent the ratios of the individual studies, the red diamond represents the meta‐analysis for each subgroup, and their width represents the confidence interval.

## Discussion

### Summary of the main results

We included 51 cohort studies with over 27 million participants from all six WHO regions. Nearly half of the studies focused on people living with HIV, and one‐third included large national databases or population‐based studies. Only a few studies included children or adolescents. The median follow‐up time was 3.5 years, and most studies reported a multivariable Cox‐proportional hazard estimate using a different set of covariates.

Overall, undernutrition probably increases the risk of TB two‐fold, and the certainty of the evidence is moderate. However, long‐term follow‐up studies (> 10 years) provided inconsistent estimates, with low certainty of the evidence. Exploration of heterogeneity using subgroup analysis yielded inconclusive results. Similar increases in the risk of recurrent tuberculosis were identified in two studies, but the evidence was scarce.

### Overall completeness and applicability of the evidence

The geographical representation of the places with the highest burden of TB is only partially represented in the current body of evidence. Even if the largest studies were conducted in Africa, the Western Pacific Region, and the Southeast Asia Region, there were no large studies in the Americas, especially South America, where the burden of TB remains significant [1]. Furthermore, the large population‐based studies were conducted in China, Singapore, South Korea, and the USA, and 22 out of the 23 studies conducted in the African region were on people living with HIV and not the general population. Moreover, few studies assessed the risk of undernutrition in children, with inconsistent results, even though they represented 12% of the new cases in 2022 [1]. Children usually have a higher risk of developing TB, including severe forms; therefore, the differential effects of undernutrition need to be further investigated in children (18).

Undernutrition as exposure was usually standardly defined; however, the diagnosis and exclusion of TB were usually not performed using culture or a molecular WHO‐recommended rapid diagnostic test (mWRD), which could have biased the estimate of the effect. However, clinical diagnosis has an important role, as some people with reduced immunity or paucibacillary disease may be unable to provide sputum. The diagnosis of TB has evolved, and the WHO consolidated guidelines on TB diagnosis recommend a mWRD as the initial diagnostic test, while culture remains the gold standard for TB diagnosis [17]. Both culture and molecular methods were rarely used in the included studies; therefore, the applicability of the findings might need to be adjusted to new evidence on the true incidence of TB disease based on more accurate diagnostic technologies. Overall, current diagnostic tools, including molecular WHO‐recommended rapid diagnostic tests and culture, are less sensitive for people living with HIV and for children; hence, TB incidence may have been underestimated in these populations [17, 18].

Subgroup analysis did not detect differences in the risk estimates in people living with HIV. This might be because the included studies in this subgroup included participants who received treatment for HIV infection and were offered, when eligible, TB preventive treatment, both contributing to lower risk estimates, similar to the general population (99; 100). Similarly, five of the studies included household contacts, which might have influenced the effect due to household contacts receiving TB preventive treatment. Two of these studies reported TB preventive treatment being given to participants, while three of the studies did not report whether participants received TB preventive treatment (Aibana 2016; Benjumea‐Bedoya 2019; Morán‐Mendoza 2010; Okwara 2017; Paradkar 2020).

The proportion of people with moderate and severe undernutrition in the countries represented in this review is also variable. Whereas classification of countries into high‐income (HIC) and low‐ and middle‐income (LMIC) may be useful for some analysis, it does not accurately reflect differences in the prevalence of undernutrition as the LMICs from the Latin America region have a much lower prevalence than the African region (101); therefore a stratified analysis would not be helpful to ascertain the differential effect of severe malnutrition, and we rely on subgroup analysis for severe versus moderate malnutrition. Unfortunately, subgroup analysis also could not detect differences in the risk estimates between those with moderate and severe undernutrition. However, these results must be interpreted cautiously, as this analysis may be underpowered. Previous research, which considered the wider spectrum of BMI from undernutrition to obesity, indicated an inverse linear relationship between BMI and TB disease [8].

The results of this review will be used to calculate the annual population attributable fraction (PAF) of undernutrition for TB in the WHO Global Tuberculosis Report. Even when we produced overall and follow‐up stratified estimates in this review, it remained a challenge to identify the best estimate for applying this calculation, considering the small variations across effect measures (HR, RR, OR) and the composition of populations. Populations with a higher degree of undernutrition in children might not have a reliable estimate for the calculation of PAF based on the small number of studies in our review. Moreover, case‐mix populations of adults, people with immunosuppression from HIV, and other risk factors may pose other challenges in estimating an accurate PAF with the results of this review. Upcoming Cochrane reviews on other risk factors (e.g. diabetes) will inform updated PAF estimates of the WHO Global Tuberculosis Report.

### Certainty of the evidence

The certainty of the evidence was mostly moderate for the large body of evidence contributing to the estimates based on 0 to 10 years of follow‐up. The main reason for downgrading the certainty of the evidence was a moderate risk of bias, considering the study limitations, primarily due to the limitations in the adjusted models in terms of variables included and the assessment of the outcome (lack of confirmation by culture or other methods). Additionally, a common reason for downgrading the certainty of the evidence was inconsistency, as the prediction intervals for some estimates were wide and included the null. We decided to downgrade due to indirectness only for one study that yielded substantially different results in the long term (≥ 10 years), considering that this study was conducted between 1971 and 1992 (Cegielski 2012), where the characteristics of the population and context of outcome assessment are substantially different to current practice.

We did not downgrade due to imprecision, as the effect estimates and confidence intervals substantially excluded the scenario of null risk, which is the minimally contextualised approach suggested for GRADEing prognostic research [44, 45]. Moreover, we were unable to identify a strong suspicion of publication bias in our contour‐enhanced funnel plot (Figure). We did not consider upgrading the certainty of the evidence as we could not identify a large unequivocal effect or a confirmed dose‐response gradient [45].

### Potential bias in the review process

Our review has several limitations. Firstly, we restricted our analysis to studies of low to moderate risk of bias, which is a small deviation from our protocol, in which we specified that our analysis would be based on studies with low risk of bias in specific domains of the QUIPS tool. We explored the effects of this decision through sensitivity analyses, and we believe that the current estimates provide a higher certainty of the evidence in terms of minimising bias and maximising precision. Secondly, we could not explain statistical heterogeneity through subgroup analysis and had scarce data to run meta‐regression. Furthermore, we had limited data on some outcomes (OR and RR) to conduct sensitivity analyses related to the risk of bias. Thirdly, we initially set out to analyse unadjusted and adjusted estimates; however, considering the large volume of information, we decided to restrict our analysis to studies with some level of adjustment for covariables. Fourth, we had planned to conduct subgroup analysis based on the type of outcome data, but we presented these data separately (HR, RR, and OR). Therefore, these subgroup analyses were not possible. Fifth, we might have missed additional studies focused on other risk factors that also assessed undernutrition as part of their derivation of multivariate models. Finally, we initially planned to assess the recurrence of TB as an outcome; however, considering our inclusion criteria (individuals free from TB at baseline), we might not have been able to capture the right study design for this outcome. Instead, a review focusing on the long‐term follow‐up of people with TB disease might provide a more comprehensive assessment of this outcome [19].

### Agreements and disagreements with other reviews and studies

The previous systematic review informing the WHO Global Tuberculosis Report provided an extensive bibliographic search until 2008, including six longitudinal studies, four of which did not include individuals with undernutrition and, therefore, were not included in this review. The previous review calculated the linear relationship between BMI and the risk of TB [8]. The previous calculation was based on the average risk reduction of 13.8% per BMI unit derived from this meta‐analysis, resulting in a relative risk of 3.2 for a BMI of 16 kg/m^2^ versus 25 kg/m^2^ [9]. Recent estimations of the population attributable fraction (PAF) have used an even higher risk ratio estimate of 4.49 from one of our included studies (Cegielski 2012), which contributed to the indirectness of the body of the evidence in our review (see 'Certainty of the evidence' above) [102]. These calculations differ from our review results: our review indicates a hazard ratio/risk ratio closer to 2 for individuals with a BMI < 18.5 kg/m^2^ compared to those with a BMI of 18.5 to 25 kg/m^2^.

Nonetheless, two of the studies included in the previous review were included in our review (Cegielski 2012; Leung 2007), and the findings are concordant with ours, highlighting the association of higher risk for TB with lower BMI values. Still, considering the severity of undernutrition, we could not prove a dose‐response relationship in our subgroup analysis, but our analysis was likely underpowered.

## Authors' conclusions

Undernutrition probably increases the risk of TB two‐fold in the short term (< 10 years) and may also increase the risk in the long term (> 10 years). Policies targeted towards the reduction of the burden of undernutrition are not only needed to alleviate human suffering due to undernutrition and its many adverse consequences, but are also an important part of the critical measures for ending the TB epidemic by 2030.

Large population‐based cohorts, including those derived from high‐quality national registries of exposures (undernutrition) and outcomes (TB disease), are needed to provide high‐certainty estimates of this risk across different settings and populations, including low‐ and middle‐income countries from different WHO regions. Moreover, studies including children and adolescents and state‐of‐the‐art methods for diagnosing TB would provide more up‐to‐date information relevant to practice and policy.

## Supporting Information

Supplementary materials are available with the online version of this article: 10.1002/14651858.CD015890.pub2.

Supplementary materials are published alongside the article and contain additional data and information that support or enhance the article. Supplementary materials may not be subject to the same editorial scrutiny as the content of the article and Cochrane has not copyedited, typeset or proofread these materials. The material in these sections has been supplied by the author(s) for publication under a Licence for Publication and the author(s) are solely responsible for the material. Cochrane accordingly gives no representations or warranties of any kind in relation to, and accepts no liability for any reliance on or use of, such material.

**Supplementary material 1** Search strategies

**Supplementary material 2** Characteristics of included studies

**Supplementary material 3** Characteristics of excluded studies

**Supplementary material 4** Characteristics of studies awaiting classification

**Supplementary material 5** Characteristics of ongoing studies

**Supplementary material 6** Data package

**Supplementary material 7** Overlap of study populations

**Supplementary material 8** Sensitivity Analyses

## Figures and Tables

**Table:** 

**Date**	**Event**	**Description**
7 February 2025	Amended	Correction to data. The authors of one study (Tchakounte Youngui 2020) subsequently provided the disaggregated data for the subgroup of BMI <18.5 (previously <20), which fits best with the inclusion criteria of the review. The new analysis warranted a correction: ‐ Published risk ratio: RR 1.95, 95% CI 1.72 to 2.20; prediction interval 1.49 to 2.55 ‐ Corrected risk ratio: RR 1.96, 95% CI 1.73 to 2.21; prediction interval 1.50 to 2.56

## Additional information

### Registration and protocol

PROSPERO registration: CRD42023408807. The protocol was published in the Cochrane Database of Systematic Reviews [103].

### Data, code and other materials

All the data related to this review are available at the Open Science Framework (https://osf.io/ncwvf/) and in Supplementary material 6.

### Notes

The methods of the protocol are based on a previous protocol co‐authored by the lead author of the protocol [104] and a recently published review [105].

### History

Protocol first published: Issue 10, 2023 Review first published: Issue 6, 2024
