# Supplementary material for: Undernutrition as a risk factor for tuberculosis disease
Source: Cochrane Database Syst Rev. 2024 Jun 11;2024(6):CD015890. doi: 10.1002/14651858.CD015890.pub2 (PMC11165671; doi:10.1002/14651858.CD015890.pub2)
Supplement: Supplementary file 1 — Supplementary material 1 Search strategies [file CD015890-SUP-01-searchStrategy.html]

Search strategies


# Supplementary material 1 to: Undernutrition as a risk factor for tuberculosis disease

Franco JVA, Bongaerts B, Metzendorf MI, Risso A, Guo Y, Peña Silva L, Boeckmann M, Schlesinger S, Damen JAAG, Richter B, Baddeley A, Bastard M, Carlqvist A, Garcia-Casal MN, Hemmingsen B, Mavhunga F, Manne-Goehler J, Viney K
  
https://doi.org/10.1002/14651858.CD015890.pub2

The material in this section has been supplied by the author(s) for publication under a Licence for Publication and the author(s) are solely responsible for the material. Cochrane has peer reviewed this material in accordance with its editorial policies, but Cochrane has not copyedited, formatted or proofread. Cochrane accordingly gives no representations or warranties of any kind in relation to, and accepts no liability for any reliance on or use of, such material.

Back to top

# Search strategies

## Search strategy

**MEDLINE (PubMed)**

#1 tubercul\*[tw]

#2 diabet\*[tw] OR BMI[tiab] OR body mass index[tiab] OR underweight[tiab] OR malnutri\*[tw] OR undernutri\*[tiab] OR wasting[tw] OR undernourish\*[tiab] OR malnourish\*[tiab] OR Kwashiorkor\*[tw] OR nutritional deficien\*[tiab]

#3 risk[tw] OR prevalence[tw] OR incidence[tw] OR cohort[tw]

#4 #1 AND #2 AND #3  
(= 3,811)

**WHO Global Index Medicus**

Title, abstract subject: tubercul\* AND (diabet\* OR BMI OR "body mass index" OR underweight OR malnutri\* OR undernutri\* OR wasting OR undernourish\* OR malnourish\* OR Kwashiorkor\* OR "nutritional deficien\*")  
(= 1,438)

**WHO ICTRP (Standard search)**

tubercul\* AND (diabet\* OR BMI OR "body mass index" OR underweight OR malnutri\* OR undernutri\* OR wasting OR undernourish\* OR malnourish\* OR Kwashiorkor\* OR "nutritional deficien\*")

(= 75)

**PubMed IDs of 43 relevant articles used to develop the search strategy**

3792471[PMID] OR 4992917[PMID] OR 8593374[PMID] OR 11532111[PMID] OR 13470303[PMID] OR 15220232[PMID] OR 16913973[PMID] OR 17278679[PMID] OR 17592104[PMID] OR 18400769[PMID] OR 18790239[PMID] OR 20505496[PMID] OR 20819255[PMID] OR 21109542[PMID] OR 22238171[PMID] OR 22331390[PMID] OR 22571347[PMID] OR 22791739[PMID] OR 23343909[PMID] OR 23510593[PMID] OR 23554875[PMID] OR 23631563[PMID] OR 24236069[PMID] OR 24278293[PMID] OR 24365557[PMID] OR 25310745[PMID] OR 25478954[PMID] OR 25884596[PMID] OR 26048371[PMID] OR 26459528[PMID] OR 27986673[PMID] OR 28169875[PMID] OR 29437750[PMID] OR 31319659[PMID] OR 31557282[PMID] OR 31931911[PMID] OR 32476692[PMID] OR 34926543[PMID] OR 35387594[PMID] OR 35709199[PMID] OR 35921384[PMID] OR 36171396[PMID] OR 36269203[PMID]
