# Supplementary material for: Undernutrition as a risk factor for tuberculosis disease
Source: Cochrane Database Syst Rev. 2024 Jun 11;2024(6):CD015890. doi: 10.1002/14651858.CD015890.pub2 (PMC11165671; doi:10.1002/14651858.CD015890.pub2)
Supplement: Supplementary file 2 — Supplementary material 2 Characteristics of included studies [file CD015890-SUP-02-characteristicsOfIncludedStudies.html]

Characteristics of included studies


# Supplementary material 2 to: Undernutrition as a risk factor for tuberculosis disease

Franco JVA, Bongaerts B, Metzendorf MI, Risso A, Guo Y, Peña Silva L, Boeckmann M, Schlesinger S, Damen JAAG, Richter B, Baddeley A, Bastard M, Carlqvist A, Garcia-Casal MN, Hemmingsen B, Mavhunga F, Manne-Goehler J, Viney K
  
https://doi.org/10.1002/14651858.CD015890.pub2

The material in this section has been supplied by the author(s) for publication under a Licence for Publication and the author(s) are solely responsible for the material. Cochrane has reviewed this material, but Cochrane has not copyedited, formatted or proofread. Cochrane accordingly gives no representations or warranties of any kind in relation to, and accepts no liability for any reliance on or use of, such material.

Back to top

# Characteristics of included studies

## Table of contents

- Studies ordered by Study ID
  - Ahmed 2018
  - Aibana 2016
  - Alemu 2020
  - Anaam 2020
  - Ayana 2021
  - Baker 2012
  - Batista 2013
  - Benjumea-Bedoya 2019
  - Beshir 2019
  - Cegielski 2012
  - Chang 2015
  - Chan-Yeung 2007
  - Chen 2022
  - Cheng 2020
  - Cho 2022
  - Choi 2021
  - Choun 2013
  - Dembélé 2010
  - Ganesan 2023
  - Gatechompol 2022
  - Gedfew 2020
  - Getu 2022
  - Hanrahan 2010
  - Jung 2016
  - Kim 2018
  - Kyaw 2022
  - Leung 2007
  - Li 2013
  - Lin 2018
  - Liu 2015
  - Long 2020
  - Maro 2010
  - Moore 2007
  - Morán-Mendoza 2010
  - Nicholas 2011
  - Okwara 2017
  - Paradkar 2020
  - Park 2022
  - Park 2023
  - Pealing 2015
  - Sabasaba 2019
  - Soh 2019
  - Sudfeld 2013
  - Tchakounte Youngui 2020
  - Tiruneh 2019
  - Van Rie 2011
  - Were 2009
  - Worodria 2011
  - Yen 2017
  - Yoo 2021a
  - Youn 2022
- Footnotes
- References to studies

## Studies ordered by Study ID

Ahmed 2018

| ***Study characteristics*** | | |
| Study details and sources | **Study design:** Retrospective cohort study  **Source of data:** Clinical Health Records  **Study dates:**2010-2015  **Setting:** Health centres and hospitals (two centres in Awash and Samara and three hospitals in Asayta, Abala and Dubti General)  **Countries:** Ethiopia | |
| Participants | **Participant eligibility:** patients with HIV/AIDS aged 15 years and above and newly enrolled for HIV care with complete information on date of enrolment, outcome of interest and follow-up data.  **Recruitment method:** All patients, as described above, in selected government health facilities in the Afar Region from July 2010 to June 2011, participated in the study.  **Participant Description:**   - All living with HIV - Age: mean age (SD) 32.6 (7.5) - Sex: 59.2% female   **Risk factors:**   - diabetes: not described - undernutrition: <18.5 51.7% - HIV infection: all participants - recent TB infection: previous TB disease 16.4% - history of untreated or inadequately treated TB disease: N/A - Inmunesupression: all with HIV infection: WHO clinical stage I and II 45.4%, CD4 >350 35% - cigarette smokers: 14% - drug or alcohol use disorders: 26% alcohol use - socioeconomic status: 47% illiterate, 38% non-employed - other:   - chronic illness 7.8%   - isoniazid preventive therapy 20.8%   **Details of treatments received (treatment for diabetes, undernutrition and other concomitant conditions):** Patients taking anti-TB treatment at the time of enrolment were excluded from the study; 91.6 % received co-trimoxazole preventive therapy (CPT)and 20.8% isoniazid preventive therapy (IPT); 47.7% antiretroviral therapy (ART) | |
| Outcomes | **Tuberculosis (active tuberculosis or tuberculosis disease)**  An event incidence of TB in this study was considered as any form of TB that was diagnosed clinically or radiographically and confirmed by laboratory examinations or by patients who have empirically started anti-TB treatment after enrolment.  Was the same outcome definition (and method for measurement) used in all patients? Yes  Type of outcome: incident TB.  Time of outcome occurrence or summary of duration of follow-up: All participants were followed up from the date of the national health screening in 2010 to the date of TB diagnosis or December 31, 2017, whichever came first.  **TB recurrence**  Not reported | |
| Risk factor: undernutrition | Definition and method for measurement: underweight (BMI l <18.5 kg/m²) compared to no underweight (BMI ≥18.5 kg/m²). It did not describe how BMI (weight and height) was assessed.  Timing of measurement: at enrollment.  Handling of predictors in the modelling: undernutrition (<18.5 kg/m²) vs. no undernutrition (BMI ≥18.5 kg/m²) | |
| Sample size, missing data and analysis | **Number of participants, number of outcomes/events and amount of accumulated person-years (PY):**  Overall: 503 total participants, 451 participants (after exclusion); 119 cases; 1,377.41 PY  **Number of participants, person-years and outcomes/events in relation to the number of candidate predictors (Events Per Variable)**   - BMI <18.5 kg/m²: 218 participants; 75 cases of incident TB; 626.46 PY; 750.95 PY - BMI ≥18.5 kg/m²: 203 participants; 42 cases of incident TB   **Number of participants with any missing value:** 52 records were excluded because of incomplete information.  **Number of participants with missing data for each predictor:** not specified; maybe none; from the text: “incomplete information, such as missing the date of enrolment, outcome of interest and follow-up data.  **Handling of missing data (e.g., complete-case analysis, imputation, or other methods):** complete-case analysis.  **Modelling method:**   - multivariate Cox regression models - Variables with P values of less than 0.2 in the bivariable analysis were considered for the multivariate Cox proportional hazard model - statistical significance was defined as 2-sided *p<0.05.*   **Assumptions**: basic assumptions of the Cox proportional hazard model were checked using the Schoenfeld residuals test.  **Adjustment factors used:** Adjusted for marital status, family size, substance use, history of TB, baseline CD4 count, WHO clinical stage, opportunistic infection, Hgb level, isoniazid preventive therapy (IPT) | |
| Results | **Interpretation**  People living with HIV with a baseline BMI less than 18.5 kg/m² were 2.53 times at higher risk of developing TB at any time than those with a BMI greater than 18.5 kg/m². Adjusted HR for incidence of TB for underweight compared to not underweight: BMI < 18.5 (underweight) 2.53 (95% CI 1.27; 5.05).  **Comparison with other studies**  In this study, the overall incidence of TB among PLHIV was 8.64 cases per 100 PY of observation. This finding is similar to those reported from Gondar and Asella, Ethiopia, which are 7 and 7.9 cases per 100 PY of observation (References in the report number 31 and 32).  Out of the anthropometric variables, patients with HIV who were underweight (BMI <18.5 kg/m2) were 2.53 times at higher risk of developing TB compared with individuals with BMI ≥18.5 kg/m2. This finding was consistent with a study in Tanzania, Ethiopia, and South Africa (References in report numbers 33, 43, and 44).  **Discussion of generalizability**  Only persons with HIV and the study conducted in a single region in Ethiopia; not representative of people without HIV and from other regions.  **Strengths and limitations**  Limitations:   - retrospective design: not all important risk factors could be included - Inability to conduct culture confirmation (the gold standard method) - The inability to address TB contacts (other family members/ coinhabitant) and the introduction of selection bias due to the exclusion of patients who did not use the selected hospitals are other drawbacks. - study conducted only in persons with HIV and in a specific region with an overall low incidence rate of TB, - undernutrition compared to no undernutrition; only binary variable; no information on how weight and height (or BMII) were assessed   **Strengths:**   - Five years follow-up; longer than similar studies. | |
| Funding | No funding. | |
| Conflict of interests | The authors declare no competing interests. | |
| Notes | We contacted 23.01.24 the author, Melaku K Yenit (melaku98@gmail.com), for details on outcome assessment and missing data. | |
| ***Risk of bias*** | | |
| **Bias** | **Authors' judgement** | **Support for judgement** |
| Study participation | Unclear risk | Participants with HIV/AIDS in selected health centres in the region. |
| Study Attrition | Unclear risk | 10.3% attrition and excluded from the analyses. |
| Prognostic Factor Measurement | Unclear risk | No details on the measurement (or self-report) of BMI. More than 10% of the population was excluded due to missing baseline data, including BMI. |
| Outcome Measurement | Unclear risk | Same for all participants but unclear how many were laboratory-confirmed. None were culture-confirmed. |
| Study Confounding | Unclear risk | All important confounders with the exception of DM. |
| Statistical Analysis and Reporting | Unclear risk | Regression model and confounding variables explained; robustness of results not tested with sensitivity analyses. |

Aibana 2016

| ***Study characteristics*** | | |
| Study details and sources | **Study design:** Prospective cohort study  **Source of data:** Program for Household Contacts  **Study dates:**September 2009 and August 2012  **Setting:** Health centres (106 centres in Lima, Peru)  **Countries:** Peru | |
| Participants | **Participant eligibility**  Any household contacts of index TB cases.  **Recruitment method**  Between September 2009 and August 2012, patients older than 15 years of age diagnosed with pulmonary TB (PTB) were identified by the National TB Program (NTP) in 106 participating health centres. These “index” TB patients were ascertained whether they had microbiologic confirmation of PTB disease with sputum smear and mycobacterial culture. Within two weeks of enrolling in an index TB case, the patient’s household was visited to enroll all household contacts and screen for TB infection and disease. Household contacts referred for evaluation were assessed by clinicians for pulmonary and extra-pulmonary TB disease as indicated.  **Participant Description**   - Adults (20+, 57%) and children and adolescents (43%) - Sex: 44.8% male   **Risk factors**   - diabetes: self reported 0% - undernutrition: 226/12,648 (1.8%) - HIV infection: 0.4% - recent TB infection: 7.8% history of TB - history of untreated or inadequately treated TB disease: not described - immunosuppressive therapy: not described - cigarette smokers: 6.1% - drug or alcohol use disorders: heavy alcohol use 5.4$ - socioeconomic status: low 34.4%, middle 44.8%, high 20.8% - other (specify)   - Comorbid disease 20.2%   - TB at baseline 43.4% (excluded from analysis)   - Isoniazide prevention 22.8%   - Index Case Characteristics     - HIV Positive 3.4%     - Tobacco use 2.8%     - Heavy Alcohol Use 10.1%   **Details of treatments received (treatment for diabetes, undernutrition and other concomitant conditions):**  Isoniazid Preventive Therapy. | |
| Outcomes | **Tuberculosis (active tuberculosis or tuberculosis disease)**  "We considered contacts to have acquired TB infection during the study follow-up period if they were TB uninfected at baseline and later converted their TST or developed secondary TB disease. We considered contacts to have developed incident secondary TB disease if they had microbiological confirmation by sputum smear or culture if they had extra-pulmonary TB, or if they were clinically diagnosed by a physician and initiated on TB treatment at least 15 days after index case enrollment."  "We defined secondary TB disease among contacts younger than 18 years of age according to the consensus guidelines for classifying TB disease in children" [Ref: 26].  Was the same outcome definition (and measurement method) used in all patients? Yes  Type of outcome: incident TB and secondary TB disease  Time of outcome occurrence or summary of duration of follow-up: 1 year.  **Recurrent TB**  Not measured | |
| Risk factor: undernutrition | **For adults:** underweight (BMI l <18.5 kg/m²), normal (BMI 18.5-25 kg/m² and overweight (BMI ≥25 kg/m²).  **For children and adolescents <20 years:** WHO age and sex-specific BMI z-score tables to classify those with BMI z-score < –2 as underweight and those with z-score >2 as overweight.  How BMI (weight and height) was assessed was not described.  **Timing of measurement:** at baseline | |
| Sample size, missing data and analysis | **Handling of predictors in the modelling:** underweight, normal weight and overweight, as defined above.  **Number of participants, number of outcomes/events and amount of accumulated person-years (PY):** 12,430 participants, TB incidence: 6,853 participants in TB infection negative at baseline; 119 cases; 3,549 PY  **Number of participants, person-years and outcomes/events in relation to the number of candidate predictors (Events Per Variable)**   - BMI at baseline: - BMI <18.5 kg/m²: 226 participants; cases and PY not reported - BMI 18.5 kg/m²-<25 kg/m²: 7,310 participants; cases and PY not reported - BMI ≥25 kg/m²: 5,112 participants; cases and PY not reported   **Number of participants with any missing value excluded:** 1,277 (9.1%) contacts without a known microbiologically confirmed index case and 119 (0.8%) contacts whose baseline height or weight was missing; 1563 with unknown follow-up information.  **Number of participants with missing data for each predictor:** 119 (0.8%) contacts whose baseline height or weight was.  **Handling of missing data (e.g., complete-case analysis, imputation, or other methods):** complete-case analysis  **Modelling method:**   - Multivariate Cox regression models - variables identified a priori as potential confounders (age, sex, heavy alcohol consumption, tobacco use, SES, HIV, DM and comorbid disease) and any others associated with the outcome with a p value < 0.2 in the univariate analysis - statistical significance was defined as 2-sided *p<0.05.*   **Assumptions:**  Proportional hazard assumptions verified for each covariate by introducing an interaction term between the covariate and time, stratified by variables for which the proportional hazards assumption did not hold.  **Adjustment factors used:**  Adjusted for age, sex, heavy alcohol consumption, tobacco use, SES, HIV, diabetes mellitus, TB history, comorbid disease and index-case characteristics. | |
| Results | **Interpretation**  Adjusted HR for incidence of TB for underweight compared to normal weight: BMI <18.5 (underweight) = 0.56 (0.31; 1.02); inversely associated but imprecisely estimated.  Adjusted HR for secondary TB for underweight compared to normal weight: BMI <18.5 (underweight) = 1.42 (0.74; 2.74); increased risk but imprecisely estimated.  **Comparison with other studies:**  "Our findings are quantitatively consistent with recent studies across various settings that have demonstrated an association between elevated BMI and decreased risk of TB disease."  **Discussion of generalizability:** only persons with household contacts.  **Strengths and limitations**  **Limitations:**   - For DM: self-reports; many people undiagnosed - BMI: TB might lead to weight loss; contacts with subclinical TB that were undetected during baseline evaluation may have had lower BMI at enrollment; this may have led to overestimation of the observed effect of BMI on TB disease. To address this, a sensitivity analysis was conducted evaluating the impact of BMI on incident TB disease diagnosed at least 90 days after index case enrollment and higher BMI was associated with an even lower risk of TB disease diagnosed later during follow-up. - BMI is not an accurate marker for fatty tissue content, especially in children.   **Strengths:**   - prospective design - large sample size | |
| Funding | "National Institute of Allergy and Infectious Diseases [U19 AI076217 to MBM; U01 AI057786 to MCB; T32 AI007433 and L30 AI120170 to GEV] and the National Institute on Drug Abuse [T32 DA013911 to OA and SC]. The funders had no role in study design, data collection and analysis, decision to publish, or preparation of the manuscript." | |
| Conflict of interests | The authors declare no competing interests. | |
| Notes | Email 12.12.23 to megan.murray.epi@gmail.com to ask about proportion of culture-confirmed diagnosis. On the 15.12 the author replied that 43% of cases were culture positive. | |
| ***Risk of bias*** | | |
| **Bias** | **Authors' judgement** | **Support for judgement** |
| Study participation | Unclear risk | Regional participants and regional TB database as index cases; household contacts. |
| Study Attrition | Unclear risk | Moderate attrition (9.9%) and poorly described. |
| Prognostic Factor Measurement | Low risk | Standard method of measurement of undernutrition. |
| Outcome Measurement | Unclear risk | Microbiological confirmation only in 43% of cases (information provided by the author). |
| Study Confounding | Low risk | The study adjusted for all the pre-defined variables. |
| Statistical Analysis and Reporting | Low risk | Adequately presented Cox regression analysis for the defined model. |

Alemu 2020

| ***Study characteristics*** | | |
| Study details and sources | **Study design:** retrospective cohort study  **Source of data:** ART clinics in Addis Ababa (Ethiopia)  **Study dates:** 1 January 2013 - 31 December 2018  **Setting:** outpatients  **Country:** Ethiopia | |
| Participants | **Participant eligibility:**   - Inclusion:   - HIV-positive people   - newly registered at ART centres of 7 public health facilities in Addis Ababa in 2013   - Free of TB infection at enrollment (baseline) - Exclusion:   - Incomplete baseline information or missing charts   Recruitment method: 18 from 123 ART clinics were selected for their high patient flow. Then, 7 of these were randomly selected using a simple random sampling technique. Data were collected retrospectively from patients' charts, which were allocated proportionally to each ART clinic using a systematic random sampling method.  **Participant Description:**   - Age: The mean age at baseline was 35.4 years (SD 8.9 years) - Sex: 32% men   **Risk factors:**   - diabetes: no - undernutrition: 32.2% had BMI <18.5 - HIV infection: all (incl criterium) - recent TB infection: 9.4% had a history of TB - history of untreated or inadequately treated TB disease immunosuppressive therapy: NR - cigarette smokers: 49.1% smoked tobacco - drug or alcohol use disorders: 55.1% had alcohol addiction; 47.7% took hard drugs/shisha; 52.3% used khat - socioeconomic status: 17.7% no formal education; 36.4 primary schools, 34.5 secondary schools, and 11.5 tertiary schools, marital status was reported (single, married, divorced and widowed); 41.7% employed - other (specify)   - Family size: 59.0% 1-3; 29.2% 4-5; 11.8% ≥5   - WHO stage: 91.2% stage I/II; 9.4% stage iii/IV   Details of treatments received (for tuberculosis): NR | |
| Outcomes | **Tuberculosis (active tuberculosis or tuberculosis disease)**  Definition and method for measurement of outcome: At least one specimen was confirmed for *Mycobacterium tuberculosis*, or there was histological or strong clinical evidence consistent with TB, with the diagnosis ultimately made by a clinician. Identified by bacteriological diagnostics (smear microscopy, Xpert MTB/RIF assay, or culture), histological methods, or clinically by clinical decision.  Was the same outcome definition (and method for measurement) used in all patients? Yes  Type of outcome: incident active TB  Time of outcome occurrence or summary of duration of follow-up: cumulative incidence.  **Recurrence of TB**  Not measured | |
| Risk factor: undernutrition | - Based on BMI: <18.5 versus >18.5 - No further definition is provided | |
| Sample size, missing data and analysis | **Number of participants and number of outcomes/events:**   - Number of participants: 566 (yet 612 enrolled) - Outcomes: 146   - Pulmonary TB: 80 (14.1%)   - Extrapulmonary TB: 72 (12.7%)   - Both: 6 (1.1%)   **Number of outcomes/events in relation to the number of candidate predictors (Events Per Variable):**   - 14 confounders for 72 events (= 5 events per confounder)   **Number of participants with any missing value (including predictors and outcomes)** 46 had been excluded due to incomplete follow-up data (for analysis)  **Number of participants with missing data for each predictor.** Not reported, but probably none.  **Handling of missing data (e.g., complete-case analysis, imputation, or other methods):** Not reported.  **Modelling method:** Descriptive analyses, incidence density per 100 PY, Kaplan Meyer survival curves (staying TB free); also stratified for variables. Univariate Cox PH regression: vars with p-value ≤0.25 were fitted in the multivariate model. Multivariate Cox regression.  **Assumptions:** proportional hazards checked.  **Adjustment factors:** Sex, employment status, marital status, family size (1-3, 4-5, >5), alcohol addiction, previous TB history, functional status (working, ambulatory, bedridden), WHO clinical staging (I or II, III or IV), CD4 count (<50, 50-200, >200 cells/ul), haemoglobin level (<10, ≥10 g/dl), BMI (<18.5, >18.5 kg/m²), on HAART, cotrimoxazole and isoniazid therapy. | |
| Results | **Interpretation:**  People living with HIV who had a baseline BMI <18.5 kg/m² were at 1.907 times the risk of developing TB at any time when compared to people with HIV who had a baseline BMI >18.5 kg/m²  **Comparison with other studies:**  The underweight finding was consistent with those of studies done in North-East Ethiopia (ref Ahmed 2018), South-West Ethiopia (Reference in the report: Taha 2011) and Western Ethiopia (Reference in the report: Tiruneh 2019)  Discussion of generalizability: none  **Strengths and limitations**  *Strengths*: not reported.  *Limitations*:   - Possible selection bias: the sociodemographic, behavioural and clinical characteristics of the 46 excluded individuals (due to missing FU data) were different from the included individuals (no further info provided) - Unrecorded information in the patient charts was not sought. | |
| Funding | The authors report no funding. | |
| Conflict of interests | The authors reported no conflicts of interests. | |
| Notes | We asked Dr Alemu (ayinalemal@gmail.com) on 23.01.24 details about culture confirmation. | |
| ***Risk of bias*** | | |
| **Bias** | **Authors' judgement** | **Support for judgement** |
| Study participation | Unclear risk | Only about HIV patients registered for ART in the region. |
| Study Attrition | Unclear risk | 7.5% attrition and excluded from the analyses |
| Prognostic Factor Measurement | Low risk | Based on clinical standard criteria, but lacking detailed descriptions. |
| Outcome Measurement | Unclear risk | Valid assessment of TB applicalbe to all participants (including culture, but proportion unclear). |
| Study Confounding | Unclear risk | Adjusted for all relevant factors except diabetes mellitus. |
| Statistical Analysis and Reporting | Low risk | Adequately presented Cox regression analysis for the defined model. |

Anaam 2020

| ***Study characteristics*** | | |
| Study details and sources | **Study design:** Prospective cohort study  **Source of data:** Clinical Health Records  **Study dates:** July 2007 - March 2014  **Setting:** Outpatient  **Countries:** Yemen | |
| Participants | **Participant eligibility:**   - Inclusion criteria:   - age 15 years and older   - smear-positive pulmonary TB   - treatment with an 8-month daily regimen (2HRZE/4HE)   - available two months smear conversion results - Exclusion criteria:   - younger than 15 years   - Smear-negative pulmonary TB   - extrapulmonary TB   - being on other TB treatment regimens than 2HRZE/4HE   Recruitment method: prospective enrollment of TB patients at ten health centres with TB units in 10 governorates throughout Yemen.  **Participant description**   - Age: mean age (SD): 32 (13); median age (IQR): 27 (22-40) - Sex: 57.6% men; 42.4% women   **Risk factors**   - diabetes: 66 (8.1%) - undernutrition: 199 (24.4%) had BMI ≤18.5 kg/m² - HIV infection: 0 had HIV infection - recent TB infection: 100% had baseline pulmonary TB - history of untreated or inadequately treated TB disease immunosuppressive therapy: 617 participants were cured; 22 had completed treatment (text page 4: § recurrence rate) - cigarette smokers: 122 (15.0%) - drug or alcohol use disorders:377 (46.3%) khat leave chewers - socioeconomic status: (from n=751): 262 (35%) illiterate; 396 (53%) unemployed - other (specify):   - Cavitary lung disease: 224 (30.0%)   - positive acid-fast bacilli smear at end of 2 months: 42 (5.2%)   - weight gain during intensive trt phase ≤5%: 415 (51.0%)   - weight loss >10%: 269 (33.1%)   - BMI (SD): 19.0 (2.9)   - Non-adherence to treatment:123 (16.4%) (from 715)   Details of treatments received (treatment for diabetes, undernutrition and other concomitant conditions): the cohort had received a complete course of treatment (see above in inclusion criteria) | |
| Outcomes | **Tuberculosis (active tuberculosis or tuberculosis disease)**  NR  **Recurrence of tuberculosis after tuberculosis infection**  Definition: a patient who successfully completed treatment and was declared cured, but was diagnosed again with bacteriologically positive TB.  Method for measurement: when patients returned to the clinic with TB-related symptoms, a chest X-ray was made, and sputum acid-fast bacilli smear tests were performed in case of suspicion of TB on the X-ray - thus smear and/or culture  Was the same outcome definition (and method for measurement) used in all patients? NO, page 4 reports that TB recurrence was identified in patients 49 of 617 considered cured (conform definition) and in 22 of 134 who had completed treatment.  Type of outcome: single.  Time of outcome occurrence or summary of duration of follow-up: 5 years of follow-up since baseline; no further details | |
| Risk factor: undernutrition | Definition: BMI ≤18.5 kg/m²  Underweight were patients with less than 90% of ideal bodyweight at time of diagnosis  Method for measurement: unclear if self-reported extracted from clinic records, or measured by investigators  Timing of measurement: at baseline  Handling of predictors in the modeling: dichotomous (≤18.5 kg/m²: yes versus no) | |
| Sample size, missing data and analysis | **Number of participants, number of outcomes/events and amount of accumulated person-years (PY):**   - Number of participants: 815 (751 with available FU data; incl in the analyses) - Outcomes: 71 (9.5%) in 751 participants   - 49 (7.8%) in the 617 considered cured   - 22 (16%) in the 134 with completed treatment   **Number of participants, person-years and outcomes/events in relation to the number of candidate predictors (Events Per Variable):**   - 10 confounders for 71 events (= 7 events per confounder)   **Number of participants with any missing value (include predictors and outcomes):** 63 had died or were lost-to-FU and had no 5 years of FU. They were excluded from the analyses, no further details.  **Number of participants with missing data for each predictor:** NR  **Handling of missing data (e.g., complete-case analysis, imputation, or other methods):** excluded  **Modelling method:**   - descriptive analyses - logistic regression analysis: univariate statistically significant risk predictors were included in the multivariate regression analysis. No details on selection of the variables for the univariate analysis.   **Assumptions:** None tested  **Adjustment factors used:**   - Illiterate (yes/no) - unemployed (yes/no) - smoking (yes/no) - chewing khat (yes/no) - cavitary disease (yes/no) - weight gain ≤5% (yes/no) - underweight (yes/no) - BMI ≤18.5 kg/m² (yes/no) - Diabetes (yes/no) - non-adherence (yes/no) | |
| Results | **Interpretation:**  “Univariate analysis showed that …., weight gain ≤5% during the intensive phase, being underweight, BMI ≤18.5 kg/m², diabetes, and …. were associated with an increased risk for recurrence.” “There was a significant association between diabetes and recurrence of TB.” “In this study, body weight at the time of diagnosis, including underweight was not a predictor of recurrence in the multivariate model.”  **Comparison with other studies:**  “Consistent with previous studies [refs. 29, 35, 44, 50-52], there was a significant association between diabetes and recurrence of TB”.  For BMI, no previous literature on the association with recurrent TB was provided.  Ref 57 for the statement on food insecurity and limited access to nutritious foods in part of Yemen.  **Discussion of generalizability:**  “Unlike some previous cohort studies that included only high-risk populations, such as HIV co-infected or homeless patients, this study was conducted in a general TB population. The study has a relatively large sample size from 10 governorates throughout the country. This could enhance its generalizability” (p6)  **Strengths and limitations:**  Strengths:   - Prospective with active FU for five years - good generalizability due to the large random sample size from the general TB population.   Limitations:   - 7.7% of participants were excluded due to death or lost-to-FU - The potential role HIV not investigated (but the proportion of co-infected patients is likely low) - Only focussed on TB patients treated with the 2RHZE/6HE regimen, not other regimens | |
| Funding | Not available. | |
| Conflict of interests | No potential conflict of interest was reported by the authors. | |
| Notes | Email 26.09.23 to contact author (alianaaly@gmail.com) requesting data on how data was collected at baseline and follow-up, the participation rate, information about other confounders, data on missing data and proportion of culture-confirmed-TB. | |
| ***Risk of bias*** | | |
| **Bias** | **Authors' judgement** | **Support for judgement** |
| Study participation | Unclear risk | Partially described subpopulation of TB patients with completed treatment. |
| Study Attrition | Unclear risk | Moderate attrition (7%) and poorly described. |
| Prognostic Factor Measurement | Low risk | Standard definitions for PF, although missing data was not described |
| Outcome Measurement | Unclear risk | Standard definitions for TB recurrence, although missing data was not described, nor proportion with culture/Xpert. |
| Study Confounding | Unclear risk | Immunosupression and alcohol were not accoutned in the analysis but other locally relevant variables were. |
| Statistical Analysis and Reporting | Low risk | No conceptual framework for the selection of confounders, otherwise well described. |

Ayana 2021

| ***Study characteristics*** | | |
| Study details and sources | **Study design:** Retrospective cohort  **Source of data:** Clinical Health Record  **Study dates:** February 2016 - December 2019  **Setting:** Outpatient - Single Hospital  **Countries:** Ethiopia | |
| Participants | **Participant eligibility:** adults living with HIV/AIDS free of TB infection.  **Recruitment method:** The source population was ALL adults with HIV/AIDS in the study period in the hospital. The required 471 participants (sample size calculation) were randomly selected using computer-generated random numbers.  **Participant description:**   - Age: median 38 years (IQR 30-46) - Sex: 43.13% Male   **Risk factors:**   - diabetes: not described - undernutrition: 18% undernutrition - HIV infection: all participants - recent TB infection: history of TB infection 19% - history of untreated or inadequately treated TB disease: not described - immunosuppressive therapy: not described - cigarette smokers: 13% - drug or alcohol use disorders: alcohol use 33% - socioeconomic status: 30% no formal education or primary school; 1% were unemployed or daily labour - other: Baseline CD4 <200 20%.   Details of treatments received (treatment for diabetes, undernutrition and other concomitant conditions): All received ART for HIV infection. | |
| Outcomes | **Tuberculosis (active tuberculosis or tuberculosis disease)**  Definition and method for measurement of outcome:  Definition: “incident TB (a new active TB) during follow-up”  Measurement: not specified  Was the same outcome definition (and method for measurement) used in all patients? Possibly yes.  Type of outcome: single  Time of outcome occurrence or summary of duration of follow-up: minimum 12 to maximum 48 months follow-up  **Recurrent TB**  Not measured | |
| Risk factor: undernutrition | *Definition and method for measurement*: normal weight is BMI 18.5–24.9 kg/m2, BMI less than 18.5, and greater than 25 is underweight and overweight, respectively. It is not defined how it is measured.  Timing of measurement: at baseline. | |
| Sample size, missing data and analysis | **Handling of predictors in the modelling (e.g., continuous, linear, non-linear transformations or categorised):** categorised  **Number of participants, number of outcomes/events and amount of accumulated person-years (PY):** 471 patients were followed for a median of 44 months (IQR 33, 46).  Participants were censored at the end of the study period, died before developing TB and being lost to follow-up.  **Number of participants, person-years and outcomes/events in relation to the number of candidate predictors (Events Per Variable)** Source population=482: 11 participants with missing data. In analysis 471 (97.7%). 56 cases of TB during follow-up. 10 Predictors in the model for 56 cases = 1 confounder per 6 cases. Normal weight: 24 cases / 368 censored participants. Underweight: 38 cases/48 censored participants.  **Number of participants with any missing value (including predictors and outcomes):** 11 (2.3%) participants were excluded because their date of TB co-infection was not well-recorded.  **Number of participants with missing data for each predictor:** not available.  **Handling of missing data (e.g., complete-case analysis, imputation, or other methods):** Complete case analysis (due to a better fit).  **Modelling method:**   - Generalised linear mixed effect model and the Cox proportional hazard model.   **Assumptions:**   - The significance level of 0.05 was used as a cut-off point for all statistical tests. - Cox Snell residual was used to check the overall goodness of fit for the survival sub-model, and the marginal residual was used for the longitudinal sub-model. - Akaike Information Criteria (AIC) was used for model selection.   **Adjustment factors used:**   - Socio-demographic characteristics: age, sex, marital status, educational status, and occupation - baseline clinical and behavioural characteristics: Baseline CD4 count, history of TB, baseline functional status, past opportunistic infection, baseline haemoglobin, baseline BMI, initial ART regimen, and adherence at baseline - Time-varying endogenous covariates: Repeated measured viral load recorded as the number of viral copies per millilitres. | |
| Results | **Interpretation:** “Baseline age, baseline BMI, and past opportunistic infection were significant predictors of the incidence of TB.”  **Comparison with other studies:** “Underweight patients had a higher hazard of experiencing active TB compared to patients with normal BMI. This finding is consistent with a study done in northeast Ethiopia.”(Reference in report number 14)  **Discussion of generalizability:** Not described  **Strengths and limitations:** Secondary analysis of medical records with missing data on socioeconomic status. | |
| Funding | Not available. | |
| Conflict of interests | The authors report no conflicts of interest for this work. | |
| Notes | 27 August 2023 - Email: temesgenyihunie@gmail.com and Temesgen.akalu@telethonkids.org.au   1. What are the number of cases and individuals who were censored for “underweight” and the other categories of BMI used in the analysis? 2. How was active TB measured? (consequences for RoB assessment) 3. What confounders were adjusted for? Those in table 5 or those mentioned in the text. (consequences for RoB assessment) | |
| ***Risk of bias*** | | |
| **Bias** | **Authors' judgement** | **Support for judgement** |
| Study participation | Unclear risk | Only about HIV patients on ART in one hospital |
| Study Attrition | Low risk | Minimal attrition (2.3%) |
| Prognostic Factor Measurement | Low risk | Based on clinical standard criteria, but lacking detailed descriptions. |
| Outcome Measurement | High risk | Incident TB was diagnosed by clinical criteria but without details on how the diagnosis was made, opportunistic infections were differentiated on HIV patients. |
| Study Confounding | Unclear risk | All except DM, immunosuppressive therapy. |
| Statistical Analysis and Reporting | Low risk | Adequately presented Cox regression analysis for the defined model. |

Baker 2012

| ***Study characteristics*** | | |
| Study details and sources | **Study design:** prospective cohort study (survey linked to registry data)  **Source of data:** interview data, registry data and death certificates.  **Study dates:** August 2001 to December 2004  **Setting:** outpatient and inpatient  **Countries:** Taiwan | |
| Participants | **Participant eligibility**  Participants of the (cross-sectional) National Health Interview Survey who consented to linkage with NHI data, >= 12 years, and who had >= 1 medical visit before December 2004. *Exclusion*: no consent to linkage with NHI registry data.  **Recruitment method**  National Health Interview survey (cross-sectional survey August 2001 - February 2002), linked with the data captured in the Taiwan National Health Insurance (NHI) database (2001- December 2004) and the death registry of the Taiwan Ministry of Health.  **Participant description**   - Age: ≥12 yrs (median age: 37.91 yrs (24.88-51.43) - Sex: Male (50%), Female (50%)   **Risk factors**   - diabetes: treated DM (y/n): 7% yes,all DM (y/n): 9% yes - undernutrition: BMI (kg/m²) (median BMI: 22.5 (20.17-25.10) - HIV infection: NR - recent TB infection: NR - history of untreated or inadequately treated TB disease immunosuppressive therapy: NR - cigarette smokers: 22% current, 3% former, 75% never - drug or alcohol use disorders: 74% never, 13% social, 9% regular, 4% heavy - socioeconomic status: Education   - l48% high school, 42% elementary school, 11% less than elementary school - other (specify)   - Living in a crowded home   - Low income   - Receiving government subsidy   - Employed   - Marriage status: never married; married or cohabitating; widowed, divorced, separated, single parents.   - Hypertension   - Heart disease   - Lung disease   Details of treatments received (treatment for diabetes, undernutrition and other concomitant conditions): NR; in text, page 819, eligible diabetes medication is listed, no numbers are provided. | |
| Outcomes | **Tuberculosis (active tuberculosis or tuberculosis disease)**  Definition and method for measurement of outcome:  Definition: incident TB.  Diagnosis: ALL of the following 3 criteria had to be present in the NHI registry: (1) ≥1 medical visit during follow-up ICD-9-CM code (010-018) for TB; (2) a prescription for ≥2 antituberculosis medications for > 28 days during follow-up; and (3) no finding of TB-misdiagnosis during follow-up on the bass of later diagnosis of nontuberculous mycobacterial infection, lung cancer, or tuberculosis infection without evidence of disease. *Hence, TB based on ICD-9 codes and prescription history rather than microbiological data.*  Was the same outcome definition (and method for measurement) used in all patients? Yes, all 3 criteria  Type of outcome: single endpoints.  Time of outcome occurrence or summary of duration of follow-up: Person-time accrued from the date of the initial interview (NHIS) until the end of follow-up (Dec 2004), death, or development of TB.  **Recurrent TB**  Not measured. | |
| Risk factor: undernutrition | Definition and method for measurement: BMI was categorized as overweight, normal weight and underweight. No mention of categorical cutoffs, though.  Timing of measurement: at baseline.  Handling of predictors in the modeling: categorical (overweight/normal/underweight). | |
| Sample size, missing data and analysis | **Number of participants and number of outcomes/events:**   - Number or participants: 17,715 - Number of outcomes: 57 TB cases /17712, 13/57 also had “treated DM”.   **Number of outcomes/events in relation to the number of candidate predictors (Events Per Variable):** 15 confounders for 57 events = 4 cases per confounder.  **Number of participants with any missing value (include predictors and outcomes):** missing data is described below.  **Number of participants with missing data for each predictor:** <0,1% missing data for sex, age, crowding, smoking, employment, residence in indigenous community, and marital status; 0,9% of household income; 0,4% for gov. subsidies; 0,1% for education; 0,2% for alcohol use; 10,6% BMI; 1,2% hypertension; 1,5% heart and lung disease.  **Handling of missing data (e.g., complete-case analysis, imputation, or other methods):** Markov Chain Monte Carlo methods to impute missing values of the covariates. Sensitivity analysis for potential bias by loss to follow-up.  **Modelling method**   - Cox proportional hazards regression analysis for univariate and multivariate HRs for TB among those with DM. - Markov Chain Monte Carlo methods were used to impute missing values of the covariates. - Sensitivity analysis: Person-time until date of last medical visit instead of the end of study (Dec 2004) - Severity analysis: The number of complications and the DCSI score as indicator variables were assessed in the multivariate Cox regression analysis for “treated DM” and the risk of TB. - All tests are 2-sided statistical tests with alpha = .05 and CI of 95%.   **Assumptions:** NR.  **Adjustment factors used:** DM/BMI, age, sex, crowding, low household income, government subsidy, employment status, residence in indigenous community, marital status, education, smoking, alcohol use, lung disease, heart disease and hypertension. | |
| Results | **Interpretation:**   - Those with DM were older, had a higher BMI, and were more likely to have a low household income, receive government subsidies, be unemployed, be married or cohabitating, receive less schooling, and have hypertension, heart disease, and lung disease, compared with those without DM.   **Comparison with other studies:**   - [References number 29, 30] Although high BMI is a strong risk factor for DM, it has been shown in numerous studies to be an independent protective factor against tuberculosis.   **Discussion of generalizability:** Not reported.  **Strengths and limitations**   - *Strengths*: None reported. - *Limitations*: 1) DM was classified based on self-report, ICD-9-CM codes, and pharmacy records; therefore, undiagnosed cases could not be included. 2) TB was defined based on ICD-9-CM codes and prescription history rather than microbiological data. 3) There was no data on HIV due to the low prevalence (< 0.02% in 2003). 4) Contact with people with TB was not included as a risk factor (unmeasured risk factors). | |
| Funding | Not reported. | |
| Conflict of interests | No reported conflicts. | |
| Notes | Emails were sent on 19.09.2023 to mmurray@hsph.harvard.edu (paper) and megan\_murray@hms.harvard.edu (internet) about cut-off points for BMI, the number of cases for each category of BMI and diabetes, and rates of self-reporting of the prognostic factor. | |
| ***Risk of bias*** | | |
| **Bias** | **Authors' judgement** | **Support for judgement** |
| Study participation | Low risk | Wide participation from a large national database. |
| Study Attrition | Low risk | All participants were included in the analysis (Imputation was used for missing data) and sensitivity analysis was assessed for potential bias by loss to follow-up. |
| Prognostic Factor Measurement | Unclear risk | BMI is self-reported. The classification of BMI in categories underweight, normal weight and overweight was described in words only; no BMI-cutpoints were specified. |
| Outcome Measurement | Unclear risk | Same for all participants but unclear how many were culture-confimed. |
| Study Confounding | Unclear risk | All except HIV, previous and a history of TB, immunosuppression. |
| Statistical Analysis and Reporting | Unclear risk | Regression method appropriate, yet analyses were not a-priori designed to estimate BMI as a risk factor for tuberculosis (focus was on diabetes). Also, the sensitiviry analyses were only informative for diabetes as a risk factor. |

Batista 2013

| ***Study characteristics*** | | |
| Study details and sources | **Study design:** prospective cohort study  **Source of data:** participant interviews  **Study dates:** July 2007 - December 2010  **Setting**: outpatients  **Country**: Brazil | |
| Participants | **Participant eligibility:**   - Inclusion:   - aged ≥18   - living with HIV.   - available questionnaire data. - Exclusion:   - being treated for TB at study entry   - developing TB in 1st month of follow-up   **Recruitment method:** All individuals with HIV who received medical care in 2 reference hospitals (responsible for care of 70% of HIV patients), aged ≥18 years, who consented to participate and who responded to the questionnaire (for data collection) were recruited. No further details were provided.  **Participant description:**   - Age: 18-39 years: 51.3%; ≥40 years: 48.7% - Sex: 61% men   **Risk factors:**   - diabetes: no - undernutrition: 7.5% had BMI <18.5 - HIV infection: all (including criterium) - recent TB infection: 21.7% had previous TB treatment - history of untreated or inadequately treated TB disease immunosuppressive therapy: NR - smoking:45.1% never; 25.9% ex-smoker; 29.0% current smoker - drug or alcohol use disorders: 88.6% none or light drinker; 11.4% heavy drinker72.6% neve user; 27.4% past or current user of recreational drugs. - socioeconomic status: head of the family monthly income: 68.7% ≥1 min wage; 31.3% <1 min wage - other (specify)   - Social support: 80.8% living with family; 19.2% living alone or in shelters   - Literacy: 88.8% yes; 11.2% no   Details of treatments received (for tuberculosis): NR | |
| Outcomes | **Tuberculosis (active tuberculosis or tuberculosis disease)**  Definition and method for measurement of outcome:   - Incident active TB: (i) a new TB diagnosis made by the attending physician; (ii) cases of TB notified to the surveillance system during the follow-up period (iii) deaths from TB in Mortality Information System (comment: dying from TB was thus also recorded as an incident TB case; thus no competing risk issues are in order here) - Identified by: (i) based on clinical findings, direct investigation of Acid-fast bacillus-AFB smear, and culture for *M.tb*.(ii) and (iii) ascertained through record linkage with the Surveillance System or Mortality Info System.   Was the same outcome definition (and measurement method) used in all patients? No, see “sample size” for numbers per definition.  Type of outcome: Single outcome (incident active TB)  Time of outcome occurrence or summary of duration of follow-up:   - Person-time at risk: time from the start of FU to the date of TB diagnosis, defined as the date if initiating (a confirmed) TB treatment, or the date of (confirmed) death from TB - Censoring: FU ended at first date of death from other causes, date of transfer to another health service not in the study, or the end of the study on 31 Dec 2010   **Recurrent TB**  Not measured. | |
| Risk factor: undernutrition | Based on BMI: <18.5 versus ≥18.5. No further details of measurements/definitions are provided.  Timing of measurement: presumably at baseline. | |
| Sample size, missing data and analysis | **Handling of predictors in the modelling (e.g., continuous, linear, non-linear transformations or categorised)** categorised.  **Number of participants and number of outcomes/events:**   - Number or participants: 2,069 (yet 2,362 enrolled) - Outcomes: 147 (7.1%)   - Def (i): 3   - Def (ii):144   - Def (iii): 3 (= the 3 from def (i)   **Number of outcomes/events in relation to the number of candidate predictors (Events Per Variable):** 6 confounders for 147 events (= 24 events per confounder)  **Number of participants with any missing value (include predictors and outcomes).** No details were provided, but the numbers in Tables 1 and 2 do not add up to 2,069.  **Number of participants with missing data for each predictor**: 49, based on Table 2  **Handling of missing data (e.g., complete-case analysis, imputation, or other methods):** NR  **Modelling method**   - descriptive analyses - incidence density per 100 PY - KM survival curves (staying TB free); - Univariate Cox PH regression: vars with p-value ≤0.2 were included according to the forward elimination procedure as confounders in the multivariate model - Multivariate Cox PH regression where HAART was modelled as a time-dependent variable.   **Assumptions**: proportional hazards checked.  **Adjustment factors:**   1. Indication for LTBI treatment (none, ind and trt, ind and no trt, tst test not performed) 2. CD4 count (≥200,<200 cells/mm³) 3. BMI (<18.5, ≥18.5 kg/m²) 4. Anemia (yes/no) 5. Previous TB treatment(yes/no) 6. Literacy (yes/no) | |
| Results | **Interpretation**  The risk of TB was higher in people living with HIV with a low BMI.  **Comparison with other studies:**  “TB and HIV infection are known to be associated with malnutrition (Ref 33), …”  “...body mass index <18.5 has been reported to be associated with TB in people living with HIV (ref 35), and this was consistent with our findings.”  **Discussion of generalizability**  Not reported.  **Strengths and limitations**  *Strengths*:   - confident to have included all diagnosed cases of TB - minimization of misclassification in relation to loss of FU and death, searches conducted in the Mortality Info System - Prospective, thus incident cases - HAART was modelled as a time-dependent variable, taking into account the time each patient was at risk.   *Limitations*:   - Possible selection bias, as people who were diagnosed with TB within one month of FU were excluded (N unclear) - heterogeneous sample due to entry in the study was not triggered by common clinical criteria. - Most tests and interventions reflected the decision of the responsible clinician. | |
| Funding | “This study received support from the Ministerio de Sauda/Programa DST/AIDS/UNESCO. The authors received partial support from the Conselho Nacional de Desenvolvimento Cientifico e Technologico.” | |
| Conflict of interests | The authors reported no conflicts of interests. | |
| Notes | 14 February 2024 Email to joannalyra@gmail.com on all details related to risk of bias assessment. 06 April 2024 the author responded clarifying details on missing data, culture (not available) | |
| ***Risk of bias*** | | |
| **Bias** | **Authors' judgement** | **Support for judgement** |
| Study participation | Unclear risk | Only about HIV patients on HAART in two regional hospitals, though 70% of HIV care in the state was initiated in those hospitals. |
| Study Attrition | Unclear risk | 22.9% had missing data on anaemia, but lacking detials. They were included in the analysis. |
| Prognostic Factor Measurement | Low risk | Weight and height measurements were taken during nursing screening using the same scale and height meter, before medical appointments (Author information) |
| Outcome Measurement | Unclear risk | Same for all participants but unclear how many were culture-confimed. |
| Study Confounding | Unclear risk | All except DM, immunosuppressive therapy. |
| Statistical Analysis and Reporting | Unclear risk | The analyses were not a-priori designed for BMI was a risk factor. No sensitivity or subgroup analyses were undertaken. |

Benjumea-Bedoya 2019

| ***Study characteristics*** | | |
| Study details and sources | **Study design:** prospective cohort  **Source of data:** Program for Household Contacts  **Study dates:** 24 months (2005-2009)  **Setting**: outpatient  **Countries:** Colombia (Medellín, Cali, Popayán) | |
| Participants | **Participant eligibility**  Children >15 years old who had spent time every week with an index case in the same household for at least one month before the confirmation of the tuberculosis case. An index case was defined as the first tuberculosis case identified in each household, diagnosed with active pulmonary, smear-positive tuberculosis. Recruitment method: once an adult case was notified to the local tuberculosis control program, the patient and children's household were invited to participate in the study, followed by the signature of informed consent. Follow-ups occurred through visits every six months, and telephone calls every three months. The total follow-up was at least 24 months. Local epidemiological surveillance databases were checked for reported active tuberculosis cases.  **Participant description**   - Age: <15 years of age divided in 4 age groups (<1 year old, 1-4 year old, 5-9 and 10-14) - Sex: Female (48,2%), Male (51,8%)   **Risk factors**   - diabetes: not mentioned - undernutrition (Z-scores): 24.2% - HIV infection: not mentioned - recent TB infection: excluded - history of untreated or inadequately treated TB disease immunosuppressive therapy: excluded - cigarette smokers: not mentioned - drug or alcohol use disorders: not mentioned - socioeconomic status (low 78,4%, no information) - other (specify)   - BCG Scar (80,1%)   - History of another relative with TB (36.3%)   - Proximity to the index case (31.6% slept in the same room)   - Persons per room (34.3%, >3)   - Sputum smear load (34.6% +, 29.2% ++, 36.3% +++)   - Exposure time (67.6% ≤ 3 months, 32.4% ≥ 3 months)   **Details of treatments received**  Not mentioned | |
| Outcomes | **Tuberculosis (active tuberculosis or tuberculosis disease)**  Definition and method for measurement of outcome:  Incident active TB was diagnosed following “Stop TB Partnership Childhood TB” guidelines, considering contact history, clinical immunology, microbial and radiographic criteria. Confirmed of *M. tb* by culture in liquid and/or solid media (Lowenstein-Jensen) from sputum (in adults) and gastric aspirate (in children) for all index and incident cases.  Was the same outcome definition (and method for measurement) used in all patients? Yes  Type of outcome: single.  Time of outcome occurrence or summary of duration of follow-up: Follow-up visit every six months, and phone calls every three months. The total follow-up lasted 24 months.  **Recurrent TB**  Not measured | |
| Risk factor: undernutrition | Definition and method for measurement: Z scores for weight and height: between -1 and 1, normal; less than -1, underweight; and score >1, overweight.  Timing of measurement (e.g., at patient presentation, diagnosis, treatment initiation) not mentioned.  Handling of predictors in the modelling (e.g., continuous, linear, non-linear transformations or categorised): categorized between normal, underweight and overweight. | |
| Sample size, missing data and analysis | **Number of participants and number of outcomes/events:** 1,040 children; 19 cases of TB  **Number of outcomes/events in relation to the number of candidate predictors (Events Per Variable):** 18 in normal Z-score, 6 in underweight, and 6 in overweight.  **Number of participants with any missing value (include predictors and outcomes):** 1.8% of Medellin. 4.8% from Cali, 1.7% Popayan were voluntarily withdrawn; 1.0% from Medellin, 5.0% from Cali, and 1.3% from popayan were lost of follow-up.  **Number of participants with missing data for each predictor**: 11 participants had missing zBMI data. For other variables, missing varied between 7 to 11.  **Handling of missing data (e.g., complete-case analysis, imputation, or other methods):** over sampling of 25% to compensate for the loss to follow-up. However, the calculated sample size was not achieved.  **Modelling method**   - chi-square test was calculated to establish significance (p <0.05) - adjusted prevalence ratio for infection was estimated using binomial regression - hazard ratios for infection and incident TB was adjusted by a Cox regression - multivariate models were built using BCG vaccination status, given that an additive interaction was found when using a stratified Mantel-Haenszel method. - The Kaplan-Meir method was used to estimate time to the disease for each age group. - Analyses were done using Stata 12.0, SPSS 22.0, and Epidat 3.1   **Assumptions:** not mentioned  **Adjustment factors used:** The incidence of active tuberculosis (TB) was adjusted for family and city intraclass correlation (clustering). | |
| Results | **Interpretation**  Underweight, being a household contact for more than 3 months, and being closer to the index case were associated with a higher prevalence of a positive tuberculin skin test (>= 10mm). The same factors showed a similar trend, although not significant, when a cutoff point of 5 mm was applied. The association between underweight and TB was not statistically significant.  **Comparison with other studies**  Reference in report number 12: Systematic review and meta-analysis found a similar prevalence of tuberculosis infection in children (51.4%). Reference in report 26: A similar study was conducted on Brazilian children. Reference in report number 30: the annual risk of infection in children's household contacts of adults with pulmonary TB is higher than the one reported in the general population in Russia. References in report number 31,32: Studies differ on the incidence in children in Zambia and South Africa, but are also calculated differently.  **Discussion of generalizability**  Not mentioned  **Strengths and limitations**  *Limitation*:   - The tuberculin test is not perfect; it is especially poor in BCG-vaccinated populations. It has cross-reactivity with non-tuberculosis mycobacteria, and its sensitivity decreases in immunocompromised patients. - The initial sample size was calculated exclusively for children. An inverse calculation was done for the cohort of children <5 years old, which consisted of an estimation of the power needed to detect a significant difference (95% confidence value) between the 2% incidence rate of active TB in individuals with a BCG scar vaccination (exposed) and the 6% of incidence in non-vaccinated individuals. - The tuberculin skin test was not available for every child in Medellin; only an incidental sample could access this test. - The estimated tuberculosis annual risk of infection is not comparable to traditional methods. Current TB contact tracing with tuberculin skin test is complemented by a commercial interferon-gamma assay.   *Strengths*: not mentioned | |
| Funding | Colciencias (grants for the Colombian Center for Tuberculosis Research). | |
| Conflict of interests | None of the authors have conflicts of interest relevant to this article to disclose. | |
| Notes | 14 February 2024 Email dionebunjumea@gmail.com Query about analysis. | |
| ***Risk of bias*** | | |
| **Bias** | **Authors' judgement** | **Support for judgement** |
| Study participation | Unclear risk | Although the population is well described, participants were recruited as household contacts. |
| Study Attrition | Low risk | Low missing data varied from 1 to 4.8% in each city. |
| Prognostic Factor Measurement | Low risk | Valid cut-off point (BMI 1 Z score) measured uniformly across participants, minimal missing data |
| Outcome Measurement | Low risk | Valid assessment of TB applicalbe to all participants. |
| Study Confounding | High risk | None of the pre-defined confounders were used (only adjusted by clustering and family), bivariate analysis for other confounders |
| Statistical Analysis and Reporting | Low risk | No conceptual framework for the selection of confounders, otherwise well described. |

Beshir 2019

| ***Study characteristics*** | | |
| Study details and sources | **Study design:** retrospective cohort study  **Source of data:** HIV/AIDS care clinic of Adama Referral Hospital and Medical College  **Study dates:** 1 January 2013 - 31 December 2017  **Setting:** outpatients  **Country:** Ethiopia | |
| Participants | **Participant eligibility**   - Inclusion:   - HIV-positive children younger than 14 years   - registered with the chronic HIV care and support program at the Hospital   - TB-free at enrollment (baseline) - Exclusion:   - incomplete baseline information (CD4 count, Hb levels)   - already started anti-TB treatment at enrollment (baseline)   **Recruitment method:** a simple random sampling technique was applied to a list of 633 HIV-infected children who had started art in the ART unit between March 2005 and January 2013.  **Participant description**   - Age: median age at baseline was 6 years (IQR 3-10 years) - Sex: 51.9% were boys   **Risk factors:**   - diabetes: no - undernutrition: yes - HIV infection: yes, all - recent TB infection: NR, likely not (TB free at baseline) - history of untreated or inadequately treated TB disease immunosuppressive therapy: NR - cigarette smokers: NR - drug or alcohol use disorders: NR - socioeconomic status: NR - other (specify)   - Residence: 68.5% urban; 31.5% rural   - Family size: 19.6% ≤2; 56.6% 3-4; 23.8% ≥%   Details of treatments received (for tuberculosis): NR | |
| Outcomes | **Tuberculosis (active tuberculosis or tuberculosis disease)**  Definition and method for measurement of outcome: (time to) incident active TB. This could be smear-positive pulmonary, smear-negative pulmonary, or extrapulmonary TB. Identified by signs and symptoms, lab tests, x-ray examinations, and/or initiation of anti-TB treatment.  Was the same outcome definition (and method for measurement) used in all patients? Yes  Type of outcome: incident active TB  Time of outcome occurrence or summary of duration of follow-up:   - Person-time: time from date of HIV/AIDS confirmation to occurrence of TB - Censoring: follow-up ended at first date of lost-to-follow-up, drop-out, transferring out, death by other causes, 31 December 2017   **Recurrence of TB**  Not measured | |
| Risk factor: undernutrition | Stunting: being 2 SD below the norm for height for age (WHO 2006 curve)  Underweight: being 2 SD below the norm of weight for age (WHO 2006 curve)  Wasting: being 2 SD below the norm of weight for height (WHO 2006 cuve) | |
| Sample size, missing data and analysis | **Number of participants and number of outcomes/events:**   - Number or participants: 428 - Outcomes: 67   - Pulmonary TB: 55   - Extrapulmonary TB: 12   **Number of outcomes/events in relation to the number of candidate predictors (Events Per Variable):**   - 12 confounders for 67 events (= 5 confounders per event)   **Number of participants with any missing value (include predictors and outcomes)**   - 38 were excluded at baseline due to incomplete data leaving 428 patients (for analysis)   **Number of participants with missing data for each predictor**   - Not reported. - Discussion reports that some data (like history of TB exposure) was often missing and hence, not incl in the analysis - Unavailable data: household income, housing condition, HIV viral load and other potential important predictors of TB   **Handling of missing data (e.g., complete-case analysis, imputation, or other methods):** NR, but were excluded from analysis  **Modelling method**   - Calculation of Z-scores by ENA for SMART 2011 - KM survival curves (staying TB free) - Univariate Cox PH regression: vars with p-value ≤0.25 were fitted in the multivariate model - Multivariate Cox PH regression   **Assumptions:** proportional hazards checked  **Adjustment factors:**   - Age (≤5, 6-10, ≥11) - Residence (urban/rural) - Family size (≤2, 3-4, ≥5) - Hemoglobin level (<10, ≥10 g/dl) - Functional status (working, ambulatory, bedridden) - WHO clinical staging (I or II, III or IV) - Underweight (normal, moderate (-2SD), severe) - Stunting (normal, moderate (-2SD), severe) - Wasting (normal, moderate (-2SD), severe) - Receiving Cotrimoxazole (yes/no) - Receiving Isoniazid preventive therapy (yes/no) - BCG vaccinated (yes/no) | |
| Results | **Interpretation**  Children who were moderately underweight at the beginning of ART therapy were at higher risk of developing TB than those who were not underweight. Children with moderate wasting were at a higher risk of developing TB than those with no wasting.  **Comparison with other studies**  The underweight finding was consistent with those of studies done in Kenya and the Democratic Republic of Congo (References in the report number 19 and 20)  **Discussion of generalizability**  Not reported  **Strengths and limitations**  *Strengths*  Not reported  *Limitations*:   - Some data, such as history of TB exposure, were frequently missing and could not be included in the data analysis. - Unavailability of data, including household income, housing condition, HIV viral load and other potentially important TB risk factors. | |
| Funding | Not available. | |
| Conflict of interests | The authors reported no conflicts of interests. | |
| Notes | 14 February 2024 Email tmulugeta79@yahoo.com about culture confirmation and missing data. | |
| ***Risk of bias*** | | |
| **Bias** | **Authors' judgement** | **Support for judgement** |
| Study participation | Unclear risk | Only about HIV children on ART and without TB. |
| Study Attrition | Unclear risk | 9.2% with incomplete data, 14% lost to follow-up. |
| Prognostic Factor Measurement | Low risk | Based on clinical standards, though missing data were not clearly described. |
| Outcome Measurement | Unclear risk | Same for all participants but unclear how many were culture-confimed. |
| Study Confounding | Low risk | All except immune suppresion and socioeconomic status (children). |
| Statistical Analysis and Reporting | Low risk | Adequately presented Cox regression analysis for the defined model. |

Cegielski 2012

| ***Study characteristics*** | | |
| Study details and sources | **Study design:** population-based cohort study  **Source of data:** Interviews, medical records and death certificates. Study dates: 1971-1992  **Setting:** Outpatient  **Countries:** 48 states in the United States | |
| Participants | **Participant eligibility:**  *Inclusion*: civilian, non-institutionalised US population from 48 states and participation in NHANES I.  *Exclusion*: reservation lands of American Indians (n=23,808), subjects with TB before the NHANES I. TB exposure without disease, primary infection without disease, and tuberculin skin test positivity without disease.  **Recruitment method:** Probability sample of the civilian noninstitutionalized US population aged 1-74 years  **Participant description:**   - Age: NHANES (1-79 years); NHEFS (25-74 years at their NHANES examination) - Sex: Men (40.1%), Women (59.9%)   **Risk factors:**   - diabetes: yes/no - undernutrition: BMI (<18.5; 18.5-<25; 25->30; >30) - HIV infection: NR - recent TB infection: NR - history of untreated or inadequately treated TB disease immunosuppressive therapy: NR - cigarette smokers: current smoking (yes/no) - drug or alcohol use disorders: alcohol consumption >7 drinks/week (yes/no) - socioeconomic status: annual income (<$10,000, $10,000-20,000, >$20,000) - other (specify)   - skinfold thickness   - arm muscle area   - race: white, black, other   - Hispanic ethnicity   - Residence in an urban area (urban, suburban, rural)   - residence in a designated poverty area   - immigrated to the US   - current smoking   - Medical history   **Details of treatments received:** not described | |
| Outcomes | **Tuberculosis (active tuberculosis or tuberculosis disease)**  Definition and method for measurement of outcome:  TB cases were ascertained through interviews (self-reported TB), medical records and death certificates (ICD-9 codes 010-018 and 137). For surveys, TB exposure, TB screening, and TB skin testing without active disease were not counted. For medical records and death certificates, TB exposure without the disease (ICD-9 code V01.1), primary infection without the disease (010.0) and TST positivity without the disease (795.5).  The author provided more information:  "Clinical criteria  A case that meets all the following criteria:  • A positive tuberculin skin test or positive interferon-gamma release assay for M. tuberculosis  • Other signs and symptoms compatible with tuberculosis (TB) (e.g., abnormal chest radiograph, abnormal  chest computerized tomography scan or other chest imaging study, or clinical evidence of current disease)  • Treatment with two or more anti-TB medications  • A completed diagnostic evaluation  Laboratory criteria for diagnosis  • Isolation of M. tuberculosis from a clinical specimen,\* OR  • Demonstration of M. tuberculosis complex from a clinical specimen by nucleic acid amplification test,\*\*  OR  • Demonstration of acid-fast bacilli in a clinical specimen when a culture has not been or cannot be  obtained or is falsely negative or contaminated.  Confirmed  A case that meets the clinical case definition or is laboratory confirmed."  Was the same outcome definition (and method for measurement) used in all patients? yes  Type of outcome: (e.g., single or combined endpoints) TB infection. single endpoint  Time of outcome occurrence or summary of duration of follow-up: Baseline data collection from 1971-1975.  Follow-up measurements (4 times) were taken between 1982 and 1992. Follow-up ended at the earliest date of a TB diagnosis or was censored on the date of the last observation.  **Recurrent tuberculosis:** not measured | |
| Risk factor: undernutrition | Definition and method for measurement: Body mass index (BMI), subcutaneous fat and lean skeletal muscle.   - BMI was categorised as low (<18.5), normal (18.5 to <25), overweight (25-30) or obese (>=30). - Subcutaneous fat was based on the sum of the right triceps and subscapular skinfold thickness. - Skeletal muscle was based on the cross-sectional area of the right mid-upper arm muscle (cm2) using Frisancho´s method.   Other measurements: Haemoglobin (serum albumin, iron, iron-binding capacity, transferrin, saturation, vitamin A, thiamine, riboflavin, and creatine) ply urinary thiamine, riboflavin, and creatine.  Timing of measurement: At the start of NHANES I (and during the 4 follow-ups).  Handling of predictors in the modelling: Categorical for BMI (low/normal/high): subcutaneous fat and skeletal muscle (low (<5th percentile)/normal/high (>60th percentile) on the basis of their sex-specific population distributions). | |
| Sample size, missing data and analysis | **Number of participants and number of outcomes/events:** 61/14189 totaling 209,013 person-years and averaging 15.8 years duration or 209013 person years. Crude incidence = 28.3/100000 person-years.  **Number of outcomes/events in relation to the number of candidate predictors (Events Per Variable)** Body mass index: <18.5 = 19; 18.5–<25 = 32; 25–<30 = 8; >30 = 2  12 confounders for 61 events (= 5 TB cases per confounder)  **Number of participants with any missing value (include predictors and outcomes):**   - 6.9% did not have a usable follow up data. - 218 participants were excluded because they had TB before NHANES I (14,189?) leaving 13,211 for the analysis cohort.   **Number of participants with missing data for each predictor:** not mentioned  **Handling of missing data (e.g., complete-case analysis, imputation, or other methods):** not reported, the number of the analysed dataset however, refers to the total population of 14,189 participants.  **Modelling method:**   - For continuous variables, the population-estimated distributions were compared using the t test or the Wilcoxon rank-sum test. - Groups were compared graphically by means of Kaplan-Meier plots and statistically by means of the log-rank test. - Control for potential confounding with multivariable Cox proportional hazards regression incorporating the complex survey design and sampling weights. - Predictor variables based on fewer than 5 actual TB cases were not included in the multivariable model. - Control variables were examined for effect modification and confounding by means of stratified analysis using Mantel-Haenszel methods and proportional hazards regression. - SAS 9.2 and SUDAAN 10.0 - A P value less than 0.05 was considered significant, and all statistical tests were 2-sided.   **Assumptions:** it was ensured that each variable and interaction term entered into the model satisfied the PH assumption  **Adjustment factors used:** Age, sex, race, Hispanic ethnicity, immigration, urban/rural residence, income, residence in a designated poverty area, excess alcohol consumption, smoking, and diabetes mellitus  The author provided additional data:  "To select variables for inclusion (adjustment) in multivariable models, I used classic biostatistical/epidemiological methods. I determined whether there was an association between the co-variate and TB, and I determined whether there was an association between the co-variate and nutritional status indicators. For variables that were associated with both main predictor and main outcome (the definition of potential confounding), I carried out stratified analysis using a Breslow-Day test, including variables with P<0.2 in the starting regression models.  In multivariable regression analysis, I retained in the final model variables that were statistically significant and variables that when added into the model or removed from the model altered the main ratio measure of effect by 10% or more. To derive the final model, I used a backwards elimination approach. I also used a “best subsets” approach developed at Emory to find a parsimonious set of covariates that gave the best model fit and that resulted in a stable ratio measure of effect.  I have carried out many sensitivity analyses to convince myself that my results were correct. I presented these to my dissertation committee, but these have not been published. For example, I looked separately at TB cases diagnosed in the first 10 years and TB cases diagnosed from years 10 to 21. I looked separately at cases ascertained through interview, through hospital records, and through death certificates. I analyzed various cut-off points for categorizing the anthropometric and laboratory predictor variables. I analyzed the possibility that cases were under-diagnosed and over-diagnosed. The findings remained consistent.  The most important sensitivity analysis was external validation against US National TB surveillance data from the CDC. The weighted number of cases in my analysis cohort and the actual reported number of cases in the country for the same age cohort and period of years were nearly identical." (published in the included paper) | |
| Results | **Interpretation:**   - TB incidence among adults with normal BMI was 24.7 per 100,000 person-years (95% CI: 13.0, 36.3)   - Underweight 260.2 (95% CI: 98.6, 421.8) – aHR: 12.43 (95% CI: 5.75, 26.95)   - Overweight 8.9 (95% CI: 2.2, 15.6) – aHR: 0.28 (95% CI: 0.13, 0.63)   - Obese 5.1 (95% CI: 0.0, 10.5) – aHR: 0.20 (95% CI: 0.07, 0.62)   - estimated TB incidence rates per 100,000 person-years, respectively   Mean BMI, skinfold thickness, and arm muscle were significantly lower in persons who subsequently developed TB, except for arm muscle area among females.  Mean skinfold thickness was 14.7 mm lower, and arm muscle area was 4.7 cm2 lower among persons who developed TB than those who did not.  As skinfold thickness increased from low (170.6 95% CI: 59.6, 281.5)to normal (22.4 170.6 95% CI: 10.6, 34.2) to high (7.5 95% CI: 1.9, 13.0), the TB incidence rate decreased. The pattern is similar, with a smaller amplitude.  **Comparison with other studies:**   - (References in the report 17–19) Cohort studies carried out among US Navy recruits and in Norway have demonstrated increased TB risk in thin persons. - (References in the report 20) In a study in Philadelphia, Pennsylvania, men with low vitamin A and C levels had a higher TB incidence than men with adequate levels. - (References in the report 21) In New York City, a clinical trial demonstrated that multivitamin-mineral supplements decreased TB incidence among family members of active TB cases. - (References in the report 22, 23) Mounting evidence suggests that obesity may decrease the risk of TB. - (References in the report 23) In China, a study of more than 42,000 elderly persons found that TB incidence was significantly lower in overweight persons than in normal-weight controls.   Discussion of generalizability: Because the population’s nutritional profile has changed, the results presented here do not reflect the population-attributable risk after 1992, but they remain valid estimates of TB risk relative to nutritional indicators measured on a fixed scale, such as BMI categories and standard laboratory criteria. In addition, risk ratios based on absolute measures of nutritional status may be used to estimate attributable risk in populations with known, corresponding measures of nutritional status.  **Strengths and limitations**  *Limitations*:   - Because follow-up interviews did not ask specifically about TB, the data may not have captured every incident of TB, especially cases not resulting in hospitalisation or death. - The nutritional status of the US population has changed. Even though obesity was not widespread in the 1970s, many people are sceptical that undernutrition was ever a substantial problem in the United States. Indeed, only 2.2% of the cohort had low BMI.   *Strengths*:   - NHANES I is documented extensively with assessments of internal and external validity - The standardised, validated indicators of nutritional status were based on body measurements and laboratory data. - Multivariable statistical methods were based on the complex study design - This is the only longitudinal cohort study of nutritional status and TB incidence based on a nationally representative sample - They separated the risk of TB in relation to adipose tissue, somatic protein, and individual micronutrients | |
| Funding | There are no direct funders for this study | |
| Conflict of interests | The authors declare no competing interests | |
| Notes | Questions to the authors  29 August 2023 - Email: gzc2@cdc.gov (2020 paper) pcegielski@cdc.gov (2012 paper)   1. Variables for adjustments 2. Person-year for each BMI category   25 January 2024 - Email peter.cegielski@emory.edu (obtained online)   1. Diagnostic criteria for TB. 2. Missing data/selection of variables for adjustment. 3. Outlier results in the meta-analysis (additional explanations).   12 February 2024 - Response: We received a thorough response with further details about the study and the context, which we incorporated into the risk of bias assessment, GRADE assessments and the discussion. | |
| ***Risk of bias*** | | |
| **Bias** | **Authors' judgement** | **Support for judgement** |
| Study participation | Low risk | Large national database (NHANES I Study). |
| Study Attrition | Unclear risk | 6.9% had no usable follow-up, no description of missing data. |
| Prognostic Factor Measurement | Low risk | Adequately presented Cox regression analysis for the defined model. |
| Outcome Measurement | Unclear risk | Same for all participants (interviews and inspection of health records), the author clarified that it could be either clinical or laboratory (no available % of culture-confirmed). |
| Study Confounding | Unclear risk | All except HIV and immunosuppressive therapy. |
| Statistical Analysis and Reporting | Low risk | The analysis technique was described and the author provided detail on the selection of confounder and sensitivity analyses. |

Chang 2015

| ***Study characteristics*** | | |
| Study details and sources | **Study design:**retrospective cohort study  **Source of data:** clinical health records  **Study dates:** May 2005 to 29 February 2012  **Setting:** Outpatient  **Countries:** Nigeria | |
| Participants | **Participant eligibility**  HIV-1-infected, antiretroviral-naive adults (age ≥15 years) who initiated ART on a standard first-line zidovudine (AZT)- or tenofovir (TDF)-containing regimen; and had ≥15 months of FU. *Exclusion*: Patients who did not include consent for their data to be used.  **Recruitment method**  Electronic data collected from 2004 to 2012 through the Harvard/APIN PEP-FAR program as part of routine clinical care. The cohort included 30 ART sites in 9 of 36 Nigerian states.  **Participant description:**   - Age: age ≥15 years (15-29, 30-34, 35-41, 42+) - Sex: Male (35.5%), Female (64.7%)   **Risk factors:**   - diabetes: not reported - undernutrition: BMI <16 (severely underweight), 16–18.49 (underweight), 18.5–24.99 (normal), 25–29.99 (overweight), ≥30 (obese). - HIV infection: ART treatment for CD4+ count <200 cells/mm3 or with symptoms and CD4+ cell count <350 cells/mm3.   - Initial ART regimen: 3 drug combination (TDF, XTC, EFV, NVP, AZT, 3TC) - recent TB infection: an initial TB date 6 months before or up to 3 months after ART therapy (Prevalent TB; yet were excluded from analyses) - history of untreated or inadequately treated TB disease immunosuppressive therapy: not reported - cigarette smokers: not reported - drug or alcohol use disorders: not reported - socioeconomic status: Employment status: non-income generating, laborer/service worker, manager/professional. Education: none, primary, secondary, tertiary - other (specify)   - Anemia: no anemia (≥12 g/dL for women and ≥13 g/dL for men), mild/moderate anemia (8–11.9 g/dL for women and 8–12.9 g/dL for men), and severe anemia (<8 g/dL).   - Viral load: 0-1000, 1001-10 000, 10 001-100 000, 101 000-1 000 000; suppressed (≤400 copies/mL) or unsuppressed (>400 copies/mL)   - Adherence: ≥95%, 80%–94.9%, and <80%   - Martial status: single, married; separated/divorced, widowed   - HIV risk factor: Heterosexual only, Other/multiple   - ART enrollment year: 2005-2010   - Treatment site type: secondary hospital, tertiary hospital   - WHO clinical stage: 1, 2, 3, 4.   - Hepatitis B: no, yes   - Hepatitis C: no, yes   - CD4+ cell count, cells/uL: <= 100, 101-200, 201-350, >350   Details of treatments received (treatment for diabetes, undernutrition and other concomitant conditions): triple-drug combination ART treatment. | |
| Outcomes | **Tuberculosis (active tuberculosis or tuberculosis disease)**  Definition and method for measurement of outcome: "TB testing with chest radiography and acid-fast bacilli (AFB) microscopy, which required collection of 3 sputum specimens over 2 consecutive days, assisted by symptom assessment (current cough, night sweats, weight loss, fever, and history of contact with a person with chronic cough).  Definition: All AFB smear-positive cases were diagnosed as active TB. If smear-negative, the treating clinician made a diagnosis of TB based on clinical symptoms and radiologic and histologic results."  Was the same outcome definition (and method for measurement) used in all patients? Yes  Type of outcome: Single outcome  “Prevalent TB” was defined as a case with an initial TB date 6 months before or up to 3 months after ART initiation.  “Incident TB” was defined as a case with an initial TB date after the first 3 months on ART.  Time of outcome occurrence or summary of duration of follow-up: the time at risk for incident TB began at month 3 after ART initiation and ended on either the date of the first recorded TB diagnosis, for those who developed incident TB, or the date of the last recorded ART pick-up, for those who were never diagnosed with TB during the observation period.  Censoring: occurred at discount. from the program due to death, transfer, withdrawal, or loss-to-FU, or at the end of the study (29 February 2012)  **Recurrent TB**  Not measured | |
| Risk factor: undernutrition | Definition and method for measurement: BMI <16 (severely underweight), 16–18.49 (underweight), 18.5–24.99 (normal), 25–29.99 (overweight), ≥30 (obese) - clinically assessed.  Timing of measurement: at baseline.  Handling of predictors in the modelling: categorized by BMI categories mentioned above. Included in the analysis as a time-dependent variable. | |
| Sample size, missing data and analysis | **Number of participants, number of outcomes/events and amount of accumulated person-years (PY)**: Of 32,611 patients included in the incident TB analyses, 2021 (6.2%) developed active incident TB after ART initiation. The median follow-up time since ART initiation for the 32 611 patients was 29.2 months (6 years) (interquartile range [IQR], 17.8–43.2), contributing a total of 78 228 PY at risk for TB. The overall TB incidence rate was 25.8 cases per 1000 PY on ART (95% confidence interval [CI], 24.7–27.0).  **Number of participants, person-years and outcomes/events in relation to the number of candidate predictors (Events Per Variable):** 17 confounders for 2021 TB cases (118 events of TB per confounder)  **Number of participants with any missing value:** A total of 19,615 (60.1%) participants had at least 1 variable with missing data (all participants minus complete cases)  **Number of participants with missing data for each predictor:** NR  **Handling of missing data (e.g., complete-case analysis, imputation, or other methods):** To address potential bias resulting from missing data, 10 imputed datasets were created in which missing values for fixed and time-updated variables were imputed using the “two-fold” fully conditional specification algorithm.  Analyses were performed for both the dataset with “complete cases” (n=12,996) and “multiple imputations” (n=32611).  **Modelling method:**   - all statistical analysis were performed using Stata version 13.1 - Tuberculosis incidence rates were calculated as the number of new cases of TB divided by total person-years (PY) at risk, and expressed per 1000 PY. - Cox proportional hazards models were generated to evaluate risk factors for incident TB during ART. - The log-rank test was used for bivariate analyses, and variables with P ≤ .20 were considered for inclusion in multivariate Cox models. All variables with a P value ≤0.05 in the multivar analysis and additional clinically relevant info were retained in the final models. - The proportionality assumption was tested for each predictor by graphing the survival functions and using Schoenfeld residuals.   Assumptions: Proportional hazards assumption.  Adjustment factors used: Age, Sex, Education, Employment status, Marital status, HIV risk factor, ART enrollment year, Treatment site type, Initial ART regimen, WHO clinical stage, Hepatitis B, Hepatitis C, BMI, CD4 cell count, Viral load status, Anemia, Severe anaemia. | |
| Results | **Interpretation**  The overall TB incidence rate on ART was 25.8 cases per 1000 PY (95% confidence interval [CI], 24.7–27.0). In contrast, the annual rate of incident TB during ART for all patients without TB at ART initiation decreased each calendar year from 49.4 cases per 1000 PY (95% CI, 40.8–59.8) in 2006 to 13.6 cases per 1000 PY (95% CI, 12.2–15.1) in 2011.   - Severely underweight patients had four times the risk, and obese patients had half the risk of developing TB compared with patients of normal weight. - Underweight males had a higher TB risk than underweight females (results not shown in table).   **Comparison with other studies**   - (Reference in the report 22) A South Africa study (n = 7536) reported an overall incidence rate of 42 cases per 1000 PY over a median follow-up of 21.4 months - (Reference in the report 23) A recent Tanzania study (n = 53 056) reported 44 cases per 1000 PY among ART patients over a median follow-up of 24 months. - (References in the report 5–7, 22, 25–29) TB incidence declined with the duration of ART, which likely reflects the concurrent increase in CD4+ cell count and immune reconstitution due to ART. - (References in the report 5–7, 22–30, 35–38) Previous studies reported that poor immunologic and/or virologic status, low BMI, and anaemia were associated with incident TB among ART patients]. - (35, 38) Two smaller studies in Mozambique and Nigeria reported an association between adherence evaluated once and incident TB, but the large Tanzania study (23) found no association between time-updated adherence and incident TB [23].   **Discussion of generalizability**  Not mentioned.  **Strengths and limitations**  *Limitations*:   - The data were limited by missing information - Although TB testing was done in accordance with the national NTBLCP guidelines, which included AFB testing, radiology, and histology, assisted by symptom screening, the possibility of missing TB cases, including extrapulmonary TB whose diagnosis in resource-limited settings relies primarily on clinical symptoms, and misdiagnosed cases cannot be ignored. - Furthermore, because patients may present to the clinic at varying stages of TB disease and reporting of test results may lag, the TB diagnosis date is only a proxy for the time of initiation of active TB disease; they used the earliest TB date based on available records. - The data did not allow us to distinguish true reinfections from recurrences in all cases accurately; therefore, they did not include patients with prevalent TB in the incidence analysis and censored patients who developed incident TB after their first occurrence.   *Strengths*:   - This is the first multicenter assessment of TB incidence and risk factors in ART patients in Nigeria, which has the second highest HIV burden and highest TB burden in Africa. - The large number of patients in our program and follow-up period of over six years (32 611 patients observed for a median of 29.2 months, totalling 78 228 PY) contribute to its significant statistical power, providing stronger evidence to support the reports that found significant correlations between immunologic, virologic, and nutritional statuses with incident TB. - Examination of relevant clinical risk factors as time-dependent covariates for the entire length of each patient’s observation period. - This is the first study to show that poor ART adherence is a continuous significant predictor of incident TB, indicating the value of adherence monitoring as a TB intervention. - This study is the first to report that gender is not an independent risk factor but an effect modifier in the significant associations of BMI and anaemia with TB, suggesting gender differences in nutrition’s role in TB immunity. | |
| Funding | "This work was supported by the US Department of Health and Human Services, Health Resources and Services Administration (grant U51HA02522) and the Centers for Disease Control and Prevention through a cooperative agreement with the AIDS Prevention Initiative in Nigeria (grant PS 001058)*."* | |
| Conflict of interests | The authors report no conflict of interests. | |
| Notes | 29 August 2023 Email to pkanki@hsph.harvard.edu requiring a number of people, PYs and a number of events according to the BMI categories used for both the complete case analysis and the multiple imputations analysis. 1 September 2023: The author (Chang, Charlotte Ajeong) sent a Word document with further details and clarified that multiple imputation analyses were used:  "The risk factor analyses used time-varying covariates, which would be difficult to present in these summary tables, so these tables show the subjects' baseline BMI categories. For the complete case table, I excluded only observations that were missing constant (not time-varying) variables. For the multiple imputation table, 10 imputed datasets were created, so the sums for all 10 datasets are shown, but these could easily be divided by 10 to show the average. Please let me know if you have any questions. If you would like the data shown in a different way, I can look at it further." | |
| ***Risk of bias*** | | |
| **Bias** | **Authors' judgement** | **Support for judgement** |
| Study participation | Unclear risk | Focused on HIV-1-infected individuals on ART. |
| Study Attrition | High risk | 55% attrition and not well-described (due to missing BMI data). |
| Prognostic Factor Measurement | Low risk | Standard definitions used for all participants. |
| Outcome Measurement | Unclear risk | Same for all participants but unclear how many were smear/culture confimed. |
| Study Confounding | Unclear risk | All except DM, history of TB, smoking, alcohol use, immunosuppressive therapy. |
| Statistical Analysis and Reporting | Low risk | Adequately presented Cox regression analysis for the defined model. |

Chan-Yeung 2007

| ***Study characteristics*** | | |
| Study details and sources | **Study design:** cohort study (elderly homes)  **Source of data:** questionnaires and clinical assessment.  **Study dates:** July 2000 - November 30, 2005.  **Setting:** Outpatient  **Countries:** China (Hong Kong) | |
| Participants | **Participant eligibility**  Residents are in 35 old-age homes in 2 districts of Hong Kong. *Exclusion*: residents (n = 27) with active TB or on anti-TB treatment at baseline.  Recruitment method: interviews using short questionnaires and clinical assessment.  **Participant Description:**   - Age: (<70, 70–79, 80–89, >=90), mean age 82.4 +/- 8.3 - Sex: Male (28,3%), Female (71.7%)   **Risk factors:**   - diabetes: not reported. - undernutrition: BMI (<18.5 vs >=18.5); - HIV infection: not reported. - recent TB infection: not reported, with active or on treatment excluded. - history of untreated or inadequately treated TB disease immunosuppressive therapy: not reported. - cigarette smokers: (non-smoker; ever smoker) - drug or alcohol use disorders: drug abuse (yes vs no). - socioeconomic status: education level (none; primary; secondary; or tertiary) - other (specify)   - physical activity, mental status, mobility, and degree of incontinence were assessed using the Norton score (1–20).   - TST positivity (>=10 mm induration vs <10 mm).   - total duration in old age home (years)   - marital status (married; widowed; separated; or single)   - place of birth (Hong Kong; China or Macau; other)   - use of corticosteroids (yes vs no)   - history of major comorbid illness: (yes vs no)     - cerebrovascular accident     - hypertension     - ischemic heart disease     - chronic obstructive lung disease     - cancer     - chronic renal failure     - gastrectomy   Details of treatments received (treatment for diabetes, undernutrition and other concurrent conditions): none mentioned. | |
| Outcomes | **Tuberculosis (active tuberculosis or tuberculosis disease)**  Definition and method for measurement of outcome: Measurement: A two-step tuberculin skin test (TST) of the tuberculin PPD-RT23 was performed. A positive tuberculin reaction was defined as one with 10 mm induration or more at 48 to 72 hours. In subjects with a negative reaction (<10 mm induration), the test was repeated 2 weeks after the first testing. In addition, chest x-ray examination was performed, and sputum examination for acid-fast bacilli was requested on those with radiological abnormalities suggestive of tuberculosis, active or inactive.  Definition: see below  Was the same outcome definition (and method for measurement) used in all patients?: No (20 per positive culture, 14 per clinical examination or radiological/histological findings together with clinical and/or radiological response after ≥6 mo of drug treatment.  Type of outcome: (e.g., single or combined endpoints): incidence of TB; single outcome.  Time of outcome occurrence or summary of duration of follow-up: Participants were followed from the day of the initial survey until: diagnosis of TB, death from any cause, end of study on 30 Nov 2006. Participants with active TB or using TB-drugs at baseline (time of survey) contributed to person-time of FU from the time they completed treatment.  **Recurrent TB**  Not measured. | |
| Risk factor: undernutrition | Definition and method for measurement: Body mass index (BMI) >= 18.5 and < 18.5 (underweight) according to WHO recommendations for Asian Populations (ref 10)  Timing of measurement: at baseline.  Handling of predictors in the modeling: categorized in underweight (< 18.5) and non-underweight (>=18.5). | |
| Sample size, missing data and analysis | Number of participants, number of outcomes/events and amount of accumulated person-years (PY): 4,212 participants; 34 cases of incident TB (323 per 100,000 person-years)  Number of participants, person-years and outcomes/events in relation to the number of candidate predictors (Events Per Variable): BMI >=18.5: n=2912, TB=19; BMI < 18.5: n=1165, TB= 14  5 Confounders were analyzed for 33 cases of TB (6 cases per confounder)  Number of participants with any missing value (including predictors and outcomes): There were some missing.  Number of participants with missing data for each predictor: Table 1 shows that 135 people, including 1 case of TB, had missing BMI data.  Handling of missing data (e.g., complete-case analysis, imputation, or other methods): not mentioned, likely excluded from analysis.  Modelling method:   - Chi-square and t-tests were conducted to compare differences in demographic and personal characteristics and medical illnesses of those who developed TB on follow-up and those who did not. - Cox regression analysis was carried out separately for one-step and two-step testing to determine whether tuberculin reactivity predicts the development of active TB on follow-up. Potential confounders were included only when their univariate analysis was statistically significant. - BMI was also analyzed as a risk factor for TB, stratified by TST cutoff and outcome, yet many more are missing.   Assumptions: not mentioned  Adjustment factors used: sex, age, smoking habits, one-step TST and BMI. | |
| Results | **Interpretation**  "34 residents developed active TB (323 per 100,000 person-years) during follow-up. The only significant risk factors associated with the development of active TB were positive TST according to one-step testing (adjusted odds ratio (OR) 5 2.91, 95% confidence interval (CI) 5 1.26–6.74) and a BMI less than 18.5 (adjusted OR 5 3.15, 95% CI 5 1.45–6.86). Residents with a BMI less than 18.5 and a negative TST also had a greater risk of active TB than residents with a BMI greater than 18.5 and negative TST (adjusted OR 5 4.36, 95% CI 5 1.04–18.3), whereas those with a positive TST had the highest risk (adjusted OR 5 10.2, 95% CI 5 2.63–39.4). Two-step testing increased the sensitivity but reduced the specificity of TST in identifying active TB on follow-up."  **Comparison with other studies**  (Reference in the report 10) In residents with a BMI of less than 18.5, a level recognized by the WHO as underweight in Asians, the tuberculin reactivity was lower, and the risk of active TB was greater, irrespective of tuberculin status.  **Discussion of generalizability**  Not mentioned  **Strengths and limitations**  Not mentioned | |
| Funding | This study was supported by the SK Yee Medical Foundation of Hong Kong. | |
| Conflict of interests | Not available. | |
| Notes | 14 February 2024 Email: mmwchan@hkucc.hku.hk requesting data on culture and adjustment. | |
| ***Risk of bias*** | | |
| **Bias** | **Authors' judgement** | **Support for judgement** |
| Study participation | Unclear risk | Focused on homes for elderly individuals in two districts in one city in a TB program. |
| Study Attrition | Low risk | Minimal attrition (3.2%). |
| Prognostic Factor Measurement | Low risk | Same standard for all participants according to the WHO/Asia criteria. |
| Outcome Measurement | Unclear risk | Same for all participants but unclear how many were culture-confimed. |
| Study Confounding | Unclear risk | All except DM, history of TB, HIV, immunosuppressive therapy. |
| Statistical Analysis and Reporting | Low risk | Adequately presented Cox regression analysis for the defined model. |

Chen 2022

| ***Study characteristics*** | | |
| Study details and sources | **Study design:** population-based prospective cohort study  **Source of data:** Face-to-face surveys and clinical examination.  **Study dates:** June 2013 - December 2015  **Setting:** Outpatient  **Countries:** China | |
| Participants | **Participant eligibility**  Participants who had resided in the 10 randomly selected study sites (communities) before screening for at least 6 months.  **Exclusion criteria**  Refusion to participation, or temporary residents; age under 15 years and active TB in baseline screening.  Recruitment method: 10 randomly selected communities in the southwestern mountainous region of China. Home visits and face-to-face surveys.  **Participant Description:**   - Age: over 15 years (mean 45.2 years ± 17.0) - Sex: Male (47.6%), Female (52.4%)   **Risk factors:**   - Diabetes: Known diabetes: 639 (2.5%) - undernutrition: BMI is categorized in BMI into three groups: underweight (<18.5 kg/m2): (2042 /7.9%), normal (18.5 – 24.0 kg/m2): 16,009 (61.5%), overweight or obese (≥24.0 kg/m2): 7,971 (30.6%) - HIV infection: Known HIV/AIDS: 21 (0.1%) - recent TB infection: Previously treated TB: yes (138 (0.5%)), no (25,884 (99.5%)) - history of untreated or inadequately treated TB disease immunosuppressive therapy: NP - cigarette smokers: never (19,308 (74.2%)), former (822 (3.2%)), current (5,892 (22.6%)) - drug or alcohol use disorders: never (20,532 (78.9%)), former (1,066 (4.1%)), current (4,424 (17.0%)) - socioeconomic status: education level: 2,048 (7.9%) illiterate or semi-illiterate; 4,671 (18.0%) primary school; 15,126 (58.1%) secondary school; 4,123 (15.8%) college and above; 54 (0.2%) unknown, annual family income per capita (<the median of 7200 Renminbi (RMB), ≥the median of 7200 RMB) - other (specify)   - Ethnicity (Han, other minority),   - marital status (married, single, widowed/divorced/ separated/other)   - close contact to an index TB case (yes, no)   - chronic bronchitis (yes, no)   - pneumoconiosis (yes, no)   - BCG vaccine scar in the arm (yes, no).   - Information on previously treated TB cases (yes, no)   Details of treatments received (treatment for diabetes, undernutrition and other concomitant conditions): Not reported | |
| Outcomes | **Tuberculosis (active tuberculosis or tuberculosis disease)**  Definition and method for measuring outcome: Incident TB was assessed after a home visit. TB symptoms positivity was defined as suspect, and then suspects underwent chest X-ray (CXR) and health examination. Those with abnormal radiographs got a sputum smear test (three sputum samples) for laboratory diagnosis. Patients with a smear of acid-fast bacilli positivity were diagnosed with laboratory-confirmed TB.  People with abnormal radiographs and negative smears, whose CXRs were reviewed by the diagnostic committee, were defined as clinically diagnosed TB.  Was the same outcome definition (and measurement method) used in all patients? Yes  Type of outcome: incident TB and time-to-incident of active TB in the second and third rounds of screening.  Time of outcome occurrence or summary of duration of follow-up: The participants in the fixed cohort were prospectively followed up until the occurrence of TB, death, moving out of the study field, refusing to participate in the 2nd and 3rd round of screening or the end of the study in December of 2015.  **Recurrent TB**  Not measured | |
| Risk factor: undernutrition | Definition and method for measurement: BMI is categorized into three groups: underweight (<18.5kg/m2), normal (18.5 – 24.0kg/m2), overweight or obese (≥24.0kg/m2). Data was actively measured by the community health workers (CHW).  Timing of measurement: at baseline.  Handling of predictors in the modelling: categorized as mentioned above. | |
| Sample size, missing data and analysis | Number of participants, number of outcomes/events and amount of accumulated person-years (PY): 26,022 participants; overall 43 cases developed TB in the observed 44574.4 PY. The TB incidence was 96.5 per 100 000 PY (95% CI 69.8 to 129.9).  Number of participants, person-years and outcomes/events in relation to the number of candidate predictors (Events Per Variable): 26,022 Participants; 43 cases of TB; 44 574.4 PYs.   - Underweight: 2,042 participants; 6 cases; 3464.8 PY - Normal weight: 16,009 participants;31 cases; 27382 PY - Overweight/obese: 7,971 participants;6 cases; 13727.7 PY   The IRs of TB were 173.2 (95% CI 63.6 to 376.9) for underweight groups, 113.2 (95% CI 76.9 to 160.7) for normal-weight, and 43.7 (95% CI 16.0 to 95.1) for overweight or obese groups per 100 000 person-years. Eight confounders for 43 cases (total): thus, per confounder, 5 cases.  Number of participants with any missing value (including predictors and outcomes): not reported.  Number of participants with missing data for each predictor: not reported.  Handling of missing data (e.g., complete-case analysis, imputation, or other methods): not reported.  Modelling method:   - The cohort characteristics were described by the proportions in BMI categories. - Person-years and TB IR were computed by BMI level and other covariates in all patients. - Kaplan-Meier analysis and log-rank test to compare the cumulative hazard of time to TB incidence in three BMI levels. - Denary logarithm-transferred TB incidence was regressed with the averaged BMI in six groups (BMI <18.5, 18.5–20.4, 20.5–22.4, 22.5–23.9, 24–27.9 and ≥28 kg/m2), then the determination coefficient R2 and adjusted R2 were used to evaluate the model. - Stepwise multivariate Cox proportional hazard regression models to estimate the HR and the corresponding 95% CI of factors. Normal BMI was set as the reference level. - The effect of DM was corrected by introducing it as a covariate in a different COX regression. - The association between BMI and TB development was evaluated in stratifications of gender and age by adjusting the confounders.   Assumptions: The restricted cubic spline (RCS) regression evaluated the potential nonlinear association.  Adjustment factors used: Adjusted with covariates: age, sex, ethnicity, marital status, previously treated TB, smoking status, alcohol use and known DM. | |
| Results | **Interpretation**  During the follow-up of 2.25 years, 43 cases developed TB in 44 574.4 person-years. TB incidence was 96.5 per 100 000 person-years.   - The negative log-linear relationship between BMI and TB incidence was fitted (adjusted R2=0.76). - Overweight or obese was associated with a lower risk of TB compared with normal weight (adjusted HR (aHR) 0.34, 95% CI 0.14 to 0.83). - Underweight was associated with a higher risk of TB compared to normal weight: (aHR 1.25 (0.52 – 3.04) - The association between underweight and TB was also modelled for subgroups of sex and age.   **Comparison with other studies**   - (Reference 4) The finding of the log-linear inverse association between BMI and TB incidence was consistent with a previous systemic review. - (Reference 15, 16) Population-based cohorts reported by Cegielski et al. in the USA and Pealing et al. in the UK stated the inverse association in low disease burden and high-income settings. - (Reference 8, 17) Zhang et al. and Cheng et al. reported a significantly higher risk (OR 1.55, 95% CI 1.09 to 2.22) or hazard (HR 2.33, 95% CI 1.32 to 4.12) of TB in the underweight group compared with the normal-weight group. - (Reference 18) Yen et al. reported that in a Taiwan cohort, compared with the normal-weight group, overweight (OR 0.67, 95% CI 0.49 to 0.91) and obesity (OR 0.43, 95% CI 0.28 to 0.67) were protective factors to disease incidence. - (Reference 19) Kim et al. reported a consistent inverse association between BMI and TB. - (29) Lin et al. stated that causal mediation is between BMI, DM, and TB.   Discussion of generalizability: the generalization of this finding should be treated with caution because the cohort sample was unrepresentative for other regions; more evidence was needed to confirm the cut-off value of BMI and the yield of screening.  **Strengths and limitations.**  *Strengths*:   - The study included many community participants in the southwestern mountainous region of China. - The study presented TB incidence under a high disease burden setting in Chinese adults. - The study provided evidence of a log-linear inverse dose-response relationship between BMI and TB incidence at the population level and of a dose-response association between BMI and individual risk. - After adjusting confounders, the overall and stratified analyses were performed. The protective effect of the higher BMI, presented in the general population, subgroups of women, and the elderly, might contribute to the TB control policy.   *Limitations*:   - The limited funding and resources led to only two years of follow-up and conducting three rounds of screening. - The cohort DM prevalence (2.5%) was lower than 9.7% in China and 6.7% in Yunnan around 2010; thus, the study failed to analyze the modified effect of DM on BMI. - BMI was measured at a single point at the baseline. It could change over time, and the dynamic BMI might influence TB in the follow-up. - Several covariates, such as smoking and drinking habits, were self-reported by participants; therefore, the association would be imprecise without accurate measurement. - A small sample size for some subpopulations and the low prevalence of comorbidities in the baseline led to an insignificant association. - The false-negative and underestimation of TB diagnosis would occur due to not using CT and culture in limited-resource settings. - The baseline survey did not include food intake and dietary information; therefore, they could not quantify the energy–BMI–TB interaction here. | |
| Funding | The National Twelfth Five-year Mega supported this work- -Scientific Projects of Infectious Diseases in China (grant number 2013ZX10003–004-001) and the Yunnan Health training project of high-level talents (grant number H-2019027). The funders had no role in study design, data collection and analysis, manuscript writing, and the decision to publish. | |
| Conflict of interests | None declared. | |
| Notes | 08.09.2023 Email: xulinth@hotmail.com   1. How many of the TB cases for the different categories of BMI were lab-confirmed and clinically diagnosed TB? | |
| ***Risk of bias*** | | |
| **Bias** | **Authors' judgement** | **Support for judgement** |
| Study participation | Low risk | Population based-sampling from 6 different sites |
| Study Attrition | Low risk | All participants were included in the analysis. |
| Prognostic Factor Measurement | Low risk | BMI was based on clinical assessments. |
| Outcome Measurement | Unclear risk | TB by smear positive or clinically diagnosed if smear-negative, unclear how many were culture positive. |
| Study Confounding | Unclear risk | All except history of TB, immunosuppressive therapy. |
| Statistical Analysis and Reporting | Low risk | Adequately presented Cox regression analysis for the defined model. |

Cheng 2020

| ***Study characteristics*** | | |
| Study details and sources | **Study design**: prospective population-based cohort  **Source of data**: Survey and Clinical follow-up  **Study dates**: July 2013 - September 2015  **Setting**: Outpatient  **Countries**: China | |
| Participants | **Participant eligibility**  A total of 34076 elderly (65 or older) without TB from 27 study sites (townships and communities) who continuously lived in the village or community for six months or longer were eligible for our prospective study.  Exclusion: active TB at baseline.  **Recruitment method**  (Also see ref 17) Random sampling of 10 townships and 17 communities in 10 Chinese countries. For each elderly, a door-to-door interview for data collection, a physical examination for calculating body mass index (BMI), and a chest X-ray (CXR) examination were provided for them.  **Participant description**   - Age: mean age of the study cohort was 73.0 (± 6.36 years old). - Sex: Male (46.7%), Female (53.3%) - Risk factors: - diabetes: 7% - undernutrition:   - BMI as underweight (BMI < 18.5 kg/m2) 10.5%   - Normal weight (≥ 18.5 kg/m2 and < 24.0 kg/m2) 60.9%   - Overweight and obese (≥ 24.0 kg/m2) 28.6%   - Unknown - HIV infection: not mentioned - Recent TB infection: previously treated TB 1.7% - History of untreated or inadequately treated TB disease immunosuppressive therapy: not mentioned - Cigarette smokers: non-smoker 80.3%, ex/current smoker 19.7%, unknown - Drug or alcohol use disorders: alcohol drinker 19.1% (others: non-drinker 80.9%, unknown 0.1%) - Socioeconomic status: - Education: illiteracy 35.5%, primary to secondary 50.4%, high school and above 10.7%, unknown 3.6%.Annual average income pp in fam: low income <2300 RMB 48.4%, 2300-9999 35%, >= 10 000 48.4%. - other (specify)   - Disease history: classified into yes or no or unknown groups.   - Nationality: Han 86.7%, others, unknown.   - Marital status: married 72.9%, others, unknown   - Residence: local resident 91.9%, others   - Chronic bronchitis: no, yes 4.8%, unknown.   - Pneumoconiosis: no, yes 0.2%, unknown.   Details of treatments received (treatment for diabetes, undernutrition and other concomitant conditions): When a patient was diagnosed with TB, treatment was arranged by local hospitals. | |
| Outcomes | **Tuberculosis (active tuberculosis or tuberculosis disease)**  Definition and method for measurement of outcome:  Active TB included bacteriologically positive TB and clinically diagnosed TB: a) A bacteriologically positive TB was defined as an individual with at least one sputum smear positive (= sputum specimen had at least one acid-fast bacillus identified within 100 fields under microscopy) or culture positive (= specimen had at least one colony of Mycobacterium tuberculosis complex being isolated by using Löwenstein-Jensen medium).  b) Clinically diagnosed TB was defined as three negative smears and CXR abnormalities consistent with active pulmonary TB and meeting at least one of the following criteria: clinical TB symptoms, strong positive purified protein derivative reaction, TB lesions confirmed by histopathological examination of extrapulmonary tissue, or excluding other pulmonary disease by diagnostic treatment or follow-up observations [19].  Method: Active case finding: providing TB symptom screening and chest X-ray during an interview (baseline and annual follow-up), with sputum smear microscopy and culture for those with positive symptoms or abnormal X-rays. Passive case finding: TB symptoms were interviewed among those who visited hospitals and village clinics during the study period. X-ray and sputum specimen collection were administered to those with suspected TB symptoms.  For those diagnosed as active TB cases, the information on TB occurrence and date were further identified (verified?) in TBIMS.  Was the same outcome definition (and measurement method) used in all patients?  215 cases → 62 bacteriologically positive cases (49 (79%) by active case finding, 13 (21%) by passive case finding). → 153 clinically positive cases (131 (86%) active case finding, 22 (14%) passive case finding)   Type of outcome: (e.g., single or combined endpoints): incident active TB: single outcome   Time of outcome occurrence or summary of duration of follow-up:  Participants were followed from baseline until the outcome of incident active TB, death, transfer or moving out from the study site, completion of follow-up or the the end (2 years after baseline) of the study (in Sept 2015).  **Recurrent TB**  Not measured | |
| Risk factor: undernutrition | Definition and method for measurement: BMI as underweight (BMI < 18.5 kg/m2), normal weight (≥ 18.5 kg/m2 and < 24.0 kg/m2), overweight and obese (≥ 24.0 kg/m2) .  Timing of measurement: at baseline (and during follow-up interviews).  Handling of predictors in the modeling: categorized: < 18.5 kg/m2, ≥ 18.5 kg/m2 and < 24.0 kg/m2, and ≥ 24.0 kg/m2 | |
| Sample size, missing data and analysis | Number of participants, number of outcomes/events and amount of accumulated person-years (PY): 34,076 participants at baseline (n=22,119 after two years of follow-up); 215 incident active TB and 62 bacteriologically confirmed in 44,622.2 person-years.  Number of participants, person-years and outcomes/events in relation to the number of candidate predictors (Events Per Variable): 337 active TB cases in 4789.2 PY for undernutrition. Nine active TB cases in 2968.9 PY for diabetes. 17 bacteriologically confirmed cases in 4789.2 PY for undernutrition. 0 bacteriologically confirmed cases of diabetes.  Number of participants with missing values (including predictors and outcomes): 11,957 were lost-to-follow-up and excluded from the analyses. Table 1 reports no unknown BMI data for 7 participants (4 for those with complete follow-up). Some other variables had participants with “unknown” info, too (alcohol, smoking, and others).  Number of participants with missing data for each predictor:  There were differences for those lost-to-follow-up and those with two years of follow-up (the main reason for the high lost-to-follow-up (35%) was that participants were healthy elderly without TB, resulting in a high proportion (about 30%) refusing the follow-up check). Seven people had unknown BMI info (4 amongst those with two years of follow-up)  Handling of missing data: NR.  Modelling method:  Pearson’s χ2 test or Fisher’s exact test was used to test for differences in proportions. The TB incidence rates for bacteriologically positive and active TB were calculated and expressed as cases per 100,000 PY. Using a forward inclusion approach, a univariate Cox proportional regression model was performed with a time-dependent covariate in relation to TB incidence. Multivariate Cox regression: variables significantly associated with the univariate model and of epidemiological interest were included—analyses for bacteriologically positive and active TB (all TB). Based on the results from multivariate analysis, they calculated the population attributable fraction (PAF) of each risk factor contributing to incident TB. Data analysis was performed using SPSS software.  Assumptions:  Not reported.  Adjustment factors used:  Active TB (n=215): age, sex, nationality, annual average income per person in family, previously treated with TB, smoking, alcohol, BMI  Bacteriologically positive TB (n= 62): age, sex, smoking, BMI. | |
| Results | **Interpretation**  BMI <18.5 was a strong predictor for developing bacterially positive TB. For those with a BMI ≥24.0, the risk was substantially reduced.  **Comparison with other studies**  Our study identified BMI < 18.5 [reference 32] as one of the main risk factors for elderly TB, being very similar to other studies globally [References 30,31,32, 33,34,35].  **Discussion of generalizability**  Not mentioned  **Strengths and limitations**  *Strengths*   - Prospective design - Population-based sampling - Quality control of measures.   *Limitations*   - Lost to follow up - Only 2-year follow-up | |
| Funding | This study was supported by The National Twelfth Five-year Mega-Scientific Projects of Infectious Diseases of China (grant No.: 2013ZX10003004–001), and the funder had no contribution to study design, data collection and analysis, result interpretation and paper writing. | |
| Conflict of interests | The authors declare that they have no competing interests. | |
| Notes | 13 February 2024 - We contacted Dr Zhang (zhanghui@chinacdc.cn) to ask for clarification about missing data, outcome data and sensitivity analses. | |
| ***Risk of bias*** | | |
| **Bias** | **Authors' judgement** | **Support for judgement** |
| Study participation | Low risk | Population-based sampling, adequately described characteristics. |
| Study Attrition | High risk | High attrition (35%), with higher income in lost-to-follow-up |
| Prognostic Factor Measurement | Low risk | Adequate definition and measuremntes, with minimal missing data |
| Outcome Measurement | High risk | Most participants had non-bacteriologically confirmed TB. |
| Study Confounding | Unclear risk | Immunosupresion was not included in the adjustment nor measured. |
| Statistical Analysis and Reporting | Unclear risk | Desription of statistical model and selection of confounders specified; no subgroup or sensititivity analyses undertaken |

Cho 2022

| ***Study characteristics*** | | |
| Study details and sources | **Study design:** population-based cohort study  **Source of data:** Korean National Health Insurance Database  **Study dates**:2010-2017  **Setting:** Outpatient  **Countries:** the Republic of Korea | |
| Participants | **Participant eligibility:**  ≥20 years of age, available data values of BMI, no diagnosis of TB before study enrollment, nor developed TB within one year from study enrollment.  **Recruitment method:**  Individuals who participated in the 2010 National Health Screening Program. Biennially national health screening program for employees of all ages and those older than 40.  **Participant description:**   - Age: mean age ranged from 40.8 to 49.1 - Sex: proportion of males ranged from 30.7 to 63.5%   **Risk factors**   - diabetes (fasting blood glucose ≥ 126 mg/dL) - undernutrition: BMI - HIV infection: not reported - Recent history of TB: not reported (patients were excluded) - History of untreated or inadequately treated TB disease immunosuppressive therapy: not reported - cigarette smokers (never, ex-smoker, current smoker) - drug or alcohol use disorders (non-drinker, mild drinker (<30 g/day of alcohol), and heavy drinker (≥30 g/day)) - socioeconomic status: individuals whose health insurance premiums were less than the lowest quintile in the insured or who were medical aid beneficiaries. - other   - physical activities (vigorous activity (≥ 20 min/day) three or more times a week), (moderate activity (≥ 30 min/day) five or more times a week   - dyslipidemia (total cholesterol ≥240 mg/dL)   - hypertension (systolic pressure ≥140 mmHg; diastolic pressure ≥ 90 mmHg)   Details of treatments received (treatment for diabetes, undernutrition and other concomitant conditions): not described | |
| Outcomes | **Tuberculosis (active tuberculosis or tuberculosis disease)**  Definition and method for measurement of outcome: Disease-specific confirmation results and doctor´s diagnoses registered in the code system (V246, V206 and V101 until June 2016; V000 thereafter (Corresponding to the International Classification of Disease, 10th revision codes (ICD-10)).  Was the same outcome definition (and method for measurement) used in all patients? Yes  Type of outcome: incident TB.  Time of outcome occurrence or summary of duration of follow-up: All participants were followed up from the date of the national health screening in 2010 to the date of TB diagnosis or December 31, 2017, whichever came first.  **Recurrent TB**  Not measured | |
| Risk factor: undernutrition | Definition and method for measurement: WHO Western Pacific Region guideline BMI (kg/m2) strata of underweight (<18.5), normal weight (18.5-22.9), overweight (23.0-24.9), and obesity (≥ 25.0). The underweight population was further categorised into mild (17.0-18.4), moderate (16.0-16.9), and severe (<16.0) thinness.  Timing of measurement: at enrollment.  Handling of predictors in the modelling: Categorical with the range of the BMI; reference category: normal BMI (18.5-22.9). | |
| Sample size, missing data and analysis | Number of participants, number of outcomes/events and amount of accumulated person-years (PY):  Overall: 11,135,332 participants; 70,063,154.3 PY; 52,615 cases of incident TB  Number of participants, person-years and outcomes/events in relation to the number of candidate predictors (Events Per Variable)   - BMI <18.5: 429,604 participants; 2,657,961.15 PY; 4549 cases of incident TB - Mild thinness (17.0≤ BMI <18.5): 349,652 participants; 2,173,728.20 PY; 3406 cases of inc TB - Moderate thinness (16.0≤ BMI <17.0): 61,445 participants; 376,382.18 PY; 786 cases of inc TB - Severe thinness (BMI <16.0): 18,507 participants; 107,850.76 PY; 357 cases of inc TB   Number of participants with any missing value (include predictors and outcomes): participants with missing data on BMI (n=275,894, 2.4%) were excluded after enrollment. No further info on potentially missing variables  Number of participants with missing data for each predictor: 275,894 (2.4%) participants with missing BMI were excluded after enrollment  Handling of missing data (e.g., complete-case analysis, imputation, or other methods): not mentioned  Modelling method:   - TB incidence rates per 1000 PY - Cox regression analyses stratified by BMI categories (reference = normal weight). Adjustment for: age, sex, smoking (never, ex, current), alcohol (non-drinker, mild, heavy drinker), regular exercise (y/n), low-income status (y/n), hypertension (y/n), diabetes (y/n), dyslipidemia (y/n). - Stratified analyses by age, sex, smoking, alcohol, regular exercise, low income, diabetes, hypertension, and dyslipidemia were performed by dividing the participants into subgroups (risk factor groups) using SAS and STATA software. - statistical significance was defined as 2-sided *p<0.05.*   Assumptions: not reported.  Adjustment factors used: age, sex, smoking (never, ex, current), alcohol (non-drinker, mild, heavy drinker), regular exercise (y/n), low-income status (y/n), hypertension (y/n), diabetes (y/n), dyslipidemia (y/n) | |
| Results | **Interpretation:**  There is a significant inverse relationship between BMI and TB incidence, which was especially profound in the underweight population.  HR for incidence of TB and BMI relative to normal weight population:   - BMI < 18.5 (underweight) = 2.08 (95% CI 2.02-2.15) - BMI > 23 (overweight) = 0.56 (95% CI 0.55-0.58) - BMI > 23 (obese) = 0.40 (95% CI 0.39-0.41)   Among the underweight population, TB risk increased with the degree of thinness increased, relative to normal weight   - mild: HR = 1.98 (1.91-2.05) - moderate: HR = 2.50 (2.33-2.68) - severe: HR = 2.83 (2.55-3.15)   **Comparison with other studies**  Reference 36: a small improvement in nutrition could substantially impact the incidence of TB in areas with a high prevalence of undernutrition.  **Discussion of generalizability**  Since the study is conducted with a population of a high-income country in Asia, it might not be generalised to situations in other countries or ethnicities with different environments.  **Strengths and limitations**  Limitations:   - The specific code for TB used included all types of TB, so a stratified analysis according to the type of TB was not possible. - Not only is nutritional status related to being underweight - but other factors (low birth weight, respiratory infection in childhood, etc.) were not available to evaluate whether BMI was a TB risk factor independent of these vars.   Strengths:   - Since environmental factors affecting BMI and TB are lower in high-income countries, the study could have measured the impact of being underweight on the risk of TB more accurately. - Representative nationwide data in a single country. - Findings are extended to the underweight population, dose-response effect. | |
| Funding | The authors reported no funding sources. | |
| Conflict of interests | The authors declare no competing interests. | |
| Notes | 28 August 2023 Email: belong@snu.ac.kr 1) How many cases of TB were confirmed by culture or rapid molecular tests? Another paper says that in South Korea, "Among the pulmonary TB cases, 8812 (35.7%) were smear-positive pulmonary cases."; but we need to estimate whether the majority had a culture (or microbiological confirmation) of cases. But we are unsure if this adequately represents your study's population (i.e., most are not microbiologically-confirmed). 2) Why wasn't immunosuppression (i.e. HIV and other forms) accounted as confounders? Could you point us to another report with this variable accounted for in the analysis? | |
| ***Risk of bias*** | | |
| **Bias** | **Authors' judgement** | **Support for judgement** |
| Study participation | Low risk | Representative population-based database from South Korea in 2010 (no sampling) with partial description of baseline characteristics. |
| Study Attrition | Low risk | Minimal attrition: 2% (275894 participants) were excluded due to missing BMI data. |
| Prognostic Factor Measurement | Low risk | Valid cut-off point (18.5 for BMI) measured uniformly across participants. |
| Outcome Measurement | Unclear risk | Same for all participants but unclear how many were culture-confimed. |
| Study Confounding | Unclear risk | Immunosupresion was not measured and missing data from covariates are not described; analysis was adequate for the measured confounders. |
| Statistical Analysis and Reporting | Low risk | Adequate analysis strategy (survival analysis) accounting for important covariates |

Choi 2021

| ***Study characteristics*** | | |
| Study details and sources | **Study design:** retrospective cohort  **Source of data:** Korean National Health Insurance Service (NHIS) database through health screenings and self-questionnaires.  **Study dates:** 2009 - December 31, 2018  **Setting:** outpatient  **Countries:** South Korea | |
| Participants | **Participant eligibility**  *Exclusion*: participants aged <20 years, those previously diagnosed with TB before enrollment, and those with missing data.  Recruitment method: participation in the health screening exams between January 1, 2009 and December 31, 2009.  **Participant Description:**   - Age: > 20 years (subgroup of <65, and >65 years) - Sex: 5,555,303 Male / 10,087,903   **Risk factors:**   - diabetes: ICD-10 diagnosis codes E11-E1 and fasting blood glucose. - undernutrition: BMI (underweight (<18.5 kg/m2), normal weight (18.5–22.9 kg/m2), overweight (23.0–24.9 kg/m2), obese (25.0–29.9 kg/m2), and severely obese (≥30 kg/m2)). - HIV infection: not mentioned - recent TB infection: excluded from the study - history of untreated or inadequately treated TB disease immunosuppressive therapy - cigarette smokers: never, former, and current smoker - drug or alcohol use disorders: heavy drinker defined as over 30 g of alcohol per day - socioeconomic status: income in the lowest 20% (n, %) - other (specify)   - Physical activity: strenuous exercise for at least 20 minutes three times per week or moderate exercise for at least 30 minutes five times weekly.   - Hypertension: ICD-10 diagnosis codes I10–I15   - Dyslipidemia: ICD-10 diagnosis codes E78   Details of treatments received (treatment for diabetes, undernutrition and other concomitant conditions): not mentioned | |
| Outcomes | **Tuberculosis (active tuberculosis or tuberculosis disease)**  Definition and method for measurement of outcome: defined using the rare intractable disease (RID) registration codes for TB (V206, V246, and V000).  Was the same outcome definition (and method for measurement) used in all patients? Yes  Type of outcome: incidence of TB  Time of outcome occurrence or summary of duration of follow-up: Follow up to the date of TB incidence, death, or until December 31st, 201, whichever came first, totalling 7,3 year follow up.  **Recurrence of Tuberculosis**  Not measured. | |
| Risk factor: undernutrition | Definition and method for measurement: BMI was calculated as body weight (kg) divided by height (m) squared and was classified into five categories—underweight (<18.5 kg/m2), normal weight (18.5–22.9 kg/m2), overweight (23.0–24.9 kg/m2), obese (25.0–29.9 kg/m2), and severely obese (≥30 kg/m2)—according to the Asia-Pacific BMI criteria established by the Western Pacific Region of the World Health Organization.  Timing of measurement: at the time of enrollment.  Handling of predictors in the modelling: Categorized according to BMI status. Subgroup analysis with stratified categories according to age, sex, smoking status, and alcohol consumption. | |
| Sample size, missing data and analysis | Number of participants, number of outcomes/events and amount of accumulated person-years (PY): Total number of participants: 10,087,903. During the 7.3-year follow-up duration, the incidence of TB was 0.92 per 1,000 person-years in the normal weight without DM, 2.26 in the normal weight with DM, 1.80 in the underweight without DM, and 5.35 in the underweight with DM.  Number of participants, person-years and outcomes/events in relation to the number of candidate predictors (Events Per Variable):  *Without DM*: Compared with the normal weight, the underweight showed an increased risk of incident TB (aHR, 2.21; 95% CI, 2.14–2.28); however, overweight (aHR, 0.54; 95% CI, 0.53–0.55), obese (aHR, 0.36; 95% CI, 0.35–0.37), and severely obese (aHR, 0.28; 95% CI, 0.26–0.31) participants showed decreased risks of incident TB.  *With DM*: participants with DM, compared with the normal weight,  the underweight showed an increased risk of incident TB [adjusted hazard ratio (aHR), 2.13; 95% confidence interval (CI), 1.93–2.35]; however, overweight (aHR, 0.58; 95% CI, 0.55–0.61), obese (aHR, 0.44; 95% CI, 0.41–0.46), and severely obese (aHR, 0.29; 95% CI, 0.25–0.33) participants showed decreased risks of incident TB.  Number of participants with any missing value (include predictors and outcomes): Participants with missing data were excluded at enrollment.  Number of participants with missing data for each predictor: not mentioned  Handling of missing data (e.g., complete-case analysis, imputation, or other methods): exclusion of missing data.  Modelling method:   - The incidence rate of TB was calculated by dividing the number of incident cases by the total follow-up duration (1,000 person-years). - Cox proportional hazards regression analyses were used to evaluate the association of BMI with the incidence of TB, without considering the time-varying nature of BMI. - Statistical analyses were performed using SAS version 9.4 - All tests were two-sided, and P-values < 0.05 were considered statistically significant.   Assumptions: not mentioned.  Adjustment factors used: adjusted for age, sex, smoking status, alcohol consumption (heavy drinker or not), regular physical activity, income (lowest 20% or not), hypertension, and dyslipidemia. | |
| Results | **Interpretation**  During the 7.3-year follow-up duration, the incidence of TB was:   - 0.92 per 1,000 person-years in the normal weight without DM, - 2.26 in the normal weight with DM, - 1.80 in the underweight without DM, and - 5.35 in the underweight with DM.   Compared to the normal weight without DM, the normal weight with DM, the underweight without DM, and the underweight with DM showed a 1.51-fold (95% CI, 1.46–1.57), a 2.21-fold (95% CI, 2.14–2.28), and a 3.24-fold (95% CI, 2.95–3.56) increased risk of TB, respectively. However, compared to the normal weight without DM, the severely obese without DM and those with DM showed a 0.37 (95% CI, 0.36-0.38) and a 0.42 (95% CI, 0.36-0.48)-fold decreased risk of TB, respectively.  There was no significant joint effect of BMI and DM on the risk of incident TB in the overall population.  In conclusion, being underweight or DM individually increases the risk of incident TB.  *Stratified by sex:* Compared with the normal weight without DM, the male and female underweight with DM showed 3.69-fold (95% CI, 3.31–4.11) and 2.39-fold (95% CI, 1.98–2.89) increased risks of TB, respectively.  *Stratified by age*: the underweight with DM aged < 65 years and those aged ≥ 65 years showed 4.99-fold (95% CI, 4.40– 5.67) and 2.17-fold (95% CI, 1.89–2.49) increased risks of TB compared with the normal weight without DM aged < 65 and ≥ 65 years, respectively.  **Comparison with other studies**  (Reference 4) Being underweight predisposes patients to the development of TB.  (Reference 5) The inverse association between BMI and TB presents a paradox concerning diabetes mellitus (DM).  (References 6, 7) Obesity is a major determinant of DM, and DM is a well-known risk factor for TB (References 3, 8, 9).  (References 5, 10) Two Asian studies examined the joint effect of DM and BMI on incident TB.  (References 4, 23, 24), this study also showed that overweight and obese individuals had a significantly lower risk of TB than normal-weight individuals.  Discussion of generalizability: Since Korea is an intermediate TB burden country, it might not be generalizable to other countries-  **Strengths and limitations**  *Strengths: The* major strength of this study was to assess the joint effects of BMI and DM on the risk of incident TB, using a nationwide population-based longitudinal cohort with exact measurements of exposures (DM and BMI)  *Limitations*: two major limitations: (1) subjects with obesity, not those with normal weight, were the reference group for the analysis, and (2)  the overall study population was small, which limited statistical  power and further stratified analyses.  Other limitations include:   1. The diagnosis of DM and comorbidities was based on ICD-10 codes and medications. Thus, there might be potential errors in diagnosing diseases. 2. This study was performed in Korea, a country with an intermediate TB burden. Therefore, our findings might not be generalizable to patients in other countries. 3. Since we assessed the BMI of each participant upon enrollment in this study, the impact of temporal changes in BMI during the follow-up period on TB risk could not be assessed in this study. 4. This study could not analyze the detailed clinical courses of subjects with DM potentially affecting BMI change and TB risk, which include glucose control trajectories, types of DM, and classifications of DM medications.   Also, there is no differentiation between Type 1 and 2 DM. | |
| Funding | Basic Science Research Program supported this research through the National Research Foundation of Korea (NRF) funded by the Ministry of Science, Information, and Communications Technologies (Grant Nos. 2020R1F1A1070468 and 2021M3E5D1A01015176 to HL and 2019R1G1A1008692 to HC) and the Korean Ministry of Education (Grant No. 2021R1I1A3052416 to HC). | |
| Conflict of interests | The authors declare no conflicts of interest. | |
| Notes | Email to dwshin.md@gmail.com asking about culture for confirmation of TB diagnosis. | |
| ***Risk of bias*** | | |
| **Bias** | **Authors' judgement** | **Support for judgement** |
| Study participation | Low risk | Large national database. |
| Study Attrition | Low risk | Minimal attrition: 3.4% (361280 participants) were excluded due to missing data. |
| Prognostic Factor Measurement | Low risk | PF was diagnosed by clinicians for all participants according to the standard criteria and attritions were minimal. |
| Outcome Measurement | Unclear risk | Using Korean rare intractable disease registration coding for data collection, unclear what proportion had culture. |
| Study Confounding | Unclear risk | All except HIV, previous TB, immunosuppressive therapy, drug use. |
| Statistical Analysis and Reporting | Low risk | Adequately presented Cox regression analysis for the defined model. |

Choun 2013

| ***Study characteristics*** | | |
| Study details and sources | **Study design:** Retrospective cohort study  **Source of data:** clinical assessment and interviews  **Study dates:** March 2003 to December 2010  **Setting:** Outpatient  **Countries:** Cambodia | |
| Participants | **Participant eligibility:**  All adult (>18 years) patients enrolling in the HIV care program and initiating ART at Sihanouk Hospital Center of Hope (SHCH) were included.  Recruitment method:  Patient sample enrolled in the HIV care program at Sihanouk Hospital Center of Hope (SHCH), Phnom Penh. Since March 2003, comprehensive HIV care has been provided at no cost as part of the national program.  **Participant Description:**   - Age: median age 34 years (IQR 29-40) - Sex: Female (53%), Male (47%)   **Risk factors:**   - diabetes: NR - undernutrition: BMI (<18.5 kg/m2 (underweight), 18.5–25 kg/m2 (normal weight) and >25 kg/m2 (overweight/obese)). - HIV infection: all patients with WHO stage IV disease, WHO stage III disease with CD4 cell count <350 cells/ul, or with CD4 cell count <200 cells/ul were eligible for ART - Recent TB infection: 31.2% had TB at enrollment (and were considered “incident TB” once TB treatment was finished). - history of untreated or inadequately treated TB disease immunosuppressive therapy: NR - cigarette smokers: NR - drug or alcohol use disorders: NR - socioeconomic status: NR - other (specify)   - Baseline hemoglobin (<8 g/dl, 8-10 g/dl, >10 g/dl)   Details of treatments received (treatment for diabetes, undernutrition and other concomitant conditions): ART: First-line treatment was a generic fixed-dose combination of stavudine, lamivudine and nevirapine. For patients with TB, nevirapine was replaced by efavirenz. | |
| Outcomes | **Tuberculosis (active tuberculosis or tuberculosis disease)**  Definition and method for measurement of outcome:   - TB diagnosis was based on microscopic examination of samples (sputum, fine-needle aspirates of lymph nodes) for acid-fast bacilli, or radiological investigations (chest radiography, abdominal ultrasound), or both. From 2009, culture on Löwenstein – Jensen medium was performed on all sputum samples (one sample per patient). - TB was diagnosed according to WHO criteria for smear-positive pulmonary TB, smear-negative pulmonary TB or extra-pulmonary TB. - *Prevalent TB*: patients who were already on TB treatment at the initiation of ART - *Incident TB*: patients diagnosed with TB after ART initiation   - *early incident TB*: defined as TB occurring within the first 6 months of ART.   - *late incident TB*: defined as TB occurring after > 6 months of ART.   Was the same outcome definition (and method for measurement) used in all patients? Same methods for all patients used, but outcome defined as either early incident TB or late incident TB.  Type of outcome: single endpoints.  Time of outcome occurrence or summary of duration of follow-up:  The status of each patient was recorded at the date of TB diagnosis, at the last visit for patients that died, who were transferred out or were lost to follow-up, and at 31 December 2010. Total of 8 years of follow up; the median follow-up period after ART initiation was 2.4 years (IQR 0.9–4.4).  **Recurrent TB**  Not measured | |
| Risk factor: undernutrition | Definition and method for measurement: BMI was categorized as <18.5 kg m2 (underweight), 18.5–25 kg m2 (normal weight) and >25 kg m2 (overweight/obese).   - In secondary analysis, a cut-off of 23 kg m2 instead of 25 kg m2 was used.   The BMI data was clinically assessed.  Timing of measurement: at baseline, and at every clinical visit (every 2-3 months for stable patients)  Handling of predictors in the modeling: Categorical for BMI | |
| Sample size, missing data and analysis | Number of participants, number of outcomes/events and amount of accumulated person-years (PY): 2,984 participants were included; 313/2052 incident TB, and 78/932 prevalent TB, had TB from a total of 2984 patients, and a total follow-up time of 8,059 person-years.  Number of participants, person-years and outcomes/events in relation to the number of candidate predictors (Events Per Variable):   - Early TB incidence: 179/2984 (6.0%) - 13.5 per 100 py.   - Being underweight (BMI <18.5 kg/m2 ) was associated with a 60% increase in risk (aHR 1.6; 95% CI 1.1–2.2). - Late incident TB 134/2984 (4.5%) - 2.0 per 100 py.   - Individuals being underweight while on ART had a significantly increased risk of developing late TB (aHR 2.4; 95% CI 1.1 –5.0)   Number of participants with any missing value (including predictors and outcomes): Not accurately reported.  Number of participants with missing data for each predictor: 72 CD4 cell count; 54 BMI, 28 hemoglobin  Handling of missing data: not reported.  Modelling method   - For initial descriptive analysis, TB incidence rates and 95% CI were calculated for different time periods after ART initiation. - They used Kaplan –Meier methods to calculate the cumulative incidence of ART-associated TB. - A risk factor analysis was performed using multivariate Cox regression - They conducted separate analyses for early and late incident TB.   - Early: only baseline factors were included   - late: time-updated (‘on-treatment’) CD4 cell counts, haemoglobin and BMI measurements were used. - Data were analyzed using STATA V.11 statistical software (StataCorp, College Station, TX, USA). - The level of significance was set at p < 0.05.   Assumptions: none reported.  Adjustment factors used: age, sex, CD4 cell count, hemoglobin, history of TB treatment prior to ART initiation and prevalent TB. | |
| Results | **Interpretation**  Risk factors for early TB included low body mass index, low baseline CD4 count and low haemoglobin levels.  Low on-treatment CD4 counts and haemoglobin levels, being underweight while on ART and prevalent TB were identified as risk factors for late TB.  **Comparison with other studies**   - (References 26, 29 - 33) Our observation is consistent with a growing number of large and carefully conducted studies, showing an inverse relationship between BMI and risk of development of incident TB, both in the general population and in cohorts of HIV patients.   **Discussion of generalizability**  Not reported.  **Strengths and limitations**  *Strengths:* not reported.  *Limitations*:   - "We conducted a retrospective analysis using routine data from a program setting. However, data were collected prospectively with regular quality checks performed, and our findings more likely reflect reality. " - "Although WHO recommendations were followed for TB diagnosis, bacteriological confirmation was often not obtained." - "Moreover, WHO recommendations have evolved over the years; asymptomatic TB infection would not have been detected, and some of the early TB incident cases might have been undiagnosed prevalent TB cases. " - "Moreover, we were not able to include reliable information on the occurrence of the immune reconstitution inflammatory syndrome, which would be particularly relevant with regard to early incident TB." - "The lack of TB information on those who died or were lost to follow-up could have led to additional bias. " - "Data on viral load and treatment adherence were not systematically available. The occurrence of other co-morbidities and causes of death would have also been of interest." | |
| Funding | NL is supported by a grant from the Research Foundation Flanders. The HIV program was supported by the Belgian Directorate General of Development Cooperation through the framework agreement with the Institute of Tropical Medicine, Antwerp, the Global Fund to fight AIDS, Tuberculosis and Malaria, World Mate, and HOPE Worldwide. JvG is supported by the InBev-Baillet Latour Fund, Belgium. | |
| Conflict of interests | None declared. | |
| Notes | Contacted Dr Choun (kimchengchoun@sihosp.org) about outcome assessment and adjustments. | |
| ***Risk of bias*** | | |
| **Bias** | **Authors' judgement** | **Support for judgement** |
| Study participation | Unclear risk | Focused on HIV patients on ART from one hospital. |
| Study Attrition | Low risk | Minimal attrition: 2.4%. |
| Prognostic Factor Measurement | Low risk | Same clinical standard for all participants, minimal attrition. |
| Outcome Measurement | Unclear risk | Same for all participants but unclear how many were culture-confimed. |
| Study Confounding | Unclear risk | All except DM, history of TB, HIV, immunosuppressive therapy. |
| Statistical Analysis and Reporting | Low risk | Adequately presented Cox regression analysis for the defined model. |

Dembélé 2010

| ***Study characteristics*** | | |
| Study details and sources | **Study design:** retrospective cohort  **Source of data:** clinical health records  **Study dates:** November 1998 - September 2005  **Setting:** Four HIV treatment centres  **Countries:** Burkina Faso | |
| Participants | **Participant eligibility**  All consecutive HIV-positive individuals aged 15 or more recorded in HAART registers, with a minimum follow-up time of 12 months.  **Recruitment method**  Consecutive participants.  **Participant Description**   - Age: 36.1 (mean), 7.9 (SD) - Sex: 70% Female   **Risk factors**   - diabetes: not reported - undernutrition: BMI <18.5 36.1% - HIV infection: all participants - recent TB infection: previous infection 4.1% - history of untreated or inadequately treated TB disease immunosuppressive therapy: not reported - cigarette smokers: not reported - drug or alcohol use disorders: not reported - socioeconomic status: not reported - other (specify)   - WHO Stage I 12%, II 33.3%, III 43.1%, IV 11.5%   - CD4 cell count >350 1.2%, 200-350 15.9%, 50-199 66.1%, <50 16.9%   Details of treatments received (treatment for diabetes, undernutrition and other concomitant conditions): HAART naive individuals. | |
| Outcomes | **Tuberculosis (active tuberculosis or tuberculosis disease)**  Definition and method for measurement of outcome: Smear microscopy for AFB or on algorithms (Chest X-ray, unresponsiveness to large-spectrum antibiotics, clinicians' decision). Extrapulmonary TB was based on the clinician's decision on the basis of signs and findings. Routine culture was not available.  Was the same outcome definition (and method for measurement) used in all patients? Yes.  Type of outcome: single (pulmonary and extrapulmonary were reported independently)  Time of outcome occurrence or summary of duration of follow-up: mean 836 days (SD 443.4)  **Recurrent TB**  Not measured. | |
| Risk factor: undernutrition | Timing of measurement: baseline but only for 1284 participants.  Handling of predictors in the modelling: category of undernutrition BMI <18.5 vs BMI >=18.5 | |
| Sample size, missing data and analysis | Number of participants, number of outcomes/events and amount of accumulated person-years (PY): 2,383 participants, 1,284 with BMI data.  Number of participants, person-years and outcomes/events in relation to the number of candidate predictors (Events Per Variable) 70 cases of TB, 43 pulmonary and 37 extrapulmonary TB. 29 in BMI <18.5 and 18 in BMI>=18.5 (BMI was available for a subset of participants)  Number of participants with any missing value (include predictors and outcomes) 1099 with missing BMI data  Number of participants with missing data for each predictor: 1099 with missing BMI data  Handling of missing data: complete case analysis  Modelling method: Cox proportional hazards  Assumptions: None defined  Adjustment factors used: sex, age and variables with significant associations in univariate analysis | |
| Results | **Interpretation**  “Risk factors that were significantly associated with increased TB risk were CD4+ T-cell count <50 and BMI <18.5”  **Comparison with other studies**  Both factors had been identified in previous studies (refs. 5 and 14).  **Discussion of generalizability**  The nature of the cohort (HAART-naive people living with HIV)  **Strengths and limitations**  Over and under-reporting due to the lack of culture confirmation, especially for extrapulmonary forms of TB. TB incidence in the first trimester could be overestimated (prevalent TB unmasked by HAART). | |
| Funding | Global Fund, Grant BUT 404-T | |
| Conflict of interests | Not available | |
| Notes | No contact with the author (high risk of bias). | |
| ***Risk of bias*** | | |
| **Bias** | **Authors' judgement** | **Support for judgement** |
| Study participation | Unclear risk | Consecutive recruitment of people living with HIV from a program, partial description of baseline characteristics. |
| Study Attrition | High risk | 47% attrition due to missing BMI (no outcome data for people with missing BMI). |
| Prognostic Factor Measurement | High risk | Adequate assessment of risk factor but 47% with missing BMI. |
| Outcome Measurement | High risk | No microbiological confirmation in 58% of pulmonary TB cases. |
| Study Confounding | Unclear risk | Diabetes, smoking, alcohol and socioeconomical status not measured. |
| Statistical Analysis and Reporting | Low risk | Adequate analysis strategy (survival analysis) accounting for important covariates. |

Ganesan 2023

| ***Study characteristics*** | | |
| Study details and sources | **Study design:** prospective cohort study  **Source of data:** clinical assessment  **Study dates:** 2013 to 2021  **Setting:** outpatient  **Countries:** Uganda, Kenya, Tanzania, and Nigeria. | |
| Participants | **Participant eligibility**  Inclusion criteria: Individuals living with HIV aged 15 years or older and consenting to data and specimen collection.  Exclusion criteria: having a significant condition including medical and psychological/psychiatric disorders, being pregnant or currently being a prisoner (screen failures not captured).  **Recruitment method**  Most HIV-infected study participants were invited to the study based on random selection from existing clinic patient lists (stratified by gender and ART status). Less than 5% were recruited from other HIV studies.  **Participant Description**   - Age: median age 37.4 xears (IQR 29.3-45.5); 17% 15-24, 42% 25-39, 26% 40-49, 15% >50 years - Sex: 41% Male, 59% Female   **Risk factors:**   - diabetes: not measured (9.3% hyperglycemia) - undernutrition: 11.7% BMI <18.5 kg/m² - HIV infection: all participants - recent TB infection: 6.7% had a history of previous pulmonary TB infection; 2.9% had active TB at enrollment - history of untreated or inadequately treated TB disease immunosuppressive therapy: not measured - cigarette smokers: 4.5% - drug or alcohol use disorders: 18.5% (consume alcohol) - socioeconomic status:   - Education: 32.0% no or some primary education; 40.4 primary of some secondary education; 27.6% secodary education and higher - other:   - Ever incarcerated 10.2%   - 52.5% married, 32% none or some primary education, 62.5% unemployed, 31..7% <3 people in household   Details of treatments received: 28.4% were naive to antiretroviral therapy. | |
| Outcomes | **Tuberculosis (active tuberculosis or tuberculosis disease)**  Definition and method for measurement of outcome: (1) bacteriologically confirmed through smear microscopy, culture, or WHO-approved rapid diagnostics (including GeneXpert MTB/RIF), (2) clinically indicated and having initiated combination therapy for active TB in the absence of bacteriological confirmation, or (3) identified by medical record abstraction within three months of enrollment  Was the same outcome definition (and method for measurement) used in all patients? No  Type of outcome: single  Time of outcome occurrence or summary of duration of follow-up: Participants were censored at competing events (either loss to follow-up or death). Time at risk of TB started at entry into the cohort, and failure or censor date was observed over the duration of the study at follow-up visits. Median observation time per person was 2.98 years (Interquartile range: 1.51–4.50)  **Recurrent TB**  Not measured | |
| Risk factor: undernutrition | Definition: BMI <18.5 kg/m²  Method of measurement: Calculated using participants’ height and weight and categorised as underweight (<18.5), normal (18.5–24.9), or overweight/obese (≥25)  Timing of measurement: at recruitment and subsequents visits every 6 months.  Handling of predictors in the modelling (e.g., continuous, linear, non-linear transformations or categorised): as a time-varying variable, categorised as normal weight, underweight and overweight. | |
| Sample size, missing data and analysis | Number of participants, number of outcomes/events and amount of accumulated person-years (PY): 3,171 participants, 93 with prevalent TB, 79 cases of incident TB in 13,161 PY follow-up  Number of participants, person-years and outcomes/events in relation to the number of candidate predictors (Events Per Variable): Not available  Number of participants with any missing value (include predictors and outcomes): 171 participants  Number of participants with missing data for each predictor: 7 participants for BMI, 49 for hyperglycaemia, 3 for smoking, 2 for alcohol consumption, 2 for education, 1 age. Not available for outcome.  Handling of missing data (e.g., complete-case analysis, imputation, or other methods): Missing data were folded into the reference category for modelling and also Complete Case Analysis.  Modelling method  Incidence rates (IRs) were calculated for participants living with HIV without TB at or within three months of enrollment, as the number of new TB diagnoses divided by person-years (PY) of follow-up. CIs were calculated using the quadratic approximation to the Poisson log-likelihood for the log–rate parameter. Cox proportional hazard models were used to assess unadjusted and adjusted hazard ratios and 95% CIs for associations between time to-incident TB and clinical and socio-behavioural predictor variables. Time-varying covariates were accounted for in the model. All variables that were associated with prevalent or incident TB (P < 0.2) in the bivariate analysis were included in the respective multivariable analysis in addition to the following variables identified a priori: age, sex, and clinical site.  Assumptions: NR  Adjustment factors used: age, sex, clinical site, time since HIV diagnosis, ART regimen, CD4 cell count, viral load. | |
| Results | **Interpretation**  PLWH in our cohort who were underweight had a higher risk of incident TB and odds of prevalent TB compared with those with a normal BMI.  **Comparison with other studies**  These findings are well established in the literature and consistent with other studies from low- and middle-income countries (LMIC) that found a bidirectional association between low BMI and TB. (References in the report 35–40)  **Discussion of generalizability**  Participants in the study have better access to TB screening and diagnostic services, given rigorous study follow-up, and may thus not be representative of the general population of clients receiving care at PEPFAR facilities.  **Strengths and limitations**   - Selection bias may be present as volunteers in the study may differ from non-participants. - Participants in the study have better access to TB screening and diagnosis, which may not reflect the general population. - Active TB cases may have been misclassified, leading to inflated prevalence numbers. - Unmeasured confounders could affect the associations observed in the study. - The causality between exposure and outcome is difficult to establish due to the study's observational nature. - The effect of pre/post-treatment on TB incidence could not be assessed accurately. - Difficulty in categorising participants into pre/post Treat All due to enrollment at different times. - The analysis did not account for latent TB disease and isoniazid prophylaxis. | |
| Funding | Henry M. Jackson Foundation for the Advancement of Military Medicine, Inc., and the US Department of Defense. National Institute of Allergy and Infectious Diseases of the National Institutes of Health. | |
| Conflict of interests | The authors declare no conflict of interest. | |
| Notes | No queries to the authors. | |
| ***Risk of bias*** | | |
| **Bias** | **Authors' judgement** | **Support for judgement** |
| Study participation | Unclear risk | Cohort of adults living with HIV (program); mostly well-described at baseline except of the rate of participation and diabetes status. |
| Study Attrition | Low risk | Minimal attrition (5% with missing data). |
| Prognostic Factor Measurement | Low risk | Presumibly valid cut-offs for BMI, uniformly across participants, complete case analysis available and imputation |
| Outcome Measurement | Low risk | TB by bacteriology, indication of TB therapy or registered in medical record for all participants. |
| Study Confounding | Unclear risk | Only age, sex and variables related to HIV status; however the analysis was adequate for the selected confounders |
| Statistical Analysis and Reporting | Unclear risk | Desription of statistical model and selection of confounders specified; no subgroup or sensititivity analyses undertaken |

Gatechompol 2022

| ***Study characteristics*** | | |
| Study details and sources | **Study design:** retrospective cohort  **Source of data:** Program/study-specific data collection  **Study dates**: 1996 to December 2020  **Setting:** Health centres  **Countries:** Thailand | |
| Participants | **Participant eligibility**  People living with HIV aged 18 years and above who initiated ART from 1996 to December 2020. Participants who initiated ART before enrolling into HIV-NAT 006 were included in the analysis if all relevant clinical information was available at the time of enrolment.  **Recruitment method**  Most cohort participants had previously participated in clinical trials, and after completion of the trials, they were automatically enrolled into the cohort to receive continuous care.  **Participant Description:**   - Age: median 32.0 (IQR 27.4–37.6) - Sex: 67.5% men   **Risk factors:**   - diabetes 5.3% - undernutrition: % not provided - HIV infection: all participants - recent TB infection: 5.3% - history of untreated or inadequately treated TB disease - immunosuppressive therapy: all with HIV infection - cigarette smokers: 20.2% - drug or alcohol use disorders: 14.6% - socioeconomic status: not available - other (specify)   - History of substance us: 6.6%   - Hypertension: 13.7%   - Hepatitis B co-infection: 13.9%   - Hepatitis C co-infection: 9.5%   Details of treatments received (treatment for diabetes, undernutrition and other concomitant conditions): *All patients on ART* | |
| Outcomes | **Tuberculosis (active tuberculosis or tuberculosis disease)**  Definition and method for measurement of outcome:  *A diagnosis of incident TB case was defined as (1) active TB bacteriologically confirmed by smear microscopy, culture or Xpert, or (2) active TB clinically diagnosed based on radiologic evidence of TB without bacteriological confirmation and with good clinical response to antituberculosis treatment.*  Was the same outcome definition (and method for measurement) used in all patients? Yes  Type of outcome: *incident TB.*  Time of outcome occurrence or summary of duration of follow-up: *Follow-up data up to June 2021 were analyzed so all of the participants had ≥ 6 months of follow-up.*  **TB recurrence (relapse or reinfection)**  *Not reported* | |
| Risk factor: undernutrition | Definition and method for measurement: *underweight (BMI l <18.5 kg/m²) compared to no normal weight (BMI 18.5-23 kg/m²). Not described how BMI (weight and height) was assessed.*  Timing of measurement: *collected at each clinic visit*  Handling of predictors in the modelling: *categorized; see above.* | |
| Sample size, missing data and analysis | Number of participants, number of outcomes/events and amount of accumulated person-years (PY): *total: 2,849; analyzed: 2,636 cases: 113; PY: 24,229*  Number of participants, person-years and outcomes/events in relation to the number of candidate predictors (Events Per Variable) *NA*  Number of participants with any missing value (include predictors and outcomes)*[231 excluded due to 1) Diagnosed with TB episode within the year prior to ART initiation =20, 2) Developed TB within 3 months after ART initiation (TB IRIS) =19, 3) Not started on ART = 102, 4) Incomplete data =72]*  Number of participants with missing data for each predictor: *not specified*  Handling of missing data (e.g., complete-case analysis, imputation, or other methods): *complete-case analysis*  Modelling method   - multivariate Cox regression models (competing risk: death) - Covariates significant in univariable analyses with p < 0.10 were adjusted for in multivariable analyses. - sensitivity analyses: the first was limited to cohort participants with bacteriologically confirmed TB, the second where time up-dated variables were modelled with a-12 month lag, to provide - more information on the temporal association of BMI and incident TB.   Assumption: not reported  Adjustment factors used:   - Time-fixed covariates were age, sex and CDC disease classification at ART initiation - Time-varying covariates were modelled with a 6-month lag, and included viral load (VL), CD4 count, smoking status, alcohol use, substance use, close contact with TB cases, other co-morbidities and BMI. | |
| Results | **Interpretation**  Low BMI < 18 kg/m² compared to BMI 18-23 kg/m2. (Normal weight) was associated with incident TB. Adjusted sub-distribution HR = 8.21, 95% CI 2.43–27.73, p = 0.001  In the sensitivity analysis where the temporal nature of the association of TB with BMI using a 12-month covariate lag was explored, the association with BMI < 18.5 was reduced (aSHR 2.58 (95% CI 1.12- 6.03).  **Comparison with other studies**  "Findings confirm that TB risk among PLWH remains high, even after long-term ART, and is consistent with those of a previous long-term ART cohort study conducted in Africa. Regarding risk factors, including low BMI, the findings are similar to those from previous studies in high TB burden settings, which have also been confirmed in animal studies."  **Discussion of generalizability**  Data were collected from a single HIV research clinic in Thailand, which might limit the generalizability of the results.  **Strengths and limitations.**  Limitations:   - retrospective design - Only 27% of incident TB cases had bacteriologically confirmed TB. It is possible that some of the participants who were diagnosed clinically had pathogens other than M. tuberculosis - since TB preventive therapy (TPT) was not evaluated in this study, the authors could not demonstrate the additional benefits of TPT in conjunction with ART.   Strengths:   - longest follow-up time in Southeast Asia (>25 years) - competing risks models | |
| Funding | This work was supported by HIV-NAT, Thai Red Cross AIDS Research Centre, Bangkok, Thailand and Center of Excellence in Tuberculosis, Faculty of Medicine, Chulalongkorn University, Bangkok, Thailand. | |
| Conflict of interests | The authors report no conflicts of interest in this work. | |
| Notes | No queries to the authors. | |
| ***Risk of bias*** | | |
| **Bias** | **Authors' judgement** | **Support for judgement** |
| Study participation | Unclear risk | Only about HIV patients undergoing ART treatments in health centres. |
| Study Attrition | Low risk | Minimal attrition (2.5% with missing data). |
| Prognostic Factor Measurement | Low risk | PF was diagnosed by clinicians for all participants according to the standard criteria. |
| Outcome Measurement | Unclear risk | Only a minority of 27% was bacteriologcally confirmed, and it was not further specified what proportion of diagnoses were culture-confirmed. |
| Study Confounding | Unclear risk | All important confounders except socioeconomic status. |
| Statistical Analysis and Reporting | Low risk | Adequately presented Cox regression analysis for the defined model. |

Gedfew 2020

| ***Study characteristics*** | | |
| Study details and sources | **Study design:** Retrospective cohort  **Source of data:** Clinical health records and health management information-system registry books  **Study dates:** 2013-2017  **Setting:** Hospital  **Countries:** Ethiopia | |
| Participants | **Participant eligibility:** patients with diabetes aged 18 or older  **Recruitment method:** Patients with diabetes registered with Debre Markos Referral Hospital for chronic follow-up care  **Participant Description:**   - all with diabetes (no gestational diabetes) - Age: median age 39.9 years - Sex: 55.7 % men   **Risk factors:**   - diabetes: all with diabetes - undernutrition: <18.5 16.4% - HIV infection: 7.6% - recent TB infection: 2.1% - history of untreated or inadequately treated TB disease: N/A - immunosuppressive therapy: N/A - cigarette smokers: 1.9% - drug or alcohol use disorder: 3.7% - socioeconomic statu: N/A - other:   - Place of residence: urban 37.6%, rural 62.4%   - Type of diabetes: T1D 51.7%, T2D 48.3%   - Blood glucose: ≤70 mg/dL 3.2%, 70-130 mg/dL 38.1%, ≥ 130 mg/dL 58.7%   - History of close contact 1.1%   - Duration of TB since DM diagnosis: ≤1 year 46.2%, >1-3 years: 53.9 years   Details of treatments received (treatment for diabetes, undernutrition and other concomitant conditions): Diabetes medication: OHA 45.5%, insulin 52.2%, OHA + insulin 2.3%. | |
| Outcomes | **Tuberculosis (active tuberculosis or tuberculosis disease)**  Definition and method for measurement of outcome: *Assessed via electronic database and health management information-system registry books .*  Was the same outcome definition (and measurement method) used in all patients? *Yes.*  Type of outcome: dichotomous/single  Time of outcome occurrence or summary of duration of follow-up: 5 years.  **TB recurrence (relapse or reinfection)**  *Not reported* | |
| Risk factor: undernutrition | Definition and method for measurement: *underweight (BMI l <18.5 kg/m²) compared to no underweight (BMI ≥18.5 kg/m²). Not described how BMI (weight and height) was assessed.*  Timing of measurement: *presumably at enrollment.*  Handling of predictors in the modelling: *undernutrition (<18.5 kg/m²) vs. no undernutrition (BMI ≥18.5 kg/m²).* | |
| Sample size, missing data and analysis | **Number of participants, person-years and outcomes/events in relation to the number of candidate predictors (Events Per Variable)** 433 participants (total)  BMI <18.5 kg/m²: 71 participants; 13 cases of incident TB; 212.5 PY  BMI ≥18.5 kg/m²: 362 participants; 13 cases of incident TB; 889 PY  **Number of participants with any missing value: not quantified:** persons with incomplete data or unavailable medical records were excluded  **Number of participants with missing data for each predictor:** see above  **Handling of missing data:** complete-case analysis  **Modelling method**  Bivariate and multivariate Cox regression models. Variables with P≤0.25 in bivariate analysis were fitted to the multivariate Cox proportional hazard regression model  **Assumption:** basic assumptions of the Cox proportional hazard model were checked using the Schoenfeld residuals test.  **Adjustment factors used:** Adjusted by type of diabetes, history of TB, diabetes medication, and history of alcohol. | |
| Results | **Interpretation**  Patients who were normal and overweight (BMI ≥18.5 kg/m2) were less likely to develop TB (HR 0.34. 95% CI 0.14-0.80; P=0.03) than their underweight (<18.5 kg/m2) counterparts, meaning: underweight participants are at increased risk for TB.  **Comparison with other studies**  The incidence rate is consistent with studies in India but not North India, Australia, Ethiopia, and Dessie. Findings on BMI and TB are consistent with studies from southeastern Amhara, Ethiopia, Egypt, the US and China.  **Discussion of generalizability**:  Only patients with DM and only in hospitals; not generalizable to patients without DM or patients with DM but not treated at the hospital.  **Limitations**   - retrospective design/secondary data use: not all important risk factors could be included - assessment of TB not reported in detail | |
| Funding | Funded by Debre Markos University. | |
| Conflict of interests | The authors report no conflicts of interest in this work. | |
| Notes | Email to the author (mihretie52@gmail.com) on 11/10/2023 asking about a possible duplicate publication with a different timeframe. As the author has not replied we included only one of the reports to avoid double-counting. | |
| ***Risk of bias*** | | |
| **Bias** | **Authors' judgement** | **Support for judgement** |
| Study participation | Unclear risk | Only about DM patients in one hospital. |
| Study Attrition | Unclear risk | Inclusion criteria was based on the availability of data, but rates of missing data is not available. |
| Prognostic Factor Measurement | Low risk | PF was diagnosed by clinicians for all participants according to the standard criteria. |
| Outcome Measurement | Unclear risk | Unclear about the detailed diagnosis criteria of TB, but all were from clinical records. |
| Study Confounding | Unclear risk | All except immunosuppresion, socioeconomic status. |
| Statistical Analysis and Reporting | Unclear risk | Desription of statistical model and selection of confounders specified; no subgroup or sensititivity analyses undertaken |

Getu 2022

| ***Study characteristics*** | | |
| Study details and sources | **Study design:** retrospective cohort study  **Source of data:** Medical records from St. Peters specialised hospital and Zewditu memorial hospital.  **Study dates:** 1 Jan 2016 - 30 November 2020  **Setting:** outpatient  **Countries:** Ethiopia | |
| Participants | **Participant eligibility:**  HIV-positive individuals (aged ≥15 years) on ART (Anti-Retroviral Therapy) enrolled at two selected hospitals in Addis Ababa between January 1, 2016, and August 25, 2020. At least one month for ART follow-up.  *Exclusion*: patients who had already developed TB before ART initiation transferred in from other institutions, with unknown date of initiation of treatment and outcome occurrence date.  **Recruitment method:**  All HIV-positive adults (aged ≥15 years) on ART (≥1 month of ART FU) were considered the target population. The required number of participants (according to sample size calculation) was selected using a simple random sampling technique through computer-generated numbers.  **Participant Description:**   - Age: age ≥15 years (median = 37 (29-45) - Sex: Men (226, 43%) and Women (303, 57%)   **Risk factors:**   - undernutrition (BMI) (18.5-24.9; >25; <18.5) - recent TB infection: NA (excl crit) - history of untreated or inadequately treated TB disease immunosuppressive therapy: no - socioeconomic status: Residence (urban or rural); Education (No; Primary, Secondary; Tertiary and higher); Occupation (Unemployed, Daily labor; Governmental employee; Private; Housewife) - other (specify)   - Haemoglobin level (1.55 (0.91-2.63))   - Baseline CD4 count (3.14 (1.64-7.10))   - Adherence (Good: 1.00, Fair: 0.45 (0.16-1.24), Poor: 2.16 (1.21-385)   - TB contact (Yes: 1.60 (0.74-3.44)   - IPT prophylaxis (Yes: 1.00, No: 2.78(1.06-7.30))   - Functional status (Working (1.00), Ambulatory (0.67(0.30-1.49), Bedridden (3.06(1.50-6.24))   - Baseline WHO stage (Stage I (1.00), Stage II (0.77(0.23-2.52), Stage III (2.33(1.08-5.02))   - Co-morbidity status (No: 1.00, Yes: 1.09(0.63-1.89)   - Opportunistic Infections (at enrolment) (No: 1.00, Yes: 1.55(0.92-2.63)   Details of treatments received: Anti-retroviral therapy (ART) | |
| Outcomes | **Tuberculosis (active tuberculosis or tuberculosis disease)**  Definition: occurrence of TB after ART initiation  Method for measurement of outcome: Bacteriologically confirmed (at least one positive AFB microscopy, Xpert MTB/Rif assay positive, or culture positive) or based on expert clinician by analysing the supportive evidence (suggestive of TB) during follow-up.  Was the same outcome definition (and method for measurement) used in all patients? No, numbers not reported.  Type of outcome (e.g., single or combined endpoints): incident TB infection  Were candidate predictors part of the outcome (e.g., in panel or consensus diagnosis)? No.  Time of outcome occurrence or summary of duration of follow-up:  Follow-up starts at the start of ART. Participants were censored when lost-to-FU (= missing an ART appointment for 1 to 3 months), death or 20 November 2020.  The study's duration was 4 years and 6 months (1529 PY of observation).  **Recurrent TB**  5 participants had re-treatment for TB but were not separately analysed | |
| Risk factor: undernutrition | Timing of measurement: BMI < 18.5 kg/m2  Handling of predictors in the modeling (e.g., continuous, linear, non-linear transformations or categorized): Categorical in different BMI ranges (<18.5; 18.5-24.9; >25) | |
| Sample size, missing data and analysis | Number of participants and outcomes/events: 529 (98%) were included in the final analysis—74 incident cases.  Number of outcomes/events in relation to the number of candidate predictors (Events Per Variable):   - 74 patients developed TB, with an overall incidence of 4.84 cases (95% CI: 3.83-6.11) per 100 PY. - 11 confounders were included in the analysis: per confounder, 6 TB cases   Number of participants with any missing value (including predictors and outcomes): 10 participants (2% of 539) were excluded because they didn´t contain the required information.  Number of participants with missing data for each predictor: In the analysis: none  Handling of missing data (e.g., complete-case analysis, imputation, or other methods): Participants with missing data were excluded from the population.  Modelling method   - STATA - Kaplan-Meier estimates for cumulative survival probability, overall and for subgroups (Log-rank test for differences) - Hazard ratio (HR) and 95% confidence intervals - Cox-proportional hazard regression; values with a p-value ≤ 0.20 in the bi-variate analysis were included in the multivariable model - P-value <0.05 from the multivariable analysis was considered statistically significant. - Cox-Snell residual plot for the model of the goodness of fit   Assumptions: Schoenfeld residual test and log (-log) (survival probability) versus log of survival time plot to check the proportional hazard assumption  Adjustment factors used:   - HB level (>10/<10 g/dl), baseline CD4 count (≤200/>200 cells/ul), adherence to ART (last known), TB contact (Y/N), TB treatment history (y/n), IPT prophylaxis (y/n), functional status at baseline (working/ambulatory/bedridden), WHO stage at baseline (stage I / stage II / Stage III/ IV), Comorbidity status (n/y), opportunistic infections at enrollment (n/y) | |
| Results | **Interpretation:**  Underweight: AHR: 2.42, 95% CI: 1.30-4.51) in comparison to BMI 18.5-24.9  **Comparison with other studies**  This study identified that being underweight was found to increase the hazard of TB compared to their counterparts. This finding is consistent with different studies done in Ethiopia, Tanzania, and South Africa [References 5,30,31,32].  **Discussion of generalizability:**  Not mentioned  **Strengths and limitations.**  *Limitations*: Some important variables like housing conditions, family size, household incomes, and substance use were not included.  Since the study was facility-based, it does not capture HIV-positive individuals who are out of care (At the community level). | |
| Funding | The authors received no specific funding for this work | |
| Conflict of interests | The authors have declared that no competing interests exist. | |
| Notes | We assume the order of the events and censors do belong together but are not listed for the correct BMI class. We use the numbers in Table 2.  14 February 2024 Email to the author antesha04@gmail.com to confirm assumption and ask for data on culture. | |
| ***Risk of bias*** | | |
| **Bias** | **Authors' judgement** | **Support for judgement** |
| Study participation | Unclear risk | It focused on HIV-positive individuals in two selected hospitals. |
| Study Attrition | Low risk | Minimal attrition: 2%. |
| Prognostic Factor Measurement | Low risk | BMI was based on clinical records. |
| Outcome Measurement | Unclear risk | Though the same clinical criteria was used for all participants, it's not unclear how many were culture-confimed. |
| Study Confounding | Unclear risk | No reports about DM, history of TB, immunosuppression, alcohol use, smoking |
| Statistical Analysis and Reporting | Unclear risk | Desription of statistical model and selection of confounders specified; no subgroup or sensititivity analyses undertaken |

Hanrahan 2010

| ***Study characteristics*** | | |
| Study details and sources | **Study design:** prospective clinical cohort study  **Source of data:** medical records, questionnaire  **Study dates:** July 2003 to December 2008  **Setting:** Outpatient  **Countries:** South Africa | |
| Participants | **Participant eligibility**  HIV-infected adults getting treated in the Perinatal HIV Research Unit (PHRU). Exclusion: Participants with TB at baseline had lost more than 10% of their body weight between the first and second visit (according to the WHO definition of wasting [19]) and/or had a baseline weight of less than 45 kg.  **Recruitment method**  Participants were either self-referred or identified through voluntary counselling and testing programs or research programs at the PHRU.  Ongoing data collection visits were scheduled 4-7 months apart. A follow-up questionnaire was administered, collecting inter alia information on hospital admission, TB symptoms, HAART (highly active antiretroviral therapy) use and compliance, and other medications. Weight and CD4 count were measured at every visit.  **Participant description**   - Age: over 18 (median age 33 yrs (28-38)) - Sex: Men (61%) and Women (63%)   **Risk factors:**   - diabetes: not mentioned - undernutrition (underweight (BMI <=18.5), normal (BMI 18.5-25), overweight (BMI 25.1-30), and obese (BMI >30). - HIV infection: all participants had HIV - recent TB infection: history of TB (n, %) - history of untreated or inadequately treated TB disease immunosuppressive therapy: no - cigarette smokers: not mentioned - drug or alcohol use disorders: not mentioned - socioeconomic status: baseline household income (<1000 Rand/month, 1000-5000 Rand/month, and > 5000 Rand/ month) - other (specify)   - Baseline CD4 count (<200, 200-349, 350-500, and >500 cells/ul)   - Years since HIV diagnosis (IQR: interquartile range)   - TB at baseline visit (n, %)   - Employed   - IPT use   - HAART use   **Details of treatments received**  HIV: symptomatic patients were provided with syndromic sexually transmitted infection treatment. Patients were put on HAART when they became eligible for the then-current South African guideline (CD4 cell count <200 cells/ul or WHO stage 4 illness).  TB: Isoniazid preventive therapy (IPT) was offered to patients with a positive tuberculin skin test (TST) until January 2009, after which it was offered to all those without a history of prior TB, as well as no signs of active TB.  Family planning methods were offered to women.  HAART and cotrimoxazole use was treated as a time-varying covariate, but once initiated, it was assumed to continue interrupted until censoring or death. IPT use was treated time-varying. | |
| Outcomes | **Tuberculosis**  Definition and method for measurement of outcome: was defined as the first instance of any one of the following: Recorded initiation of multidrug TB therapy, presence of acid-fast bacilli on microscopy or a biopsy suggestive TB, mycobacterial culture positive for acid-fast bacilli of Mycobacterium tuberculosis, being admitted to hospital with a diagnosis of TB, or cause of death ascribed to TB.  Was the same outcome definition (and method for measurement) used in all patients? Yes, following the definitions mentioned above.  Type of outcome (e.g., single or combined endpoints): TB infection.  Time of outcome occurrence or summary of duration of follow-up: The time to incident TB was recorded from the date of baseline visit to the date of first occurrence of TB for those without TB at the baseline visit.  **Recurrent TB**  Not reported. | |
| Risk factor: undernutrition | Definition and method for measurement: BMI (kg/m2) categorised according to the WHO: underweight (BMI <=18.5), normal (BMI 18.5-25), overweight (BMI 25.1-30), and obese (BMI >30). Wasting was defined as losing 10% of the body weight between the first two visits.  Timing of measurement (e.g., at patient presentation, at diagnosis, at treatment initiation): At baseline and as a time-varying covariate  Were predictors assessed blinded for outcome and for each other (if relevant)? Yes  Handling of predictors in the modelling (e.g., continuous, linear, non-linear transformations or categorised): Categorical in different BMI ranges. | |
| Sample size, missing data and analysis | Number of participants and number of outcomes/events: 3,456 total participants; 226/3456 with 4.5/100 person-years  Number of participants, person-years and outcomes/events in relation to the number of candidate predictors (Events Per Variable)  Incidence rates for each baseline BMI category were as follows:   - underweight, 7.3/100 person-years (95% CI 4.9–10.9); normal, 6.0/100 person-years (95% CI 5.1–7.0); overweight, 3.2/100 person-years (95% CI 2.4–4.4); and obese, 1.9/100 person-years (95% CI 1.2–3.0). - TB incidence among those not on HAART was 4.7/100 person-years (95% CI 4.0–5.8) - For those on HAART was 4.1/100 person-years (95% CI 3.2–5.3).   → The results remained significant and unchanged after excluding 681 individuals with either prevalent TB or a history of TB  Number of participants with any missing value (include predictors and outcomes): 179  Number of participants with missing data for each predictor: 130 (3%) were excluded due to missing baseline CD4 cell count; 46 (2%) due to missing baseline weight; 3 (<1%) due to missing date of birth.  Handling of missing data (e.g., complete-case analysis, imputation, or other methods):  Patients not returning for study visits were actively followed through phone calls, reminders mailed to their addresses, and home visits. death was ascertained through family contacts or home visits and were verified by death certificate whenever available.  Modelling method   - STATA - baseline values of BMI are reported using the mean value of these imputed heights, whereas those in the Cox proportional hazard model results are reported using Rubin´s pooling method. - Stata ICE package was used to perform multiple imputation - Confidence intervals (CIs) for binomial proportions were calculated using the Wlad technique and the log-rank statistic was used to compare Kaplan-Meier curves. - Cox proportional hazard models were used for all survival analysis - The proportional hazard assumptions were checked using Schoenfeld residuals. - Statistical significance was set at 0,05 for all analyses.   Assumptions: not mentioned  Adjustment factors used: adjusted for age, sex, years since HIV diagnosis | |
| Results | **Interpretation:**   - TB incidence rates by baseline BMI were 7.3/100 person-years for underweight, 6.0/100 person-years for normal, 3.2/100 person-years for overweight, and 1.9/100 person-years for obese. - Compared to those with normal BMI, those with overweight and obese BMI were at a significantly reduced risk of developing TB (0.56 (95% CI 0.38-0.83) and 0.33 (95% CI 0.19-0.55).   **Comparison with other studies:**   - Shor-Posner et al. [Reference in the report 8] reported an inverse relationship between BMI and mortality, independent of baseline CD4 cell count in a cohort of HIV-positive intravenous drug users in Miami - Shuter et al. [Reference in the report 9] found that overweight individuals had slower disease progression and lower viral load among a cohort of AIDS-free HIV-positive individuals in New York City after adjusting for baseline CD4 cell count and time to antiretroviral initiation. - Among a multisite US cohort of HIV-positive women, Jones et al. [Reference in the report 7] found that both higher baseline and time-varying BMI were associated with slower disease progression, lower risk of clinical AIDS, and decreased risk of HIV-related mortality after adjusting for CD4 cell count and viral load. - Individuals with obese and overweight BMI were also recently shown to be at a lower risk of TB, as compared to those with normal and underweight BMI, in a large cohort of elderly health-centre patients in Hong Kong [Reference 20]. - Two earlier large studies, one conducted by Tverdal in Norway in 1986 [Reference in the report 21], showed a decreased risk of TB with increasing BMI, and one by Edwards et al. in 1971 [Reference 22], found a decreased progression to active TB disease among US naval recruits with an ‘overweight body build’ compared to those who were underweight. - Recently, in Mumbai, India, overweight men and women were shown to be at decreased risk for death attributed to TB [Reference in the report 23]. - Differences in micronutrient intake could also be implicated in the associations shown here, as many micronutrient deficiencies have been described among those with TB and HIV [25–29, and in children 30, only vitamin C; reference in the report 31]. - Effectiveness of HAART in reducing both mortality and incident TB among HIV-positive individuals (References in the report 32-35).   **Discussion of generalizability:**  Not mentioned.  **Strengths and limitations:**  *Limitations*:   - It was conducted using a clinical cohort relying on self-reports for many exposure variables. The overall mortality rate reported here (3.5%) suggests an incomplete reporting of deaths among the cohort and that a portion of those lost to follow-up are likely misclassified deaths; however, there is no reason to believe that such misclassification would occur differentially by participant BMI. - The temporality of exposure and outcome is also an issue in this study, particularly in looking at TB, of which weight loss is one classic symptom. - Residual confounding due to unmeasured factors – particularly socioeconomic – cannot be excluded, though they did include employment and household income in our multivariate models. - The effect of cotrimoxazole use on mortality within this cohort could explain the attenuated effect size [aHR 0.84 (95% CI 0.65–1.1)]. - They have no measures of viral load, dietary intake, or plasma micronutrient levels, making it difficult to identify a single protective factor.   *Strengths*   - The study includes several thousand HIV-infected adults using easily measured anthropomorphic measures in a cohort from a relatively well-resourced setting with access to both cotrimoxazole and HAART. | |
| Funding | "Patient care is funded by a US PEPFAR grant through USAID South Africa (674-A-00-08-00009-00). NIH grants AI066994, AI001637, HL090312, and AI048526 supported the analysis. T.M. received research training through Fogarty International Center Grants (2RTW007370/3). C.H. received travel support through an award from the Johns Hopkins Center for Global Health. The opinions herein do not necessarily reflect those of USAID or the US Government." | |
| Conflict of interests | Not available. | |
| Notes | 13 February 2024 - Email to Neil1.martinson@gmail.com requesting data on culture and adjusted analyses.  14 February 2024 - They provided data on culture; no further analysis were available. | |
| ***Risk of bias*** | | |
| **Bias** | **Authors' judgement** | **Support for judgement** |
| Study participation | Unclear risk | It focused on HIV-infected individuals in one local hospital. |
| Study Attrition | Low risk | Minimal attrition: 4.9%. |
| Prognostic Factor Measurement | Low risk | BMI was based on clinical records. |
| Outcome Measurement | Low risk | The authors clarify that the majority were laboratory confirmed cases and tested with smear and culture. |
| Study Confounding | Unclear risk | No reports about DM, history of TB, immunosuppression, alcohol use, smoking. |
| Statistical Analysis and Reporting | Low risk | Adequately presented Cox regression analysis for the defined model. |

Jung 2016

| ***Study characteristics*** | | |
| Study details and sources | **Study design:** retrospective cohort study  **Source of data:** clinical health records  **Study dates:** January 1st, 2007 to December 31st, 2009  **Setting:** Outpatient  **Countries:** South Korea | |
| Participants | **Participant eligibility:**  Patients who underwent gastrectomies for gastric cancer at Severance Hospital. Patients who received chemotherapy after gastrectomy (n = 1836), who had a history of previous gastrectomy (n = 10), who were treated for TB within six mo before surgery or developed TB within two months after surgery  (n = 8), and those who visited the hospital only once after surgery (n = 42) were excluded.  Recruitment method: Retrospective data collection of 2933 patients who underwent gastrectomies for stomach cancer; after exclusion, the study population consisted of 1776 patients. The Korean Tuberculosis Surveillance Center provided data covering the incidence of TB in the general population, and an estimate of the total population was obtained from the Korean National Statistical Office.  **Participant Description:**   - Age: median 58 y, range: <40, 41-60, 61-80, >80 - Sex: male 65.1%, female 34.9%   **Risk factors:**   - diabetes: 9.7% - undernutrition: BMI < 18.5 (4.0%)   - Postoperative BMI (kg/m2) - HIV infection: NR - recent TB infection: previous TB infection (11.3%) - history of untreated or inadequately treated TB disease: NR - immunosuppressive therapy: NR - cigarette smokers: yes (49.9%) - drug or alcohol use disorders: alcohol (yes; 33.4%) - socioeconomic status: NR - other (specify)   - Laboratory data: Hemoglobin, Albumin, Cholesterol   - ASA physical status: Class I, II, III   - Surgery extent, total gastrectomy   - Cancer Stage: I A, I B, II, III, IV   - Cancer cell type: Adenocarcinoma, Signet-ring cell, others   - Death   Details of treatments received (treatment for diabetes, undernutrition and other concomitant conditions): Not described. | |
| Outcomes | **Tuberculosis (active tuberculosis or tuberculosis disease)**  Definition and method for measurement of outcome:  *Active TB infection:* TB diagnosis was confirmed if Mycobacterium tuberculosis (M. tuberculosis) was isolated in the culture of any clinical specimen or if M. tuberculosis DNA was identified by polymerase chain reaction from any clinical specimen. Histopathological diagnoses were also accepted.  Patients who had a high clinical likelihood of active TB and a negative mycobacterial culture finding but who also had good clinical and radiographic responses to anti-TB treatment were also included as active TB patients.  *Previous TB infection*: was defined as a history of TB treatment or radiological evidence of previously healed TB. Board-certified pulmonologists reviewed chest films. Abnormal chest radiographic findings consistent with previously healed TB were defined as fibro-nodular lesions and multiple non-calcified nodules in the upper zones of the lung.  Was the same outcome definition (and method for measurement) used in all patients? 8x culture-based, 5x histologically-based, 3x clinically-based  Type of outcome: (e.g., single or combined endpoints)  Time of outcome occurrence or summary of duration of follow-up: The mean duration of follow-up was 1469 (range: 42-2279) days. Of the patients, 1687 (95%) were followed for more than one year and 1609 (92.1%) of the survivors were followed for more than two years.  **TB recurrence (relapse or reinfection)**  Not measured | |
| Risk factor: undernutrition | Definition and method for measurement: Measured BMI before gastrectomy; < 18.5, 18.5-24.99, ≥ 25  Measured BMI about 1 yr after gastrectomy; only median and IQR.  Timing of measurement: (e.g., at patient presentation, at diagnosis, at treatment initiation)  Handling of predictors in the modelling (e.g., continuous, linear, non-linear transformations or categorised): Only the category “low BMI” was analysed | |
| Sample size, missing data and analysis | Number of participants, number of outcomes/events and amount of accumulated person-years (PY): 1,776 participants with gastrectomy; mean follow-up duration 1,469 days; 16 cases of TB; no person time collected.  Number of participants, person-years and outcomes/events in relation to the number of candidate predictors (Events Per Variable): 6 confounders for 16 cases (2.6 cases per confounder)  Number of participants with any missing value (include predictors and outcomes): Not reported  Number of participants with missing data for each predictor: From table 1 it appears that 4 patients have no BMI data (none of them developed TB)  Handling of missing data (e.g., complete-case analysis, imputation, or other methods): excluded from analysis.?  Modelling method:   - Categorical variables were analyzed using the chi-square test or Fisher’s exact test. - Continuous variables were analyzed using the t-test or Mann-Whitney U-test. - To evaluate independent risk factors for post-gastrectomy TB, multivariate analysis using logistic regression was conducted using potentially associated explanatory variables detected in a univariate analysis (P < 0.1). - Multi-collinearity of these variables was checked, and the goodness-of-fit of the model was verified using the Hosmer‐Lemeshow test. - Standardized incidence ratios (SIRs) of TB were calculated to compare the incidence of TB in gastrectomy patients with that in the general Korean population. - The SPSS software (ver. 19; SPSS Inc., Chicago, IL, United States) was used for all statistical analyses.   Assumptions: The 95% confidence intervals (CIs) for the SIRs were estimated by assuming that the observed cases had a Poisson distribution using Byar’s normal approximation method.  Adjustment factors used: sex, smoking, alcohol, low BMI, previous TB infection, surgery extent, total gastrectomy. | |
| Results | **Interpretation**  "Among the 1,776 gastrectomy patients, 0.9% (16/1776) developed post-gastrectomy TB, with an incidence of 223.7 cases per 100,000 patients per year. The overall incidence of TB in gastrectomy patients, adjusted by sex and age, was significantly higher than that in the general population (SIR = 2.22, 95% CI: 1.27-3.60). Previous TB infection [odds ratio (OR) = 7.1, P < 0.001], lower body mass index (BMI) (kg/m2; OR = 1.21, P = 0.043) and gastrectomy extent (total gastrectomy vs subtotal gastrectomy) (OR = 3.48, P = 0.017) were significant risk factors for TB after gastrectomy in a multivariate analysis."  **Comparison with other studies**   - [References 3,4] Risk factors for the development of active TB include HIV infection, TB scars on chest radiographs, diabetes, malnutrition, chronic renal failure, silicosis, and transplantation. - [References 16-18] Malnutrition increases susceptibility to TB by impairing the immune response. - [References 8,11,19] Low BMI and TB Those results can be interpreted as being consistent with the explanation given above as well as in previous reports.   **Discussion of generalizability**  “Another limitation of our study is that it was retrospective in design and conducted at a single medical centre. Thus, our results may not reflect those at other institutions or in other areas.”  **Strengths and limitations**  *Limitations*:   - Most patients had not undergone a tuberculin skin test (TST) or interferon-gamma release assay (IGRA), which are relevant to the risk of developing TB and useful in LTBI diagnosis. - Another limitation of our study is that it was retrospective in design and conducted at a single medical centre. - Additionally, the follow-up period differed among subjects, and patients lost to follow-up could have developed TB. - Some risk factors for TB development were not available for analysis   Strengths: The authors investigated a greater variety of parameters than previous studies, including those related to TB risk and those related to surgery and gastric cancer. | |
| Funding | The data on funding was not available. | |
| Conflict of interests | None of the authors has a conflict of interest. | |
| Notes | 14 February 2024 mdkang@yuhs.ac about data on risk of bias. | |
| ***Risk of bias*** | | |
| **Bias** | **Authors' judgement** | **Support for judgement** |
| Study participation | Unclear risk | It focused on gastric cancer patients after gastrectomies in one local hospital. |
| Study Attrition | Low risk | Presumably no attrition. |
| Prognostic Factor Measurement | Low risk | BMI was based on clinical records. |
| Outcome Measurement | Unclear risk | Though the same clinical criteria was used for all participants, it's not unclear how many were culture-confimed. |
| Study Confounding | Unclear risk | No reports about HIV, history of TB, immunosuppression, socioeconomic status. |
| Statistical Analysis and Reporting | Unclear risk | Desription of statistical model and selection of confounders specified; no subgroup or sensititivity analyses undertaken |

Kim 2018

| ***Study characteristics*** | | |
| Study details and sources | **Study design:** retrospective cohort study  **Source of data:** clinical health records  **Study dates:** January 1, 2002 - December 31, 2013  **Setting:** Outpatient  **Countries:** South Korea | |
| Participants | **Participant eligibility:**  Inclusion criteria: Participants who participated in national health examination services between January 1, 2002 and December 31, 2006 and were aged 20-89 years in 2007. Exclusion criteria: individuals diagnosed with TB before January 2007 and patients with human immunodeficiency virus (HIV) infection.  **Recruitment method:**  A proportionate stratified random sample of individuals who visited hospitals under the National Health Insurance Database (NHI) program was drawn.  **Participant Description:**  Baseline characteristics were described according to BMI category (<18.5, 18.5–23, 23–25, 25–30, and >=30 kg/m²)   - Age: mean age (SD) ranged between 41.7 (17.1) and 50.6 (13.0) years - Sex: percentage male ranged between 31.4 and 62.6%   **Risk factors:**   - Diabetes: ranged between 1.2 and 5.9% - undernutrition: 4.0% BMI <18.5 kg/m2. - HIV infection: excluded from the study - recent TB infection: NR - history of untreated or inadequately treated TB disease immunosuppressive therapy: 0% (exclusion criterion) - cigarette smokers: ranged between 20.0 and 28.3% - drug or alcohol use disorders: alcohol cunsumption ranged between 41.5 and 49.7% - socioeconomic status: Education: NR - other (specify):   - Household income: <50th percentile versus ≥50th percentile, between32.5 and 43.3% were <50th percentile   Details of treatments received (treatment for diabetes, undernutrition and other concomitant conditions): NR | |
| Outcomes | **Tuberculosis (active tuberculosis or tuberculosis disease)**  Definition: incident TB was defined as the first recorded diagnosis of TB based on the ICD-10 code (A15.x-A19.x) during the follow-up period.  Method for measurement of outcome: extracted from the National Health Insurance database; no further details provided.  Was the same outcome definition (and method for measurement) used in all patients? Yes.  Type of outcome: single endpoint.  Time of outcome occurrence or summary of duration of follow-up: between January 1. 2007, and December 31, 2013; censoring events not defined.  **Recurrent TB**  Not reported. | |
| Risk factor: undernutrition | Definition: BMI <18.5 kg/m².  Method for measurement: not detailed, extracted from clinical records.  Timing of measurement: the record which date was closest to January 2007.  Handling of predictors in the modeling: Categorized into five BMI groups: <18.5, 18.5 to 22.9, 23.0 to 24.9, 25.0 to 29.9, and 30.0 kg/m². | |
| Sample size, missing data and analysis | Number of participants, number of outcomes/events and amount of accumulated person-years (PY): 313,425 participants undderwent the national health examination between 2002 and 2006; 301,081 participantswere included in the analyses, with a total person-years of follow-up of 1,919,106 person-years for the TB outcome. There were 3,772 cases of incident TB.  Number of participants, person-years and outcomes/events in relation to the number of candidate predictors (Events Per Variable) 5 Confounders were analyzed for 3,772 cases of TB (754.4 cases per confounder)  Number of participants with any missing value (include predictors and outcomes): participants with TB at baseline (+ 4 years), and those infected with HIV.  Number of participants with missing data for each predictor: 2,242 subjects were excluded because of diagnosis of TB between January 1, 2002 and December 31, 2006, and 102 patients for infection with HIV.  Handling of missing data (e.g., complete-case analysis, imputation, or other methods): Those with TB and HIV are excluded from the analysis.  Modelling method:   - A multivariate Cox proportional hazards regression analysis was performed to evaluate the effects of BMI on the incidence of TB. - All statistical analyses were performed using SAS version 9.3 software (SAS Institute Inc., Cary, NC)   Assumptions: Before the analysis, the assumption of proportionality was confirmed by Schoenfeld residuals.  Adjustment factors used: Multivariate Cox proportional hazards model was used, adjusted by sex, household income, smoking status, alcohol use and the presence or absence of DM. | |
| Results | **Interpretation**  "Among 301,081 individuals, 3,772 (1.26%) incident TB cases were detected. The incidence rate of the event was 19.65 per 10,000 person-years. Development of TB was decreased as BMI was increased, being 2.1% for <18.5 kg/m2, 1.6% for 18.5–22.9 kg/m2, 1.1% for 23–24.9 kg/m2, 0.8% for 25–29.9 kg/m2, and 0.7% for 30 kg/ m2 (P <0.001)."  **Comparison with other studies**   - (Reference 1) Low body mass index (BMI) is an important risk factor for the development of tuberculosis (TB). - (References 2-5) There is growing evidence that high BMI is a protective factor of TB. - (Reference 8) A BMI above 28 kg/m2 was independently associated with host susceptibility to TB in rural China.   **Discussion of generalizability**  Not reported.  **Strengths and limitations**  *Strengths*:   - It was a nationwide population-based study that included the largest study population compared with previous studies. - Furthermore, the authors performed stratification analyses based on various subgroups. - Additionally, there was a time lag between the record of the latest BMI and the diagnosis of TB based on the ICD-10 code. - Moreover, overall mortality between high BMI groups was evaluated in our study.   *Limitations*:   - This study was a retrospective study. - In addition, BMI does not distinguish between adipose fat, muscle, bone, and water. - Third, the authors could not evaluate hormonal levels or HbA1c because they used insurance claim data. - Finally, the authors could not clarify the exact underlying pathophysiology of the effect of BMI on reduced risk of incident TB. | |
| Funding | The authors received no specific funding for this work. | |
| Conflict of interests | The authors have declared that no competing interests exist. | |
| Notes | 15 February 2024 Email cem@ewha.ac.kr about missing data and culture. | |
| ***Risk of bias*** | | |
| **Bias** | **Authors' judgement** | **Support for judgement** |
| Study participation | Low risk | Large national database |
| Study Attrition | Unclear risk | For some variables in Table 1, it was apparent that missing data amounted more than 10%. |
| Prognostic Factor Measurement | Low risk | PF was diagnosed by clinicians for all participants according to the standard criteria. |
| Outcome Measurement | Unclear risk | Though the same clinical criteria was used for all participants, it's not unclear how many were culture-confimed. |
| Study Confounding | Unclear risk | All except previous and recent TB, immunosuppressive therapy. |
| Statistical Analysis and Reporting | Low risk | Adequately presented Cox regression analysis for the defined model. |

Kyaw 2022

| ***Study characteristics*** | | |
| Study details and sources | **Study design:** prospective cohort study  **Source of data:** clinical assessment and clinical health records  **Study dates:** January 2011 to 31, August 2018  **Setting:** outpatient  **Countries:** Myanmar | |
| Participants | **Participant eligibility**  Participants included persons living with HIV (PWH) at least 15 years who enrolled in the Integrated HIV Care Program between January 2011 and August 2017. Exclusion criteria included TB treatment before enrollment or isoniazid TB preventive treatment during the follow-up.  **Recruitment method**  Data was routinely collected from the Integrated HIV Care Program. There were 49 HIV clinics operated by this program which were located in five out of 15 states and regions in Myanmar.  **Participant Description**   - Age: ≥15 (median = 36 years) - Sex: 55% male, 45% female   **Risk factors**   - diabetes: Random blood glucose (RBG): < 110, 100–139, 140–199, and > 199 mg/dl. - undernutrition: BMI - HIV infection: Antiretroviral therapy (ART) status: yes or no - recent TB infection: NR - history of untreated or inadequately treated TB disease immunosuppressive therapy: NR - cigarette smokers: NR - drug or alcohol use disorders: never, weekly, or daily - socioeconomic status: NR - other (specify)   - Hepatitis B: positive or negative serology   - Hepatitis C: positive or negative serology   - Anaemia: based on HB levels - normal (male, 13 g/dl; female, 12 g/dl), mild (male, 11–12.9 g/dl; female, 11–11.9 g/dl), moderate (both sexes, 8.0–10.9 g/dl), and severe anaemia (both sexes <8.0 g/dl)   - CD4+ cell count: cells/ul   **Details of treatments received (treatment for diabetes, undernutrition and other concomitant conditions):**   - For TB: all patients diagnosed with TB were immediately initiated on the WHO-recommended treatment regimen unless they died or were lost to follow-up - The preferred three-drug ART regimen during the study period included tenofovir or stavudine, lamivudine or zidovudine, and nevirapine or efavirenz. | |
| Outcomes | **Tuberculosis (active tuberculosis or tuberculosis disease)**  Definition and method for measurement of outcome: incident TB, defined as patients starting first-line TB treatment regimens that included isoniazid, rifampicin, ethambutol, pyrazinamide, or streptomycin (either for pulmonary or extrapulmonary disease)  Was the same outcome definition (and method for measurement) used in all patients? Incident TB was measured with sputum smear microscopy for acid-fast bacilli, a sputum specimen for Xpert MTB/RIF assay, and/or chest radiograph. All patients had the same measurement when symptoms of TB were shown.  Type of outcome: incident TB, single endpoint.  Time of outcome occurrence or summary of duration of follow-up: From the date of enrollment and date of death, loss to follow-up, treatment transfer, or end of the study (31 August 2018). Median follow-up of 2.2 years. Patients were screened for TB by symptom evaluation at enrollment and at every follow-up visit (monthly or quarterly).  **Recurrent TB**  Not measured. | |
| Risk factor: undernutrition | Definition and method for measurement: BMI was categorized as low, normal, overweight, and obese following WHO guidelines and was also dichotomized as low (<18.5 kg/m2 ) or normal/high (18.5 kg/m 2). Data was clinically assessed.  Timing of measurement: (e.g., at patient presentation, diagnosis, treatment initiation): at baseline.  Handling of predictors in the modelling: categorized as mentioned above. | |
| Sample size, missing data and analysis | Number of participants, number of outcomes/events and amount of accumulated person-years (PY): participants included in the study = 20,865. During a median follow-up of 2.2 years (IQR: 0.5 – 4.2), 17% (n = 3,628) developed active TB among 53,880 person-years (PY) follow-up.  Number of participants, person-years and outcomes/events in relation to the number of candidate predictors (Events Per Variable): for joint exposure.  Number of participants with any missing value (include predictors and outcomes): 38% of the eligible patients were excluded from the analysis due to missing data on BMI or RBG.  Number of participants with missing data for each predictor: Of eligible patients, 12,928 records did not have RBG, weight, or height measurements at enrollment and were excluded.  Handling of missing data (e.g., complete-case analysis, imputation, or other methods): To account for competing risk due to death and loss to follow-up before the incident TB event, they have fitted a cause-specific model and a subdistribution hazard model.  Modelling method:   - TB incidence rates were calculated by dividing the number of incident TB cases by the total number of person-years of follow-up. - Hazard rate ratios and 95% confidence intervals (CIs) were estimated using Cox proportional hazard models to determine patient characteristics associated with incident TB. - Nelson–Aalen cumulative hazard estimates were used to plot the cumulative hazard of incident TB during follow-up, stratified by BMI categories and hyperglycemia status. - They also estimated the relative excess risk of incident TB due to interaction or synergy (RERI), the attributable proportion of incident TB due to synergy, and the synergy index, along with their 95% CI. - Sensitivity analysis to assess systematic error due to outcome misclassification by estimating hazard ratios using an alternate definition of incident TB.   - In this sensitivity analysis, incident TB was defined only among those who started TB treatment more than 3 months after enrollment to exclude those patients who could have had prevalent TB at enrollment due to masking of TB in the early ART period. - STATA USA, College Station, Texas, USA) was used for all analyses   Assumptions: Proportional hazard assumptions were assessed using log-log survival curves and with goodness-of-fit using Schoenfeld residual  Adjustment factors used: overall incidence of TB: not adjusted. The joint exposure analysis was adjusted for age, sex, CD4+ cell count, anaemia and alcohol use at registration, and ART status during follow-up. | |
| Results | **Interpretation**  "There is a high incidence of TB among those with concurrent low BMI and hyperglycemia at enrollment to HIV care. The incidence rate of TB in the jointly exposed group was 21 cases per 100 PY, two times higher than the rate of those with low BMI alone and four times higher than the rate of those with hyperglycemia alone. In addition, our results suggested that almost one quarter (23%) of incident TB among PWH with low BMI and hyperglycemia occurred due to the synergistic nature of these two combined exposures."  **Comparison with other studies**   - [References 4,5, 6,7] One established risk factor for TB in PWH is low BMI. - [References 10–13] Some studies have reported that synergy (joint exposure) due to low BMI and T2DM increases TB incidence, whereas other studies have reported no difference in TB risk. - [References 11,12] Large cohort studies from Singapore (n = 63 257) and from Taiwan (n = 119 340) both reported that patients with low BMI and T2DM had the highest risk of TB compared with those with low BMI only or T2DM only.   **Discussion of generalizability**  Not reported.  **Strengths and limitations**  *Limitations*:   - First, some misclassification of hyperglycemic status was likely given they used RBG to measure glycemic status.RBG has low sensitivity and specificity for defining hyperglycemia. - Second, they measured RBG and BMI at a one-time point and did not have information on the duration of hyperglycemia.   - They were also unable to determine if patients had a previous history of diabetes mellitus diagnosis or treatment for hyperglycemia. - Third, 38% of the eligible patients were excluded from the analysis due to missing data on BMI or RBG. - Fourth, unmeasured confounders such as smoking and socioeconomic status may have resulted in bias. - Fifth, they could not determine what proportion of incident TB cases were laboratory confirmed (smear or Xpert positive) versus those diagnosed by chest radiograph or other clinical information.   *Strengths*: not reported | |
| Funding | The study was partially funded by the National Institutes of Health (R03AI133172, R21AI156161, R21TW011157, and R01AI153152 to M.J.M.). The funder has no role in study design; in the collection, analysis, and interpretation of data; in the writing of the report; and in the decision to submit the article for publication. | |
| Conflict of interests | All authors have declared that they have no conflict of interest. | |
| Notes | 15 February 2024 Email to the author nangthu82@gmail.com about risk of bias and adjustments. | |
| ***Risk of bias*** | | |
| **Bias** | **Authors' judgement** | **Support for judgement** |
| Study participation | Unclear risk | It focused on HIV patients in several regions. |
| Study Attrition | High risk | 38% attrition and lost to follow-up was not described. |
| Prognostic Factor Measurement | Low risk | Based on clinical assessment. |
| Outcome Measurement | Unclear risk | Though the same clinical criteria was used for all participants, it's not unclear how many were culture-confimed. |
| Study Confounding | Unclear risk | No reports about DM, history of TB, immunosuppression, alcohol use, smoking. |
| Statistical Analysis and Reporting | Low risk | Adequately presented Cox regression analysis for the defined model. |

Leung 2007

| ***Study characteristics*** | | |
| Study details and sources | **Study design:** prospective cohort  **Source of data:** questionnaires, medical examination  **Study dates:** January 1, 2000 to December 31, 2005  **Setting:** Outpatient  **Countries:** Hong Kong | |
| Participants | **Participant eligibility**  Elderly above 65 years enrolled in health maintenance programs of 18 Elderly Health Centers from Jan-Dec 2000.   *Exclusion*: known active TB on presentation or active tuberculosis notified within three months of enrollment; missing or incomplete info on sex, age, weight and/or height; missing or invalid ID numbers.  **Recruitment method**  Enrolled from January 1 to December 31, 2000, in one of the 18 elderly health centers. In the recruitment method described in reference 9, they followed up using their identity card, in which all medical data is registered and cross-matched with the death and tuberculosis notification registries.  **Participant description**   - Age: > 65 years or older (mean 72.6 y; no SD) - Sex: Male 34.7%   **Risk factors**   - diabetes: No, HbA1c (<7%, >=7%, not measured at enrollment) - undernutrition: BMI (<18.5 (underweight), 18.5 - <23 (normal), 23 - <25 (at risk), 25 - < 30 (overweight), and >=30 (obese)). - HIV infection: voluntarily testing - recent TB infection: NR(89 were excl due to TB at baseline or within 3 months after baseline). Tab 2: 82/477 were retreatement cases. Info on previous TB was retrieved from the notification registry, yet no numbers were reported. - History of untreated or inadequately treated TB disease immunosuppressive therapy: NR Tab 2: 82/477 were retreatment cases. - cigarette smokers: never (70.8%), ex-smoker (20.2%), current (9.0%) - drug or alcohol use disorders: never (71.2%), ex-drinker (10.1%), social drinker (14.9%) and regular drinker (3.8%) - socioeconomic status:Education: postsecondary (3.6%), secondary (13.7%), primary (37.5%), no formal (16.7%), illiterate (28.6%) - other (specify)   - Housing: public, privately rented, privately owned, other   - working part-time or fully   - receiving means-tested public assistance   - Language: cantonese, mandarin, other dialect, other   - Marital status: never, married, widowed, divorced, or other   - Cholesterol: <200.8 mg/dL, 200.8-239.4, >239.4, not measured   - Hypertension   - Cardiovascular disease   - COPD and/or asthma   - malignant disease   - Recent weight loss   - Admission within 1 year   - Activities of daily living score, mean   Details of treatments received (treatment for diabetes, undernutrition and other concomitant conditions): no treatment | |
| Outcomes | **Tuberculosis (active tuberculosis or tuberculosis disease)**  Definition and method for measurement of outcome:  An active case of tuberculosis was defined as disease proved by isolation of Mycobacterium tuberculosis or, in the absence of bacteriological confirmation, disease diagnosed on clinical, radiological, and/or histological grounds with an appropriate response to antituberculosis treatment.  Was the same outcome definition (and method for measurement) used in all patients? Active tuberculosis (all cases), culture confirmed (68.3%),Tab 2: 82/477 were retreatment cases.  Type of outcome: single (results for all (active) cases of TB and for lab-confirmed subgroup  Time of outcome occurrence or summary of duration of follow-up:  Development of active tuberculosis from 3 months (91 days) after enrollment to the date of notification of tuberculosis, the date of death, or December 31, 2005.  **Recurrent cases**  82/477 were retreatment cases (no analysis of the risks). | |
| Risk factor: undernutrition | Definition and method for measurement: *(indicate cut-off points for definitions and if the data was self-reported, clinically assessed, etc.)*: BMI was measured by weight and height measured by study staff. BMI for Asian populations: less than 18.5 (underweight), 18.5 to less than 23 (normal), 23 to less than 25 (at risk), 25 to less than 30 (overweight), and 30 or higher (obese).  Timing of measurement: at enrollment  Handling of predictors in the modelling: categorized in 5 BMI categories. | |
| Sample size, missing data and analysis | Number of participants, number of outcomes/events and amount of accumulated person-years (PY): 42,659 people recruited, 42,116 individuals analysed; 477 cases of TB; mean PY: 881 (SD 583) days.  Number of participants, person-years and outcomes/events in relation to the number of candidate predictors (Events Per Variable): 19 confounders for 477 cases (25 TB per confounder)  Number of participants with any missing value (include predictors and outcomes): total of 543 individuals were excl after baseline enrollment.  Number of participants with missing data for each predictor: 304 with missing or invalid identification numbers, 127 with missing or incomplete data on sex, age, weight/height, 89 with TB at enrollment, 23 duplicate entries.  Handling of missing data (e.g., complete-case analysis, imputation, or other methods): excluded from analyses.  Modelling method   - Univariate analysis was first performed to analyze the relationship between BMI and other baseline variables. - The X2 and Fisher exact tests were used as appropriate for categorical variables, and analysis of variance was used for numerical variables. - The incidence of active and culture-confirmed tuberculosis was calculated by assuming a Poisson distribution in the rate of occurrence of the events. - age and sex adjusted Cox proportional hazards analysis of the effects of BMI on the development of active tuberculosis, culture-confirmed tuberculosis, pulmonary tuberculosis, and extrapulmonary tuberculosis (alone) - Multivariable Cox regression adj for effects of potential confounders by entering them together with BMI in the overall Cox proportional hazards model. - Multicollinearity was considered, with analysis repeated after exclusion of individuals with risk factors at baseline. - 2-tailed P<.05 was considered statistically significant. - The attributable risk was derived by applying the adjusted hazard ratios (HRs) to a modified version of the Levin formula11. - Population-attributable risk of a factor = 1 − (rate in absence of factor/observed rate)=1−[1/(HRiPi)], where HRi and Pi are the adjusted HR and prevalence, respectively, of level i of the factor, and HR0 (adjusted HR of reference level) is 1.   Assumptions: not reported.  Adjustment factors used: adjusted for sex, age, smoking, alcohol use, language, marital status, educational level, housing, work status, means-test public assistance status, diabetes mellitus, cholesterol level, cardiovascular disease, hypertension, chronic obstructive pulmonary disease and/or asthma, malignancy, recent weight loss of 5% or more within 6 months, hospital admission within 12 months, and activities of daily living scores. | |
| Results | **Interpretation**  "447 cases of active tuberculosis were analyzed, 326 (68.3%) of which were culture-confirmed. Those individuals who developed active tuberculosis had a significantly greater mean ± SD baseline body height (1.56 ± 0.08 vs 1.54 ± 0.08; P.001) but lower body weight (54.9 ± 10.3 vs 57.6 ± 10.2; P.001) and BMI (22.5 ± 3.7 vs 24.3 ± 3.6; P.001) than those who did not."  **Comparison with other studies**   - (References 12-14) Being underweight has been associated with a higher risk of tuberculosis in several studies. - (References 32 - 34) Low body weight has been associated with the risk of tuberculosis disease, the severity of the disease, and unfavourable response to treatment.   **Discussion of generalizability**  Not reported.  **Strengths and limitations**  Not reported. | |
| Funding | Not available. | |
| Conflict of interests | Not available. | |
| Notes | 15 Feburary 2024 Email: leung@dh.gov.hk - query about adjustment due to immunosupression. | |
| ***Risk of bias*** | | |
| **Bias** | **Authors' judgement** | **Support for judgement** |
| Study participation | Unclear risk | Though it focused on people over 65 enrolled in regional elderly centers. |
| Study Attrition | Low risk | Minimal attrition: 1.3%. |
| Prognostic Factor Measurement | Low risk | Based on clinical records. |
| Outcome Measurement | Low risk | Clearly described data resource and standards were for all participants |
| Study Confounding | Unclear risk | No reports about HIV (few were voluntarily tested), immunosuppression. |
| Statistical Analysis and Reporting | Low risk | Adequately presented Cox regression analysis for the defined model. |

Li 2013

| ***Study characteristics*** | | |
| Study details and sources | **Study design:** prospective cohort study  **Source of data:** clinical examinations  **Study dates:** November 2004 to September 2011  **Setting**: Outpatient  **Countries**: Tanzania | |
| Participants | **Participant eligibility:**  Inclusion criteria: HIV-infected children aged <15 years; being enrolled in the Management and Development for Health pediatric program.  Exclusion criteria: diagnosed with TB at enrollment or within 30 days after enrollment; having unavailable information on TB treatment at enrollment or during follow-up.  Recruitment method: being enrolled in the pediatric Management and Development for Health (MDH) program from 28 clinics in urban Dar es Salaam between November 2004 to September 2011.  Participant Description:   - Age: median age 5 years (IQR 1-9) - Sex: 49% male   Risk factors:   - diabetes: NR - undernutrition:   - wasting (weight-for-length/BMI Z scores): 72% no, 14% moderate, 14% severe   - stunting (height-for-age Z-score): 50% no, 24% moderate, 26% severe   - low mid upper arm circumference (MUAC): 17% yes - HIV infection: 100%   - HIV-associated immunodeficiency: 27% not significant/mild, 36% advanced, 37% severe - recent TB infection: NR - history of untreated or inadequately treated TB disease immunosuppressive therapy: 0% (exclusion criterion) - cigarette smokers: NR - drug or alcohol use disorders: NR - socioeconomic status: NR - other (specify)   - year of enrollment: 2004-2006, 2007-2008, 2009-2011   - season; Apr-May, Oct-Nov, Dec-Mar, Jun-Sep   - district: Ilala, Kinondoni, Temeke   - anemia: yes, no   - WHO Stage: I, II, III, IV   - elevated alanine aminotransferase (ALT): yes, no   - other children in the household: yes, no   - family size: ≤3 family members, >3 family members   - on Cotrimoxazole: yes, no   Details of treatments received (for HIV-infection): a combination of 3 of the following ART medications were prescribed; nevirapine (NVP), lamivudine (3TC), zidovudine (ZDV), stavudine (D4T) efavirenz (EFV). | |
| Outcomes | **Tuberculosis (active tuberculosis or tuberculosis disease)**  Definition: Incident TB, defined as children who were prescribed anti-TB medications during follow-up and who were not diagnosed with TB at enrollment or within 30 days after enrollment.  Method for measurement of outcome: children were screened for tuberculosis based on clinical symptoms, sputum smear (for children 5 years of age or older) or chest X-ray (in younger children). Sputum culture was not available.  Was the same outcome definition (and method for measurement) used in all patients? No further details were provided.  Type of outcome: single endpoint  Time of outcome occurrence or summary of duration of follow-up: time to first TB diagnosis after enrollment; children who did not experience incident TB were censored at death or loss to follow-up.  **TB recurrence (relapse or reinfection)**  not measured | |
| Risk factor: undernutrition | Definition: Wasting, for children under or equal age 2, wasting was measured by weight for length Z-score; for children above age 2, wasting was defined by BMI Z-score. Z-score at least -2 was defined as non-wasting; -3 ≤ Z-score ≤ -2 was defined as moderate wasting; Z-score -3 or less was defined as severe wasting.  Method for measurement: hight and weight were otained by nurses using standardized techniques.  Timing of measurement: at each monthly patient visit forthose receiving ART; for children not receiving ART, visits were scheduled monthly for those aged 5 years of age and below, and at 3-monthly intervals for children above age 5 years.  Handling of predictors in the modelling: categorized in Z scores | |
| Sample size, missing data and analysis | Number of participants: 6,579 children were enrolled, 5,040 were eligible for analysis.  Number of outcomes/events: 376 out of 5,040 childrendeveloped TB.  Amount of accumulated person-years (PY): 7,220.6 person-years of follow-up.  Number of participants, person-years and outcomes/events in relation to the number of candidate predictors (Events Per Variable): 10 confounders for 376 events (37 cases per confounder).  Number of participants with any missing value (include predictors and outcomes): 1,237 participants with missing data at enrollment were excluded (18.8%). Among the 5,040 participants eligible for analysis, there was a moderate amount of missing data (no further details provided).  Number of participants with missing data for each predictor: NR.  Handling of missing data: A missing indicator was created when there were missing values and included in the analysis (no further details provided).  Modelling method:   - The Kaplan–Meier method was used to estimate the incidence of TB after enrollment. - The Andersen-Gill formulation of the Cox proportional hazards model was applied to evaluate the association between each characteristic measured at enrollment and selected time-varying parameters with the time to first TB diagnosis after enrollment. - Multivariate models were fit including all variables that were associated with the primary outcome with P less than 0.20 in the univariate analysis. - The possibility of nonlinear relations between continuous covariates and the risk of incident TB was examined nonparametrically with restricted cubic spline.   Assumptions: not reported.  Adjustment factors used: age, district, year of enrollment, stunting at enrollment, low MUAC, WHO stage at enrollment, immune suppression, anemia, days on ART therapy. | |
| Results | **Interpretation**  Severe wasting was associated with a 1.8 times higher risk of TB than non-wasting in children (RR 1.8, 95%CI 1.3-2.5).  **Comparison with other studies**  Among adults, the triple burden of tuberculosis, HIV infection and malnutrition has been well-documented.  **Discussion of generalizability**  Not reported.  **Strengths and limitations**  *Limitations*: Information on viral load and detailed socio-economic data were not routinely collected in this study. There was a moderate amount of missing data. They relied on the physician's recorded TB treatment as our case definition of TB instead of more standard microbiological confirmation of infection.  *Strengths*: large sample size and relatively long follow-up period. | |
| Funding | This study is supported by U.S. President’s Emergency Plan for AIDS Relief (PEPFAR). Dr Duggan was supported in part by the Eunice Kennedy Shriver National Institute of Child Health and Human Development (NICHD K24HD058795). | |
| Conflict of interests | There were no conflicts of interest reported. | |
| Notes | No email to author. | |
| ***Risk of bias*** | | |
| **Bias** | **Authors' judgement** | **Support for judgement** |
| Study participation | Unclear risk | Focused on HIV-infected children in one region. |
| Study Attrition | Unclear risk | 18.8% attrition, but no detailed descriptions about missing values. |
| Prognostic Factor Measurement | Low risk | Based on clinical records. |
| Outcome Measurement | Unclear risk | Diagnosis of incident TB was not based on culture, but on clinical findings and sputum. |
| Study Confounding | Low risk | Not adjusted by socioeonomic status (children). |
| Statistical Analysis and Reporting | Low risk | Adequately presented Cox regression analysis for the defined model. |

Lin 2018

| ***Study characteristics*** | | |
| Study details and sources | **Study design:** Retrospective cohort studies  **Source of data:** Survey data and insurance claims  **Study dates:** 2005 to 2008  **Setting:** Outpatient  **Countries:** Taiwan, China | |
| Participants | **Participant eligibility:** Adults who took part in a community-based voluntary health screening programm in New Taipei City. Exclusion criteria: having missing covariate data; having a previous history of tuberculosis.  **Recruitment method:** voluntary free screening for chronic diseases and common cancers.  **Participant description:**   - Age: median age 51 years (IQR: 43–59) - Sex: 35.6% male   **Risk factors:**   - diabetes: 9.0% - undernutrition: 2.8% underweight (BMI <18.5 kg/m²) - HIV infection: NR - recent TB infection: 0% (exclusion criterium) - history of untreated or inadequately treated TB disease immunosuppressive therapy: N/A - cigarette smokers: 14.9% current smokers - drug or alcohol use disorders: 7.1% current alcohol use - socioeconomic status (educational level): 20.6% collage or above, 28.4% high school, 51.0% junior high school or below - other (specify)   - marital status: 84.5% married or cohabitating, 5.0% single, 10.5% widowed, divorced, separated or other   - employed: N/A   - low-income household (monthly household income <30000 Taiwan dollars): N/A   **Details of treatments received (for tuberculosis):** NR | |
| Outcomes | **Tuberculosis (active tuberculosis or tuberculosis disease)**  Definition: Incident active tuberculosis.  Method for measurement of outcome: The diagnosis of tuberculosis is based on physical examination, bacteriologic evidence, chest radiography, and response to antituberculosis treatment. Cases of tuberculosis were defined as bacteriologically confirmed cases in the National Tuberculosis Registry.  Was the same outcome definition (and method for measurement) used in all patients? Yes.  Type of outcome: Single endpoint.  Time of outcome occurrence or summary of duration of follow-up: The cohort was followed up until the end of 2013.  **Recurrence of tuberculosis**   Not measured | |
| Risk factor: undernutrition | Definition: Having a BMI <18.5 kg/m² (following the World Health Organization classification).  Timing of measurement: At baseline.  Handling of predictor in the modeling: categorized; underweight (<18.5 kg/m²), normal (≥18.5 kg/m² and <25 kg/m²), overweight (≥25 kg/m² and <30 kg/m²), and obese (≥30 kg/m²). | |
| Sample size, missing data and analysis | **NIHS Cohort** (not included due to overlap; see Supplementary material 7), total number of participants: 48713.  **NTC Cohort**  Number of participants: 119,340 participants.  Number of outcomes/events: 322 cases of incident active tuberculosis.  Amount of accumulated person-years: 868,641 person-years.  Number of participants, person-years and outcomes/events in relation to the number of candidate predictors (Events Per Variable): 322 cases for 7 confounders (46 cases per confounder).  Number of participants with any missing value (include predictors and outcomes): 6,525 participants.  Number of participants with missing data for each predictor: NR.  Handling of missing data (e.g., complete-case analysis, imputation, or other methods): complete-case analysis.  Modelling method:   - Cox proportional hazards regression models to estimate the adjusted hazard ratios (aHRs) of different levels of BMI and corresponding 95% confidence intervals (CIs), using the normal-weight group as the reference. - Causal diagrams to determine which variables to adjust for in the multivariable analysis. - Restricted cubic spline regression to investigate the potential nonlinear relationship between BMI and tuberculosis, usign the likelihood ratio tests as the test for nonlinearity. - Subgroup analysis to estimate effect modification by age, sex, and smoking status. - Causal mediation analysis to characterize the relation between BMI, diabetes, and tuberculosis. Two logistic regression models were constructed, with one regressing the risk of diabetes on BMI levels and the other regressing the risk of tuberculosis on BMI levels and diabetes status. The direct and indirect (mediated through the risk of diabetes) effects of BMI on the risk of tuberculosis were estimated using odds ratios. - Two sensitivity analyses: 1) restricting to culture-confirmed tuberculosis as the outcome, and 2) correcting for self-reporting bias of height and weight.   Assumptions: NR  Adjustment factors used: age (categorical), sex, marital status, education, smoking, alcohol use and diabetes. | |
| Results | **Interpretation**  "During a median of >7 years of follow-up, 491 individuals developed incident tuberculosis. In the causal mediation analysis, being underweight had a harmful effect (direct and indirect effect combined) on tuberculosis (adjusted hazard ratio, 2.28; 95% CI, 3.39–3.73) compared with normal-weight individuals."  **Comparison with other studies**   - "A previous systematic review of 6 cohort studies showed an inverse relationship between body mass index (BMI) and tuberculosis incidence, and the incidence of tuberculosis was lower in the overweight and obese population than in those with normal weight." - "In a prospective cohort study of >42 000 elderly persons in Hong Kong, overweight and obese individuals had a significantly lower risk of tuberculosis than normal-weight persons after adjustment for other tuberculosis risk factors." - "Inverse association between BMI and tuberculosis risk after adjusting for confounders."   **Discussion of generalizability**  Not reported  **Strengths and limitations**  *Limitations:*   - BMI was used to measure adiposity in the body. BMI cannot distinguish between weight and fat and weight from muscle and bone. - No information was provided on latent tuberculosis infection among the study participants. As a result, the authors could not determine whether the association between BMI and active tuberculosis was due to the increased risk of tuberculosis infection, the increased risk of disease progression from latent infection, or both. - There was no adjustment for human immunodeficiency virus (HIV) status in the analysis, but there is a very low prevalence (<1 in 1000) of HIV in Taiwan.   *Strengths*: NR | |
| Funding | This work was supported by the Taiwan Ministry of Science and Technology (grant MOST105-2628-B-002-025-MY3). The funder had no role in the study design, data collection, data analysis, data interpretation, or report writing. | |
| Conflict of interests | The authors reported no conflicts of interest. | |
| Notes | 13 February 2024 Email at hsienho@ntu.edu.tw requesting data on missing data, outcome measure and adjusted analyses.  14 February 2024 The author provided information on all questions (changed our risk of bias assessment). | |
| ***Risk of bias*** | | |
| **Bias** | **Authors' judgement** | **Support for judgement** |
| Study participation | Low risk | Large database of adults, adequately described. |
| Study Attrition | Unclear risk | Total attrition 5.2% (4.22% due to missing covariate data). No further analysis were considered. |
| Prognostic Factor Measurement | Low risk | BMI was measured according to clinical standards for all patients |
| Outcome Measurement | Low risk | The author provided information: 79.5% were bacteriologically confirmed cases. |
| Study Confounding | Unclear risk | Analyses were not adjusted for immunosuppresive status (HIV). |
| Statistical Analysis and Reporting | Low risk | Adequate presentation of logistic regession analyses (mediation analysis) for the defined model. |

Liu 2015

| ***Study characteristics*** | | |
| Study details and sources | **Study design:** Prospective cohort study  **Source of data:** Survey and medical examinations  **Study dates**: November 2004 - December 2012  **Setting:** Outpatient  **Countries:** Tanzania | |
| Participants | **Participant eligibility**  Inclusion criteria: >= 15 years of age, HIV-infected and attending an HIV clinic in Dar es Salaam.  Exclusion criteria: previously having had ART (n = 8912), being diagnosed with TB within 30 days after enrolment (n = 9711), having an unrecorded TB status within 30 days after enrolment (n = 6680), not having any follow-up visits (n = 15 565).  Recruitment method:HIV-infected individuals enrolled in one of the 50 Management and Development for Health (MDH)-)-supported HIV care and treatment clinics in three municipalities of Dar es Salaam (Temeke, Ilala and Kinondoni).  Participant description:   - Age: mean 35.6 years (SD 9.7) - Sex: Male 25%   Risk factors:   - diabetes: NR - undernutrition:   - 10.2% BMI <17 kg/m²; 10.9% BMI 17 to <18.5 kg/m² - HIV infection: 100%   - ART initiation: 78.4%   - CD4+ cell count (cells/uL): 15.6% <50; 11.4% 50 to <100; 20.6% 100 to <200; 52.4% 200 cells/ml   - WHO Stage: 26.6% I; 22.6% II; 38.0% III; 12.8% IV - recent TB infection: 0% (exclusion criterium) - history of untreated or inadequately treated TB disease: NR - immunosuppressive therapy: NR - cigarette smokers: NR - drug or alcohol use disorders: NR - socioeconomic status: Education: NR - other (specify)   - anemia: none, mild, moderate, severe     - 12.7% severe anemia, 44.8% moderate anemia, 21.0% mild anemia, 21.4% no anemia   - Alanine aminotransferase >40 U/l: 11.3%   - Cotrimoxazole use: 33.2%   - Isoniazid (INH) preventive therapy: no, yes   - Family size > 3 members: 14.6%   Details of treatments received (treatment for tuberculosis and other concomitant conditions):  HIV: Antiretroviral treatment for those who were eligible to initiate. Standard first-line ART regimens included two nucleoside reverse-transcriptase inhibitors (NRTIs) [lamivudine (3TC) or emtricitabine (FTC), as well as stavudine (d4T) or zidovudine (ZDV) or Tenofovir (TDF)], and one nonnucleoside reverse-transcriptase inhibitor (NNRTI) [efavirenz (EFV) or nevirapine (NVP)].  Tuberculosis: Rifampin therapy. | |
| Outcomes | **Tuberculosis (active tuberculosis or tuberculosis disease)**  Definition: Incident TB cases were defined as patients who were free of TB at enrolment and newly diagnosed with TB during the study period.  Method for measurement of outcome: At each clinic visit, patients were screened for TB on the basis of clinical symptoms, including cough, fever, night sweats, weight loss and haemoptysis. Sputum smears and chest radiographs were administered to all TB-suspected patients to assist with diagnosis. Another diagnostic criterium was being prescribed anti-TB medications during follow-up. Sputum cultures were not available in the HIV care and treatment clinics in Dar es Salaam  Was the same outcome definition (and method for measurement) used in all patients? No, the majority of TB diagnosis were based on clinical syndromes. 12% had a sputum-based diagnosis, 7% a chest X-ray-based diagnosis.  Type of outcome: single endpoint.  Time of outcome occurrence or summary of duration of follow-up: Time to first TB diagnosis after enrollment; Participants who did not experience incident TB were censored at death or loss to follow-up or the last visit date by 30 September 2012.  **Recurrence of tuberculosis**   Not measured | |
| Risk factor: undernutrition | Definition: BMI <18.5,  Method for measurement: BMI was calculated as weight in kilograms divided by the square of the height in meters.  Timing of measurement: at enrollment.  Handling of predictors in the modeling: categorized; <17, 17 to <18.5, 18.5 to <25, 25 to <30, 30 kg/m². | |
| Sample size, missing data and analysis | Number of participants: 108,554 initially enrolled, 83,251 included in the initial sample, 67,686 participants included in the final sample (with follow-up data)  Number of outcomes/events: 7,602 participants were diagnosed with active TB.  Amount of accumulated person-years (PY): NR.  Number of participants, person-years and outcomes/events in relation to the number of candidate predictors (Events Per Variable): 7602 cases of TB per 15 confouders (50 cases per confounder).  Number of participants with any missing value (include predictors and outcomes): NR.  Number of participants with missing data for each predictor: NR.  Handling of missing data: Missing indicator variables were created for covariates with incomplete information (no further details reported).  Modelling method:   - The Andersen–Gill formulation of the Cox proportional hazard model was applied to evaluate the associations of each baseline characteristic measured at enrolment and selected time-varying parameters with time to first TB event after enrolment - Multivariate models were fit including all variables that were associated with the primary outcome with P value less than 0.20 in the univariate analysis. - Likelihood ratio tests were used to calculate the P values for nominal variables, including district, season and calendar months. - The possibility of nonlinear relations between continuous covariates and the risk of incident TB was examined nonparametrically with restricted cubic splines.   Assumptions: NR.  Adjustment factors used: age, sex, district, family size, year of enrollment, season of visit (time-varying), BMI (time-varying), Middle Upper Arm Circumference (time-varying), Anemia status (time-varying), CD4+ count (time-varying), WHO HIV disease stage (time-varying), ALT (time-varying), Cotrimoxazole (time-varying), Isoniazid (time-varying), months on ART (time-varying). | |
| Results | **Interpretation**  Time-varying nutritional status was significantly associated with TB risk. Compared with patients with normal BMI (18.5–<25.0kg/m2), patients with BMI<17.0 had a 2-fold increased risk of TB, while overweight and obese patients had 36% and 45% per cent reduced risks, respectively (P value for trend<0.001).  **Comparison with other studies**   - The TB incidence pattern following ART initiation in the current study is consistent with findings from previous studies. - The bidirectional association between malnutrition and TB has been well documented in the general population. - Several studies have indicated that poor nutritional status is associated with higher risks of mortality and opportunistic infection, including TB, after ART initiation.   **Discussion of generalizability**  Not reported.  **Strengths and limitations**  *Limitations*   - The majority of the TB diagnoses were based on clinical syndromes. Sputum cultures were not available in the HIV care and treatment clinics in Dar es Salaam. Because of the high TB incidence among the target population, sputum microbiological confirmation was not required for the diagnosis and treatment of TB for patients attending the HIV clinics. - As viral loads were not routinely measured in the study population, socio-economic data, and other lifestyle factors, including smoking and diet, were not collected, they were not able to take them into account, and potential residual confounding from these factors cannot be excluded.   *Strengths*:   - The large sample size and relatively long follow-up period. - More than 75% of the patients started ART during follow-up and contributed data and person-years in pre- and post-ART periods. This allows the authors to calculate the TB incidence by ART status and examine the short-term and long-term effects of ART on incidence. | |
| Funding | The HIV care and treatment programme was supported by U.S. Presidents’ Emergency Plan for AIDS Relief (PEPFAR, grant number U51HA02522), and the Centers for Disease Control and Prevention (grant number 5U2GPS001966). The contents of this manuscript are solely the responsibility of the authors and do not necessarily represent the official views of the Centers for Disease Control and Prevention or the Department of Health and Human Services. | |
| Conflict of interests | There are no conflicts of interest. | |
| Notes | No email to author (high risk of bias). | |
| ***Risk of bias*** | | |
| **Bias** | **Authors' judgement** | **Support for judgement** |
| Study participation | Unclear risk | HIV-population in one city; important baseline information unavailable |
| Study Attrition | Unclear risk | 14.3% attrition due to loss to follow-up, but no details described. Missing indicator variables were created for covariates with incomplete information. |
| Prognostic Factor Measurement | Low risk | Based on clinical records. |
| Outcome Measurement | High risk | Diagnosis of incident TB was mostly based on clinical evaluation. Culture-confirmed diagnoses were not available. |
| Study Confounding | Unclear risk | No information available on diabetes, history of TB, immunosuppression, alcohol use, smoking, socioeconomic status. |
| Statistical Analysis and Reporting | Low risk | Adequately presented Cox regression analysis for the defined model. |

Long 2020

| ***Study characteristics*** | | |
| Study details and sources | **Study design:** prospective cohort study  **Source of data**: clinical examinations  **Study dates**: October 2012 - 2018  **Setting**: Outpatient  **Countries**: China | |
| Participants | **Participant eligibility:**  Inclusion criteria: Adults and children with rheumatic diseases treated with glucocorticoid for at least 4 weeks from October 2012 to May 2016; informed consent given.  Exclusion criteria: having possible active tuberculosis; being treated with TNF-a and non-TNF-a biological agents; Refusal of the T-SPOT TB-test.  Recruitment method: Patients were recruited at the Nanchang University Hospital in China; no further details were provided.  **Participant Description:**  Reported for participants with (23.6%) and without (76.4%) a latent TB infection, resp.   - Age: mean age 46.5 years (SD 25.2); mean age 42.8 years (SD 17.6) - Sex: 28% male; 24.2&% male   **Risk factors:**  Reported for participants with a (23.6%) and without (76.4%) a latent TB infection, resp.   - diabetes: 26.6%; 16.9% - undernutrition: 9.4% BMI <18.5 kg/m²; 12.8% BMI <18.5 kg/m² - HIV infection: NR - active TB infection: 0& (exclusion criterion) - history of untreated or inadequately treated TB disease: NR - immunosuppressive therapy: 72.2%; 86.2% - cigarette smokers: NR - drug or alcohol use disorders: NR - socioeconomic status: NR - other (specify)   - Rheumatic disease duration: 67.5 months (SD 79.3); 71.8 months (SD 54.2)   - Interstitial Lung Disease: 10.0%; 12.0%   - Chronicb Renal Failure: 6.8%; 6.0%   - Silicosis: 0.86%; 0.97%   - Cancer: 1.72%, 2.0%   Details of treatments received: Glucocorticoids (prednisone), Immunosuppressant agents included Azathioprine (AZA), Cyclosporin A (CsA), Cyclophosphamide (CTX,), Tacrolimus (FK-506), Hydroxychloroquine (HCQ), Mycophenolate mofetil (MMF), Methotrexate (MTX) and Thalidomide (ThD). | |
| Outcomes | **Tuberculosis (active tuberculosis or tuberculosis disease)**  Definition: activation of latent TB infection (A latent tuberculosis infection (LTBI) was defined as a state of immune response to stimulation by antigens of Mycobacterium tuberculosis without evidence of active tuberculosis).  Method for measurement of outcome: The criteria for active tuberculosis were cough with or without sputum production, haemoptysis, fever, night sweat, unexplained weight loss, abnormal chest X-ray, signs of extrapulmonary tuberculosis and a definitive diagnosis was established by isolation of M. tuberculosis from a bodily secretion (e.g. culture of sputum, bronchoalveolar lavage or pleural fluid) or tissue (pleural biopsy or lung biopsy) and resulting positive cultures.  Was the same outcome definition (and method for measurement) used in all patients? yes  Type of outcome: single endpoint  Time of outcome occurrence or summary of duration of follow-up: Latent TB infection was measured at baseline. Occurence of active TB was established during a two-year follow-up period had occurred by telephone or in-person interviews regularly every 3 months.  **TB recurrence (relapse or reinfection)**  not measured | |
| Risk factor: undernutrition | Definition: BMI <18.5 kg/m²  Method for measurement: NR, likely taken from clinical records  Timing of measurement: at baseline  Handling of predictors in the modeling: categorical; BMI <18.5 kg/m² versus BMI ≥18.5 kg/m² | |
| Sample size, missing data and analysis | Number of participants: 349 participants had a latent TB infection and were considered for analysis (1,788 participants with rheumatic disease and receiving glucocorticoid treatment were screened with interferon-release assays)  Number of outcomes/events: 18 cases of active TB among those with a latent TB infection.  Amount of accumulated person-years (PY): N/A  Number of participants, person-years and outcomes/events in relation to the number of candidate predictors (Events Per Variable): 18 cases for 5 confounders (3 cases per confounder).  Number of participants with any missing value (include predictors and outcomes): 183 patients were excluded due to possible active TB at enrollment, 5 patients refused testing for latent TB infection, 121 had indeterminate test results for a latent TB infection, 11 patients died and 6 were lost to follow-up.  Number of participants with missing data for each predictor: NR.  Handling of missing data: participants with missing data were excluded from the analysis.  Modelling method:   - Logistic regression models were used for univariate and multivariate regression analyses to calculate odds ratios for various factors and the correlation with the incidence of activation of LTBI.   Assumptions: not reported.  Adjustment factors used: age, high dose glucocorticoid therapy, interstitial lung disease, chronic renal failure and cancer. | |
| Results | **Interpretation**  The incidence density of TB in the patients with LTBI was 15 times higher than in the LTBI-negative group. Low BMI (OR 1.83; 95% CI, 1.61–2.82), high-dose glucocorticoid therapy (not low dose of 15 mg/d), and comorbidities that include interstitial lung disease and malignant tumors were associated with increased incidence of activation of LTBI.  **Comparison with other studies**  Not reported.  **Discussion of generalizability**  Not reported.  **Strengths and limitations**  *Limitations*:   - There is no ‘gold standard’ test for diagnosing LTBI. The T-SPOTV-TB assay used in this study offers a distinct advantage over the tuberculin skin test because BCG vaccination status does not affect it. However, the immunosuppression associated with rheumatic diseases may lead to false negative or inconclusive results due to decreased peripheral blood mononuclear cell count. - Although 1600 patients were recruited, and follow-up was for 2 years, the number of cases of active TB in the cohort was limited.   *Strengths*: NR. | |
| Funding | NR. | |
| Conflict of interests | No potential conflict of interest was reported by the authors. | |
| Notes | 13 February 2024 Email to tcmclinic@163.com requesting data on adjustment and missing data. | |
| ***Risk of bias*** | | |
| **Bias** | **Authors' judgement** | **Support for judgement** |
| Study participation | Unclear risk | Focused on patients with rheumatic diseases in one local hospital; important baseline information unavailable. |
| Study Attrition | Unclear risk | 18.2% attrition due to possible active TB, refusal of TB testing, inconclusive TB results, death, and loss to follow-up, but no more details were described. |
| Prognostic Factor Measurement | Low risk | Based on clinical records |
| Outcome Measurement | Low risk | TB diagnosis was defined by positive culture results and sample origins were clarified. |
| Study Confounding | Unclear risk | No information available about HIV, history of TB, immunosuppression, alcohol use, smoking, socioeconomic status |
| Statistical Analysis and Reporting | Unclear risk | Description of statistical model and selection of confounders specified; no subgroup or sensititivity analyses undertaken |

Maro 2010

| ***Study characteristics*** | | |
| Study details and sources | **Study design:** prospective cohort study  **Source of data:** medical examination  **Study dates:** not mentioned.  **Setting:** Outpatient  **Countries:** Tanzania | |
| Participants | **Participant eligibility**  Subjects were enrolled if they were aged ⩾18 years, had HIV infection, baseline CD4 counts of ⩾200 cells/μl and a scar from childhood Bacille Calmette-Guérin (BCG) immunization. *Exclusion*: pregnant women and subjects with active TB detected at baseline via clinical evaluation or cultures of sputum and blood.  **Recruitment method**  Subjects were placebo recipients in the DarDar Health Study, a 7-year Phase III randomized placebo-controlled trial of a novel TB booster vaccine in Dar es Salaam, Tanzania.  **Participant Description:**   - Age: ⩾18 years of age, mean age 33.7 years - Sex: Male 26.5%, Female 73.5%   **Risk factors:**   - diabetes: NR - undernutrition: BMI < 17 kg/m2 - HIV infection: all subjects had HIV - recent TB infection: previous TB treatment (%) - history of untreated or inadequately treated TB disease: NR - immunosuppressive therapy: % - cigarette smokers: NR - drug or alcohol use disorders: NR - socioeconomic status: NR - other (specify)   - CD4 count, cells/μl   - TST, mean, mm   - TST ⩾ 5 mm, %   Details of treatments received (treatment for diabetes, undernutrition and other concomitant conditions): Placebo TB vaccine; some subjects received HAART (highly active antiretroviral therapy). | |
| Outcomes | **Tuberculosis (active tuberculosis or tuberculosis disease)**  Definition and method for measurement of outcome: TB cases were defined by a blinded three-person expert panel according to the pre-defined and published study definitions detailed in Table 1 (below).  Definite TB   - 1. One or more sputum cultures positive for *Mycobacterium tuberculosis* with ⩾10 cfu; or, - 2. Two or more sputum cultures with 1–9 cfu of *M. tuberculosis* (indeterminate *M. tuberculosis* culture); or, - 3. Two or more positive sputum smears for AFB; or, - 4. One or more cultures for *M. tuberculosis* from the blood or other sterile body site.   Probable TB   - 5. Positive chest X-ray plus eitheri) one positive sputum AFB smear, or,ii) one indeterminate *M. tuberculosis* culture result; or, - 6. Clinical symptoms/signs plus eitheri) one positive sputum AFB smear, or,ii) an indeterminate *M. tuberculosis* culture result; or, - 7. Clinical symptoms/signs and a positive X-ray plus a response to anti-tuberculosis therapy; or, - 8. One positive sputum AFB smear from a sterile site plus clinical symptoms/signs of TB; or, - 9. Caseous necrosis on a tissue biopsy.   The evaluation for TB involved physical examination, a single view chest X-ray, three sputum collections for acid- fast bacilli smear and culture and blood culture for *Mycobacterium tuberculosis*.  At baseline all subjects had a tuberculin skin test (TST) with 0.1 ml intradermal purified protein derivative. Subjects with ⩾5 mm induration at 48–72 h were considered to have a positive TST and were offered a 6-month course of isoniazid (INH), with an 88% completion rate among the placebo recipient subset.  Was the same outcome definition (and method for measurement) used in all patients? Yes  Type of outcome: single endpoints - TB incidence  Time of outcome occurrence or summary of duration of follow-up: median follow up of 2.3 years.  **TB recurrence (relapse or reinfection)**  Not mentioned, only the % subjects with previous TB treatment. | |
| Risk factor: undernutrition | Definition and method for measurement:BMI was calculated with height and weight. The standard equations to calculate BMI, and categorized BMI results by quartiles.  Timing of measurement: at baseline and at a second visit (‘Year 1 visit’) an average of 471 days (standard deviation 130 days) after the baseline visit  Handling of predictors in the modelling: Categorized baseline BMI, Year 1 BMI, and the mean change in BMI from baseline to Year 1 by quartiles. | |
| Sample size, missing data and analysis | Number of participants, number of outcomes/events and amount of accumulated person-years (PY): 92 cases of TB among 979 subjects followed for a mean of 3.2 years.  Number of participants, person-years and outcomes/events in relation to the number of candidate predictors (Events Per Variable): 92 cases for 3 confounders (30.67 cases per confounder)  Number of participants with any missing value (including predictors and outcomes): not reported  Number of participants with missing data for each predictor: not reported  Handling of missing data (e.g., complete-case analysis, imputation, or other methods): not reported  Modelling method   - The authors used the Mann-Whitney U-test for univariate comparisons of BMI and clinical characteristics between subjects who did or did not develop TB during prospective follow-up. - For analyses of the relation of TB risk with the Year 1 BMI or the change in BMI from baseline to Year 1, the authors excluded data from subjects who developed TB before the Year 1 BMI measurement. - To determine if BMI was independently associated with the risk of developing HIV-associated TB, the authors conducted a multivariate Cox proportional hazards regression model adjusting for clinical characteristics that differed at baseline between subjects who did or did not develop TB. - The authors confirmed that the proportional hazards assumption was not violated using log-log plots and examined if BMI variables had a linear effect on the log hazard using penalized splines, and no significant departure was found. - All statistical analyses were conducted using STATA 9 (Stata Corp, College Station, TX, USA).   Assumptions: Low BMI among the subject population is predominantly driven by malnutrition.  CD4 count, TST status, and previous TB treatment history were adjusted factors. | |
| Results | **Interpretation**  There was a higher incidence of TB among subjects with a lower baseline BMI, a lower Year 1 BMI, and a greater BMI decrease from baseline to Year 1.  Subjects with a baseline BMI < 17 kg/m2 had a high incidence of subsequent TB disease compared to subjects with a higher baseline BMI (27.3% vs. 9.2%, *P* = 0.0411).  BMI < 17 kg/m2 and a decrease in BMI of ⩾0.5 kg/m2 were associated with increased risk of TB in univariate and multivariate models  **Comparison with other studies**   - [References 12, 13, 14] Malnutrition enhances susceptibility to infection, and in subjects without HIV infection, low BMI is a risk factor for the development of tuberculosis (TB). - [References 15-18] Among patients with HIV, TB disease often arises during malnutrition and even exacerbates it. - [References 19, 20] Malnutrition is associated with more severe pulmonary manifestations of TB disease and poorer outcomes after TB therapy. - [References 21, 22, 23] TB is a leading cause of death among people with HIV and malnutrition is associated with poor clinical outcomes in subjects with HIV. - [References 28] Low BMI is related to a greater bacillary burden during HIV-associated TB.   **Discussion of generalizability**  Not mentioned.  **Strengths and limitations**  *Strengths*:   - The size, prospective nature, and rigorous and predefined case definitions used for TB diagnoses.   *Limitations*: none specifically reported. | |
| Funding | National Institutes of Health (NIH), Division of Ac- quired Immunodeficiency Syndrome, AI 45407 (CFR) and Fogarty International Center, D43-TW006807 (IM, CFR); NIH, National Center for Research Resources, Centers of Biomedical Research Excellence 5P20RR016437-08 (TL). | |
| Conflict of interests | Not available. | |
| Notes | No email to authors. | |
| ***Risk of bias*** | | |
| **Bias** | **Authors' judgement** | **Support for judgement** |
| Study participation | Unclear risk | Focused on HIV-infected patients involved in one RCT trial; important baseline information not available |
| Study Attrition | Low risk | No attrition |
| Prognostic Factor Measurement | Low risk | Based on clinical records. |
| Outcome Measurement | Low risk | Adequately measured by clinical experts and based on culture. |
| Study Confounding | Unclear risk | No information available about DM, history of TB, alcohol use, smoking, socioeconomic status |
| Statistical Analysis and Reporting | Unclear risk | Description of statistical model and selection of confounders specified; no subgroup or sensititivity analyses undertaken |

Moore 2007

| ***Study characteristics*** | | |
| Study details and sources | **Study design:** prospective cohort study  **Source of data:** medical examination  **Study dates:** 1 May 2003 and 30 June 2005  **Setting:** Outpatient  **Countries:** Uganda | |
| Participants | **Participant eligibility:** Adult patients with AIDS who are eligible for ART.  Recruitment method: Patients were recruited either from the Home-based AIDS Care Project (HBAC): a clinical trial of three different monitoring strategies for patients receiving ART in Tororo district, a rural area in eastern Uganda, or patients registered in the AIDS Support Organization (TASO): a local HIV/AIDS care and support organization in Tororo and Busia districts. Patients from TASO were invited to be screened for ART eligibility. The study also included participants from a prior diarrhoea prevention and co-trimoxazole study described elsewhere (10), as well as newly recruited clients.  **Participant Description:**   - Age: median age 37 - Sex: Female 72.5%, Male 27.5%   **Risk factors:**   - diabetes: NR - undernutrition: Percentage with body mass index <= 18 - HIV infection: RNA > 100000 copies/ml - recent TB infection   - TB at baseline (%)   - Prior TB treatment (%) - history of untreated or inadequately treated TB disease: NR - immunosuppressive therapy: all patients - cigarette smokers: NR - drug or alcohol use disorders: NR - socioeconomic status: NR - other (specify)   - Percentage by baseline CD4 cell count (cells/ul): <50, 50–199, >200   - Participant in previous safe water/co-trimoxazole study   Details of treatments received (treatment for diabetes, undernutrition and other concomitant conditions):  Treatment for HIV: ART= with nevirapine, stavudine and lamivudine as the standard regimen. All ART-eligible participants and HIV-infected household members were prescribed daily co-trimoxazole therapy. Participants diagnosed with TB were provided with home-based TB treatment. | |
| Outcomes | **Tuberculosis (active tuberculosis or tuberculosis disease)**  Definition and method for measurement of outcome: Diagnostic methods were restricted to sputum smears, radiographs, and clinical judgment.   - Patients with symptoms of pulmonary TB were required to have two positive sputum smears results for acid-fast bacilli to be diagnosed with TB. Those without two positive smears underwent chest radiography and were given a 2-week course of broad-spectrum antibiotics. If they were still symptomatic after this and their chest radiographs were compatible with TB, they were then diagnosed with smear-negative pulmonary TB. The diagnosis of extrapulmonary TB was based on symptomatology and clinical presentation.   Was the same outcome definition (and method for measurement) used in all patients? Same outcome: Incident of TB, but different methods of measurement.  Type of outcome: single endpoints  Time of outcome occurrence or summary of duration of follow-up: Median follow-up was 1.4 years. Participants were screened for active TB at baseline and then monitored at weekly home visits.  **TB recurrence (relapse or reinfection)**  Not measured | |
| Risk factor: undernutrition | Definition and method for measurement: Body mass index <= 18 kg/m2  Timing of measurement: at baseline  Handling of predictors in the modeling: one variable | |
| Sample size, missing data and analysis | Number of participants, number of outcomes/events and amount of accumulated person-years (PY): from a total of 1,044 participants, 53 (5.5%)were diagnosed with TB over a median of 1.4 years of follow-up (3.90 cases/100 person-years)  Number of participants, person-years and outcomes/events in relation to the number of candidate predictors (Events Per Variable): 53 cases in 4 confounders (13.25 cases per confounder).  Number of participants with any missing value (include predictors and outcomes): 28 (37%) were not classified because of missing data, largely within the group that were diagnosed with TB prior to study initiation. One subject died with an incomplete TB assessment. Two subjects (4%) were not classified because of missing data from the newly TB cases.  Number of participants with missing data for each predictor: 28 with baseline TB, 1 dead, 2 with newly diagnosed TB.  Handling of missing data (e.g., complete-case analysis, imputation, or other methods): excluded in the analysis.  Modelling method:   - Participants taking TB treatment or diagnosed with TB at ART-eligibility screening were compared with those without TB using multivariate logistic regression analysis to examine which baseline factors were independently associated with having TB. - Incidence rates of TB diagnoses and mortality rates were calculated using Kaplan – Meier methods and Cox proportional hazards modeling was used to examine associations between baseline variables and TB incidence, and between baseline variables, variables associated with TB diagnosis, and treatment, and mortality. - The effect of IRIS on TB- associated mortality was approximated by examining associations of the time at which TB was diagnosed in relation to ART start date, with diagnoses occurring within the first 3 months of ART initiation used as a proxy measure for IRIS. - The estimated effectiveness of ART in reducing TB incidence and associated mortality was calculated using the formula: 1–(observed/expected) both for the total follow-up time and for time periods that excluded the first 6 months of ART, when clinical responses to therapy are still incomplete. - All statistical analyses were conducted in SAS version 9.0 (SAS Institute, Cary, North Carolina, USA).   Assumptions:   - The expected number of TB cases was calculated by applying the incidence rate of 5.28/100 person-years from an observational study of HIV-infected individuals with WHO clinical stages III or IV or CD4 cell counts < 250 cells/ul in rural Masaka district Uganda from 1990 to 2006 (L. VanderPaal, personal communication) to the study population, multiplied by the number of person-years of follow-up time from the current study. - The expected number of TB deaths in follow-up was calculated by applying a mortality rate of 36% to the expected number of incident TB cases above. This is a published 1-year mortality rate for HIV-infected individuals with CD4 cell cell count < 200 cells/ul diagnosed with active TB at Mulago Hospital in Kampala.   Adjustment factors used: body mass index (BMI), age, having a prior history of TB treatment, being a participant in previous co-trimoxazole/safe water vessel study. | |
| Results | **Interpretation**  A total of 53 subjects were diagnosed with new TB events over a median of 1.4 years of follow-up, for a cumulative incidence rate of 3.90/100 person-years. TB incidence was associated with a BMI <= 18 at baseline (RH, 2.80; 95% CI, 1.59 – 4.92) and marginally associated with a prior history of TB treatment (RH, 1.74; P = 0.07).  **Comparison with other studies**   - [References 6] Antiretroviral therapy (ART) in Africa is likely to significantly reduce the incidence and mortality of TB in HIV-infected individuals in sub-Saharan Africa. - Previous studies conducted in Rio de Janeiro, Brazil [Reference 12] and Cape Town, South Africa [Reference 6] have estimated an 80% reduction of TB incidence for HIV-infected adults associated with the use of ART.   **Discussion of generalizability**  Not reported  **Strengths and limitations.**  *Limitations*:   - There is a lack of directly comparable data on TB incidence and mortality from ART-eligible individuals not receiving ART in Uganda. - It is possible that some of the analyses, such as those comparing mortality associated with TB diagnoses within three months of initiating ART or mortality associated with the type of TB, lacked statistical power to show differences.   *Strengths*: none reported | |
| Funding | There is no mention of the funding source | |
| Conflict of interests | Not available. | |
| Notes | No email to author. | |
| ***Risk of bias*** | | |
| **Bias** | **Authors' judgement** | **Support for judgement** |
| Study participation | Unclear risk | Participants recruited from previous studies and a registry; important baseline information not available |
| Study Attrition | Low risk | Minimal attrition (3%) |
| Prognostic Factor Measurement | Low risk | Based on clinical records. |
| Outcome Measurement | Unclear risk | TB diagnosis was based on smear results. The proportion of negative smear results was high (43%). Culture-confirmed diagnoses not available. |
| Study Confounding | Unclear risk | No information available about DM, history of TB, immunosuppression, alcohol use, smoking, socioeconomic status |
| Statistical Analysis and Reporting | Unclear risk | Description of statistical model and selection of confounders specified; no subgroup or sensititivity analyses undertaken |

Morán-Mendoza 2010

| ***Study characteristics*** | | |
| Study details and sources | **Study design:** retrospective cohort study  **Source of data:** Close contacts program  **Study dates:** 1990 to 2000  **Setting:** outpatient  **Countries:** Canada | |
| Participants | **Participant eligibility**  Contacts of active cases recorded in the Division of TB Control at the British Columbia Centre for Disease Control (BCCDC)  **Recruitment method:**  *Probably consecutive*  **Participant description:**   - Age: *0-10 8.2%, >10 91.8%, median 35 (range 0 to 107)* - Sex: *59% female*   **Risk factors:**   - diabetes 1.9% - undernutrition 0.1% - HIV infection: excluded - recent TB infection: Chest X-ray compatible with previous TB 0.7%, TB infection treatment 4.8% - history of untreated or inadequately treated TB disease: not reported: previous active TB excluded, but TB infection with incomplete treatment 3% - immunosuppressive therapy: corticosteroids 0.4% and other co-morbidities described elsewhere - cigarette smokers: not reported - drug or alcohol use disorders: alcoholism 0.9% - socioeconomic status: 1st quintile 24.5%, 2nd 21.7%, 3rd 19.5%, 4th 18.3%, 5th 16% - others:   - Malignancy 1.5%   - Renal failure 0.3%   - Intravenous drug use 0.4%   - Ethnicity: foreign-born 34.4%, aboriginal 10.3%   - A recent arrival from high prevalence TB countries 4%   - Employed in high-risk settings 4.8%   - Hospital personnel 14.3%   - Previous BCG vaccination 20.3%   Details of treatments received (treatment for diabetes, undernutrition and other concomitant conditions): not specified | |
| Outcomes | **Tuberculosis (active tuberculosis or tuberculosis disease)**  *Definition and method for measurement of outcome:* 1) smear and/or culture positive for tubercle bacilli, 2) histopathological diagnosis or 3) clinical and radiological diagnosis of active TB, with complete treatment response, when smears and cultures were negative.  Was the same outcome definition (and method for measurement) used in all patients? Yes  Type of outcome: single  **Time of outcome occurrence or summary of duration of follow-up:**  Median follow-up 6 years (range <1-12)  **TB recurrence (relapse or reinfection)**  Not measured | |
| Risk factor: undernutrition | Definition and method for measurement: diagnosed by nurse or physician for all participants  Timing of measurement: at presentation from the database.  Handling of predictors in the modelling: dichotomous. | |
| Sample size, missing data and analysis | Number of participants, number of outcomes/events and amount of accumulated person-years (PY): 42,593 total number of close contacts.  Incidence of 668 per 100,000 population, 74% within the first year, 86% during the second year  Number of participants, person-years and outcomes/events in relation to the number of candidate predictors (Events Per Variable):  624/100,000 participants with diabetes  14706/100,000 participants with malnutrition  Number of participants with any missing value (include predictors and outcomes): 19.8% of the initial sample of 42,593 had missing risk factor data  Number of participants with missing data for each predictor: not available  Handling of missing data (e.g., complete-case analysis, imputation, or other methods): complete case analysis  Modelling method: Cox regression with robust variance estimation.  Assumptions:first univariate analysis, then all risk factors, and then forward and backward stepwise selection methods. Significant variables in all models and potential confounders were included in the final model.  Adjustment factors used: diabetes, malignancy, corticosteroids, alcoholism, malnutrition, TB infection treatment, age, closeness of contact, tuberculin test size, intravenous drug use, infectivity of source, ethnicity, socioeconomic status, sex, arrival from high prevalence country, previous BCG vaccination. | |
| Results | **Interpretation**  “Once adjusted for other risk factors, having diabetes mellitus, renal failure and a chest X-ray compatible with previous TB did not significantly increase the risk of developing TB.” “Malnutrition was the most important risk factor in our study, and likely refl ects contacts with severe malnutrition.”  **Comparison with other studies**  “although they have been recognised as risk factors in concerned populations. [refs 9,11,26]”  **Discussion of generalizability**  “In Canada, TST sizes of ⩾5 mm are considered significant in close contact of active TB cases and indicate the need for treatment; however, at the time of this study, LTBI treatment was not recommended in subjects aged >35 years if they were considered at low risk for TB.”  **Strengths and limitations:**  1) Lack of BMI data, 2) Few cases in some risk categories (imprecision), 3) Missing data or errors when cross-linking information across databases. | |
| Funding | Not available | |
| Conflict of interests | Not available | |
| Notes | 25 August 2023 Email: morano@queensu.ca  What were the definitions for diabetes and undernutrition? How many of the TB cases were not confirmed with culture? 25 August 2023 - Response that information will be retrieved. 26 August 2023 - “-Diagnosis of DM and malnutrition: the presence of diabetes mellitus, chronic renal failure, any malignancy, aplastic anemia, malnutrition was considered as present/positive if reported in the databases, which depends on the clinical diagnosis of the physician or public health nurse. -How many of the TB cases were not confirmed by culture: among the 33,146 contacts of interest, there were 228 who developed TB during the 12-year follow-up period. Among these, 120 TB cases had a negative smear or culture (52.6%) and 108 (47.4%) had a positive smear and/or culture: 49 of them had a positive smear (21.5%) and 103 had a positive culture for MTB (45.2%).” | |
| ***Risk of bias*** | | |
| **Bias** | **Authors' judgement** | **Support for judgement** |
| Study participation | Unclear risk | Although the population is well described it represents partially the target population of household contacts. |
| Study Attrition | High risk | Attrition was high (22%) due to missing risk factor data |
| Prognostic Factor Measurement | Low risk | Based on clinical measurements |
| Outcome Measurement | Unclear risk | Half of participants had a negative culture or smear, less than half had a culture-confirmed diagnosis (45%) |
| Study Confounding | Unclear risk | Almost all relevant covariates except for tobacco consumption |
| Statistical Analysis and Reporting | Low risk | Adequately presented Cox regression analysis for the defined model. |

Nicholas 2011

| ***Study characteristics*** | | |
| Study details and sources | **Study design:** Prospective cohort study  **Source of data:** Clinical health records  **Study dates:** 2006 - 2008  **Setting**: Outpatient  **Countries:** Sub-Saharan Africa (Guinea, Kenya, Malawi, Mozambique, Nigeria, Uganda) | |
| Participants | **Participant eligibility**  All HIV-infected adults who entered the programs from January 1, 2006, to September 30, 2008. Those with prevalent tuberculosis at program entry or with less than 15 days of follow-up were excluded.  **Recruitment method**  Consecutive participants from the program  **Participant Description**   - Age: Median 34 years - Sex: 65.9% women   **Risk factors**   - diabetes: Not available - undernutrition: BMI <18.5 24.7% (preART) 34% (ART) - HIV infection: All participants - recent TB infection: Prior TB infection 2.5% (preART) 8.8% (ART) - history of untreated or inadequately treated TB disease: Not available - immunosuppressive therapy: None - cigarette smokers: Not available - drug or alcohol use disorders: Not available - socioeconomic status: Not available - other: 14% WHO STAGE 4   Details of treatments received (treatment for diabetes, undernutrition and other concomitant conditions): Prior history of antirretroviral therapy use 3% | |
| Outcomes | **Tuberculosis (active tuberculosis or tuberculosis disease)**  Definition and method for measurement of outcome:  Pulmonary tuberculosis was diagnosed in patients with symptoms or signs suggestive of tuberculosis, such as fever, night sweats, weight loss, chest radiographic findings (where available), and/or lack of response to at least one course of antibiotics. The results of sputum microscopy for acid-fast bacilli determined whether the diagnosis was a positive or negative smear. Extrapulmonary tuberculosis diagnosis was based on clinical signs or symptoms suggestive of systemic and site-specific tuberculosis (e.g., lymphadenopathy, meningism) and radiological, biochemistry, or microscopic findings. The culture of *Mycobacterium tuberculosis* was not routinely available in the programs.  Was the same outcome definition (and method for measurement) used in all patients? Yes  Type of outcome: combined (all types of diagnosis)  **TB recurrence (relapse or reinfection)**  Measured but not analysed for diabetes or BMI as a risk factor. | |
| Risk factor: undernutrition | Definition and method for measurement: BMI <18.5  Timing of measurement: at patient presentation  Handling of predictors in the modelling: categories | |
| Sample size, missing data and analysis | Number of participants, number of outcomes/events and amount of accumulated person-years (PY): 30134 eligible for inclusion, 8,998 patients with ≥3 months of pre-ART follow-up and 19,325 patients with >15 days of follow-up on ART, adding up to 7754 person-years and 18,162 person-years respectively.  Number of participants, person-years and outcomes/events in relation to the number of candidate predictors (Events Per Variable): 1,536 person-year pre-ART, 362 cases (low BMI), 5,260 person-year ART, 436 cases (low BMI)  Number of participants with any missing value (include predictors and outcomes): Not available  Number of participants with missing data for each predictor: 1% in pre-ART period and 5% in the ART period for BMI  Handling of missing data: sensitivity analysis with complete case for CD4 and BMI.  Modelling method: A random effects Poisson models, assuming a gamma distribution for the random parameter, which accounted for potential intraprogram variation.  Assumptions: Backward stepwise procedure using P > 0.10 for variable exclusion and log-likelihood ratio tests for association (P < 0.05) were used.  Adjustment factors used: treatment site (urban or rural), sex, calendar year at ART initiation or program enrollment (2006 and 2007-2008), age (as a continuous variable), prior history of ART use (naive or experienced), body mass index (BMI: <18.5, ≥18.5 kg/m2, and missing), nadir CD4 cell count (<50, 50-99, 100-199, ≥200 cells/μL, and missing), time since ART start, and prior tuberculosis history (recorded history of tuberculosis at program entry, patients with tuberculosis relapse, failure or treated after defaulting) | |
| Results | **Interpretation:**  “We observed that tuberculosis incidence was 49% lower in patients with normal BMI than in malnourished adults.”  **Comparison with other studies:**  “Poor nutritional status is known to be a risk factor for tuberculosis development (refs 10,13,29,30) and weight loss is a common consequence of tuberculosis.”  **Discussion of generalizability:**  None (besides mentioning the special Doctors Without Borders Program)  **Strengths and limitations:**   - Large sample size - Homogeneity in care provision and data collection - Quality of management heterogeneous - Short follow-up - Low rates of missing data | |
| Funding | Not available | |
| Conflict of interests | Not available | |
| Notes | No email to authors. | |
| ***Risk of bias*** | | |
| **Bias** | **Authors' judgement** | **Support for judgement** |
| Study participation | Unclear risk | Focused on AIDS adults; important baseline variables were not available |
| Study Attrition | Low risk | Minimal attrition (5%) |
| Prognostic Factor Measurement | Low risk | Based on clinical records. |
| Outcome Measurement | Unclear risk | TB diagnosis was based on smear results. Negative smear results were high(67%). Culture-confirmed diagnoses not available |
| Study Confounding | Unclear risk | No information available about DM, history of TB, immunosuppression, alcohol use, smoking, socioeconomic status |
| Statistical Analysis and Reporting | Low risk | Adequately presented Poisson regression analysis for the defined model. |

Okwara 2017

| ***Study characteristics*** | | |
| Study details and sources | **Study design:** prospective cohort  **Source of data:** Close Contacts Program  **Study dates:** December 2011 - July 2013  **Setting:** Outpatient  **Countries:** Kenya | |
| Participants | **Participant eligibility:**  Children < 5 years, living and sleeping under the same roof and sharing facilities with an index case for at least two weeks before the index case’s TB diagnosis (smear-positive TB in the preceding month) was made. Excluded: those with pre-existing co-morbid chronic illnesses like cerebral palsy, congenital cardiac disease and diabetes mellitus.  **Recruitment method:**  Consecutive from TB clinic registers.  **Participant Description:**   - Age: *<24 months 56%* - Sex: *male 51%*   **Risk factors:**   - diabetes: not described - undernutrition: 11.7% - HIV infection: 7% - recent TB infection: not described - history of untreated or inadequately treated TB disease: not described - immunosuppressive therapy: excluded at baseline - cigarette smokers: not described - drug or alcohol use disorders: not described - socioeconomic status: not described - other:   - Birth weight <2500 g 2,7%   - >1 patient visit in 1 year 14%   - BCG vaccination: 92%   - TB suggestive symptoms: 34%   - Visiting social places: 79%   - Baseline TST reaction: 21% positive   - HIV positive: 7%   Details of treatments received (treatment for diabetes, undernutrition and other concomitant conditions): Isoniazid prophylaxis. | |
| Outcomes | **Tuberculosis (active tuberculosis or tuberculosis disease)**  Definition and method for measurement of outcome: Clinical score (age, BCG scar, cough, weight loss, malnutrition, unexplained fever, fatigue, lymphadenopathy, respiratory symptoms despite antibiotics, spine deformity, unexplained abdominal swelling, change in temperament, suggestive or non-specific X-ray, TST positive, sputum positive, suggestive histology/cytology) >7. No culture.  Was the same outcome definition (and measurement method) used in all patients? Yes  Type of outcome: dichotomous (IPT failure)  Time of outcome occurrence or summary of duration of follow-up: 1 year  **TB recurrence (relapse or reinfection)**  Not measured. | |
| Risk factor: undernutrition | Definition and measurement method:   - ‘Weight for age’ was used to assess chronic malnutrition - <60% as severe malnutrition - between 60 and 80% as moderate malnutrition - Weight faltering from growth charts.   Measurement by a qualified paediatrician.  Timing of measurement: at patient presentation.  Handling of predictors in the modelling: dichotomous | |
| Sample size, missing data and analysis | Number of participants, number of outcomes/events and amount of accumulated person-years (PY): 428 close contacts, of which 368 completed 1 year follow up.  Number of participants, person-years and outcomes/events in relation to the number of candidate predictors (Events Per Variable): 6 incident TB cases, 3 in normal nutrition, 3 in malnutrition.  Number of participants with any missing value (including predictors and outcomes): Not described  Number of participants with missing data for each predictor: not described.  Handling of missing data: not described.  Modelling method: “For advanced statistics, factors showing significant associations on bivariate analysis were entered into a multiple logistic regression model, to establish those with an independent relationship with IPT failure.” No other specifications were provided.  Assumptions: “Data on children whose guardians declined further study participation, and those that did not continue follow-up to 1 year were censored at the date of the last visit and excluded from the analysis.”  Adjustment factors used: malnutrition, TB suggestive symptoms at enrollment, baseline TST and HIV status. | |
| Results | **Interpretation:**  “However, our findings suggest that nutrition support of contacts may optimize IPT benefits in resource restricted settlements.”  **Comparison with other studies:**  “Malnutrition is one of the stressors and increases the risk of progression to disease by 2–5 times [ref 19].”  **Discussion of generalizability:**  “Fairly good completion rates (88.8%) and compliance rates (89.0%) were observed in this study.” (for IPT therapy) and “The risk of selection bias in this study arises from the exclusion of transfers-out and those lost to follow-up from analysis for technical and logistical reasons.”  **Strengths and limitations:**   - Adequate follow-up of children but transfer out of the cohort and migration. - Lack of microbiological confirmation by culture. | |
| Funding | The study was partly funded by Kenya National Council of Science Technology and Innovation (NACOSTI) and Kenyatta University, Nairobi. | |
| Conflict of interests | The authors declare that they have no competing interests. | |
| Notes | No queries to authors (study at high risk of bias). | |
| ***Risk of bias*** | | |
| **Bias** | **Authors' judgement** | **Support for judgement** |
| Study participation | Unclear risk | Although the population is well described it represents partially the target population (general population) |
| Study Attrition | High risk | 89% of follow-up with little information about the missing data |
| Prognostic Factor Measurement | Low risk | Based on clinical assessment |
| Outcome Measurement | Unclear risk | Diagnosis based on a clinical scale that included smear test, but it was not mandatory; culture-confirmed diagnoses not available. |
| Study Confounding | High risk | Important confounders were not used in the analyses |
| Statistical Analysis and Reporting | Unclear risk | Poor description of the statistical modelling, although final model presented adequately |

Paradkar 2020

| ***Study characteristics*** | | |
| Study details and sources | Study design: prospective cohort study  Source of data: Patient data and medical examinations  Study dates: August 2014 to December 2017  Setting: outpatient  Countries: India | |
| Participants | **Participant eligibility:**  Household contacts\* (adults and children) of newly diagnosed pulmonary tuberculosis (PTB) adult (> 18 years) patients enrolled into C-TRIUMPH. \*A person who lived in the same house during the three months prior to the diagnosis of TB disease in the index patient (PTB patient)  **Recruitment method:**  Index TBD patients were initially diagnosed in local clinics run by the Indian Revised National Tuberculosis Control Program (RNTCP) and then referred to C-TRIUMPH study sites in Chennai and Pune, within one week of index patient’s anti-TB treatment initiation. After obtaining informed consent from index PTB patients, study staff approached HHC and those willing to participate in the study consented and assented as applicable.  **Participant Description:**   - Household contacts of PTB adult without TB disease at baseline and at least one TB infection test available - Age: 8% <6 years, 14% 6-12 yrs, 9% 13-17 yrs, 50% 18-44 yrs, 18% ≥45 yrs - Sex: 56% female   **Risk factors:**   - diabetes: baseline DM 9% (67/997), incident DM 0% (3/997) - undernutrition (BMI <18.5 ≥18 yrs; BMI/age ≤ minus 2SD for HHC 10-18 yrs; weight/age ≤ minus 2SD for HHC ≤ 10 yrs): 19% (187/997) - HIV infection: 2% (15/997) - recent TB infection:   - Past history of TB 4% (36/997)   - Baseline TST and/or IGRA positive: 71% (707/997) - history of untreated or inadequately treated TB disease: not reported - immunosuppressive therapy: not reported - cigarette smokers: current 9% (58/997), former 3% (21/997) - drug or alcohol use disorders: alcohol consumption (history of any consumption of alcoholic drink) 13% (129/997) - socioeconomic status:   - Level of education: ≤ primary 37% (351/997), high school 42% (399/997), > junior college 21% (199/997)   - Family income (rupees): <10.000 43% (412/997), 10.000-30.000 53% (510/997), >30.000 4% (33/997) - other   - Marital status: married 69% (498/997), divorced/widowed 5% (34/997), never married 26% (189/997)   - BCG scar: present 70% (569/997)   - Baseline TST: positive (≥5mm) 56% (524/997)   - Baseline IGRA: positive (≥0.35 IU/ml) 52% (484/997)   - Baseline TST and IGRA: both positive 55% (301/997)   - Family size (median) 5 (4-6)   - Family type: nuclear 65% (647/997), joint 35% (350/997)   - Residing in slum: yes 27% (262/997)   - Residence type: urban 69% (687/997), rural 31% (310/997)   - Windows (median): 2 (1-3)   - Sleep index (sleep location of the PTB index patient): different room 30% (276/997), same room/different bed 29% (276/997), same room/same bed 42% (397/997)   Details of treatments received (treatment for diabetes, undernutrition and other concomitant conditions): not reported | |
| Outcomes | **Tuberculosis (active tuberculosis or tuberculosis disease)**  Tuberculosis disease (TBD):   - Confirmed TBD: if the HCC had a specimen from any source positive by TB culture or GeneXpert/MTB Rif. - Probable TBD: negative culture or GeneXpert but a specimen from any source positive o AFB smear - Possible TBD: all specimens negative (by culture, GeneXpert and AFB smear) but the person was treated empirically for TB based on clinical and/or radiologic findings.   Was the same outcome definition (and method for measurement) used in all patients? Yes  Type of outcome: Incident TB disease  Time of outcome occurrence or summary of duration of follow-up: 24-month follow-up period. The median time from enrollment of the PTB index patient to the development of iTBD in the HHC was 5.6 months (Range: 4.4 to 10.4 months).  **TB recurrence (relapse or reinfection)**  Not measured | |
| Risk factor: undernutrition | Definition and method for measurement: BMI <18.5 kg/m2 ≥18 yrs; BMI/age ≤ minus 2SD for HHC 10-18 yrs; weight/age ≤ minus 2SD for HHC ≤ 10 yrs (clinically assessed)  Timing of measurement: at enrollment  Handling of predictors in the modelling: Undernourished vs no undernourished | |
| Sample size, missing data and analysis | Number of participants, number of outcomes/events and amount of accumulated person-years (PY): 997 participants (after exclusion), 20 cases, overall incident TB disease rate of 12 per 1000 PY (95% CI = 8 to 19/1000 PY).  Number of participants, person-years and outcomes/events in relation to the number of candidate predictors (Events Per Variable)   - Baseline DM: 67 participants, 1 case of incident TBD, 8 (0-47) IR/1000PY (95% CI) - Incident DM: 3 participants, 0 cases - Undernourished: 187 participants, 7 cases of incident TBD, 23 (9-48) IR/1000PY (95% CI)   Number of participants with any missing value: 42 HHC were excluded because of TBD at baseline and 12 because of no baseline TBI testing available  Number of participants with missing data for each predictor: probably none  Handling of missing data: complete-case analysis  Modelling method: univariate, multivariable, and mixed-effect Poisson regression analyses.   - The HHC characteristics found to be associated with incident tuberculosis disease in the univariate analysis were included in the overall model and/or the adult multivariate models as relevant. - Additionally, those HHC characteristics that were not statistically significant in the univariate analysis but known to be the published risk factors for incident tuberculosis disease were included in the multivariate model   Assumptions: None  Adjustment factors used: HHC characteristics (age, gender, HIV infection status, nutritional status, and baseline TBI status), index patient characteristics (age, gender, HIV infection status, sputum smear and culture status), and household characteristics (residence type, family income, and sleep index) | |
| Results | **Interpretation:**  Undernourished HHC patients with PTB are at higher risk of developing TB disease (aIRR = 6.16, 95%CI: 1.89 to 20.03, p = 0.003) than those who are not undernourished.  DM HCC of patients with PTB are not at higher risk of developing TB disease (unadjusted IRR = 0.64, 95% CI: 0.21–1.91, p = 0.42). However, it was not for incident TB disease in either of the analytical models.  **Comparison with other studies:**  None  **Discussion of generalizability:**  Only HCC of PTB patients and studies conducted in India are not representative of the general population and from other regions.  **Strengths and limitations:**  Strengths: none reported.  Limitations: DM was not included in the adjusted model. | |
| Funding | "Data in this manuscript were collected as part of the Regional Prospective Observational Research for Tuberculosis (RePORT) India Consortium. C-TRIUMPH is part of the RePORT consortium funded with Federal funds from the Government of India’s Department of Biotechnology (DBT), the Indian Council of Medical Research (ICMR), the USA National Institutes of Health (NIH), the National Institute of Allergy and Infectious Diseases (NIAID), the Office of AIDS Research (OAR), and distributed in part by CRDF Global. This work was also supported by the National Institutes of Health, the NIH funded Johns Hopkins Baltimore-Washington-India Clinical Trials Unit for NIAID Networks. Aarti Kinikar, Rajesh Kulkarni and Rahul Lokhande are supported by the BJGMC JHU HIV TB Program funded by the Fogarty International Center, NIH (D43TW009574 to RCB). The contents of this publication are solely the responsibility of the authors and do not represent the official views of the DBT, the ICMR, the NIH, or CRDF Global. Any mention of trade names, commercial projects or organisations does not imply endorsement by any of the sponsoring organisations. The funders had no role in study design, data collection and analysis, decision to publish, or preparation of the manuscript." | |
| Conflict of interests | The authors declared no competing interests. | |
| Notes | No queries to the authors. | |
| ***Risk of bias*** | | |
| **Bias** | **Authors' judgement** | **Support for judgement** |
| Study participation | Unclear risk | Participants were selected in one region and only concerned household contacts of index TB cases referred to the study program. |
| Study Attrition | Low risk | Minimal attrition (1%) |
| Prognostic Factor Measurement | Unclear risk | Partly based on self-report, partly on clinical measurements |
| Outcome Measurement | Low risk | TB was confirmed by biological methods including sputum smear, culture, and Xpert MTB/Rif. Negative results plus anti-TB response were categorized as possible TB cases. |
| Study Confounding | Unclear risk | Only 1 important confounder (immunosuppressive status) missing from the analyses |
| Statistical Analysis and Reporting | Low risk | Adequately presented Poisson regression analysis for the defined model. |

Park 2022

| ***Study characteristics*** | | |
| Study details and sources | **Study design:** retrospective cohort study  **Source of data:** Claims data  **Study dates:** 2009 to 2017  **Setting: Outpatient**  **Countries:** Republic of Korea | |
| Participants | **Participant eligibility:**  People who received a health examination through the NHIS between 1 Jan 2009 and 31 December 2010, and who had received health examinations for four consecutive years prior to 2009. Subjects were excluded if they were diagnosed with TB during 2006-2009 (period for which change of body weight was measured), died or were diagnosed with TB within one year after examination were excluded or if they had any missing variable.  **Recruitment method:**  All patients as described above examined by the NHIS between 1 and 2009-2010 and followed (without further measurements until 31 December 2010 in Korea  **Participant Description:**  Characteristics described according to how many times in the 4 years preceding 2009 the participant was categorized as being underweight.   - Age: undernutrition group (1 year): 34 (29-45); not undernourished: 43 (34-5?) - Sex: undernutrition group (1 year): 47% male; not undernourished: 76.61% male   **Risk factors:**   - diabetes: not undernourished 7.33%; undernutrition group (1 year) 3.17% - HIV infection not reported - recent TB infection not reported - history of untreated or inadequately treated TB disease not reported - immunosuppressive therapy not reported - cigarette smokers   - non: not undernourished 43.89%; undernutrition group (1 year) 61.69%   - ex: not undernourished 20.61%; undernutrition group (1 year) 10.25%   - current not undernourished 35.5%; undernutrition group (1 year) 28.06% - alcohol use disorders   - non: not undernourished 37.64%; undernutrition group (1 year) 48.3%   - mild: not undernourished 54.03%; undernutrition group (1 year) 47.05%   - heavy: not undernourished 4.65%; undernutrition group (1 year) 4.65% - socioeconomic status not reported - other (specify)   - Hypertension: not undernourished 19.93%; undernutrition group (1 year) 6.77%   - Dyslipidemia: not undernourished 16.18%; undernutrition group (1 year) 6.3%   - Waist Circumference: not undernourished 81 (76-87); undernutrition group (1 year) 69 (66-74)   - BMI: not undernourished 23.8 (21.94-25.78); undernutrition group (1 year) 19.05 (18.51-19.77)   Details of treatments received (treatment for diabetes, undernutrition and other concomitant conditions): not reported | |
| Outcomes | **Tuberculosis (active tuberculosis or tuberculosis disease)**  Not measured, but taken form medical data from the NHIS  Type of outcome: occurrence of newly diagnosed TB  Time of outcome occurrence or summary of duration of follow-up: The cohort was followed from 1 year after the health examination date to the date of occurrence of TB or until the end of the study period (7.27 years)  **TB recurrence (relapse or reinfection)**  *Not measured* | |
| Risk factor: undernutrition | **Definition and method for measurement:**  BMI<18.5 kg/m2, clinically assessed; people were stratified according to how often they were categorized as being underweight from 2006-2009 or 2007-2010.  **Timing of measurement:**  At patient presentation  **Handling of predictors in the modelling:**  The cumulative number of underweights was defined as the number of times recorded as underweight at the national health examination during the four consecutive years before the health examination in 2009–2010. | |
| Sample size, missing data and analysis | Number of participants, number of outcomes/events and amount of accumulated person-years (PY): 2,396,434 patients included in analyses; 9,322 cases  Number of participants, person-years and outcomes/events in relation to the number of candidate predictors (Events Per Variable)   - BMI <18.5 kg/m²: (only group 1): 50,077 participants; 335 cases of newly diagnosed TB; 0.9461 IR per 1000 - BMI <18.5 kg/m²: (only group 4): 43,744 participants; 403 cases of newly diagnosed TB; 1.30373 IR per 1000 - BMI ≥18.5 kg/m²: 2,248,457 participants; 8,154 cases of newly diagnosed TB; 0.50811 IR per 1000   Number of participants with any missing value: 112,810 (4.5%)  Number of participants with missing data for each predictor: not specified  Handling of missing data: participants with any missing value were excluded from the analysis  Modelling method:  -multivariable-adjusted proportional hazards model was applied  - P-value < 0.05 was considered statistically significant.  Assumptions  Adjustment factors used: Model 2 was adjusted for age, sex, DM, HTN, and dyslipidemia | |
| Results | **Interpretation:**  "The highest accumulated number of underweight was significantly associated with occurrence of TB. In patient with the highest accumulated number of underweight demonstrated the highest risk of occurrence of TB, event after adjusting for demographic factors and underlying diseases (aHR 3.326, 95% CI 3.004– 3.84)"  **Comparison with other studies:**  "Underweight has been shown to be associated with host susceptibility to TB in several studies. In a similar context, several studies have shown overweight people to have a lower incidence of TB. Lee et al. explored the prevalence rate of active TB among homeless people who had poor nutrition and unsafe housing conditions in Seoul, Korea. In that study, underweight, defined as BMI < 18.5, was an independent risk factor for active pulmonary TB. Kim et al. evaluated the association between BMI and incidence of TB and showed that incident TB decreased as BMI increased after adjusting for age, sex, income, smoking, alcohol, and diabetes. Badawi et al. performed systematic analysis of the relationship between obesity and TB and reported that the adjusted odds ratio of TB was 4.96 in underweight people and 0.26 in obese people. Another systematic literature review found a strong and consistent log-linear inverse relationship between BMI and TB incidence in countries with a variety of TB burdens."  **Discussion of generalizability:**  All of the study subjects were Korean.  **Strengths and limitations:**  Strengths   - Introduced the definition of the accumulated underweight and investigated whether the occurrence of TB increased as the accumulated underweight increased. - It is a nationwide, population-based study with a large population of more than two million. - The detailed personal data collected at baseline enabled the investigators to stratify and adjust for relevant risk factors for TB. Furthermore, there was a one-year time lag between the latest record of weight calculation and TB diagnosis based on ICD codes. In general, retrospective cohort studies may not be able to determine strong cause-effect relationships. Still, setting a time lag could explain the cause-effect relationship even in a retrospective study design.   Limitations   - Being underweight is a heterogeneous and complex condition found in otherwise healthy subjects and patients with underlying diseases, and there is no information about the effect of body composition. Lean mass and fat mass would have different effects on the development of TB. - They could not elucidate the exact pathophysiology of the effect of duration of underweight on the occurrence of TB. More research is needed to clarify the pathophysiology, focusing on TB and the impairment of immune function according to accumulated weight. - The accumulated underweight may not be relevant to the duration of malnutrition. Furthermore, the accumulated number of underweight is not the number of consecutively confirmed underweight. - They did not analyze severe underweight with a BMI of less than 16.5 kg/m2. | |
| Funding | This work was supported as a research program funded by the Konkuk University Medical Center | |
| Conflict of interests | All authors declare that they have no conflict of interests. | |
| Notes | 15 February 2024 Email hkd917@naver.com about cultures. | |
| ***Risk of bias*** | | |
| **Bias** | **Authors' judgement** | **Support for judgement** |
| Study participation | Low risk | Sample from the general population of the entire country |
| Study Attrition | Low risk | Minimal attrition (4.5%) |
| Prognostic Factor Measurement | Low risk | Based on clinical records. |
| Outcome Measurement | Unclear risk | TB was based on medical records, but details on diagnostic practice lacking. |
| Study Confounding | Unclear risk | No information available about HIV, immunosuppression, socioeconomic status |
| Statistical Analysis and Reporting | Low risk | Adequately presented Cox regression analysis for the defined model. |

Park 2023

| ***Study characteristics*** | | |
| Study details and sources | **Study design:** retrospective cohort study  **Source of data:** claims data  **Study dates:** 2002-2017  **Setting:** Outpatient  **Countries:** Korea | |
| Participants | **Participant eligibility:**  Patients who underwent gastrectomy (total or subtotal) or endoscopic submucosal dissection after being newly diagnosed with gastric cancer between 2004 and 2016 and their 10-fold matched controls, excluding any patient who had cancer or TB at least two years prior to gastric cancer diagnosis and those with covariate missing values.  **Recruitment method:**  As described above, all patients were examined by the National Health Insurance Service in Korea from 2002 to 2017.  **Participant Description:**   - Age: mean age 62.1 years (11.2 SD) in each group - Sex: 26.6% of females in each group   Risk factors:   - diabetes n=5,943 (27.6%) in the study group, n=49,147 (21.3%), in the control group - undernutrition BMI <18.5: in study group n=1726 (3.0%), in control group n=5551 (2.4%) - HIV infection: not reported - recent TB infection: not reported - history of untreated or inadequately treated TB disease: not reported - immunosuppressive therapy: not reported - cigarette smokers:   - Current: 16,409 (28.4%) in study group, 55,798 (24.1%) in the control group   - Former: 11,168 (19.3%) in study group, 42,512 (18.4%) in the control group - drug or alcohol use disorders   - Heavy drinkers: 8030 (13.9%) in study group, 28,020 (12.1%) in control group - socioeconomic status (low income): 8298 (14.4%) study group, 34,282 (14.8%) control group - other (specify)   - Mild liver disease: 20,029 (34.7%) study group, 49,253 (21.3%) control group   - Moderate or severe liver disease 450 (0.8%) study group, 1,037 (0.5%) control group   - Renal failure: 984 (1.7%) study group, 2599 (1.1%) control group   - End stage kidney disease: 201 (0.4%) study group, 391 (0.2%) control group   - Chronic obstructive pulmonary disease: 5,529 (32.1%) study group, 8,308 (3.6%) control group   - Interstitial pulmonary diseases: 206 (0.4%) study group, 405 (0.2%) control group   - Other respiratory disease: 18,539 (32.1%) study group, 64,105 (27.7%) control group   - Hypertension: 23,724 (41.1%) study group, 90,005 (38.9%) control group   - None regular exercise: 22,668 (39.2%) study group, 91,683 (39.7%) control group   - Prior chemotherapy: 595 (1.0%) study group, 452 (0.2%) control group   Details of treatments received (treatment for diabetes, undernutrition and other concomitant conditions): not reported | |
| Outcomes | **Tuberculosis (active tuberculosis or tuberculosis disease)**  Definition: incident TB  Method for outcome measurement: extracted from the insurance claims database defined as follows: 1) having a TB dianostic code (A15-A19 or U843) after the dat of gastrectomy or ESD; and 2) having a prescription of 3 or more TB treatment drugs (isoniazid, rifampin, ethambutol, pyrazinamide, and rifabutin) within one year of the TB diagnosis. No details on diagnostic procedure provided.  Was the same outcome definition (and method for measurement) used in all patients? NR  Type of outcome: single endpoint  Time of outcome occurrence or summary of duration of follow-up: The median follow-up duration was 7.45 years for all patients. Total person-years amounted to 179713.  **TB recurrence (relapse or reinfection)**  *Not measured* | |
| Risk factor: undernutrition | Definition and method for measurement: BMI <18.5 clinically assessed.  Timing of measurement: at patient presentation.  Handling of predictors in the modelling: categorical; BMI <18.5, ≥18.5-<23, ≥23-<25, ≥25-<27.5, ≥27.5 kg/m² | |
| Sample size, missing data and analysis | Number of participants, number of outcomes/events and amount of accumulated person-years (PY):  Total number: 288,955 (57,791 for the gastrectomy group, 231,164 for the control group); 1,997 cases in the control cohort; 179,713 PY in the control cohort  Number of participants, person-years and outcomes/events in relation to the number of candidate predictors (Events Per Variable): 1,997 cases for 16 confounders (124 cases per confounder)  Number of participants with any missing value: 1,903 in the study group, 26,057 in the control group  Number of participants with missing data for each predictor: not specified  Handling of missing data (e.g., complete-case analysis, imputation, or other methods): complete case analysis  Modelling method:  - Cox proportional hazard model  - Results were summarized with an adjusted HR and the corresponding 95% CI.  Assumptions: NR  Adjustment factors used: age, sex, liver disease, renal failure, diabetes, ESRD, COPD, interstitial pneumonial disease, other respiratory disease,hypertension, smoking status, alcohol consumption, exercise status, income level, adjuvant chemotherapy status, and fasting plasma glucose level. | |
| Results | **Interpretation:**  A lower BMI and diabetes increased the risk of TB incidence.  **Comparison with other studies:**  None  **Discussion of generalizability:**  "To generalize the results of this study, caution is needed because the incidence and risk factors of TB are closely related to country-specific confounders, such as health expenditure per capita and prevalence rate of HIV, diabetes, undernourishment, smoking, and alcohol use disorders. Especially, the epidemic situation that Korea is currently classified as a middle-burden country of TB, and the age-standardized incidence rate of gastric cancer in Korea (29 per 100,0000 person-years) is 1.8 times higher than that of the global average (16 per 100,000 person-years) should be considered."  **Strengths and limitations**  Limitations:   - since the analysis target disease was defined based on ICD-10 codes of gastric cancer and treatment code of the claims database, the study cohort may not be perfectly consistent with the target patients diagnosed in the clinical field - the history of TB, TB lesions, or contact with tuberculosis patients was not included in the adjustment variables in this analysis | |
| Funding | "This work was supported by a grant from the National Research Foundation of Korea (NRF) funded by the Ministry of Science and ICT of the Korean government (Grant number: NRF-2022R1A2C1004822). The funders played no role in the study design, collection, analysis, and interpretation of data; in the writing of the manuscript; or in the decision to submit the manuscript for publication." | |
| Conflict of interests | The authors declare no conflicts of interest. | |
| Notes | 15 February 2024 Email jwkwon@knu.ac.kr about culture. | |
| ***Risk of bias*** | | |
| **Bias** | **Authors' judgement** | **Support for judgement** |
| Study participation | Unclear risk | Large national database, but focused on people with gastrectomy. |
| Study Attrition | Low risk | Minimal attrition (2.4%) |
| Prognostic Factor Measurement | Low risk | Based on clinical measurements |
| Outcome Measurement | Unclear risk | Based on ICD coding , yet unclear what proportion had culture-confirmed diagnoses |
| Study Confounding | Unclear risk | Information on immunosuppresive status missing |
| Statistical Analysis and Reporting | Low risk | Adequately presented Cox regression analysis for the defined model. |

Pealing 2015

| ***Study characteristics*** | | |
| Study details and sources | **Study design**: Retrospective cohort study  **Source of data**: Electronic health records and claims data  **Study dates**: January 1990 -December 2012  **Setting**: inpatient and outpatient  **Country**: United Kingdom | |
| Participants | **Participant eligibility:**  Inclusion criteria: An exposed cohort was created, composed of patients within the UK Clinical Practice Research Datalink with incident diabetes (types 1 and 2 included), ≥5-years old, who had their first recorded diagnosis for diabetes in the study period, from 1st of January 1990 to 31st of December 2012 (A diagnosis of diabetes was considered incident if the Read code was first recorded on a date at least 12 months after the patient’s current practice registration date).  An unexposed matched cohort was created, composed of those who did not have a prevalent diagnosis of diabetes on the matched index date, which also had to fall 12 months on or after their current registration date. Individual in the unexposed cohort, who developed diabetes would join the exposed cohort for the remainder of their follow-up. Up to six unexposed patients were randomly selected and matched for age +/− 5 years, gender and General Practice with every exposed patient.  Exclusion criteria: Patients were excluded if they had a secondary, gestational or genetic cause of diabetes recorded or had codes indicating the earlier diabetes Read code was incorrect. Those who did not have a feasible temporal sequence for dates of birth, registration and death were excluded. People without a matched control were excluded.  Recruitment method: Consecutive  Participant description:   - Age: Median age of patients with diabetes 62.9 (IQR 52.5–72.5); patients without diabetes 63.6 (53.3–72.9) - Sex: 55% men in the group of patients with diabetes, 53.1% men in the group of patients without diabetes   Risk factors:   - diabetes: 222,731 patients with DM, 1,218,616 without DM - undernutrition: (BMI <20) 4,648 patients (2.1%) in the group of patients with DM, 50,404 (4.1%) in the group without DM - HIV infection: not reported - recent TB infection: not reported - history of untreated or inadequately treated TB disease: 2,376 (1.1%) in the group of patients with DM, 13,867 (1.1%) in the group without DM had previous TB. - immunosuppressive therapy: NR - cigarette smokers:   - current smokers: 42,414 (19.0%) in the DM group, 244,247 (20.0%) in the non DM group   - ex smokers: 93,491 (42.0%) in the DM group, 404,929 (33.2%) in the non DM group   - non smokers: 82,856 (37.2%) in the DM group, 525,881 (43.2%) in the non DM group   - missing 3,970 (1.8%) in the DM group, 43,559 (3.6%) in the non DM group - drug or alcohol use disorders   - non drinker: 31,232 (14.0%) in the DM group, 134,862 (11.1%) in the non DM group   - Ex-drinker 12,151 (5.5%) in the DM group, 44,158 (3.6%) in the non DM group   - Moderate drinker 145,695 (65.4%) in the DM group, 793,877 (65.2%) in the non DM group   - Heavy drinker 18,843 (8.5%) in the DM group, 99,444 (8.2%) in the non DM group   - Missing 14,810 (6.7%) in the DM group, 146,275 (12.0%) in the non DM group - socioeconomic status (Index of Multiple Deprivation)   - 1 – Least deprived: 26,071 (11.7%) DM group, 164,172 (13.5%) non DM group   - 2: 30,191 (13.6%) DM group, 174,611 (14.3%) non DM group   - 3: 27,406 (12.3%) DM group, 149,262 (12.3%) non DM group   - 4: 27,207 (12.2%) DM group, 136,276 (11.2%) non DM group   - 5 – Most deprived: 24,080 (10.8%) DM group, 113,147 (9.3%) non DM group   - Missing: 87,776 (39.4%) DM group, 481,148 (39.5%) non DM group - other (specify) - none   Details of treatments received (treatment for diabetes, undernutrition and other concomitant conditions): not reported | |
| Outcomes | **Tuberculosis (active tuberculosis or tuberculosis disease)**  Definition: Read codes for all forms of tuberculosis (not including presciptions of anti-tuberculosis drugs).  Method for measurement of outcome: NR  Was the same outcome definition (and method for measurement) used in all patients? Likely yes  Type of outcome: TB cases (all types)  Time of outcome occurrence or summary of duration of follow-up: Follow-up ended at the earliest occurrence of first TB diagnosis post-index date, transfer out of practice, last practice data collection, death from any cause or the end of the study, 31 December 2012. The median follow-up time was 4.4 years (IQR 1.9 to 7.8 years) for patients with incident diabetes and 3.8 years (IQR 1.6 to 7.0 years) for matched control patients.  **TB recurrence (relapse or reinfection)**  Not measured | |
| Risk factor: undernutrition | Definition: Not defined as such; BMI < 20 kg/m²  Method for measurement: extracted from electronic health records.  Timing of measurement: at enrollment  Handling of predictors in the modelling: categorical; BMI <20, BMI 20- <25, BMI 25- < 30, BMI ≥30 kg/m² | |
| Sample size, missing data and analysis | Number of participants: 1,441,347 participants.  Number of outcomes/events: 969 cases.  Amount of accumulated person-years (PY): a little less than seven million person-years of follow-up.  Number of participants, person-years and outcomes/events in relation to the number of candidate predictors (Events Per Variable):   - DM: 222,731 participants 97,861 (43.9 %) with complete data included in the analysis, 57.68 PY and 779 cases - non DM: 1,218,616 participants, 461,792 (37.9 %) with complete data included in the analysis, 11.73 PY and 190 cases   Number of participants with any missing value: 881,694 participants with any missing value  Number of participants with missing data for each predictor:   - Smoking: DM 3,970 (1.8%), non-DM 43,559 (3.6%) - Alcohol: DM 14,810 (6.7%), non-DM 146,275 (12.0%) - BMI: DM 11,372 (5.1%), 159,713 (13.1%) - Ethnicity: 90,408 (40.6%), 562,407 (46.2%) - Deprivation: 87,776 (39.4%), 481,148 (39.5%)   Handling of missing data: complete case analysis.  Modelling method:   - Initial univariate analysis of diabetes exposure and the outcome of TB were explored by dividing the exposed cohort into type 1 and type 2 diabetes categories. - A Poisson regression model was used for the adjusted rate ratio of the first diagnosis of TB, comparing patients with diabetes with their matched unexposed cohort, guided by the postulated causal relationships between variables. - Variables were kept in the model if they changed the point estimate for the rate ratio of TB for the exposed cohort. With the addition of each variable, they assessed for possible collinearity by studying changes in standard errors.   Assumptions: it was assumed that UK incidence rates of TB do not vary rapidly over time. An unmatched analysis was used as this does not introduce bias when analysing matched cohort studies.  Adjustment factors used: age, gender, diabetes, alcohol intake, smoking status, Index of multiple deprivation and ethnicity. | |
| Results | **Interpretation**  Results for the association between BMI level and TB were not mentioned in the manuscript text, as the paper aimed to study the association between diabetes and TB incidence. BMI was used as a confounding variable in the analysis.  **Comparison with other studies**  Not reported.  **Discussion of generalizability**  However, the effect size found for diabetes as a risk factor for incident TB is smaller than that found for other studies, probably because the other studies were conducted in countries with high TB incidence, and some included renal transplanted patients. The finding of only a modest increase in the risk of TB with diabetes agrees with more recent studies.  **Strengths and limitations**  *Strengths:*   - One of the largest cohort studies exploring the association between diabetes and TB in a general population adjusted for important individual-level confounding demographic, socioeconomic and lifestyle factors. - The study explores the effects of important patient characteristics, such as age and ethnicity, and aspects of the risk factor of diabetes, such as duration and severity, that have not been previously explored within one cohort. - The study evaluated how TB risk for diabetes patients varies with consultation patterns.   *Limitations:*   - Potential for misclassification of diabetes and TB. - Information on country of origin was not taken into account, nor was HIV status. - Important missing data. | |
| Funding | This article presents independent research supported by a National Institute for Health Research (NIHR) In Practice Fellowship to LP (grant number NIHR/ IPF/11/05). DAJM received Wellcome Trust funding (grant number 092691/Z/ 10/Z). LS is supported by a Wellcome Trust Senior Research Fellowship in Clinical Science. All authors carried out this research independently of the funding bodies. The findings and conclusions in this manuscript reflect those of the authors alone. | |
| Conflict of interests | The authors declare that they have no competing interests. | |
| Notes | No queries to the authors (high risk of bias) | |
| ***Risk of bias*** | | |
| **Bias** | **Authors' judgement** | **Support for judgement** |
| Study participation | Low risk | Participants from a representative sample of general practices in one country |
| Study Attrition | High risk | High rates of missing data (48.8%) |
| Prognostic Factor Measurement | High risk | Based on clinical measurements, yet a large proportion unavailable for analyses |
| Outcome Measurement | Unclear risk | Clinical records for data collection, unclear what proportion had culture-confirmed diagnoses |
| Study Confounding | Unclear risk | No information available on history of TB, immunosuppressive status |
| Statistical Analysis and Reporting | Low risk | Adequately presented regression analysis for the defined model. |

Sabasaba 2019

| ***Study characteristics*** | | |
| Study details and sources | **Study design:** retrospective cohort study  **Source of data**: clinical health records  **Study dates**: January 2011 to December 2014  **Setting**: Outpatient  **Countries**: Tanzania | |
| Participants | **Participant eligibility:**  Inclusion Criteria: HIV-positive adults (≥15 years) enrolled in care and treatment clinics in Dar es Salaam region for the first time between January 2011 and December 2014.  Exclusion Criteria: Patients aged below 15 years, those with missing age information, and patients diagnosed with TB or taking anti-TB medications at enrollment.  **Recruitment method:**  De-identified secondary data of HIV-positive patients enrolled in 50 Care and treatment clinics (CTCs) in Dar es Salaam region between 2011 and 2014. The data was obtained from the electronic database at the National Aids Control Program (NACP), which stores routinely collected HIV information for patient clinical monitoring and reporting to the government. All patients enrolling in HIV care were registered in the database.  **Participant Description:**   - Age: >15 y, median age of 35 y, range: 15-25, 26-35, 36-45, 45+ - Sex: females (75.3%), male (24.7%)   **Risk factors:**   - diabetes: not reported - undernutrition: BMI <18.5 kg/m2 - HIV infection: all patients.   - CD4 count (cells/ul) <200, 200-350, >350   - WHO clinical stage for HIV/AIDS: I, II, III, IV   - ART use: yes, no - recent TB infection: exclusion of those with TB at enrollment excluded, otherwise NR. - history of untreated or inadequately treated TB disease: NR - immunosuppressive therapy: NR - cigarette smokers: NR - drug or alcohol use disorders: NR - socioeconomic status: NR - other (specify)   - marital status: single, married/co-habiting, widowed/divorced   - Cotrimoxazole use: yes, no   - Functional status at enrollment: ambulatory, bedridden, working.   - Health facility type: dispensary, health centre, hospital   - Health facility ownership: government (public), private/FBO   **Details of treatments received (treatment for diabetes, undernutrition and other concomitant conditions):**   - Isoniazid preventive therapy (IPT): HIV-positive adults without signs and symptoms of active TB as well as those treated for TB more than two years earlier. - ART (for some patients) - Cotrimoxazole | |
| Outcomes | **Tuberculosis (active tuberculosis or tuberculosis disease)**  Definition and method for measurement of outcome:  Incident TB was documented in the dataset as a patient newly diagnosed with TB by either an Acid-Fast Bacilli sputum smear or chest radiograph or prescribed anti-TB medications during follow-up. Method of Measurement: Clinical diagnosis and prescribed medications.  Was the same outcome definition (and method for measurement) used in all patients? Yes, incident TB.  Type of outcome: single endpoints  Time of outcome occurrence or summary of duration of follow-up: Patient time at risk for TB was defined as the time from when the client was first enrolled in the CTC to the date of first diagnosis of TB, the last date when the patient visited the clinic or 31st December 2014 (i.e. censoring date) whichever occurred first.  The median follow-up time between patients on IPT was 1.3 years, and for non-ITP, 3.4 years.  **TB recurrence (relapse or reinfection)**  Not measured. | |
| Risk factor: undernutrition | Definition and method for measurement: BMI categories (Underweight <18.5, Normal 18.5-24.5, Overweight 25-29.9, Obese >=30), clinically assessed.  Timing of measurement: at baseline.  Handling of predictors in the modelling: categorized into BMI levels. | |
| Sample size, missing data and analysis | Number of participants, number of outcomes/events and amount of accumulated person-years (PY): 68,378 participants (final eligible sample); 3,124 TB cases; 114,926 Person-Years.  Number of participants, person-years and outcomes/events in relation to the number of candidate predictors (Events Per Variable): 3,124 cases and 8 confounders, 390.5 cases per confounder.  Number of participants with any missing value (include predictors and outcomes): 125 participants had missing age and were excluded.  Number of participants with missing data for each predictor: only those missing age are mentioned.  Handling of missing data (e.g., complete-case analysis, imputation, or other methods): other than exclusion before analysis, not mentioned.  Modelling method:   - Stata version 13.1 (Stata Corp, College Station, TX). - A variable for IPT use was treated as time-updated variable whereas a patient in the IPT group accrued person-time when initiated IPT. - Kruskal-Wallis test was used to compare the medians for continuous variables. - Chi square test was used to determine the association between a set of independent categorical variables and IPT status in bivariate analysis. - Kaplan-Meiyer survival curves were used to estimate cumulative probabilities of being TB free across explanatory variables and then tested using a log-rank test. - To adjust for the clustering effect of the multi-site level correlation of data, each health facility was considered as a primary sampling unit and proper adjustments were considered during data analysis. - Variable was incorporated in multivariate analysis if P-value was 0.05 in the univariate analysis.   Assumptions: not reported.  Adjustment factors used: age, sex, BMI, CD4 count, WHO stage, IPT use, ART status and Functional status at enrollment. | |
| Results | **Interpretation**  "IPT reduces TB incidence rates among HIV-infected adults. Risk factors include being male, underweight, advanced WHO stage, use of cotrimoxazole and ART. The overall TB incidence rate was 2.7/100 person-years (95% CI, 2.6-2.8)."  "Factors associated with significantly increased risk for TB included male patients (aHR = 1.8, 95% CI; 1.6–2.0) compared to females, WHO stage III (aHR = 2.7, 95% CI; 2.3–3.3 and IV (aHR = 2.4, 95% CI; 1.9–3.1) compared to patients with WHO stage I, being underweight (aHR = 1.7, 95% CI; 1.5–1.9) compared to normal weights as well as the history of using ART (aHR = 1.5, 95% CI; 1.0–2.2) and cotrimoxazole (aHR = 2.2, 95% CI; 1.4–3.5) compared to those never used ART or cotrimoxazole respectively."  **Comparison with other studies**   - This finding agrees with that reported in South Africa and Ivory Coast [References 19, 20]. - Risk factors for incident TB in this study are consistent with those reported in previous studies [References 13, 21–24].   **Discussion of generalizability**  Not reported  **Strengths and limitations**  *Strengths*: it involved a large cohort of patients from different facilities (multi-sites).  Limitations:   - Firstly, the eligibility criteria for IPT initiation undertaken in this analysis did not consider past exposure to Isoniazid or repeated course of IPT (i.e. after two years as recommended). - Secondly, the shorter follow-up time for patients on IPT limited the observed TB cases in this group. - Thirdly, they did not also assess the differences in loss to follow-up between those receiving IPT and those not. - Fourthly, they excluded patients with missing age status records, but only a small percentage had missing records. - Fifth, information on routine drug use such as IPT, ART and cotrimoxazole used in this study did not consider details on completion, defaulting or adherence levels during the drug course. | |
| Funding | The authors received no funding for this work. | |
| Conflict of interests | The authors declare that they have no competing interests. | |
| Notes | No queries to authors (high risk of bias). | |
| ***Risk of bias*** | | |
| **Bias** | **Authors' judgement** | **Support for judgement** |
| Study participation | Unclear risk | Focused on AIDS adults in the region |
| Study Attrition | Low risk | 0.2 % Minimal attrition. |
| Prognostic Factor Measurement | Low risk | Based on clinical records. |
| Outcome Measurement | High risk | TB diagnosis was based on smear results, but 74% had negative smear tests. |
| Study Confounding | Unclear risk | No reports about DM, history of TB, immunosuppression, alcohol use, smoking, socioeconomic status. |
| Statistical Analysis and Reporting | Low risk | Adequately presented Cox regression analysis for the defined model. |

Soh 2019

| ***Study characteristics*** | | |
| Study details and sources | **Study design:** prospective cohort study  **Source of data:** surveys and national health TB registry  **Study dates:** 1993 - 31 December, 2014  **Setting**: Outpatient  **Countries**: Singapore | |
| Participants | Participant eligibility: Participants were citizens or permanent residents of Singapore and belonged to major Chinese dialect groups (Hongkoniens and Cantonese). There were 63257 men and women; the final analysis included 50398. Exclusions: participants with a history of active TB before recruitment (n = 3012) and participants with missing data on height and/or weight (n = 9847).  Recruitment method: Recruitment between 1993 and 1998 through in-person interviews to collect information.  Participant Description:   - Age: from 45-74 years, mean 56 years - Sex: male 43.65%, female 56.35%   Risk factors:   - diabetes: history of diabetes (yes %) - undernutrition: <18.5 kg/m2 (underweight), 18.5–<23 kg/m2, 23– <27.5 kg/m2 and >=27.5 kg/m2 (obese) - HIV infection: NR - recent TB infection: excluded - history of untreated or inadequately treated TB disease: excluded - immunosuppressive therapy: NR - cigarette smokers: never, former, current. - drug or alcohol use disorders: alcohol - none, monthly, weekly, daily. - socioeconomic status: based on highest education received. - other (specify)   - dialect group: Cantonese or Hokkien   - level of education: no formal education, primary school, secondary school or above.   - Tea intake: none, monthly, weekly, daily.   - Total energy intake (kcal/day) mean   - Energy-adjusted intake: Protein, cholesterol, marine omega-3 fatty acid, omega-6 fatty acid, Vitamin A, Vitamin C.   Details of treatments received (treatment for diabetes, undernutrition and other concomitant conditions): none reported. | |
| Outcomes | **Tuberculosis (active tuberculosis or tuberculosis disease)**  Definition and method for measurement of outcome:  Active TB cases were identified via linkage with the National TB Registry. The notification of TB cases in Singapore is mandatory by law, and all suspected and confirmed TB cases must be notified to the Ministry of Health within 72 h of the initial treatment for TB and/or the observation of laboratory-confirmed results.  Was the same outcome definition (and method for measurement) used in all patients?: Yes  Type of outcome: single endpoints.  Time of outcome occurrence or summary of duration of follow-up: Person-years of follow-up for each participant were calculated from the date of recruitment to the date of diagnosis of active TB, death, lost to follow-up, or 31 December 2014, whichever occurred earlier.  **TB recurrence (relapse or reinfection)**  Not measured. | |
| Risk factor: undernutrition | Definition and method for measurement: BMI of participants was calculated using weight (kg) divided by height (m) squared (clinically assessed). BMI categories: <18.5 kg/m2 (underweight), 18.5–<23 kg/m2, 23– <27.5 kg/m2 and >=27.5 kg/m2 (obese).  Timing of measurement: (e.g., at patient presentation, at diagnosis, at treatment initiation): at baseline.  Handling of predictors in the modeling:   - Categorized in BMI levels for Asian populations by the WHO to examine the relation between T2D and TB risk, and - Linear trend for the relation between BMI categories and TB risk was tested using median BMI in each category as a continuous variable in the model. | |
| Sample size, missing data and analysis | Number of participants, number of outcomes/events and amount of accumulated person-years (PY): 50,398 participants included; 990 cases of active TB over a mean follow-up period of 16.9 +/- 5.0 years.  Number of participants, person-years and outcomes/events in relation to the number of candidate predictors (Events Per Variable): 144 cases in diabetes, 164 in undernutrition  Number of participants with any missing value (include predictors and outcomes): As of 31 December 2014, only 52 participants were known to be lost to follow-up mainly due to migration out of Singapore.  Number of participants with missing data for each predictor: not reported.  Handling of missing data (e.g., complete-case analysis, imputation, or other methods): excluded in analysis, otherwise not reported.  Modelling method:   - Cox regression models were used to estimate the hazard ratios (HRs) and 95% confidence intervals (CIs) for the relations of T2D and BMI, independently and jointly, with TB risk - Baseline characteristics of participants by their BMI categories and T2D status were compared using the X2 test for categorical variables and analysis of variance or the Student’s t-test for continuous variables. - The Cox models included adjustments for factors that have been shown to affect TB risk in the literature or our cohort. (15–21) - All analyses were conducted using SAS v9.4 (SAS Institute, Cary, NC, USA) statistical software package   Assumptions: none mentioned.  Adjustment factors used (Results in Table 4)   - \* Adjusted for age at recruitment (years), year of baseline interview (1993–1995, 1996–1998), sex, dialect group (Hokkien, Cantonese), level of education (no formal education, primary school, secondary school or higher), smoking status and intensity (never, former, current 1–12 cigarette/day, current 13–22 cigarette/day, current 23 cigarette/day), alcohol intake (none, monthly, weekly, daily), tea intake (none, monthly, weekly, daily), total energy intake (kcal/day) and energy-adjusted intake of protein, cholesterol, marine omega-3 fatty acids, omega-6 fatty acids, vitamin A and vitamin C (quartiles). - † Additionally adjusted for body mass index (<18.5, 18.5 – <23, 23 - <27.5, >=27.5 kg/m2 ) or type 2 diabetes status (no, yes) at baseline. | |
| Results | **Interpretation**:   - "T2D was associated with increased TB risk (HR 2.31, 95% CI 1.93–2.78). Conversely, BMI was inversely associated with TB risk: HR for underweight (BMI < 18.5 kg/m2) was 2.87 (95% CI 2.15 – 3.82) compared to obese (BMI >= 27.5 kg/m2) individuals." - "Compared to obese individuals without T2D, HR for active TB among underweight individuals with T2D was 8.30 (95% CI 4.43–15.54). There was no statistically significant interaction between BMI and T2D on TB risk (P interaction = 0.85)." - "Participants with lower BMI were more likely to be older, current smokers, daily alcohol drinkers, non-drinkers of tea and have lower energy-adjusted daily intake of protein, marine omega-3 and omega-6 fatty acids, vitamin A and vitamin C." - "Every unit increase in BMI was associated with 9% reduction in TB risk (HR 0.91, 95% CI 0.90 – 0.93)."   **Comparison with other studies**   - (Reference 5) A strong inverse relationship exists between BMI and active TB. - (Reference 6) the joint effect of DM and BMI on risk of active TB in two retrospective cohorts in Taiwan. - (Reference 9) The examination of TB risk in lean individuals with T2D carries important implications in Asian populations where T2D develops at lower BMI.   **Discussion of generalizability**  Not reported.  **Strengths and limitations**  *Limitations*:   - The use of self-reported data could be subject to measurement error. - The analysis was performed using baseline BMI, which could change over time. - No data was collected on the use of T2D medication or glycemic control among participants. - Lack of information on other medical conditions associated with increased TB risk and the use of immunosuppressants. - As an observational study, residual confounding could still be present.   *Strengths:* not reported. | |
| Funding | National Institutes of Health, Bethesda, MD, USA (R01 CA144034 and UM1 CA182876). WPK is supported by the National Medical Research Council, Singapore (NMRC/CSA/0055/2013) | |
| Conflict of interests | The authors declare no conflicts of interest. | |
| Notes | No queries to the authors (high risk of bias) | |
| ***Risk of bias*** | | |
| **Bias** | **Authors' judgement** | **Support for judgement** |
| Study participation | Low risk | Large dabatese of Singapore from a population-cohort study |
| Study Attrition | Low risk | Less than 1% |
| Prognostic Factor Measurement | High risk | They excluded 9847 participants with missing data of BMI, no additional information. |
| Outcome Measurement | Unclear risk | Linkage with National Reporting System, unclear how many of these had culture |
| Study Confounding | Unclear risk | Not all important factors, missing definitions (Secondary study from the Cohort) |
| Statistical Analysis and Reporting | Low risk | Statistically with cox regression model. |

Sudfeld 2013

| ***Study characteristics*** | | |
| Study details and sources | **Study design**: prospective cohort study  **Source of data**: medical examinations  **Study dates**: 2006-2009  **Setting**: Outpatient  **Countries**: Tanzania | |
| Participants | **Participant eligibility:**  Tanzanian adults (>=18 y; men and women) initiating ART enrolled in a multivitamin trial (vitamins B complex, C, and E) at high amounts compared with standard amounts of the Recommended Dietary Allowance (RDA), and intended to stay in Dar es Salaam for >=2 y. Women who were pregnant or lactating were excluded.  **Recruitment method:**  Recruited in the context of a trial (NCT00383669).  **Participant Description:**   - Age: (<30 15%, 30–3949%, 40–49 27%, or >=50 y 10%) - Sex: 68% Female   **Risk factors:**   - diabetes: NR - Undernutrition:   - moderate-to-severe malnutrition (<17.0) 13%   - mild malnutrition (>=17.0 and <18.5) 15%   - normal (>=18.5 and <25.0) 58%   - overweight or obese (>=25) 15% - HIV infection: all patients - recent TB infection: baseline diagnosis or receipt of pulmonary tuberculosis treatment 9.4% - history of untreated or inadequately treated TB disease: NR - immunosuppressive therapy: NR - cigarette smokers: NR - drug or alcohol use disorders: NR - socioeconomic status: district (Ilala, Kinondoni, or Temeke) - other (specify)   - highest attained education (none/primary 45% or secondary/advanced 28%)   - number of household assets (0–1 18%, 2–3 30%, or 4–5 27%)   - season of ART initiation [long rain (December to March), harvest (April to May), postharvest (June to August), and short rain (September to November)] - not reported   - randomized multivitamin regimen (single RDA or multiple RDA) - not reported   - baseline CD4 T cell count (<50 23%, 50–99 19%, 100–199 38%, or >=200 cells/mL 21%)   - baseline hemoglobin concentration (<8.5 22%, >=8.5 and <11 43%, or >=11 g/dL 35%)   - baseline WHO HIV stage (I/II 22%, III 64%, or IV 14%)   - baseline oral candidiasis 6%   - Chronic diarrhoea 2%   Details of treatments received (treatment for diabetes, undernutrition and other concomitant conditions): First-line drug combinations for ART included stavudine, lamivudine, nevirapine, zidovudine, and efavirenz.   - Efavirenz was substituted for nevirapine in patients who could not tolerate nevirapine. - Cotrimoxazole prophylaxis was provided when CD4 T cell counts were <200 cells/mL, and treatment of all opportunistic infections was prescribed according to Tanzanian national and WHO guidelines. | |
| Outcomes | **Tuberculosis (active tuberculosis or tuberculosis disease)**  Definition and method for measurement of outcome: Pulmonary tuberculosis was diagnosed according to Tanzanian National Tuberculosis and Leprosy Program guidelines.   - Participants with symptoms of pulmonary tuberculosis were asked to provide the following 3 sputum samples: a spot sputum specimen at the study visit at which symptoms were first reported, an early morning sputum before a second clinic visit the next day, and a third sputum specimen at the second clinic visit. - Participants were diagnosed with pulmonary tuberculosis if at least one of the 3 sputum smears was positive for acid-fast bacilli by using Ziehl-Nielsen staining or when a chest X-ray exhibited radiologic features consistent with tuberculosis when all sputum smears were negative for acid-fast bacilli..   Was the same outcome definition (and method for measurement) used in all patients? Yes  Type of outcome: Single  Time of outcome occurrence or summary of duration of follow-up: monthly clinic visits; median follow-up time was 19.7 months.  **TB recurrence (relapse or reinfection)**  Not measured | |
| Risk factor: undernutrition | Definition and method for measurement: BMI at ART initiation was calculated as the patient’s weight divided by the patient’s height squared. BMI was defined as moderate-to-severe malnutrition (<17.0), mild malnutrition (>=17.0 and <18.5), normal (>=18.5 and <25.0), and overweight or obese (>=25) categories.  Timing of measurement: at baseline and at scheduled monthly clinic visits  Handling of predictors in the modeling: categorized according to BMI level. | |
| Sample size, missing data and analysis | Number of participants, number of outcomes/events and amount of accumulated person-years (PY): 3,389 patients had their BMI measured, 2,255 had their weight measured at 1 month; 147 patients had TB at ART initiation, 83 cases of TB 1 month after ART initiation; PY not mentioned.  Number of participants, person-years and outcomes/events in relation to the number of candidate predictors (Events Per Variable): 83 cases and 12 confounders, 6.92 cases per confounder.  Number of participants with any missing value (include predictors and outcomes): not reported.  Number of participants with missing data for each predictor: Education (n = 905), number of households (n = 857).  Handling of missing data (e.g., complete-case analysis, imputation, or other methods): Missing data for covariates were retained in analyses by using the missing indicator method  Modelling method:   - Proportional hazard models were used to analyze mortality and morbidity associations, whereas generalized estimating equations were used for CD4 T cell counts. - Weight loss at 1 mo was also associated with incident pulmonary tuberculosis (P < 0.001) - P-trend values were calculated by treating the median value of each baseline BMI category as a continuous variable. - Individuals who experienced morbidity events of interest at ART initiation were excluded from baseline BMI analyses. - Individuals without events were censored at the date of the last follow-up visit. - All P-values were 2 sided, and P < 0.05 was considered statistically significant. - Statistical analyses were performed with SAS v 9.2 software (SAS Institute Inc).   Assumptions: proportionality assumptions of Cox models were verified by using time-by-variable interaction terms.  Adjustment factors used: sex, age, district, highest attained education, number of household assets, season of ART initiation randomized multivitamin regimen, baseline CD4 T cell count, baseline hemoglobin concentration, baseline WHO HIV stage, baseline oral candidiasis, baseline diagnosis or receipt of pulmonary tuberculosis treatment, and ART regimen. | |
| Results | **Interpretation**   - "Low BMI at ART initiation and weight loss after one month of treatment were independently associated with subsequent mortality in HIV-infected adults in Tanzania. In addition, the magnitude of the weight-loss association significantly varied across baseline BMI strata." - "Lower BMI at ART initiation was significantly associated with an increased hazard of mortality (P-trend < 0.001)." - "There was also some indication of increased risk of incident pulmonary tuberculosis with lower baseline BMI, but results were not statistically significant (P-trend = 0.063)"   **Comparison with other studies**   - (References 5-11) Multiple studies have shown that low BMI (in kg/m2) at ART initiation is a strong independent predictor of mortality in HIV-infected adults. - (References 9, 10) Previous studies have used 5% weight change categories after three months of ART. - (References 7, 36, 37) Studies conducted in Zambia, Cote d’Ivoire, and Singapore also showed no association of low baseline BMI with the CD4 T cell count change. - (References 38) A study of adults initiating ART in the United States showed significantly greater CD4 T cell increases after 12 mo of ART for individuals with a baseline BMI of 25–30 compared with individuals with a baseline BMI <25.   **Discussion of generalizability**  These findings may be particularly useful in settings where regular laboratory testing of CD4 T cell counts and HIV viral loads are limited or nonexistent.  **Strengths and limitations.**  *Limitations*:   - First, participants did not receive routine HIV viral load monitoring, which may have confounded the results. - Second, only 20% of the sample had baseline CD4 T cell counts >200 cells/mL. As a result, the authors may not have had adequate statistical power to detect the effect modification of any association for individuals with CD4 T cell counts >200 cells/mL at ART initiation. - Third, the authors did not have data on changes in body composition and fat distribution during the study period. - Consequently, the authors could not differentiate individuals who experienced a loss of lean body mass from individuals who experienced lipoatrophy (44).   *Strengths*: not reported. | |
| Funding | Data on funding was not available. | |
| Conflict of interests | None of the authors had a conflict of interest*.* | |
| Notes | No queries to the authors (high risk of bias). | |
| ***Risk of bias*** | | |
| **Bias** | **Authors' judgement** | **Support for judgement** |
| Study participation | Unclear risk | Participants form a clinical trial for people living with HIV. |
| Study Attrition | High risk | 33% of patients possibly lost at follow-up, no detailed information on causes or characteristics. |
| Prognostic Factor Measurement | Low risk | Clinical measure of BMI, 99.2% with BMI data at baseline. |
| Outcome Measurement | Unclear risk | Sputum positive or clinical radiology, not confirmed by culture. |
| Study Confounding | Unclear risk | Some but not all relevant variables (alcohol, diabetes and tobacco missing) |
| Statistical Analysis and Reporting | Unclear risk | Description of statistical model and selection of confounders specified, yet TB was on of the many secondary study outcomes, the analyses were not designed for TB as a secondary outcome; no subgroup or sensititivity analyses undertaken |

Tchakounte Youngui 2020

| ***Study characteristics*** | | |
| Study details and sources | **Study design**: retrospective cohort study  **Source of data**: surveys and medical examination  **Study dates**: 2010-2016  **Setting**: Outpatient  **Countries**: 9 West African countries | |
| Participants | **Participant eligibility:**  HIV-infected patients aged ≥16 years who started ART from 2010 to 2016 and who had ≥1 follow-up visit post–ART initiation who did not receive isoniazid preventive treatment (IPT). *Exclusion*: those with prevalent TB.  **Recruitment method:**  Patients initiated on ART in sites from the IeDEA West Africa Cohort, an international epidemiological research collaboration among 16 adult HIV treatment centres in 9 West African countries.  Four outpatient HIV clinics with recognized quality for TB data collection contributed to this study:   - the daycare center of the Souro Sanou University Teaching Hospital (HDJ/CHUSS) in Bobo Dioulasso, Burkina Faso; - the CRFC, Infectious and Tropical Disease Department, Fann University Teaching Hospital (CRCF/SMITD) in Dakar, Senegal; - the CePReF and CIRBA HIV clinics, both in Abidjan, Côte d’Ivoire.   **Participant Description:**   - Age: 16-30, 30-50, ≥50, median 38.5 years - Sex: female 70.5%, male 29.5%   **Risk factors:**   - diabetes: NR - undernutrition: BMI ≤16, 16-21, ≥21 (see Notes) - HIV infection: all patients   - ART starting year - recent TB infection: TB history (yes, no), defined as TB reported more than 6 months before ART initiation. - history of untreated or inadequately treated TB disease: NR - immunosuppressive therapy: NR - cigarette smokers: NR - drug or alcohol use disorders: NR - socioeconomic status: NR - other (specify)   - Center: CEPREF, CIRBA, HDJ/CHUSS, CRCF/SMITD   - CD4 count: ≥500, 200-500, <200   - Hemoglobin level: ≥11, 9-11, <9   - Serum creatinine   Details of treatments received (treatment for diabetes, undernutrition and other concomitant conditions): ART was initiated in patients with CD4 ≤350 CD4/mm3 in all sites except in CIRBA (Côte d´Ivoire), where it was initiated at CD4 ≤200 before 2012 and ≤350 CD4/mm3 thereafter. | |
| Outcomes | **Tuberculosis (active tuberculosis or tuberculosis disease)**  Definition and method for measurement of outcome:  They defined   1. History of TB as TB reported more than 6 months before ART initiation, 2. Prevalent TB as TB reported between 6 months before and 1 week after ART initiation, and 3. Incident TB as the first TB episode reported after 1 week following ART initiation and during the first year of follow-up.   TB diagnosis was done according to the national TB program (NTP) recommendations of each country. Active screening for symptoms suggestive of tuberculosis (fever, cough, night sweats, weight loss) was conducted, per routine, at ART initiation and at each visit only in the CePReF-CI since 2009.  Was the same outcome definition (and method for measurement) used in all patients? yes after initiation of ART and prevalent TB patients were excluded.  Type of outcome: single endpoints  Time of outcome occurrence or summary of duration of follow-up: Patients were followed up for a median risk period (IQR) of 1.00 (0.73–1.00) years, with 5679.31 person-years of risk period accrued during the study.  **Recurrent TB**  Not measured. | |
| Risk factor: undernutrition | Definition and method for measurement: BMI ≤16, 16-21, ≥21  Timing of measurement: at baseline, otherwise not reported.  Handling of predictors in the modeling: categorized into BMI levels. | |
| Sample size, missing data and analysis | Number of participants with any missing value (including predictors and outcomes): 6,938 total participants in multivariate analysis (n = 4,249, 2,689 observations deleted due to missing data)).  Number of participants with missing data for each predictor: not reported.  Handling of missing data (e.g., complete-case analysis, imputation, or other methods): The missing indicator variable (MIV) method was used for other observations with missing values on BMI, CD4 cell count, TB history, and Hb level.  Modelling method:   - Univariate Poisson models with an offset term to estimate TB incidence rates according to different factors. - Multivariate Poisson model including factors significantly associated with TB incidence at a threshold of .05 in univariate analyses and age that was forced in the model. - Analyses performed using R Studio, version 1.1.456 (R Development Core Team, Vienna, Austria). - All P values were 2-sided and were considered statistically significant if <.05.   Assumptions: none reported  Adjustment factors used: center, gender, age, BMI, CD4 count, Hb level | |
| Results | **Interpretation**  "A total of 189 TB cases were reported from a total of 6,938 patients, for an overall incidence rate of 3.33 cases per 100 person-years (95% CI, 2.85–3.80). The median time from ART initiation to incident TB (IQR) was 2.84 (0.92–5.55) months. In multivariate analysis, TB incidence remained significantly higher in sites in Côte d’Ivoire, in male patients, in patients with low CD4 count, in those with low BMI, in those with a low hemoglobin count, and in the youngest patients."  **Comparison with other studies**   - (References 10) An early observational study conducted in Brazil showed the superiority of combined ART and IPT compared with ART or IPT alone to reduce TB incidence in PWH - (References 11-15) Several observational studies conducted in Africa and a cluster randomized trial conducted in Brazil have shown that using IPT in ART-treated adults in high-incidence settings significantly reduces TB incidence. - (References 16-19) As reported previously, a previous TB history, low CD4 count, young age, and male gender were associated with higher TB incidence, as were anaemia and low BMI.   **Discussion of generalizability**  The study was conducted in 9 countries representing West Africa; otherwise, the authors have not reported anything else.  **Strengths and limitations**  *Limitations*:   - First, the authors may have underestimated TB incidence due to the lack of standardized procedures for TB diagnosis and missed TB in those patients who died or those who were lost to follow-up, further contributing to the underestimation of TB incidence. - Second, the authors chose to define incident TB as occurring after the first week on ART, and this time, the cutoff may not always be appropriate to distinguish between undiagnosed prevalent TB and incident TB, including unmasking IRIS. - Third, the authors could not consider other key co-factors explaining TB incidence, such as tuberculin skin test, BCG immunization, or socio-economic status, because these were not collected.   *Strengths*: None reported. | |
| Funding | "The study was conducted under the IeDEA West Africa collaboration grants funded by the National Cancer Institute (NCI), the National Institute of Mental Health (NIMH), the Eunice Kennedy Shriver National Institute of Child Health & Human Development (NICHD), and the National Institute of Allergy and Infectious Diseases (NIAID); grant number: 5U01AI069919). The funders had no role in the study design and analysis, decision to publish, or preparation of the manuscript." | |
| Conflict of interests | The authors report no conflict of interests. | |
| Notes | We requested information from the authors for the subgroup of BMI <18.5, and they provided the analysis that allowed us to incorporate these findings (18.11.2024). | |
| ***Risk of bias*** | | |
| **Bias** | **Authors' judgement** | **Support for judgement** |
| Study participation | Unclear risk | Cohort of people living with HIV, no data on tobacco and alcohol. |
| Study Attrition | High risk | Identified 8652 patients in database. They have to exclude 1436 patients because of no follow up visit (16%). |
| Prognostic Factor Measurement | Low risk | Unclear how BMI was measure. |
| Outcome Measurement | Unclear risk | They did not clearly explain how they measured TB. They said it was by the national TB program and symptons, but no more. |
| Study Confounding | Unclear risk | Adjusted for some but not all important variables, for instance, not alcohol nor tobacco. |
| Statistical Analysis and Reporting | Unclear risk | Description of statistical model and selection of confounders specified; no subgroup or sensititivity analyses undertaken |

Tiruneh 2019

| ***Study characteristics*** | | |
| Study details and sources | **Study design**: retrospective cohort study  **Source of data**: clinical health records  **Study dates**: September 2009 - 10th September, 2012  **Setting**: Outpatient  **Countries**: Ethiopia | |
| Participants | Participant eligibility:  Inclusion criteria: Adult patients enrolled on ART, aged 18 and older, free of active TB at enrollment, and who completed six months of Isoniazid therapy were included in the exposed group (n = 200). For the unexposed cohort, patients who had never initiated IPT and met all other previously stated criteria were included (n = 400). Exclusion criteria: NR.  Recruitment method: patients receiving ART/HAART (antiretroviral therapy/highly active antiretroviral therapy) at two health facilities in Nekemte town: Nekemte Referral Hospital and Nekemte Health Center.  Participant Description:   - Age: mean 33 years (SD 9); age groups (18-30, 31-40, 41-50, >50) - Sex: 40.7% Male   Risk factors:   - diabetes: NR - undernutrition: 40.7% BMI <18.5 kg/m²   - Weight: 40.7% ≤50 kg - HIV infection: 100%   - WHO Stage: 76.3% stage I/II, 23.7% stage III/IV   - CD4 Count: 4.7% ≤200, 30.5% 201-350, 24.8% 351-500, 30% >500 cells/uL - recent TB infection: NR - history of untreated or inadequately treated TB disease: 20.8% previous TB - immunosuppressive therapy: NR - cigarette smokers: NR - drug or alcohol use disorders: NR - socioeconomic status: Education: 18.4% no education, 22.1% primary education, 26.6% secondary education, 9.3% tertiary education - other (specify)   - Religion: 20.5% Muslim, 40.5% Orthodox, 36.2% protestant, 2.7% Other (Catholic or Adventist)   - Marital status: 51.0% married, 17.3% single, 13.4% divorced/separated, 18.4% Widowed/Widower   - Functional status: 86.2% working, 12.2% ambulatory/Bedridden   - Opportunistic infection: 20.8% yes   - Cotrimoxazole treatment: 71.5% yes   Details of treatments received: both cohorts received ART/HAART (antiretroviral therapy/highly active antiretroviral therapy). Exposed group: ART + Isoniazid from 6 months; Unexposed group: only ART. Cotrimoxazole for some patients. | |
| Outcomes | **Tuberculosis (active tuberculosis or tuberculosis disease)**  Definition: incident tuberculosis  Method for measurement of outcome: NR; extracted from clincal records.  Was the same outcome definition (and method for measurement) used in all patients? Unclear  Type of outcome: single endpoint  Time of outcome occurrence or summary of duration of follow-up: every participant was followed-up for 3 year (median 26 months). Every patient was followed until the occurrence of TB or any of the censorship: lost to follow-up, study cessation year, 10th of September 2012, death, or transferr out of the area, whichever occurred first.  **TB recurrence (relapse or reinfection)**  not measured | |
| Risk factor: undernutrition | Definition: NR; BMI <18.5 kg/m²  Method for measurement: extracted from clinical records.  Timing of measurement: at baseline.  Handling of predictors in the modeling: categorized; BMI <18.5 versus BMI ≥18.5 kg/m². | |
| Sample size, missing data and analysis | Number of participants: 600 adult HIV patients receiving ART.  Number of outcomes/events: 53 cases of incident TB.  Amount of accumulated person-years (PY): 1482.55 person-years.  Number of participants, person-years and outcomes/events in relation to the number of candidate predictors (Events Per Variable): 53 cases and 9 confounders (5 cases per confounder).  Number of participants with any missing value (include predictors and outcomes): NR.  Number of participants with missing data for each predictor: 20 had missing educational status; 25 had missing martital status; Both variables were not accounted for in the analysis.  Handling of missing data (e.g., complete-case analysis, imputation, or other methods): Likely, the variables used in the analysis had no missings.  Modelling method:   - Every variable in the bivariate analysis of a Cox proportional hazard model with a crude hazard ratio of p-value < 0.2 was selected to build the multivariate model. - Each variable was checked for a confounding effect against IPT (main exposure), and the change in the regression coefficient of each variable by less than 20% when compared with a crude model revealed no confounding. - Effect modification was assessed to check whether the effect of IPT (main exposure) was modified when variables were included in the multivariate model. Henceforth, all interaction models were statistically insignificant. - The assumption of a Cox proportional hazard model was not violated.   Assumptions: It was assumed that Isoniazid completers were exposed throughout the follow-up, and untreated groups were not exposed.  The adjustment factors used were age group, CD4 count, weight, WHO stage, functional status, cotrimoxazole treatment, opportunistic infection, and cohort group. | |
| Results | **Interpretation**  Tuberculosis incidence among the Isoniazid-treated group was 1.98 per 100 person-years and 4.52 per 100 person-years in the untreated group. BMI was a significant predictor of tuberculosis risk. At baseline, patients with a BMI below 18.5kg/m² had a 1.85 greater chance of developing TB than those with a BMI above 18.5kg/m2 (AHR= 1.85, CI=1.02-3.55).  **Comparison with other studies**   - "A study conducted in northwest Ethiopia revealed that patients with a BMI of <18.5 at baseline were 1.62 times more likely to get TB as compared to adults with BMI≥18.5 at baseline." - "Another prospective study from Tanzania showed that lower BMI and falling BMI in HIV positive patients was a strong predictor of active TB."   **Discussion of generalizability**  Not reported.  **Strengths and limitations**  *Limitation*: The retrospective nature of the cohort; some important variables like viral load, haemoglobin, adherence status, and ART drug regimen were not included due to problems associated with data inconsistency and incompleteness. | |
| Funding | NR | |
| Conflict of interests | The authors declare that they have no conflicts of interest regarding the publication of this manuscript. | |
| Notes | 15 February 2024 Email gemechu333@gmail.com asking about culture and missing data.  23 February 2024 The author indicated that no culture was available but some had XPert testing, we required additional information to further define the risk of bias judgement. | |
| ***Risk of bias*** | | |
| **Bias** | **Authors' judgement** | **Support for judgement** |
| Study participation | Unclear risk | It was a cohort study from two referal centers for HIV. |
| Study Attrition | Unclear risk | Of those 600 patients, it is not well describe how many were lost to follow up. |
| Prognostic Factor Measurement | Low risk | BMI using clinical health records. |
| Outcome Measurement | Unclear risk | They did not clearly explain how they measured TB, except that it was retrieved from clinical records. |
| Study Confounding | Unclear risk | Not accounted for tobacco, alcohol, previous TB, sex, socioeconomic level. |
| Statistical Analysis and Reporting | Unclear risk | Description of statistical model and selection of confounders specified; no subgroup or sensititivity analyses undertaken |

Van Rie 2011

| ***Study characteristics*** | | |
| Study details and sources | **Study design**: prospective cohort study  **Source of data**: clinical examinations  **Study dates**: April 2004 to March 2008.  **Setting**: Outpatient  **Countries**: South Africa | |
| Participants | Participant eligibility  Inclusion criteria: Adults with HIV infection who initiated ART in the Themba Lethu Clinic between April 2004 and March 2007. Exclusion criteria: NR  Recruitment method: NR.  Participant Description:   - Age: mean age ranged between 36.3 and 36.6 years - Sex: ranged between 37.7% and 37.6% male   Risk factors:   - diabetes: NR - undernutrition: ranged between 74.8% and 84.7% with BMI <18.5 kg/m² - HIV infection: 100%   - CD4 count: median ranged between 56 and 99 cells/mm³ - recent TB infection: NR - history of untreated or inadequately treated TB disease: ranged between 85.2 and 85.8% - immunosuppressive therapy: NR - cigarette smokers: NR - drug or alcohol use disorders: NR - socioeconomic status: NR - other (specify)   - Employed; yes/no   - Low haemoglobin: yes/no   Details of treatments received (for HIV infection): First-line ART regimen of stavudine, lamivudine, and either efavirenz or nevirapine. Second-line ART consisted of azathioprine, didanosine, and ritonavir-boosted lopinavir. | |
| Outcomes | **Tuberculosis (active tuberculosis or tuberculosis disease)**  Definition: Incident TB; a first episode of TB within 6 months after initiation of ART.  Method for measurement of outcome: Pulmonary TB was diagnosed using smear microscopy or algorithms for smear-negative TB that included chest x-ray, culture and the decision of a physician to prescribe a full course of anti-TB treatment. Extrapulmonary TB was defined as a patient in whom a clinician decided to prescribe a full course of anti-TB treatment based on clinical, radiologic, microbiologic, or histopathologic information. Culture-confirmation of a TB diagnosis was not available.  Was the same outcome definition (and method for measurement) used in all patients? No, no further details on diagnostic methods for the study sample available.  Type of outcome: single endpoint.  Time of outcome occurrence or summary of duration of follow-up: Incident TB was diagnosed within 6 months after the initiaton of ART; censoring of patients occurred at death from any cause, loss to follow-up, or administratively on March 31, 2008.  **TB recurrence (relapse or reinfection)**  Definition: Individuals on TB treatment at the time of ART initiation were classified as prevalent TB cases and became at risk of incident TB after completion of treatment for prevalent TB.  Method for measurement of outcome: see above.  This outcome was not considered in the analyses for incident TB. | |
| Risk factor: undernutrition | Definition: BMI <18.5 kg/m²  Method for measurement: NR, likely taken from clinical examinations.  Timing of measurement: at baseline and every 6 months.  Handling of predictors in the modeling: categorized; BMI <18.5, 18.5–24.9, and ≥25 kg/m² | |
| Sample size, missing data and analysis | Number of participants: 7,536 enrolled.  Number of outcomes/events: 284 cases of TB within the first 6 months after starting ART.  Amount of accumulated person-years (PY): 161,000 person-years.  Number of participants, person-years and outcomes/events in relation to the number of candidate predictors (Events Per Variable): 284 cases and 6 confounders (47 cases per confounder).  Number of participants with any missing value (include predictors and outcomes): After enrollment 496 patients (6.6%) died and 1036 patients (13.7%) were lost to follow-up.  Number of participants with missing data for each predictor: 0 participants had missing data for BMI; 8.9% had missing data for CD4 cell count.  Handling of missing data (e.g., complete-case analysis, imputation, or other methods): NR  Modelling method:   - Hazard ratios and 95% confidence intervals (CIs) for risk factors for incident TB (0-6 months) using multivariate Cox proportional hazards regression.   Assumptions: not reported.  Adjustment factors used: History of TB treatment (yes/no), sex, baseline age > median (yes/no), employment (yes/no), low hemoglobin (yes/no), CD4 count (≤ 50, 51-100, 101-200, > 200 cells/mm³). | |
| Results | **Interpretation**  "The cumulative probability of developing TB within 6 months on ART was 5.7% (95% CI, 5.2–6.3) in the first year of ART, 7.6% (95% CI, 6.9–8.3) by the end of the second year, 9% (95% CI, 8.2–9.8) by the end of the third year, and 10.1.% (95% CI, 8.9–11.5) by the end of the fourth year of ART. Poor nutritional status at the time of presentation for ART was also associated with risk of early incident TB with increasing TB rates as BMI decreased: aHR 1.69 (95% CI, 1.18–2.42) and 2.03 (95% CI, 1.34–3.07) for those with BMI 18.5 to 24.9 kg/m2 and less than 18.5 kg/m2, respectively, when compared to BMI greater than 25 kg/m2."  **Comparison with other studies**   - "A study by Lawn et al. in Cape Town, South Africa observed extremely high incidence rates in the first months of ART." - "A log-linear association between risk of TB and BMI in the range 18.5 and 30 kg/m2 was recently demonstrated in HIV uninfected individuals."   **Discussion of generalizability**  Not reported.  **Strengths and limitations**  *Limitations*:   - Patients are mainly diagnosed with TB at primary healthcare clinics, resulting in the lack of bacteriologic confirmation of TB and the possibility of misclassification of TB status. - Causes of death are unknown in most cases, resulting in possible underestimation of TB rates.   *Strengths*: not reported. | |
| Funding | Clinical activities at the Themba Lethu Clinic are supported by the South African National and Gauteng provincial Department of Health with additional funding support from the US President’s Emergency Plan for AIDS Relief (PEPFAR) in a grant by USAID to Right to Care and the Institution (674-A-00-08-00007-00).  A.V.R., D.W., and I.S. received funding from the National Institute for Health National Institute of Allergy and Infectious Diseases (NIAID) DAIDS division (CIPRA IU19 AI53217 and PEPFAR 3 U19 AI 053217-04SI-R2C). A.V.R. also received funding from National Institutes of Health (NIH) Fogarty (IU2R TW 007370). D. W. also received support from an unrestricted educational training grant from the UNC-GSK Center for Excellence in Pharmacoepidemiology and Public Health, UNC School of Public Health, and NIH/NIAID 5 T32 AI 07001-31 Training in STD and AIDS. | |
| Conflict of interests | The authors have no financial, consultant, institutional or other conflict of interest to declare. | |
| Notes | 15 February 2024 Email vanrie@email.unc.edu about culture and missing data. | |
| ***Risk of bias*** | | |
| **Bias** | **Authors' judgement** | **Support for judgement** |
| Study participation | Unclear risk | Single-centre cohort of patients with HIV initiating ART; cohort not extensively described. |
| Study Attrition | High risk | Slighly more than 20% attrition. |
| Prognostic Factor Measurement | Low risk | Based on clinical examinations |
| Outcome Measurement | Unclear risk | Based on clinical symptoms, sputum smears, and chest X-rays- No culture-confirmation of diagnoses available |
| Study Confounding | Unclear risk | No information available for socioeconomic status, alcohol use , smoking and diabetes |
| Statistical Analysis and Reporting | Unclear risk | Description of statistical model given; no specification of the selection of confounders and no subgroup or sensititivity analyses undertaken |

Were 2009

| ***Study characteristics*** | | |
| Study details and sources | **Study design**: prospective cohort study  **Source of data**: clinical assessment and laboratory data  **Study dates**: May 2003 and June 2005  **Setting**: Outpatient  **Countries**: Uganda | |
| Participants | Participant eligibility:  Inclusion criteria: HIV infected adults; aged ⩾18 years; eligible for ART. Exclusion criteria: having active TB at baseline.  Recruitment method: HIV infected adults in the Home-Based AIDS Care (HBAC) project in Uganda enrolled. Clients registered with the AIDS Support Organization (a local HIV/acquired immunodeficiency syndrome care and support organization), were invited to be screened for ART eligibility.  Participant description:   - Age: median 37 years (IQR 52-198) - Sex: 25% males   Risk factors:   - diabetes: NR - undernutrition: 24% BMI ⩽18 kg/m2 - HIV infection: 100%   - CD4 count: no details provided - recent TB infection: NR - history of untreated or inadequately treated TB disease: 0% (exclusion criterion) - immunosuppressive therapy: NR - cigarette smokers: NR - drug or alcohol use disorders: NR - socioeconomic status: NR - other (specify)   - Lymphadenopathy: not further specified; 13%   - Night sweats: 9.3%   - Weight loss: not further specified; .2%   - Cough ⩾3 weeks: 10.1%   - Fever ⩾1 month; 6.0%   Details of treatments received (treatment for diabetes, undernutrition and other concomitant conditions): NR | |
| Outcomes | **Tuberculosis (active tuberculosis or tuberculosis disease)**  Definition: Incident tuberculosis, diagnosed after three months of ART.  Method for measurement of outcome: Patients with symptoms of pulmonary TB were required to have two AFB-positive sputum smears collected on separate occasions to be diagnosed with TB. Patients without two positive smears underwent chest radiography (CXR) and were given a 2-week course of broad-spectrum antibiotics which excluded fluoroquinolones. Patients who were still symptomatic thereafter and had CXRs that were compatible with TB, were then diagnosed with smear-negative pulmonary TB. The diagnosis of extra-pulmonary TB was based on clinical presentation. Microbacterial culture was not available.  Was the same outcome definition (and method for measurement) used in all patients?: No  Type of outcome: single endpoint  Time of outcome occurrence or summary of duration of follow-up: Outcome could only occur after 3 months of ART; median follow-up was 1.4 years.  **TB recurrence (relapse or reinfection)**  Not measured | |
| Risk factor: undernutrition | Definition: BMI ⩽18 kg/m²  Method for measurement: NR.  Timing of measurement: at baseline.  Handling of predictors in the modeling: categorical; BMI ⩽18 kg/m² versus >18 kg/m² | |
| Sample size, missing data and analysis | Number of participants: 1,995 participants enrolled, of whom 1,015 were eligible for ART.  Number of outcomes/events: 28 cases of TB after 3 months of ART.  Amount of accumulated person-years (PY): N/A  Number of participants, person-years and outcomes/events in relation to the number of candidate predictors (Events Per Variable): 28 cases and 6 confounders (4 cases per confounder).  Number of participants with any missing value (include predictors and outcomes): 38 (of whon 32 died, and 6 were discharged from ART before three months on therapy).  Number of participants with missing data for each predictor: NR  Handling of missing data (e.g., complete-case analysis, imputation, or other methods): NR  Modelling method:   - For incident TB, cases were compared to controls (ratio 1:5) matched by age, sex and time on ART for a period that included 3 months before developing TB using conditional logistic regression.   Assumptions: none reported.  Adjustment factors used: for incident TB: cough ⩾3 weeks, weight loss, fever ⩾4 weeks, night sweats lasting ⩾4 weeks, night sweats lymphadenopathy, general weakness ⩾2 weeks. | |
| Results | **Interpretation**  In matched analyses compared to non-TB cases, BMI ≤18 kg/m² had a higher odds of being associated with TB (OR 1.5; 95% CI: 0.4-5.2).  **Comparison with other studies**  No reports of other studies that evaluated the association between undernutrition and incident TB.  **Discussion of generalizability**  Not reported.  **Strengths and limitations**  *Limitations*:   - The diagnosis of active TB was not restricted to cultures of Mycobacterium tuberculosis or biopsy results. Still, it was based on clinical and radiological features, AFB smears and lack of response to non-antimycobacterial antibiotics. - The small number of active TB patients analyzed made it difficult to clearly distinguish between the performance of the different clinical algorithms.   *Strengths*: none reported. | |
| Funding | Funding was provided by the US Centers for Disease Control and Prevention and the US Agency for International Development through the President’s Emergency Plan for AIDS Relief. | |
| Conflict of interests | Potential conflicts of interest were not reported. | |
| Notes | 15 February 2024 Email Dr Were wgw7@ug.cdc.gov about culture and sensitivity analyses. | |
| ***Risk of bias*** | | |
| **Bias** | **Authors' judgement** | **Support for judgement** |
| Study participation | Unclear risk | Population small and not elaborately described. |
| Study Attrition | Low risk | Minimal attrition (3.3%) |
| Prognostic Factor Measurement | Low risk | Likely based on clinical records. |
| Outcome Measurement | Unclear risk | TB diagnosis was based on clinical symptomes, smear results, chest X-rays, or response to a 2-weak sntibiotic treatment. Culture-confirmed diagnoses were not available |
| Study Confounding | Unclear risk | No information on diabetes, immunosuppressive status, alcohol use, smoking, socioeconomic status. |
| Statistical Analysis and Reporting | Unclear risk | Statistical model and selection of confounding variables described; Main purpose was to develop a screening score; no subgroup or sensitivity analyses done. |

Worodria 2011

| ***Study characteristics*** | | |
| Study details and sources | **Study design**: prospective cohort study  **Source of data**: clinical examinations.  **Study dates**: NR.  **Setting**: Outpatient  **Countries**: Uganda | |
| Participants | Participant eligibility:  Inclusion criteria: age >18 years; documented HIV infection; ART eligibility according to Uganda Ministry of Health guidelines (CD4 < 250 cells/μL); ART-naive; no evidence of active TB disease by acid-fast bacilli (AFB) smear microscopy and chest radiograph (CXR); willingness to participate in all followup visits and clinical examinations and to have blood drawn for clinical and immunological studies.  Exclusion criteria: TB at enrollment.  Recruitment method: patients with HIV and eligible for ART at the Infectious Disease Institute in Kampala were enrolled.  Participant Description:   - Age: median 35.7 years (IQR: 30.5-41.3) - Sex: 30% males   Risk factors:   - diabetes: NR - undernutrition: BMI < 18.5 kg/m²; number of participants NR - HIV infection: all patients   - CD4 count: median 130 cells/uL (IQR: 62-170)   - WHO Clinical stage: I, II, III, IV - recent TB infection: 30% Tuberculin Skin Test positive at ART initiation - history of untreated or inadequately treated TB disease: NR - immunosuppressive therapy: NR - cigarette smokers: NR - drug or alcohol use disorders: NR - socioeconomic status: NR - other (specify)   - Hemoglobin (g/dL)   - C-reactive protein (mg/dL)   Details of treatments received (treatment for diabetes, undernutrition and other concomitant conditions): NR. | |
| Outcomes | **Tuberculosis (active tuberculosis or tuberculosis disease)**  Definition: ART-associated TB (defined as all TB diagnosed during ART).  Method for measurement of outcome: following TB screening, participants were judged to have active TB based on clinical assessment, sputum microscopy (incl mycobacterial cultures) or cheast X-ray.  Was the same outcome definition (and method for measurement) used in all patients? No, 50% of the diagnoses were culture-confirmed, the remainder was based on non-culture comfirmed diagnostics.  Type of outcome: single endpoints  Time of outcome occurrence or summary of duration of follow-up: Study participants were followed up and regularly assessed for signs and symptoms suggestive of TB at 2, 4, 8, and 12 weeks after ART initiation, and quarterly thereafter up to one year.  **TB recurrence (relapse or reinfection)**  Not measured | |
| Risk factor: undernutrition | Definition: BMI <18.5 kg/m²; according to WHO recommendations.  Method for measurement:Based on clinical records.  Timing of measurement: at baseline.  Handling of predictors in the modeling: categorized; BMI <18.5 versus BMI ≥18.5. | |
| Sample size, missing data and analysis | Number of participants: 225 participants enrolled.  Number of outcomes/events: 14 cases of TB ( 8 were ART-associated TB).  Amount of accumulated person-years (PY): N/A  Number of participants, person-years and outcomes/events in relation to the number of candidate predictors (Events Per Variable): 14 cases of TB and 7 confounders (2 cases per condounder).  Number of participants with any missing value (include predictors and outcomes): From the 225, 6 participants were excluded before starting ART, and 24 were excluded due to not completing follow up.  Number of participants with missing data for each predictor: NR.  Handling of missing data: excluded from analysis.  Modelling method:   - The predictive value of clinical and laboratory parameters for ART-associated TB was examined by Cox proportional hazards and reported as hazard ratios.   Assumptions: not reported  Adjustment factors used: age, sex, tuberculin skin test (positive/negative), baseline C-reactive protein (mg/L), baseline haemoglobin (g/dL), baseline CD4 cell counts (cells/uL), and WHO clinical stage (1 and 2, 3 and 4). | |
| Results | **Interpretation**  "A body mass index of less than 18.5 kg/m² BMI (HR 5.85 95% CI 1.24–27.46) was a risk factor for ART-associated TB at multivariate analysis."  **Comparison with other studies**  Low BMI is a marker for poor prognosis in patients with HIV and has also been associated with an increased risk of TB and death.  **Discussion of generalizability**  Not reported.  **Strengths and limitations**  *Limitations*:   - The sample size was small. - 24 Participants did not complete the one-year follow-up, and 9 of them died. Postmortem examinations were not performed, and therefore, they possibly underestimated the burden of TB. - Definite diagnosis (sputum smear positivity or culture-positive results) could not be ascertained in 2 patients considered to have ART-associated TB. - Sputum induction, bronchoscopy, liquid culture, and/or molecular techniques may have detected more patients with subclinical TB.   *Strengths*: not mentioned. | |
| Funding | This study received financial support of an EC FP6 Specific Targeted Research Project (STREP) no. LSHP-CT-2007- 037659-TBIRIS. | |
| Conflict of interests | COIs were not declared | |
| Notes | No queries to the authors were necessary. | |
| ***Risk of bias*** | | |
| **Bias** | **Authors' judgement** | **Support for judgement** |
| Study participation | Unclear risk | HIV infected adults from on undefined clinical center; population sparsely described |
| Study Attrition | Unclear risk | 11% attrition due to loss to follow-up |
| Prognostic Factor Measurement | Low risk | Based on clinical records. |
| Outcome Measurement | Unclear risk | Based on a clincial assessment, sputum microscopy or chest X-ray; 50% of the diagnoses was culture-confirmed. |
| Study Confounding | Unclear risk | No information on diabetes, history of TB, immunosuppression, alcohol use, smoking, socioeconomic status. |
| Statistical Analysis and Reporting | Unclear risk | Only the analysis techniques was described. Selection of confounding variables and subgroup- or sensitivity analyses were not described or performed. |

Yen 2017

| ***Study characteristics*** | | |
| Study details and sources | **Study design**: retrospective cohort study  **Source of data**: survey and national insurance claims.  **Study dates**: 2005 to 2013  **Setting**: Outpatient  **Countries**: Taiwan | |
| Participants | Participant eligibility  Inclusion criteria:Adults (aged ⩾18 years); participation in the cross-sectional National Health Survey (NHIS) in 2001, 2005 or 2009. Exclusion criteria: tuberculosis at baseline.  Recruitment method: all participants from three rounds (2001, 2005 and 2009) of the National Health Interview Survey (NHIS).  Participant description:   - Age: mean age 42.5 years (SD 16.4) - Sex: 50.1% Male   Risk factors:   - diabetes: 14.4% - undernutrition (BMI < 18.5 kg/m²): 6.7% - HIV infection: NR - recent TB infection: NR - history of untreated or inadequately treated TB disease: 0% (exclusion criterium) - immunosuppressive therapy: NR - cigarette smokers: 25.4% - drug or alcohol use disorders: 41.8% alcohol consumption - socioeconomic status: 22.6% ≤elementary school, 45.2% high school, 32.1% university or higher - other:   - Marriage status (married/cohabiting, never married, other).   - Comorbidities: hypertension, chronic renal failure, COPD, asthma, cancer   Details of treatments received (treatment for diabetes, undernutrition and other concomitant conditions): NR | |
| Outcomes | **Tuberculosis (active tuberculosis or tuberculosis disease)**  Definition: incident active tuberculosis  Method for measurement of outcome: The presence of an ICD-9-CM code 010–018 and a prescription for at least two anti-TB drugs (e.g., isoniazid, ethambutol, rifampin or pyrazinamide) for 4 weeks.  Was the same outcome definition (and measurement method) used in all patients?: yes.  Type of outcome: single endpoint  Time of outcome occurrence or summary of duration of follow-up: Incident cases of active TB during follow-up were identified by linking the NHIS database to the National Health Insurance Research database.  **TB recurrence (relapse or reinfection)**  NR | |
| Risk factor: undernutrition | Definition: BMI <18.5 kg/m²  Method for measurement: BMI was self reported.  Timing of measurement: at baseline interview.  Handling of predictors in the modeling: categorized: obese (BMI ⩾27), overweight (BMI 24–26.9), normal (BMI 18.5–23.9), and underweight (BMI<18.5). | |
| Sample size, missing data and analysis | Number of participants: 46,028  Number of outcomes/events: 241  Amount of accumulated person-years (PY): N/A  Number of participants, person-years and outcomes/events in relation to the number of candidate predictors (Events Per Variable): 241 cases and 13 confounders (18 cases per confounder).  Number of participants with any missing value (include predictors and outcomes): 2,303 (4.7%).  Number of participants with missing data for each predictor: 1 for sex; 2,303 for BMI.  Handling of missing data: excluded from data analysis.  Modelling method:   - Crude associations between covariates with the outcome (active TB) by computing odds ratios and corresponding 95% confidence intervals (CI). - Multivariable logistic regression was used to estimate the association between BMI and active TB, with adjustment for potential confounders. - Dose–response relations were evaluated between BMI (as a continuous variable) and incident TB. - A sensitivity analysis excluding new TB cases within one year and two years after enrollment. - Adjusted odds ratios (AOR) with 95% CI were reported to show the strength and direction of these associations. - All data management and analyses were performed using SAS software (version 9.4; SAS Institute,Cary, NC, USA).   Assumptions: none reported.  Adjustment factors used: age, sex, smoking, alcohol consumption, socioeconomic status , diabetes, hypertension, chronic renal failure, COPD, asthma, and cancer). | |
| Results | **Interpretation**  "After controlling for subject demographics and comorbidities, underweight was associated with a higher risk of incident active TB (OR, 1.32; 95% CI, 0.79 − 2.20). Factors independently associated with incident TB were older age, male sex, current smoking, diabetes and COPD. This study observed a strong linear dose–response relation of increasing BMI with lower risk of incident TB. The findings indicate that obesity is a protective factor in TB development."  **Comparison with other studies**   - Two cohort studies showed that obesity and overweight were associated with a lower risk of active TB in adults and elderly adults. - Two prospective studies found that obese/overweight HIV-infected individuals had a lower risk of TB development than those of normal weight. - A prior report showed that improved nutrition in a population could reduce TB incidence.   **Discussion of generalizability**  The study studied a general population from a nationally representative sample, thus resulting in greater generalizability. However, the population was only Taiwanese, so generalizability to other Asian ethnic populations requires further verification.  **Strengths and limitations**  *Strengths*:   - Large health surveys were used to assemble a cohort and cross-link with a national health insurance database with more than 99% coverage nationwide. - The prospective direction of follow-up in this study.   *Limitations*:   - Information on deaths among study subjects was not recorded in the national health insurance database. Because obese individuals have a higher hazard rate for competing death than normal-weight persons, it would lead to underestimating the association between obesity and incident TB. - Data on the results of bacteriological studies for TB diagnosis were unavailable. The present definition of TB relied on ICD-9-CM codes and prescription history, and TB outcomes may have been misclassified. - This study only collected data on BMI at the baseline. - The external validity of our findings may be a concern because almost all our enrollees were Taiwanese. Thus, the generalizability of our results to other non-Asian ethnic groups requires further verification. | |
| Funding | Not available | |
| Conflict of interests | The authors declare no conflict of interest. | |
| Notes | 15 February 2024 Email DAD57@tpech.gov.tw about culture confirmation. | |
| ***Risk of bias*** | | |
| **Bias** | **Authors' judgement** | **Support for judgement** |
| Study participation | Low risk | Large sample of the national population. |
| Study Attrition | Low risk | Minimal attrition (4.7%). |
| Prognostic Factor Measurement | Unclear risk | BMI was based on self-report in in-person interviews. |
| Outcome Measurement | Unclear risk | Based on ICD coding, yet unclear what proportion had culture-confirmed diagnoses. |
| Study Confounding | Unclear risk | No information on immunosuppresive status available. |
| Statistical Analysis and Reporting | Low risk | Adequately presented logistic regression analysis for the defined model. |

Yoo 2021a

| ***Study characteristics*** | | |
| Study details and sources | **Study design**: Retrospective cohort study  **Source of data**: Survey and insurance claims data  **Study dates**: January 2009 - December 2018  **Setting**: Outpatient  **Countries**: South Korea | |
| Participants | Participant eligibility  Inclusion criteria: aged 66 years; participation in the National Screening Program for Transitional Ages for Koreans (NSPTA) between 2009 and 2014.  Exclusion criteria: any registered disability, missing data, having a diagnosis of TB, anaemia, cancer or end-stage renal disease before participation in the NSPTA.  Recruitment method: voluntary participation in the free-of-charge NSPTA.  Participant Description:   - Age: 66 years - Sex: 44.8% male   Risk factors:   - diabetes: 19.5% - undernutrition: 1.9% BMI <18.5 kg/m² - HIV infection: NR - recent TB infection: NR - history of untreated or inadequately treated TB disease: 0% (exclusion criterium) - immunosuppressive therapy: NR - cigarette smokers: 12.7% current smokers - drug or alcohol use disorders: alcohol - 23.9%moderate consumers (<30 g/day) and 4.7% heavy consumers (⩾30 g/day) - socioeconomic status: Education: NR - other (specify)   - Income (quartiles)   - Hemoglobin (g/L)   - Sarcopenia (TUG test; sec)   - Physical activity: none, irregular (<30 min moderate activity at least 5 times/week, or <20 min strenuous activity at least 3 times/week), regular (>30 min moderate activity at least 5 times/week, or >20 min strenuous activity at least 3 times/week)   - glomerular filtration rate (ml/min/1.73 m²)   - Ischemic heart disease: 11.3%   - stroke: 3.9%   - pulmonary disease:   Details of treatments received: NR | |
| Outcomes | **Tuberculosis (active tuberculosis or tuberculosis disease)**  Definition: Incident tuberculosis  Method for measurement: Based on insurance codes that are required for patients with active TB after confirmation of their diagnosis to receive free medication (V000, V206, and V246).  Was the same outcome definition (and method for measurement) used in all patients? Yes  Type of outcome: single endpoints  Time of outcome occurrence or summary of duration of follow-up: The cohort was followed from baseline to the date of incident TB or death, or until the end of the study period (31 December 2018), whichever came first.  **TB recurrence (relapse or reinfection)**  NR | |
| Risk factor: undernutrition | Definition: BMI <18.5 kg/m² (according to the AsiaPacific criteria of the World Health Organization) Method for measurement: BMI (kg/m²) was calculated as the subject’s weight in kilograms divided by the square of the subject’s height in meters.  Timing of measurement: at baseline.  Handling of the predictor in the modelling: categorized; underweight (<18.5 kg/m2), normal (18.5–23 kg/m2), overweight (23–25 kg/m2), obese (25–30 kg/m2), and severely obese (⩾30 kg/m2) | |
[truncated: 28,411 more chars]
