# Supplementary material for: Undernutrition as a risk factor for tuberculosis disease
Source: Cochrane Database Syst Rev. 2024 Jun 11;2024(6):CD015890. doi: 10.1002/14651858.CD015890.pub2 (PMC11165671; doi:10.1002/14651858.CD015890.pub2)
Supplement: Supplementary file 3 — Supplementary material 3 Characteristics of excluded studies [file CD015890-SUP-03-characteristicsOfExcludedStudies.html]

Characteristics of excluded studies


# Supplementary material 3 to: Undernutrition as a risk factor for tuberculosis disease

Franco JVA, Bongaerts B, Metzendorf MI, Risso A, Guo Y, Peña Silva L, Boeckmann M, Schlesinger S, Damen JAAG, Richter B, Baddeley A, Bastard M, Carlqvist A, Garcia-Casal MN, Hemmingsen B, Mavhunga F, Manne-Goehler J, Viney K
  
https://doi.org/10.1002/14651858.CD015890.pub2

The material in this section has been supplied by the author(s) for publication under a Licence for Publication and the author(s) are solely responsible for the material. Cochrane has reviewed this material, but Cochrane has not copyedited, formatted or proofread. Cochrane accordingly gives no representations or warranties of any kind in relation to, and accepts no liability for any reliance on or use of, such material.

Back to top

# Characteristics of excluded studies

## Table of contents

- Studies ordered by Study ID
  - Aachari 2022
  - Abedi 2019
  - Adem 2020
  - Afshari 2023
  - Agarwal 2017
  - Aguilera 2016
  - Al Kubaisy 2003
  - Allen 1992
  - Ambrosetti 1999
  - Anaam 2023
  - Antonio-Arques 2022
  - Anyalechi 2022
  - Arriaga 2021
  - Auguste 2021
  - Auld 2014
  - Auld 2021
  - Bai 2016
  - Benoit 2017
  - Bhargava 2014
  - Bridson 2015
  - Byberg 2012
  - Campbell 2022
  - Centis 2000
  - Centis 2002
  - Chao 2016
  - Chen 2006
  - Chen 2013
  - Chen 2014
  - Cheng 2018
  - Cheng 2020a
  - Cherkezov 1973
  - Christopoulos 2009
  - Chu 2019
  - Chung 2014
  - Chung-Delgado 2012
  - Ciang 2020
  - Critchley 2018
  - DeAtley 2021
  - Delgado-Sánchez 2015
  - Demlow 2015
  - Dobler 2012
  - Dyck 2007
  - Eksombatchai 2023
  - Eswarappa 2020
  - Eun-Kyoung 2010
  - Fang 2015
  - Fikrie 2019
  - Franke 2013
  - Frans 2020
  - Golub 2019
  - Hermosilla 2017
  - Herzmann 2017
  - Hoge 1994
  - Hoon 2006
  - Hoshino 2009
  - Hsien 2017
  - Hu 2014
  - Ilievska-Poposka 2018
  - Jayalakshmi 1958
  - Ji 2020
  - John 2001
  - Jurcev-Savicevic 2013
  - Ju-Young 2008
  - Kamper-Jørgensen 2015
  - Kawatsu 2015
  - Khan 2006
  - Khanna 1968
  - Kim 1995
  - Kim 2014
  - Klote 2006
  - Ko 2022
  - Koesoemadinata 2017
  - Ku 2019
  - Kubjane 2020
  - Kufa 2016
  - Kumar 2018
  - Kuo 2013
  - Kuo 2013a
  - Laghari 2018
  - Lee 2008
  - Lee 2013
  - Lee 2015
  - Lee 2016
  - Leow 2014
  - Leung 2007a
  - Leung 2008
  - Li 2020
  - Li 2021
  - Li 2022
  - Liao 2016
  - Lin 1998
  - Lin 2019
  - Liu 2020
  - Lo 2016
  - Lozada Muñoz 2018
  - Lu 2021
  - Ludes 1965
  - Luk 2021
  - Martinez 2017
  - Martinez 2023
  - McAllister 2020
  - McDermid 2013
  - Meinerz 2016
  - Mor 2016
  - Moreno Díaz 2011
  - Moura 2012
  - Mujtaba 2022
  - Mupfumi 2018
  - Naidoo 2022
  - Nakanjako 2010
  - Nanda 1968
  - Nanta 2011
  - Narain 2018
  - Ogbo 2018
  - Olmos 1989
  - Pan 2020
  - Park 2019
  - Park 2022a
  - Patra 2014
  - Pearson 2019
  - Pereira e Silva 1985
  - Pérez-Navarro 2011
  - Picon 2007
  - Picon 2007a
  - Ponce-De-Leon 2004
  - Prakash 2013
  - Prince 2016
  - Qiu 2017
  - Rahim 2012
  - Rajan 2017
  - Ronald 2019
  - Rueda 2013
  - Salvadori 2017
  - Saunders 2017
  - Saunders 2020
  - Scordo 2021
  - Shadrach 2021
  - Shen 2014
  - Sil 2021
  - Singh 2016
  - Sinha 2023
  - Skodrić-Trifunović 2004
  - Stein 2018
  - Tao 2021
  - Tatar 2009
  - Teng 2019
  - Tian 2014
  - Tipayamongkholgul 2016
  - Tverdal 1986
  - Umeta 2022
  - Velen 2021
  - Walker 2010
  - Wang 2019
  - Watch 2017
  - Wu 2015
  - Yang 2018
  - Yoo 2021b
  - Young 2012
  - Zahr 2016
  - Zakopaĭlo 1991
- Footnotes
- References to studies

## Studies ordered by Study ID

| Study | Reason for exclusion |
| --- | --- |
| Aachari 2022 | Wrong study design: cross-sectional study. |
| Abedi 2019 | Wrong patient population: All participants had TB at baseline. |
| Adem 2020 | Wrong study design: cross sectional study |
| Afshari 2023 | Wrong study design: cross-sectional study. |
| Agarwal 2017 | Wrong exposure: No assessment of undernutrition as a risk factor. |
| Aguilera 2016 | Wrong outcomes: no risks estimates (only incidence). |
| Al Kubaisy 2003 | Wrong outcomes: latent TB infection |
| Allen 1992 | Wrong exposure: BMI in quartiles, no cut-off point for undernutrition |
| Ambrosetti 1999 | Wrong patient population: All participants had TB at baseline. |
| Anaam 2023 | Wrong outcomes: no risks estimates (only incidence) |
| Antonio-Arques 2022 | Wrong exposure: Undernutrition is not assessed as a risk factor |
| Anyalechi 2022 | Wrong outcome: Only univariate (crude) estimate reported for incidence TB. |
| Arriaga 2021 | Wrong exposure: Undernutrition is not assessed as a risk factor |
| Auguste 2021 | Wrong patient population: All participants had TB at baseline. |
| Auld 2014 | Wrong outcome: prevalent TB rather than incident TB |
| Auld 2021 | Wrong exposure: weight for age modelled as a continuous exposure |
| Bai 2016 | Wrong patient population: All participants had TB at baseline. |
| Benoit 2017 | Wrong study design: cross-sectional study. |
| Bhargava 2014 | Wrong study design: calculation of population attributable fraction from estimates from the literature |
| Bridson 2015 | Wrong study design: cross-sectional study. |
| Byberg 2012 | Wrong exposure: Undernutrition is not assessed as a risk factor (BMI as a continous variable). |
| Campbell 2022 | Wrong outcomes: recurence is a composite of treatment failure and true recurrence. |
| Centis 2000 | Wrong patient population: All participants had TB at baseline. |
| Centis 2002 | Wrong patient population: All participants had TB at baseline. |
| Chao 2016 | Wrong exposure: low weight as BMI <21. |
| Chen 2006 | Wrong exposure: Undernutrition is not assessed as a risk factor |
| Chen 2013 | Wrong exposure: Undernutrition is not assessed as a risk factor |
| Chen 2014 | Wrong study design: cross-sectional study. |
| Cheng 2018 | Wrong exposure: Undernutrition is not assessed as a risk factor |
| Cheng 2020a | Wrong exposure: Famine. |
| Cherkezov 1973 | Wrong study design: descriptive study of the epidemiology of TB. |
| Christopoulos 2009 | Wrong exposure: Undernutrition is not assessed as a risk factor (relative risk of TB according to BMI compared to the general population). |
| Chu 2019 | Wrong exposure: Undernutrition is not assessed as a risk factor |
| Chung 2014 | Wrong study design: cross-sectional study. |
| Chung-Delgado 2012 | Wrong exposure: BMI in relation to obesity/overweight. |
| Ciang 2020 | Wrong exposure: Undernutrition is not assessed as a risk factor |
| Critchley 2018 | Wrong outcomes: no risks estimates (only incidence). |
| DeAtley 2021 | Wrong study design: cross-sectional study. |
| Delgado-Sánchez 2015 | Wrong patient population: All participants had TB at baseline. |
| Demlow 2015 | Wrong study design: cross-sectional study. |
| Dobler 2012 | Wrong exposure: Undernutrition is not assessed as a risk factor |
| Dyck 2007 | Wrong exposure: Undernutrition is not assessed as a risk factor |
| Eksombatchai 2023 | Wrong exposure: Undernutrition is not assessed as a risk factor |
| Eswarappa 2020 | Wrong outcomes: no risks estimates (only incidence). |
| Eun-Kyoung 2010 | Wrong exposure: people with diabetes vs people with hypertension. |
| Fang 2015 | Wrong exposure: Undernutrition is not assessed as a risk factor |
| Fikrie 2019 | Wrong exposure: TB as a risk factor for recovery of malnutrition. |
| Franke 2013 | Wrong outcome: Only univariate (crude) estimate reported |
| Frans 2020 | Wrong study design: cross-sectional study. |
| Golub 2019 | Wrong exposure: Undernutrition is not assessed as a risk factor (BMI as a continous variable). |
| Hermosilla 2017 | Wrong study design: cross-sectional study. |
| Herzmann 2017 | Wrong outcome: Only univariate (crude) estimate reported (P value). |
| Hoge 1994 | Wrong outcome: unadjusted estimate. |
| Hoon 2006 | Wrong outcome: no risk estimates (only p values from univariate analysis). |
| Hoshino 2009 | Wrong study design: cross-sectional study. |
| Hsien 2017 | Wrong exposure: Undernutrition is not assessed as a risk factor |
| Hu 2014 | Wrong exposure: Undernutrition is not assessed as a risk factor |
| Ilievska-Poposka 2018 | Wrong exposures: no assessment of undernutrition or diabetes as risk factors. |
| Jayalakshmi 1958 | Wrong outcome: vitamin deficit as risk factor. |
| Ji 2020 | Wrong study design: case-control study |
| John 2001 | Wrong exposure: Undernutrition is not assessed as a risk factor |
| Jurcev-Savicevic 2013 | Wrong study design: case-control study. |
| Ju-Young 2008 | Wrong exposure: Undernutrition is not assessed as a risk factor. |
| Kamper-Jørgensen 2015 | Wrong patient population: All participants had TB at baseline. |
| Kawatsu 2015 | Wrong study design: calculation of population attributable fraction from estimates from the literature. |
| Khan 2006 | Wrong outcomes: no risks estimates (only incidence), wrong definition of relapse/recurrence (persistent culture). |
| Khanna 1968 | Wrong patient population: All participants had TB at baseline. |
| Kim 1995 | Wrong exposure: Undernutrition is not assessed as a risk factor |
| Kim 2014 | Wrong exposures: BMI <22 and no assesment of diabetes. |
| Klote 2006 | Wrong exposure: BMI defined in quartiles. |
| Ko 2022 | Wrong exposure: Undernutrition is not assessed as a risk factor (only as a covariate). |
| Koesoemadinata 2017 | Wrong study design: case-control study. |
| Ku 2019 | Wrong exposure: BMI <25 vs >25. |
| Kubjane 2020 | Wrong study design: two assessments of prevalence (not incidence) in cross-sectional measurements. |
| Kufa 2016 | Wrong outcome: unadjusted estimate and adjusted estimate by antiretroviral status only. |
| Kumar 2018 | Wrong study design: cross-sectional study. |
| Kuo 2013 | Wrong exposure: Undernutrition is not assessed as a risk factor |
| Kuo 2013a | Wrong exposure: Undernutrition is not assessed as a risk factor |
| Laghari 2018 | Wrong study design: cross-sectional study. |
| Lee 2008 | Wrong exposure: Undernutrition is not assessed as a risk factor |
| Lee 2013 | Wrong exposure: Undernutrition is not assessed as a risk factor |
| Lee 2015 | Wrong exposures: risk of TB related to co-interventions (drugs). |
| Lee 2016 | Wrong exposure: Undernutrition is not assessed as a risk factor (BMI of 25 as a risk factor). |
| Leow 2014 | Wrong exposures: all participants with diabetes, low BMI <23. |
| Leung 2007a | Wrong exposure: BMI as a continous variable |
| Leung 2008 | "Wrong outcome: Only univariate (crude) estimate reported |
| Li 2020 | Wrong outcomes: no risks estimates (only incidence). |
| Li 2021 | Wrong exposure: Undernutrition is not assessed as a risk factor |
| Li 2022 | Wrong exposure: BMI <20 as cut-off point and diabetes only as a covariate |
| Liao 2016 | Wrong exposure: Undernutrition is not assessed as a risk factor |
| Lin 1998 | Wrong patient population: All participants had TB at baseline. |
| Lin 2019 | Wrong outcome: latent TB infection. |
| Liu 2020 | Wrong outcome: risk for TB infection, not active TB. |
| Lo 2016 | Wrong exposure: Undernutrition is not assessed as a risk factor |
| Lozada Muñoz 2018 | Wrong study design: case-control study. |
| Lu 2021 | Wrong exposure: wrong definition of undernutrition (bmi <24) |
| Ludes 1965 | Wrong study design: cross-sectional study. |
| Luk 2021 | Wrong exposure: Undernutrition is not assessed as a risk factor |
| Martinez 2017 | Wrong study design: cross-sectional study. |
| Martinez 2023 | Wrong exposure: weight modelled continously (not as underweight). |
| McAllister 2020 | Wrong exposure: all participants with diabetes, BMI <23 as cut-off point |
| McDermid 2013 | Wrong outcome: Only univariate (crude) estimate reported |
| Meinerz 2016 | Wrong outcome: no risk estimates (only p values). |
| Mor 2016 | Wrong exposure: Undernutrition is not assessed as a risk factor |
| Moreno Díaz 2011 | Wrong study design: ecological study. |
| Moura 2012 | Wrong outcome: repetition of tuberculin test. |
| Mujtaba 2022 | Wrong study design: case-control study. |
| Mupfumi 2018 | Wrong exposure: BMI as a continous variable |
| Naidoo 2022 | Wrong exposure: BMI as a continous variable. |
| Nakanjako 2010 | Wrong exposure: BMI <20 as cut-off point. |
| Nanda 1968 | Wrong patient population: All participants had TB at baseline. |
| Nanta 2011 | Wrong study design: cross-sectional study. |
| Narain 2018 | Wrong outcome: no risk estimates. |
| Ogbo 2018 | Wrong study design: estimation of burden of disease. |
| Olmos 1989 | Wrong exposures: all participants with diabetes, BMI not assessed. |
| Pan 2020 | Wrong exposures: all participants with diabetes, BMI not assessed. |
| Park 2019 | Wrong exposure: Undernutrition is not assessed as a risk factor (BMI as a continous variable) |
| Park 2022a | Wrong exposures: all participants with diabetes, BMI modelled but not as a risk factor. |
| Patra 2014 | Wrong outcome: symptoms related to TB (also cross-sectional). |
| Pearson 2019 | Wrong exposure: Undernutrition is not assessed as a risk factor |
| Pereira e Silva 1985 | Wrong study design: case report. |
| Pérez-Navarro 2011 | Wrong study design: case-control study. |
| Picon 2007 | Wrong exposure: Undernutrition is not assessed as a risk factor |
| Picon 2007a | Wrong exposure: no enough cases of TB to assess relevant risk factors. |
| Ponce-De-Leon 2004 | Wrong outcome: incidence rates in low BMI but no analysis. |
| Prakash 2013 | Wrong study design: cross-sectional study. |
| Prince 2016 | Wrong study design: cross-sectional study. |
| Qiu 2017 | Wrong exposures: all participants with diabetes, BMI modelled continously. |
| Rahim 2012 | Wrong study design: cross-sectional study. |
| Rajan 2017 | Wrong exposure: Undernutrition is not assessed as a risk factor |
| Ronald 2019 | Wrong exposures: no risk estimates (only incidence in groups). |
| Rueda 2013 | Wrong outcome: sputum conversion. |
| Salvadori 2017 | Wrong outcome: Only univariate (crude) estimate reported |
| Saunders 2017 | Wrong exposure: BMI as a continous variable |
| Saunders 2020 | Wrong exposure: BMI <20 as cut-off point. |
| Scordo 2021 | Wrong study design: cross-sectional study. |
| Shadrach 2021 | Wrong study design: cross-sectional study. |
| Shen 2014 | Wrong exposure: Undernutrition is not assessed as a risk factor |
| Sil 2021 | Wrong study design: cross-sectional study. |
| Singh 2016 | Wrong study design: cross-sectional study. |
| Sinha 2023 | Wrong outcome: treatment failure and relapse combined. |
| Skodrić-Trifunović 2004 | Wrong study design: review article. |
| Stein 2018 | Wrong outcome: the outcome 'secondary tuberculosis' includes co-prevalent TB. |
| Tao 2021 | Wrong outcome: resistance patterns of TB. |
| Tatar 2009 | Wrong patient population: All participants had TB at baseline. |
| Teng 2019 | Wrong exposure: Undernutrition is not assessed as a risk factor |
| Tian 2014 | Wrong study design: case-control study. |
| Tipayamongkholgul 2016 | Wrong exposure: Undernutrition is not assessed as a risk factor |
| Tverdal 1986 | Wrong exposure: BMI <21 as cut-off point. |
| Umeta 2022 | Wrong outcome: acceleration factor. |
| Velen 2021 | Wrong study design: case-control study. |
| Walker 2010 | Wrong study design: calculation of population attributable fraction from estimates from the literature |
| Wang 2019 | Wrong exposures: no risk estimates (only incidence in groups). |
| Watch 2017 | Wrong patient population: All participants had TB at baseline. |
| Wu 2015 | Wrong exposure: Undernutrition is not assessed as a risk factor |
| Yang 2018 | Wrong exposures: no diabetes or BMI as risk factors. |
| Yoo 2021b | Wrong exposure: Undernutrition is not assessed as a risk factor (BMI as a continous variable) |
| Young 2012 | Wrong exposure: Undernutrition is not assessed as a risk factor |
| Zahr 2016 | Wrong study design: cross-sectional study. |
| Zakopaĭlo 1991 | Wrong study design: review article. |

## Footnotes

**BMI:** Body Mass Index. **TB:** Tuberculosis

## References to studies

### Aachari 2022 {published data only}

- Aachari I, Tahiri L, Elolemy G, Taik FZ, Afilal S, Fellous S, et al. Active tuberculosis infection in Moroccan patients with rheumatic diseases under biologic therapy: a multicenter national study. International Journal of Mycobacteriology 2022;11(2):175-82. [DOI: 10.4103/ijmy.ijmy\_153\_20]

### Abedi 2019 {published data only}

- Abedi S, Moosazadeh M, Tabrizi R, Afshari M, Nezammahalleh A, Akbari M. The impact of diabetics and smoking on gender differences of smear positive pulmonary tuberculosis incidence. Indian Journal of Tuberculosis 2019;66(3):353-7. [DOI: 10.1016/j.ijtb.2017.01.004]

### Adem 2020 {published data only}

- Adem F, Edessa D, Bayissa B, Mohammed Hassen M, Mohammed MA. Treatment outcomes and associated factors in hospitalised children with severe acute malnutrition: a prospective cohort study. Pediatric Health, Medicine and Therapeutics 2020;11:235-43. [DOI: 10.2147/phmt.S253396]

### Afshari 2023 {published data only}

- Afshari M, Dehmardeh A, Hoseini A, Moosazadeh M. Tuberculosis infection among children under six in contact with smear positive cases: a study in a hyper endemic area of Iran. Journal of Clinical Tuberculosis and Other Mycobacterial Diseases 2023;30:100347. [DOI: 10.1016/j.jctube.2023.100347]

### Agarwal 2017 {published data only}

- Agarwal SK, Bhowmik D, Mahajan S, Bagchi S. Impact of type of calcineurin inhibitor on post-transplant tuberculosis: single-center study from India. Transplant Infectious Disease 2017;19(1):Epub 2016 Dec 16. [DOI: 10.1111/tid.12626]

### Aguilera 2016 {published data only}

- Aguilera XP, González C, Nájera-De Ferrari M, Hirmas M, Delgado I, Olea A, et al. Tuberculosis in prisoners and their contacts in Chile: estimating incidence and latent infection. International Journal of Tuberculosis and Lung Disease 2016;20(1):63-70. [DOI: 10.5588/ijtld.15.0056]

### Al Kubaisy 2003 {published data only}

- Al Kubaisy W, Al Dulayme A, Hashim DS. Active tuberculosis among Iraqi schoolchildren with positive skin tests and their household contacts. Eastern Mediterranean Health Journal 2003;9(4):675-88.

### Allen 1992 {published data only}

- Allen S, Batungwanayo J, Kerlikowske K, Lifson AR, Wolf W, Granich R, et al. Two-year incidence of tuberculosis in cohorts of HIV-infected and uninfected urban Rwandan women. American Review of Respiratory Disease 1992;146(6):1439-44. [DOI: 10.1164/ajrccm/146.6.1439]

### Ambrosetti 1999 {published data only}

- Ambrosetti M, Besozzi G, Codecasa LR, Farris B, Nutini S, Saini L, et al. The Italian AIPO study on tuberculosis treatment results, report 1997. National AIPO "Tuberculosis" Study Group. Monaldi Archives for Chest Disease 1999;54(5):407-12.
- Ambrosetti M, Besozzi G, Farris B, Nutini S, Saini L, Casali L, et al. The Italian AIPO study on tuberculosis treatment results, report 1996. National AIPO "Tuberculosis" Study Group. Associazione Italiana Pneumologi Ospedalieri. Monaldi Archives for Chest Disease 1999;54(3):237-41.

### Anaam 2023 {published data only}

- Anaam MS, Alrasheedy AA. Recurrence rate of pulmonary tuberculosis in patients treated with the standard 6-month regimen: findings and implications from a prospective observational multicenter study. Tropical Medicine and Infectious Disease 2023;8(2):110. [DOI: 10.3390/tropicalmed8020110]

### Antonio-Arques 2022 {published data only}

- Antonio-Arques V, Franch-Nadal J, Moreno-Martinez A, Real J, Orcau À, Mauricio D, et al. Subjects with diabetes mellitus are at increased risk for developing tuberculosis: a cohort study in an inner-city district of Barcelona (Spain). Frontiers in Public Health 2022;10:789952. [DOI: 10.3389/fpubh.2022.789952]

### Anyalechi 2022 {published data only}

- Anyalechi GE, Bain R, Kindra G, Mogashoa M, Sogaula N, Mutiti A, et al. Tuberculosis prevalence, incidence and prevention in a South African cohort of children living with HIV. Journal of Tropical Pediatrics 2022;68(6):fmac084. [DOI: 10.1093/tropej/fmac084]

### Arriaga 2021 {published data only}

- Arriaga MB, Rocha MS, Nogueira BMF, Nascimento V, Araújo-Pereira M, Souza AB, et al. The effect of diabetes and prediabetes on mycobacterium tuberculosis transmission to close contacts. Journal of Infectious Diseases 2021;224(12):2064-72. [DOI: 10.1093/infdis/jiab264]

### Auguste 2021 {published data only}

- Auguste M, McGuire-Wolfe C, Alonso A, Okobi OE. Analysis of some risk factors of active tuberculosis in three South Florida counties. Cureus 2021;13(11):e19852. [DOI: 10.7759/cureus.19852]

### Auld 2014 {published data only}

- Auld AF, Tuho MZ, Ekra KA, Kouakou J, Shiraishi RW, Adjorlolo-Johnson G, et al. Tuberculosis in human immunodeficiency virus-infected children starting antiretroviral therapy in Côte d'Ivoire. International Journal of Tuberculosis and Lung Disease 2014;18(4):381-7. [DOI: 10.5588/ijtld.13.0395]

### Auld 2021 {published data only}

- Auld AF, Kerkhoff AD, Hanifa Y, Wood R, Charalambous S, Liu Y, et al. Derivation and external validation of a risk score for predicting HIV-associated tuberculosis to support case finding and preventive therapy scale-up: a cohort study. PLOS Medicine 2021;18(9):e1003739. [DOI: 10.1371/journal.pmed.1003739]

### Bai 2016 {published data only}

- Bai KJ, Lee JJ, Chien ST, Suk CW, Chiang CY. The influence of smoking on pulmonary tuberculosis in diabetic and non-diabetic patients. PLOS One 2016;11(6):e0156677. [DOI: 10.1371/journal.pone.0156677]

### Benoit 2017 {published data only}

- Benoit SR, Gregg EW, Jonnalagadda S, Phares CR, Zhou W, Painter JA. Association of diabetes and tuberculosis disease among US-bound adult refugees, 2009-2014. Emerging Infectious Diseases 2017;23(3):543-5. [DOI: 10.3201/eid2303.161053]

### Bhargava 2014 {published data only}

- Bhargava A, Benedetti A, Oxlade O, Pai M, Menzies D. Undernutrition and the incidence of tuberculosis in India: national and subnational estimates of the population-attributable fraction related to undernutrition. National Medical Journal of India 2014;27(3):128-33.

### Bridson 2015 {published data only}

- Bridson T, Matthiesson A, Owens L, Govan B, Norton R, Ketheesan N. Diabetes: a contributor to tuberculosis in tropical Australia. American Journal of Tropical Medicine and Hygiene 2015;93(3):547-8. [DOI: 10.4269/ajtmh.15-0264]

### Byberg 2012 {published data only}

- Byberg S, Soborg B, Andersson M, Bjerregaard P, Jørgensen ME. Diabetes is a risk factor for tuberculosis in the Inuit population of Greenland. European Respiratory Journal 2012;40(5):1289-91. [DOI: 10.1183/09031936.00039612]

### Campbell 2022 {published data only}

- Campbell JR, Chan ED, Falzon D, Trajman A, Keshavjee S, Leung CC, et al. Low body mass index at treatment initiation and rifampicin-resistant tuberculosis treatment outcomes: an individual participant data meta-analysis. Clinical Infectious Diseases 2022;75(12):2201-10. [DOI: 10.1093/cid/ciac322]

### Centis 2000 {published data only}

- Centis R, Ianni A, Migliori GB. Evaluation of tuberculosis treatment results in Italy, report 1998. Tuberculosis section of the National AIPO Study Group on Infectious Disease and the SMIRA Group. Monaldi Archives for Chest Disease 2000;55(4):293-8.

### Centis 2002 {published data only}

- Centis R, Migliori GB. Evaluation of tuberculosis treatment results in Italy, report 1999. Monaldi Archives for Chest Disease 2002;57(5-6):297-305.

### Chao 2016 {published data only}

- Chao WC, Wu CL, Liu PY, Shieh CC. Regular sputum check-up for early diagnosis of tuberculosis after exposure in healthcare facilities. PLOS One 2016;11(6):e0157054. [DOI: 10.1371/journal.pone.0157054]

### Chen 2006 {published data only}

- Chen CH, Lian JD, Cheng CH, Wu MJ, Lee WC, Shu KH. Mycobacterium tuberculosis infection following renal transplantation in Taiwan. Transplant Infectious Disease 2006;8(3):148-56. [DOI: 10.1111/j.1399-3062.2006.00147.x]

### Chen 2013 {published data only}

- Chen W, Shu W, Wang M, Hou Y, Xia Y, Xu W, et al. Pulmonary tuberculosis incidence and risk factors in rural areas of China: a cohort study. PLOS One 2013;8(3):e58171. [DOI: 10.1371/journal.pone.0058171]

### Chen 2014 {published data only}

- Chen CH, Shu KH, Ho HC, Cheng SB, Lin CC, Wei HJ, et al. A nationwide population-based study of the risk of tuberculosis in different solid organ transplantations in Taiwan. Transplantation Proceedings 2014;46(4):1032-5. [DOI: 10.1016/j.transproceed.2013.10.051]

### Cheng 2018 {published data only}

- Cheng KC, Liao KF, Lin CL, Liu CS, Lai SW. Chronic kidney disease correlates with increased risk of pulmonary tuberculosis before initiating renal replacement therapy: a cohort study in Taiwan. Medicine 2018;97(39):e12550. [DOI: 10.1097/md.0000000000012550]

### Cheng 2020a {published data only}

- Cheng Q, Trangucci R, Nelson KN, Fu W, Collender PA, Head JR, et al. Prenatal and early-life exposure to the Great Chinese Famine increased the risk of tuberculosis in adulthood across two generations. Proceedings of the National Academy of Sciences of the United States of America 2020;117(44):27549-55. [DOI: 10.1073/pnas.2008336117]

### Cherkezov 1973 {published data only}

- Cherkezov R. The incidence of tuberculosis in diabetic patients in the city of Plovdiv. Vutreshni Bolesti 1973;12(5):158-60.

### Christopoulos 2009 {published data only}

- Christopoulos AI, Diamantopoulos AA, Dimopoulos PA, Goumenos DS, Barbalias GA. Risk factors for tuberculosis in dialysis patients: a prospective multi-center clinical trial. BMC Nephrology 2009;10:36. [DOI: 10.1186/1471-2369-10-36]

### Chu 2019 {published data only}

- Chu KA, Hsu CH, Lin MC, Chu YH, Hung YM, Wei JC. Association of iron deficiency anemia with tuberculosis in Taiwan: a nationwide population-based study. PLOS One 2019;14(8):e0221908. [DOI: 10.1371/journal.pone.0221908]

### Chung 2014 {published data only}

- Chung WS, Lin CL, Hung CT, Chu YH, Sung FC, Kao CH, et al. Tuberculosis increases the subsequent risk of acute coronary syndrome: a nationwide population-based cohort study. International Journal of Tuberculosis and Lung Disease 2014;18(1):79-83. [DOI: 10.5588/ijtld.13.0288]

### Chung-Delgado 2012 {published data only}

- Chung-Delgado K, Guillén-Bravo S, Navarro-Huamán L, Quiroz-Portella R, Revilla-Montag A, Ruíz-Alejos A, et al. Medical students at risk: prevalence and incidence of tuberculin skin test conversion. Revista Chilena de Infectologia 2012;29(4):375-81. [DOI: 10.4067/s0716-10182012000400002]

### Ciang 2020 {published data only}

- Ciang NC, Chan SCW, Lau CS, Chiu ETF, Chung HY. Risk of tuberculosis in patients with spondyloarthritis: data from a centralized electronic database in Hong Kong. BMC Musculoskeletal Disorders 2020;21(1):832. [DOI: 10.1186/s12891-020-03855-5]

### Critchley 2018 {published data only}

- Critchley JA, Carey IM, Harris T, DeWilde S, Hosking FJ, Cook DG. Glycemic control and risk of infections among people with type 1 or type 2 diabetes in a large primary care cohort study. Diabetes Care 2018;41(10):2127-35. [DOI: 10.2337/dc18-0287]

### DeAtley 2021 {published data only}

- DeAtley T, Workman L, Theron G, Bélard S, Prins M, Bateman L, et al. The child ecosystem and childhood pulmonary tuberculosis: A South African perspective. Pediatric Pulmonology 2021;56(7):2212-22. [DOI: 10.1002/ppul.25369]

### Delgado-Sánchez 2015 {published data only}

- Delgado-Sánchez G, García-García L, Castellanos-Joya M, Cruz-Hervert P, Ferreyra-Reyes L, Ferreira-Guerrero E, et al. Association of pulmonary tuberculosis and diabetes in Mexico: analysis of the National Tuberculosis Registry 2000-2012. PLOS One 2015;10(6):e0129312. [DOI: 10.1371/journal.pone.0129312]

### Demlow 2015 {published data only}

- Demlow SE, Oh P, Barry PM. Increased risk of tuberculosis among foreign-born persons with diabetes in California, 2010-2012. BMC Public Health 2015;15:263. [DOI: 10.1186/s12889-015-1600-1]

### Dobler 2012 {published data only}

- Dobler CC, Flack JR, Marks GB. Risk of tuberculosis among people with diabetes mellitus: an Australian nationwide cohort study. BMJ Open 2012;2(1):e000666. [DOI: 10.1136/bmjopen-2011-000666]

### Dyck 2007 {published data only}

- Dyck RF, Klomp H, Marciniuk DD, Tan L, Stang MR, Ward HA, et al. The relationship between diabetes and tuberculosis in Saskatchewan: comparison of registered Indians and other Saskatchewan people. Canadian Journal of Public Health 2007;98(1):55-9. [DOI: 10.1007/bf03405386]

### Eksombatchai 2023 {published data only}

- Eksombatchai D, Jeong D, Mok J, Jeon D, Kang HY, Kim HJ, et al. Sex differences in the impact of diabetes mellitus on tuberculosis recurrence: a retrospective national cohort study. International Journal of Infectious Diseases 2023;127:1-10. [DOI: 10.1016/j.ijid.2022.11.037]

### Eswarappa 2020 {published data only}

- Eswarappa M, H JGd, John MM, Chennabasappa GK, Siddaiah GM. Tuberculosis in renal transplant recipients: our decade long experience with an opportunistic invader. Indian Journal of Tuberculosis 2020;67(1):73-8. [DOI: 10.1016/j.ijtb.2019.05.001]

### Eun-Kyoung 2010 {published data only}

- Eun-Kyoung Park, June-Ho Bae, Yu-Min Jung, Sook-Hee Chung, Jae-Hyung Lee, Sang-Hoon Kim, et al. Incidence of tuberculosis in Korean diabetics: comparison with that in non-diabetic hypertensive subjects. Korean Journal of Medicine 2010;79(6):646-51.

### Fang 2015 {published data only}

- Fang WL, Hung YP, Liu CJ, Lan YT, Huang KH, Chen MH, et al. Incidence of and risk factors for tuberculosis (TB) in gastric cancer patients in an area endemic for TB: a nationwide population-based matched cohort study. Medicine 2015;94(47):e2163. [DOI: 10.1097/md.0000000000002163]

### Fikrie 2019 {published data only}

- Fikrie A, Alemayehu A, Gebremedhin S. Treatment outcomes and factors affecting time-to-recovery from severe acute malnutrition in 6-59 months old children admitted to a stabilization center in Southern Ethiopia: A retrospective cohort study. Italian Journal of Pediatrics 2019;45(1):46. [DOI: 10.1186/s13052-019-0642-x]

### Franke 2013 {published data only}

- Franke MF, Appleton SC, Mitnick CD, Furin JJ, Bayona J, Chalco K, et al. Aggressive regimens for multidrug-resistant tuberculosis reduce recurrence. Clinical Infectious Diseases 2013;56(6):770-6. [DOI: 10.1093/cid/cis1008]

### Frans 2020 {published data only}

- Sitepu FY, Aditama W, Depari E. Having contact history with TB active cases and malnutrition as risk factors of TB incidence: a cross-sectional study in North Sumatera, Indonesia. Malaysian Journal of Public Health Medicine 2020;20(1):192-8. [DOI: 10.37268/mjphm/vol.20/no.1/art.482]

### Golub 2019 {published data only}

- Golub JE, Mok Y, Hong S, Jung KJ, Jee SH, Samet JM. Diabetes mellitus and tuberculosis in Korean adults: impact on tuberculosis incidence, recurrence and mortality. International Journal of Tuberculosis and Lung Disease 2019;23(4):507-13. [DOI: 10.5588/ijtld.18.0103]

### Hermosilla 2017 {published data only}

- Hermosilla S, You P, Aifah A, Abildayev T, Akilzhanova A, Kozhamkulov U, et al. Identifying risk factors associated with smear positivity of pulmonary tuberculosis in Kazakhstan. PLOS One 2017;12(3):e0172942. [DOI: 10.1371/journal.pone.0172942]

### Herzmann 2017 {published data only}

- Herzmann C, Sotgiu G, Bellinger O, Diel R, Gerdes S, Goetsch U, et al. Risk for latent and active tuberculosis in Germany. Infection 2017;45(3):283-90. [DOI: 10.1007/s15010-016-0963-2]

### Hoge 1994 {published data only}

- Hoge CW, Fisher L, Donnell HDJr, Dodson DR, Tomlinson GVJr, Breiman RF, et al. Risk factors for transmission of Mycobacterium tuberculosis in a primary school outbreak: lack of racial difference in susceptibility to infection. American Journal of Epidemiology 1994;139(5):520-30. [DOI: 10.1093/oxfordjournals.aje.a117035]

### Hoon 2006 {published data only}

- Hoon Jung, Yeon-Mok Oh, Sang-Do Lee, Woo-Sung Kim, Dong-Soon Kim, Won-Dong Kim, et al. Clinical characteristics of tuberculosis in liver or heart transplant recipients. Tuberculosis and Respiratory Diseases 2006;61(5):440-6. [DOI: 10.4046/trd.2006.61.5.440]

### Hoshino 2009 {published data only}

- Hoshino H, Uchimura K, Yamauchi Y. Comparison of TB incidence of young and middle age groups between urban/suburban prefectures and other prefectures. Kekkaku [Tuberculosis] 2009;84(1):1-8.

### Hsien 2017 {published data only}

- Hsien Feng Lin, Kuan Fu Liao, Ching Mei Chang, Shih Wei Lai, Pang Yao Tsai, Fung Chang Sung. Anti-diabetic medication reduces risk of pulmonary tuberculosis in diabetic patients: a population-based cohort study in Taiwan. Kuwait Medical Journal 2017;49:22-8.

### Hu 2014 {published data only}

- Hu HY, Wu CY, Huang N, Chou YJ, Chang YC, Chu D. Increased risk of tuberculosis in patients with end-stage renal disease: a population-based cohort study in Taiwan, a country of high incidence of end-stage renal disease. Epidemiology and Infection 2014;142(1):191-9. [DOI: 10.1017/s0950268813000551]

### Ilievska-Poposka 2018 {published data only}

- Ilievska-Poposka B, Zakoska M, Pilovska-Spasovska K, Simonovska L, Mitreski V. Tuberculosis in the prisons in the Republic of Macedonia, 2008-2017. Open Access Macedonian Journal of Medical Sciences 2018;6(7):1300-4. [DOI: 10.3889/oamjms.2018.281]

### Jayalakshmi 1958 {published data only}

- Jayalakshmi VT, Gopalan C. Nutrition and tuberculosis. I. An epidemiological study. Indian Journal of Medical Research 1958;46(1):87-92. [PMID: 13501881]

### Ji 2020 {published data only}

- Ji Y, Cao H, Liu Q, Li Z, Song H, Xu D, et al. Screening for pulmonary tuberculosis in high-risk groups of diabetic patients. International Journal of Infectious Diseases 2020;93:84-9. [DOI: 10.1016/j.ijid.2020.01.019]

### John 2001 {published data only}

- John GT, Shankar V, Abraham AM, Mukundan U, Thomas PP, Jacob CK. Risk factors for post-transplant tuberculosis. Kidney international 2001;60(3):1148-53. [DOI: 10.1046/j.1523-1755.2001.0600031148.x]

### Jurcev-Savicevic 2013 {published data only}

- Jurcev-Savicevic A, Mulic R, Ban B, Kozul K, Bacun-Ivcek L, Valic J, et al. Risk factors for pulmonary tuberculosis in Croatia: a matched case-control study. BMC Public Health 2013;13:991. [DOI: 10.1186/1471-2458-13-991]

### Ju-Young 2008 {published data only}

- Ju-Young Sung, Chi-Won Kim, Hyun-Hee Lee, Woo-Kyung Chung, Yeon-Ho Park, Jongwon Ha, et al. Clinical manifestations of mycobacterium tuberculosis infection after renal transplantation. Journal of the Korean Society for Transplantation 2008;22(1):58-65.

### Kamper-Jørgensen 2015 {published data only}

- Kamper-Jørgensen Z, Carstensen B, Norredam M, Bygbjerg IC, Andersen PH, Jørgensen ME. Diabetes-related tuberculosis in Denmark: effect of ethnicity, diabetes duration and year of diagnosis. International Journal of Tuberculosis and Lung Disease 2015;19(10):1169-75. [DOI: 10.5588/ijtld.14.0932]

### Kawatsu 2015 {published data only}

- Kawatsu L, Ishikawa N, Uchimura K. Risk groups for tuberculosis in Japan: analysis of relative risk and population attributable fraction. Kekkaku [Tuberculosis] 2015;90(3):395-400.

### Khan 2006 {published data only}

- Khan A, Sterling TR, Reves R, Vernon A, Horsburgh CR. Lack of weight gain and relapse risk in a large tuberculosis treatment trial. American Journal of Respiratory and Critical Care Medicine 2006;174(3):344-8. [DOI: 10.1164/rccm.200511-1834OC]

### Khanna 1968 {published data only}

- Khanna BK. Pulmonary tuberculosis and diabetes mellitus. Journal of the Indian Medical Association 1968;50(9):407-14. [PMID: 5666158]

### Kim 1995 {published data only}

- Kim SJ, Hong YP, Lew WJ, Yang SC, Lee EG. Incidence of pulmonary tuberculosis among diabetics. Tubercle and Lung Disease 1995;76(6):529-33. [DOI: 10.1016/0962-8479(95)90529-4]

### Kim 2014 {published data only}

- Kim HW, Park JK, Yang JA, Yoon YI, Lee EY, Song YW, et al. Comparison of tuberculosis incidence in ankylosing spondylitis and rheumatoid arthritis during tumor necrosis factor inhibitor treatment in an intermediate burden area. Clinical Rheumatology 2014;33(9):1307-12. [DOI: 10.1007/s10067-013-2387-z]

### Klote 2006 {published data only}

- Klote MM, Agodoa LY, Abbott KC. Risk factors for Mycobacterium tuberculosis in US chronic dialysis patients. Nephrology, Dialysis, Transplantation 2006;21(11):3287-92. [DOI: 10.1093/ndt/gfl488]

### Ko 2022 {published data only}

- Ko TH, Chang YC, Chang CH, Liao KC, Magee MJ, Lin HH. Prediabetes and risk of active tuberculosis: a cohort study from Northern Taiwan. International Journal of Epidemiology 2022;52(3):932-41. [DOI: 10.1093/ije/dyac214]

### Koesoemadinata 2017 {published data only}

- Koesoemadinata RC, McAllister SM, Soetedjo NNM, Febni Ratnaningsih D, Ruslami R, Kerry S, et al. Latent TB infection and pulmonary TB disease among patients with diabetes mellitus in Bandung, Indonesia. Transactions of the Royal Society of Tropical Medicine and Hygiene 2017;111(2):81-9. [DOI: 10.1093/trstmh/trx015]

### Ku 2019 {published data only}

- Ku SW, Jiamsakul A, Joshi K, Pasayan MKU, Widhani A, Chaiwarith R, et al. Cotrimoxazole prophylaxis decreases tuberculosis risk among Asian patients with HIV. Journal of the International AIDS Society 2019;22(3):e25264. [DOI: 10.1002/jia2.25264]

### Kubjane 2020 {published data only}

- Kubjane M, Berkowitz N, Goliath R, Levitt NS, Wilkinson RJ, Oni T. Tuberculosis, human immunodeficiency virus, and the association with transient hyperglycemia in periurban South Africa. Clinical Infectious Diseases 2020;71(4):1080-8. [DOI: 10.1093/cid/ciz928]

### Kufa 2016 {published data only}

- Kufa T, Chihota V, Mngomezulu V, Charalambous S, Verver S, Churchyard G, et al. The incidence of tuberculosis among HIV-positive individuals with high CD4 counts: implications for policy. BMC Infectious Diseases 2016;16:266. [DOI: 10.1186/s12879-016-1598-8]

### Kumar 2018 {published data only}

- Kumar D, Goel C, Bansal AK, Bhardwaj AK. Delineating the factors associated with recurrence of tuberculosis in programmatic settings of rural health block, Himachal Pradesh, India. Indian Journal of Tuberculosis 2018;65(4):303-7. [DOI: 10.1016/j.ijtb.2018.07.001]

### Kuo 2013 {published data only}

- Kuo MC, Lin SH, Lin CH, Mao IC, Chang SJ, Hsieh MC. Type 2 diabetes: an independent risk factor for tuberculosis: a nationwide population-based study. PLOS One 2013;8(11):e78924. [DOI: 10.1371/journal.pone.0078924]

### Kuo 2013a {published data only}

- Kuo SC, Chen YT, Li SY, Lee YT, Yang AC, Chen TL, et al. Incidence and outcome of newly-diagnosed tuberculosis in schizophrenics: a 12-year, nationwide, retrospective longitudinal study. BMC infectious Diseases 2013;13:351. [DOI: 10.1186/1471-2334-13-351]

### Laghari 2018 {published data only}

- Laghari M, Sulaiman SAS, Khan AH, Memon N. Epidemiology of tuberculosis and treatment outcomes among children in Pakistan: a 5 year retrospective study. PeerJ 2018;6:e5253. [DOI: 10.7717/peerj.5253]

### Lee 2008 {published data only}

- Lee MS, Leung CC, Kam KM, Wong MY, Leung MC, Tam CM, et al. Early and late tuberculosis risks among close contacts in Hong Kong. International Journal of Tuberculosis and Lung Disease 2008;12(3):281-7.

### Lee 2013 {published data only}

- Lee CH, Lee MC, Shu CC, Lim CS, Wang JY, Lee LN, et al. Risk factors for pulmonary tuberculosis in patients with chronic obstructive airway disease in Taiwan: a nationwide cohort study. BMC Infectious Diseases 2013;13:194. [DOI: 10.1186/1471-2334-13-194]

### Lee 2015 {published data only}

- Lee MY, Lin KD, Hsu WH, Chang HL, Yang YH, Hsiao PJ, et al. Statin, calcium channel blocker and Beta blocker therapy may decrease the incidence of tuberculosis infection in elderly Taiwanese patients with type 2 diabetes. International Journal of Molecular Sciences 2015;16(5):11369-84. [DOI: 10.3390/ijms160511369]

### Lee 2016 {published data only}

- Lee PH, Fu H, Lai TC, Chiang CY, Chan CC, Lin HH. Glycemic control and the risk of tuberculosis: a cohort study. PLOS Medicine 2016;13(8):e1002072. [DOI: 10.1371/journal.pmed.1002072]

### Leow 2014 {published data only}

- Leow MK, Dalan R, Chee CB, Earnest A, Chew DE, Tan AW, et al. Latent tuberculosis in patients with diabetes mellitus: prevalence, progression and public health implications. Experimental and Clinical Endocrinology & Diabetes 2014;122(9):528-32. [DOI: 10.1055/s-0034-1377044]

### Leung 2007a {published data only}

- Leung CC, Yew WW, Law WS, Tam CM, Leung M, Chung YW, et al. Smoking and tuberculosis among silicotic patients. European Respiratory Journal 2007;29(4):745-50. [DOI: 10.1183/09031936.00134706]

### Leung 2008 {published data only}

- Leung CC, Lam TH, Chan WM, Yew WW, Ho KS, Leung GM, et al. Diabetic control and risk of tuberculosis: a cohort study. American Journal of Epidemiology 2008;167(12):1486-94. [DOI: 10.1093/aje/kwn075]

### Li 2020 {published data only}

- Li Y, Guo J, Xia T, Wu F, Tian J, Cheng M, et al. Incidence of pulmonary tuberculosis in Chinese adults with type 2 diabetes: a retrospective cohort study in Shanghai. Scientific Reports 2020;10(1):8578. [DOI: 10.1038/s41598-020-65603-y]

### Li 2021 {published data only}

- Li CH, Chen HJ, Chen WC, Tu CY, Hsia TC, Hsu WH, et al. The risk of tuberculosis infection in non-dialysis chronic kidney disease patients. Frontiers in Medicine 2021;8:715010. [DOI: 10.3389/fmed.2021.715010]

### Li 2022 {published data only}

- Li H, Chee CBE, Geng T, Pan A, Koh WP. Joint associations of multiple lifestyle factors with risk of active tuberculosis in the population: the Singapore Chinese Health Study. Clinical Infectious Diseases 2022;75(2):213-20. [DOI: 10.1093/cid/ciab935]

### Liao 2016 {published data only}

- Liao TL, Lin CH, Chen YM, Chang CL, Chen HH, Chen DY. Different risk of tuberculosis and efficacy of isoniazid prophylaxis in rheumatoid arthritis patients with biologic therapy: a nationwide retrospective cohort study in Taiwan. PLOS One 2016;11(4):e0153217. [DOI: 10.1371/journal.pone.0153217]

### Lin 1998 {published data only}

- Lin S, Shen M, Sun Y. Epidemiological characteristics of tuberculosis patients complicated with diabetes in Shanghai. Zhonghua Jie He He Hu Xi Za Zhi = Zhonghua Jiehe He Huxi Zazhi [chinese Journal of Tuberculosis and Respiratory Diseases] 1998;21(8):504-6.

### Lin 2019 {published data only}

- Lin CH, Kuo SC, Hsieh MC, Ho SY, Su IJ, Lin SH, et al. Effect of diabetes mellitus on risk of latent TB infection in a high TB incidence area: a community-based study in Taiwan. BMJ Open 2019;9(10):e029948. [DOI: 10.1136/bmjopen-2019-029948]

### Liu 2020 {published data only}

- Liu Q, Lu P, Martinez L, Peng H, Zhu T, Zhu L, et al. Undiagnosed diabetes mellitus and tuberculosis infection: a population-based, observational study from eastern China. Diabetes/Metabolism Research and Reviews 2020;36(3):e3227. [DOI: 10.1002/dmrr.3227]

### Lo 2016 {published data only}

- Lo HY, Yang SL, Lin HH, Bai KJ, Lee JJ, Lee TI, Chiang CY. Does enhanced diabetes management reduce the risk and improve the outcome of tuberculosis? International Journal of Tuberculosis and Lung Disease 2016;20(3):376-82. [DOI: 10.5588/ijtld.15.0654]

### Lozada Muñoz 2018 {published data only}

- Lozada Muñoz RL, Rincón Ardila OI, Román RamÍrez Y. Asociación diabetes tipo 2 y tuberculosis pulmonar en pacientes de imsalud en la zona atalaya de cúcuta entre 2013 y 2015. Rev. Med 2018;26(2):7-14.

### Lu 2021 {published data only}

- Lu P, Zhang Y, Liu Q, Ding X, Kong W, Zhu L, Lu W. Association of BMI, diabetes, and risk of tuberculosis: a population-based prospective cohort. International Journal of Infectious Diseases 2021;109:168-73. [DOI: 10.1016/j.ijid.2021.06.053]

### Ludes 1965 {published data only}

- Ludes H, Pappas A. On the incidence of manifest and latent diabetes mellitus in tuberculosis. Munchener Medizinische Wochenschrift (1950) 1965;107(27):1344-9.

### Luk 2021 {published data only}

- Luk AOY, Wu H, Lau ESH, Yang A, So WY, Chow E, et al. Temporal trends in rates of infection-related hospitalisations in Hong Kong people with and without diabetes, 2001-2016: a retrospective study. Diabetologia 2021;64(1):109-18. [DOI: 10.1007/s00125-020-05286-2]

### Martinez 2017 {published data only}

- Martinez L, Zhu L, Castellanos ME, Liu Q, Chen C, Hallowell BD, et al. Glycemic control and the prevalence of tuberculosis infection: a population-based observational study. Clinical Infectious Diseases 2017;65(12):2060-8. [DOI: 10.1093/cid/cix632]

### Martinez 2023 {published data only}

- Martinez L, Gray DM, Botha M, Nel M, Chaya S, Jacobs C, et al. The long-term impact of early-life tuberculosis disease on child health: a prospective birth cohort study. American Journal of Respiratory and Critical Care Medicine 2023;207(8):1080-8. [DOI: 10.1164/rccm.202208-1543OC]

### McAllister 2020 {published data only}

- McAllister SM, Koesoemadinata RC, Santoso P, Soetedjo NNM, Kamil A, Permana H, et al. High tuberculosis incidence among people living with diabetes in Indonesia. Transactions of the Royal Society of Tropical Medicine and Hygiene 2020;114(2):79-85. [DOI: 10.1093/trstmh/trz100]

### McDermid 2013 {published data only}

- McDermid JM, Hennig BJ, van der Sande M, Hill AV, Whittle HC, Jaye A, et al. Host iron redistribution as a risk factor for incident tuberculosis in HIV infection: an 11-year retrospective cohort study. BMC Infectious Diseases 2013;13:48. [DOI: 10.1186/1471-2334-13-48]

### Meinerz 2016 {published data only}

- Meinerz G, da Silva CK, Goldani JC, Garcia VD, Keitel E. Epidemiology of tuberculosis after kidney transplantation in a developing country. Transplant Infectious Disease 2016;18(2):176-82. [DOI: 10.1111/tid.12501]

### Mor 2016 {published data only}

- Mor A, Berencsi K, Nielsen JS, Rungby J, Friborg S, Brandslund I, et al. Rates of community-based antibiotic prescriptions and hospital-treated infections in individuals with and without type 2 diabetes: a Danish nationwide cohort study, 2004-2012. Clinical Infectious Diseases 2016;63(4):501-11. [DOI: 10.1093/cid/ciw345]

### Moreno Díaz 2011 {published data only}

- Moreno Díaz EN, González Cruz R, Sotolongo Castillo A, Martínez Porras M, González Pérez M. Estratificación epidemiológica en el control de los factores de riesgo de la tuberculosis. Rev. cienc. med. Pinar Rio 2011;15(4):62-74.

### Moura 2012 {published data only}

- Moura LC, Ximenes RA, Lacerda HR, Miranda-Filho DB, Barbosa MT, Byington MR, et al. Predictive factors for repetition of the tuberculin test after a nonreactive test in patients with HIV/AIDS. Revista Panamericana de Salud Publica 2012;31(2):121-8. [DOI: 10.1590/s1020-49892012000200005]

### Mujtaba 2022 {published data only}

- Mujtaba MA, Richardson M, Shahzad H, Javed MI, Raja GK, Shaiq PA, et al. Demographic and clinical determinants of tuberculosis and TB recurrence: a double-edged retrospective study from Pakistan. Journal of Tropical Medicine 2022;2022:4408306. [DOI: 10.1155/2022/4408306]

### Mupfumi 2018 {published data only}

- Mupfumi L, Moyo S, Molebatsi K, Thami PK, Anderson M, Mogashoa T, et al. Immunological non-response and low hemoglobin levels are predictors of incident tuberculosis among HIV-infected individuals on Truvada-based therapy in Botswana. PLOS One 2018;13(1):e0192030. [DOI: 10.1371/journal.pone.0192030]

### Naidoo 2022 {published data only}

- Naidoo K, Moodley MC, Hassan-Moosa R, Dookie N, Yende-Zuma N, Perumal R, et al. Recurrent subclinical tuberculosis among antiretroviral therapy-accessing participants: incidence, clinical course, and outcomes. Clinical Infectious Diseases 2022;75(9):1628-36. [DOI: 10.1093/cid/ciac185]

### Nakanjako 2010 {published data only}

- Nakanjako D, Mayanja-Kizza H, Ouma J, Wanyenze R, Mwesigire D, Namale A, et al. Tuberculosis and human immunodeficiency virus co-infections and their predictors at a hospital-based HIV/AIDS clinic in Uganda. International Journal of Tuberculosis and Lung Disease 2010;14(12):1621-8.

### Nanda 1968 {published data only}

- Nanda CN, Tripathy SN. Association of diabetes mellitus with pulmonary tuberculosis. Journal of the Association of Physicians of India 1968;16(10):741-6. [PMID: 5715499]

### Nanta 2011 {published data only}

- Nanta S, Kantipong P, Pathipvanich P, Ruengorn C, Tawichasri C, Patumanond J. Screening scheme development for active TB prediction among HIV-infected patients. Southeast Asian Journal of Tropical Medicine and Public Health 2011;42(4):867-75.

### Narain 2018 {published data only}

- Narain U, Gupta A. Incidence of tuberculosis in nondialysis-requiring CKD patients. International Journal of Advances in Medicine 2018;5(1):141-4. [DOI: 10.18203/2349-3933.ijam20180073]

### Ogbo 2018 {published data only}

- Ogbo FA, Ogeleka P, Okoro A, Olusanya BO, Olusanya J, Ifegwu IK, et al. Tuberculosis disease burden and attributable risk factors in Nigeria, 1990-2016. Tropical Medicine and Health 2018;46:34. [DOI: 10.1186/s41182-018-0114-9]

### Olmos 1989 {published data only}

- Olmos P, Donoso J, Rojas N, Landeros P, Schurmann R, Retamal G, et al. Tuberculosis and diabetes mellitus: a longitudinal-retrospective study in a teaching hospital. Revista Medica de Chile 1989;117(9):979-83.

### Pan 2020 {published data only}

- Pan SW, Feng JY, Yen YF, Chuang FY, Shen HS, Su VY, et al. Metformin use and post-exposure incident tuberculosis: a nationwide tuberculosis-contact cohort study in Taiwan. ERJ Open Research 2020;6(3):00050-2020. [DOI: 10.1183/23120541.00050-2020]

### Park 2019 {published data only}

- Park S, Lee S, Kim Y, Lee Y, Kang MW, Cho S, et al. Association of CKD with incident tuberculosis. Clinical Journal of the American Society of Nephrology 2019;14(7):1002-10. [DOI: 10.2215/cjn.14471218]

### Park 2022a {published data only}

- Park J, Yoon JH, Ki HK, Han K, Kim H. Lifestyle changes and risk of tuberculosis in patients with type 2 diabetes mellitus: a nationwide cohort study. Frontiers in Endocrinology 2022;13:1009493. [DOI: 10.3389/fendo.2022.1009493]

### Patra 2014 {published data only}

- Patra J, Jha P, Rehm J, Suraweera W. Tobacco smoking, alcohol drinking, diabetes, low body mass index and the risk of self-reported symptoms of active tuberculosis: individual participant data (IPD) meta-analyses of 72,684 individuals in 14 high tuberculosis burden countries. PLOS One 2014;9(5):e96433. [DOI: 10.1371/journal.pone.0096433]

### Pearson 2019 {published data only}

- Pearson F, Huangfu P, McNally R, Pearce M, Unwin N, Critchley JA. Tuberculosis and diabetes: bidirectional association in a UK primary care data set. Journal of Epidemiology and Community Health 2019;73(2):142-7. [DOI: 10.1136/jech-2018-211231]

### Pereira e Silva 1985 {published data only}

- Pereira e Silva JL, Araujo Neto CA, Ramos EAG. Tuberculose pulmonar de apresentaçäo atípica e diabetes mellitus. Jornal Brasileiro de Pneumologia 1985;11(3):169-74.

### Pérez-Navarro 2011 {published data only}

- Pérez-Navarro LM, Fuentes-Domínguez F, Morales-Romero J, Zenteno-Cuevas R. Factors associated to pulmonary tuberculosis in patients with diabetes mellitus from Veracruz, México. Gaceta Medica de Mexico 2011;147(3):219-25.

### Picon 2007 {published data only}

- Picon PD, Bassanesi SL, Caramori ML, Ferreira RL, Jarczewski CA, Vieira PR. Risk factors for recurrence of tuberculosis. Jornal Brasileiro de Pneumologia 2007;33(5):572-8. [DOI: 10.1590/s1806-37132007000500013]

### Picon 2007a {published data only}

- Picon Pedro Dornelles, Bassanesi Sergio Luiz, Caramori Maria Luiza Avancini, Ferreira Roberto Luiz Targa, Jarczewski Carla Adriane, et al. Fatores de risco para a recidiva da tuberculose. Jornal Brasileiro De Pneumologia 2007;33(5):572-8.

### Ponce-De-Leon 2004 {published data only}

- Ponce-De-Leon A, Garcia-Garcia Md Mde L, Garcia-Sancho MC, Gomez-Perez FJ, Valdespino-Gomez JL, Olaiz-Fernandez G, et al. Tuberculosis and diabetes in southern Mexico. Diabetes Care 2004;27(7):1584-90. [DOI: 10.2337/diacare.27.7.1584]

### Prakash 2013 {published data only}

- Prakash BC, Ravish KS, Prabhakar B, Ranganath TS, Naik B, Satyanarayana S, et al. Tuberculosis-diabetes mellitus bidirectional screening at a tertiary care centre, South India. Public Health Action 2013;3 Suppl 1:S18-22. [DOI: 10.5588/pha.13.0032]

### Prince 2016 {published data only}

- Prince L, Andrews JR, Basu S, Goldhaber-Fiebert JD. Risk of self-reported symptoms or diagnosis of active tuberculosis in relationship to low body mass index, diabetes and their co-occurrence. Tropical Medicine & International Health 2016;21(10):1272-81. [DOI: 10.1111/tmi.12763]

### Qiu 2017 {published data only}

- Qiu H, Shi Y, Li Y, Shen X, Li R, Yang Q, Pan Q, Yan F. Incident rate and risk factors for tuberculosis among patients with type 2 diabetes: retrospective cohort study in Shanghai, China. Tropical Medicine & International Health 2017;22(7):830-8. [DOI: 10.1111/tmi.12884]

### Rahim 2012 {published data only}

- Rahim Z, Momi MS, Saha SK, Zaman K, Uddin KN, Jamil SN, et al. Pulmonary tuberculosis in patients with diabetes mellitus in Bangladesh. International Journal of Tuberculosis and Lung Disease 2012;16(8):1132-3. [DOI: 10.5588/ijtld.11.0846]

### Rajan 2017 {published data only}

- Rajan JV, Ferrazoli L, Waldman EA, Simonsen V, Ferreira P, Telles MA, et al. Diabetes increases the risk of recent-transmission tuberculosis in household contacts in São Paulo, Brazil. International Journal of Tuberculosis and Lung Disease 2017;21(8):916-21. [DOI: 10.5588/ijtld.16.0043]

### Ronald 2019 {published data only}

- Ronald LA, Campbell JR, Rose C, Balshaw R, Romanowski K, Roth DZ, et al. Estimated impact of World Health Organization latent tuberculosis screening guidelines in a region with a low tuberculosis incidence: retrospective cohort study. Clinical Infectious Diseases 2019;69(12):2101-8. [DOI: 10.1093/cid/ciz188]

### Rueda 2013 {published data only}

- Rueda ZV, López L, Vélez LA, Marín D, Giraldo MR, Pulido H, et al. High incidence of tuberculosis, low sensitivity of current diagnostic scheme and prolonged culture positivity in four the Colombian prisons. A cohort study. PLOS One 2013;8(11):e80592. [DOI: 10.1371/journal.pone.0080592]

### Salvadori 2017 {published data only}

- Salvadori N, Ngo-Giang-Huong N, Duclercq C, Kanjanavanit S, Ngampiyaskul C, Techakunakorn P, Puangsombat A, et al. Incidence of tuberculosis and associated mortality in a cohort of human immunodeficiency virus-infected children initiating antiretroviral therapy. Journal of the Pediatric Infectious Diseases Society 2017;6(2):161-7. [DOI: 10.1093/jpids/piw090]

### Saunders 2017 {published data only}

- Saunders MJ, Wingfield T, Tovar MA, Baldwin MR, Datta S, Zevallos K, et al. A score to predict and stratify risk of tuberculosis in adult contacts of tuberculosis index cases: a prospective derivation and external validation cohort study. The Lancet. Infectious Diseases 2017;17(11):1190-9. [DOI: 10.1016/s1473-3099(17)30447-4]

### Saunders 2020 {published data only}

- Saunders MJ, Wingfield T, Datta S, Montoya R, Ramos E, Baldwin MR, et al. A household-level score to predict the risk of tuberculosis among contacts of patients with tuberculosis: a derivation and external validation prospective cohort study. The Lancet. Infectious Diseases 2020;20(1):110-22. [DOI: 10.1016/s1473-3099(19)30423-2]

### Scordo 2021 {published data only}

- Scordo JM, Aguillón-Durán GP, Ayala D, Quirino-Cerrillo AP, Rodríguez-Reyna E, Mora-Guzmán F, et al. A prospective cross-sectional study of tuberculosis in elderly Hispanics reveals that BCG vaccination at birth is protective whereas diabetes is not a risk factor. PLOS One 2021;16(7):e0255194. [DOI: 10.1371/journal.pone.0255194]

### Shadrach 2021 {published data only}

- Shadrach BJ, Kumar S, Deokar K, Singh GV, Hariharan, Goel R. A study of multidrug resistant tuberculosis among symptomatic household contacts of MDR-TB patients. Indian Journal of Tuberculosis 2021;68(1):25-31. [DOI: 10.1016/j.ijtb.2020.09.030]

### Shen 2014 {published data only}

- Shen TC, Lin CL, Wei CC, Liao WC, Chen WC, Chen CH, et al. Increased risk of tuberculosis in patients with type 1 diabetes mellitus: results from a population-based cohort study in Taiwan. Medicine 2014;93(16):e96. [DOI: 10.1097/md.0000000000000096]

### Sil 2021 {published data only}

- Sil A, Patra D, Dhillon P, Narasimhan P. Co-existence of diabetes and TB among adults in India: a study based on National Family Health Survey data. Journal of Biosocial Science 2021;53(5):758-72. [DOI: 10.1017/s0021932020000516]

### Singh 2016 {published data only}

- Singh SP, Kishan J, Kaur S, Ramana S. Association of tuberculosis and diabetes mellitus: an analysis of 1000 consecutively admitted cases in a tertiary care hospital of North India. Pan African Medical Journal 2016;24:4. [DOI: 10.11604/pamj.2016.24.4.8153]

### Sinha 2023 {published data only}

- Sinha P, Ponnuraja C, Gupte N, Prakash Babu S, Cox SR, Sarkar S, et al. Impact of undernutrition on tuberculosis treatment outcomes in india: a multicenter, prospective, cohort analysis. Clinical Infectious Diseases 2023;76(8):1483-91. [DOI: 10.1093/cid/ciac915]

### Skodrić-Trifunović 2004 {published data only}

- Skodrić-Trifunović V. Risk factors for developing tuberculosis. Medicinski Pregled 2004;57 Suppl 1:53-8.

### Stein 2018 {published data only}

- Stein CM, Zalwango S, Malone LL, Thiel B, Mupere E, Nsereko M, et al. Resistance and susceptibility to Mycobacterium tuberculosis infection and disease in tuberculosis households in Kampala, Uganda. American Journal of Epidemiology 2018;187(7):1477-89. [DOI: 10.1093/aje/kwx380]

### Tao 2021 {published data only}

- Tao NN, Li YF, Song WM, Liu JY, Zhang QY, Xu TT, et al. Risk factors for drug-resistant tuberculosis, the association between comorbidity status and drug-resistant patterns: a retrospective study of previously treated pulmonary tuberculosis in Shandong, China, during 2004-2019. BMJ Open 2021;11(6):e044349. [DOI: 10.1136/bmjopen-2020-044349]

### Tatar 2009 {published data only}

- Tatar D, Senol G, Alptekin S, Karakurum C, Aydin M, Coskunol I. Tuberculosis in diabetics: features in an endemic area. Japanese Journal of Infectious Diseases 2009;62(6):423-7.

### Teng 2019 {published data only}

- Teng CJ, Huon LK, Zheng YM, Yeh CM, Tsai CK, Liu JH, et al. Increased risk of tuberculosis in oral cancer patients in an endemic area: a nationwide population-based study. Clinical oral investigations 2019;23(12):4223-31. [DOI: 10.1007/s00784-019-02864-6]

### Tian 2014 {published data only}

- Tian PW, Wang Y, Shen YC, Chen L, Wan C, Liao ZL, et al. Different risk factors of recurrent pulmonary tuberculosis between Tibetan and Han populations in Southwest China. European Review for Medical and Pharmacological Sciences 2014;18(10):1482-6.

### Tipayamongkholgul 2016 {published data only}

- Tipayamongkholgul M, Marin W, Sujirarat D, Pokaew P, Pungrassami P. Non-communicable diseases increased risk of recurrent tuberculosis in epidemic area of human immunodeficiency virus infection, Thailand. Tropical Biomedicine 2016;33(1):190-6.

### Tverdal 1986 {published data only}

- Tverdal A. Body mass index and incidence of tuberculosis. European Journal of Respiratory Diseases 1986;69(5):355-62.

### Umeta 2022 {published data only}

- Umeta AK, Yermosa SF, Dufera AG. Bayesian parametric modeling of time to tuberculosis co-infection of HIV/AIDS patients at Jimma Medical Center, Ethiopia. Scientific Reports 2022;12(1):16475. [DOI: 10.1038/s41598-022-20872-7]

### Velen 2021 {published data only}

- Velen K, Nhung NV, Anh NT, Cuong PD, Hoa NB, Cuong NK, et al. Risk factors for tuberculosis (TB) among household contacts of patients with smear-positive TB in 8 provinces of Vietnam: a nested case-control study. Clinical Infectious Diseases 2021;73(9):e3358-64. [DOI: 10.1093/cid/ciaa1742]

### Walker 2010 {published data only}

- Walker C, Unwin N. Estimates of the impact of diabetes on the incidence of pulmonary tuberculosis in different ethnic groups in England. Thorax 2010;65(7):578-81. [DOI: 10.1136/thx.2009.128223]

### Wang 2019 {published data only}

- Wang S. Development of a predictive model of tuberculosis transmission among household contacts. Canadian Journal of Infectious Diseases & Medical Microbiology 2019;2019:5214124. [DOI: 10.1155/2019/5214124]

### Watch 2017 {published data only}

- Watch V, Aipit J, Kote-Yarong T, Rero A, Bolnga JW, Lufele E, Laman M. The burden of presumed tuberculosis in hospitalized children in a resource-limited setting in Papua New Guinea: a prospective observational study. International Health 2017;9(6):374-8. [DOI: 10.1093/inthealth/ihx043]

### Wu 2015 {published data only}

- Wu PH, Lin YT, Yang YH, Lin YC. The increased risk of active tuberculosis disease in patients with dermatomyositis - a nationwide retrospective cohort study. Scientific Reports 2015;5:16303. [DOI: 10.1038/srep16303]

### Yang 2018 {published data only}

- Yang BR, Kang YA, Heo EY, Koo BK, Choi NK, Hwang SS, et al. Regional differences in the incidence of tuberculosis among patients with newly diagnosed diabetes mellitus. Clinical Respiratory Journal 2018;12(4):1732-8. [DOI: 10.1111/crj.12737]

### Yoo 2021b {published data only}

- Yoo JE, Kim D, Han K, Rhee SY, Shin DW, Lee H. Diabetes status and association with risk of tuberculosis among Korean adults. JAMA Network Open 2021;4(9):e2126099. [DOI: 10.1001/jamanetworkopen.2021.26099]

### Young 2012 {published data only}

- Young F, Wotton CJ, Critchley JA, Unwin NC, Goldacre MJ. Increased risk of tuberculosis disease in people with diabetes mellitus: record-linkage study in a UK population. Journal of Epidemiology and Community Health 2012;66(6):519-23. [DOI: 10.1136/jech.2010.114595]

### Zahr 2016 {published data only}

- Zahr RS, Peterson RA, Polgreen LA, Cavanaugh JE, Hornick DB, Winthrop KL, et al. Diabetes as an increasingly common comorbidity among patient hospitalizations for tuberculosis in the USA. BMJ Open Diabetes Research & Care 2016;4(1):e000268. [DOI: 10.1136/bmjdrc-2016-000268]

### Zakopaĭlo 1991 {published data only}

- Zakopaĭlo GG, Khoshaba AM, Primakovskiĭ VP, Regan SN. Causes of recurrences of pulmonary tuberculosis. Problemy Tuberkuleza 1991;(8):74-5.
