# Supplementary material for: Undernutrition as a risk factor for tuberculosis disease
Source: Cochrane Database Syst Rev. 2024 Jun 11;2024(6):CD015890. doi: 10.1002/14651858.CD015890.pub2 (PMC11165671; doi:10.1002/14651858.CD015890.pub2)
Supplement: Supplementary file 4 — Supplementary material 4 Characteristics of studies awaiting classification [file CD015890-SUP-04-characteristicsOfAwaitingStudies.html]

Characteristics of studies awaiting classification


# Supplementary material 4 to: Undernutrition as a risk factor for tuberculosis disease

Franco JVA, Bongaerts B, Metzendorf MI, Risso A, Guo Y, Peña Silva L, Boeckmann M, Schlesinger S, Damen JAAG, Richter B, Baddeley A, Bastard M, Carlqvist A, Garcia-Casal MN, Hemmingsen B, Mavhunga F, Manne-Goehler J, Viney K
  
https://doi.org/10.1002/14651858.CD015890.pub2

The material in this section has been supplied by the author(s) for publication under a Licence for Publication and the author(s) are solely responsible for the material. Cochrane has reviewed this material, but Cochrane has not copyedited, formatted or proofread. Cochrane accordingly gives no representations or warranties of any kind in relation to, and accepts no liability for any reliance on or use of, such material.

Back to top

# Characteristics of studies awaiting classification

## Table of contents

- Studies ordered by Study ID
  - Nigam 1984
  - Schulz 1986
  - Silwer 1958
  - Trauth 1980
- References to studies

## Studies ordered by Study ID

Nigam 1984

| Notes | Full-text not available (no abstract available) |

Schulz 1986

| Notes | Full-text not available (no abstract available) |

Silwer 1958

| Notes | Full-text not available (no abstract available) |

Trauth 1980

| Notes | Full-text not available (no abstract available) |

## References to studies

### Nigam 1984 {published data only}

- Nigam P, Kapoor KK, Gupta AK, Gupta RK, Goyal BM. Profile of pulmonary infections in diabetes mellitus. Indian Journal of Chest Diseases & Allied Sciences 1984;26(3):150-4.

### Schulz 1986 {published data only}

- Schulz KT, Fischer P, Schilling W. Tuberculosis as a secondary disease. Zeitschrift fur Arztliche Fortbildung 1986;80(16):687-90.

### Silwer 1958 {published data only}

- Silwer H, Oscarsson PN. Incidence and coincidence of diabetes mellitus and pulmonary tuberculosis in a Swedish county. Acta Medica Scandinavica. Supplementum 1958;335:1-48.

### Trauth 1980 {published data only}

- Trauth HA, Schmidt W. The role of latent diabetes in the re-activation of tuberculosis of the lungs (author's transl). Praxis und Klinik der Pneumologie 1980;34(12):724-31.
