# Supplementary material for: Undernutrition as a risk factor for tuberculosis disease
Source: Cochrane Database Syst Rev. 2024 Jun 11;2024(6):CD015890. doi: 10.1002/14651858.CD015890.pub2 (PMC11165671; doi:10.1002/14651858.CD015890.pub2)
Supplement: Supplementary file 5 — Supplementary material 5 Characteristics of ongoing studies [file CD015890-SUP-05-characteristicsOfOngoingStudies.html]

Characteristics of ongoing studies


# Supplementary material 5 to: Undernutrition as a risk factor for tuberculosis disease

Franco JVA, Bongaerts B, Metzendorf MI, Risso A, Guo Y, Peña Silva L, Boeckmann M, Schlesinger S, Damen JAAG, Richter B, Baddeley A, Bastard M, Carlqvist A, Garcia-Casal MN, Hemmingsen B, Mavhunga F, Manne-Goehler J, Viney K
  
https://doi.org/10.1002/14651858.CD015890.pub2

The material in this section has been supplied by the author(s) for publication under a Licence for Publication and the author(s) are solely responsible for the material. Cochrane has reviewed this material, but Cochrane has not copyedited, formatted or proofread. Cochrane accordingly gives no representations or warranties of any kind in relation to, and accepts no liability for any reliance on or use of, such material.

Back to top

# Characteristics of ongoing studies

## Table of contents

- Studies ordered by Study ID
  - Marambire 2022
  - NCT04526613 2020
- References to studies

## Studies ordered by Study ID

Marambire 2022

| Study name | Early Risk Assessment in Household Contacts (≥10 Years) of TB Patients by New Diagnostic Tests in 3 African Countries (ERASE-TB) |
| Starting date | Study Start (Actual): 1 March 2021  Primary Completion (Estimated): 30 April 2024  Study Completion (Estimated): 30 April 2024 |
| Contact information | Edson Tawanda Marambire: edsonmarambire@gmail.com |
| Notes | Based on Tanzania, Zimbawe and Mozambique - Follow-up of household contacts. |

NCT04526613 2020

| Study name | The Influence of Malnutrition, Diabetes Mellitus, and Helminth Infections on Biosignatures in Latent Tuberculosis in a South Indian Population |
| Starting date | Study Start (Actual): 19 April 2021  Primary Completion (Estimated): 25 June 2025  Study Completion (Estimated) 25 June 2025  Enrollment (Actual): 626  Study Type: Observational |
| Contact information | Not available. |
| Notes | The study is classified as 'cross sectional' but it is unclear if it has a prospective follow-up element. |

## References to studies

### Marambire 2022 {published data only}

[ctg: NCT04781257]

- Marambire ET, Banze D, Mfinanga A, Mutsvangwa J, Mbunda TD, Ntinginya NE, et al. Early risk assessment in paediatric and adult household contacts of confirmed tuberculosis cases by novel diagnostic tests (ERASE-TB): protocol for a prospective, non-interventional, longitudinal, multicountry cohort study. BMJ Open 2022;12(7):e060985. [DOI: 10.1136/bmjopen-2022-060985]

### NCT04526613 2020 {published data only}

[ctg: NCT04526613 ]

- NCT04526613. The influence of malnutrition, diabetes mellitus, and helminth infections on biosignatures in latent tuberculosis in a South Indian population [A cross-sectional study to estimate the influence of malnutrition, diabetes mellitus and helminth infections on biosignatures in latent tuberculosis in a South Indian population]. https://classic.clinicaltrials.gov/ct2/show/NCT04526613 2020.
