# Supplementary material for: Undernutrition as a risk factor for tuberculosis disease
Source: Cochrane Database Syst Rev. 2024 Jun 11;2024(6):CD015890. doi: 10.1002/14651858.CD015890.pub2 (PMC11165671; doi:10.1002/14651858.CD015890.pub2)
Supplement: Supplementary file 7 — Supplementary material 7 Overlap of study populations [file CD015890-SUP-07-other.html]

Overlap of study populations


# Supplementary material 7 to: Undernutrition as a risk factor for tuberculosis disease

Franco JVA, Bongaerts B, Metzendorf MI, Risso A, Guo Y, Peña Silva L, Boeckmann M, Schlesinger S, Damen JAAG, Richter B, Baddeley A, Bastard M, Carlqvist A, Garcia-Casal MN, Hemmingsen B, Mavhunga F, Manne-Goehler J, Viney K
  
https://doi.org/10.1002/14651858.CD015890.pub2

The material in this section has been supplied by the author(s) for publication under a Licence for Publication and the author(s) are solely responsible for the material. Cochrane has reviewed this material, but Cochrane has not copyedited, formatted or proofread. Cochrane accordingly gives no representations or warranties of any kind in relation to, and accepts no liability for any reliance on or use of, such material.

Back to top

# Overlap of study populations

| Study ID | Period | Country | Sources of participants |
| --- | --- | --- | --- |
| Chan-Yeung 2007 | 2000-2005 | China | TB control program |
| Chen 2022 | 2013-2015 | China | General Population |
| Cheng 2020 | 2013-2015 | China | Elderly adults |
| Leung 2007 | 2000-2005 | China | Population-based cohort |
| Long 2020 | 2012-2018 | China | Focus on participants with rheumatic diseases |
| Ahmed 2018 | 2010-2015 | Ethiopia | Afar Regional State, north-east Ethiopia |
| Alemu 2020 | 2013-2018 | Ethiopia | Addis Ababa Region (multiple centres) |
| Ayana 2021 | 2016-2019 | Ethiopia | Addis Ababa (Zewditu, **covered in** Getu 2022) |
| Beshir 2019 | 2013-2017 | Ethiopia | Adama Referral Hospital and Medical College, Oromia |
| Gedfew 2020 | 2013-2017 | Ethiopia | Debre Markos Referral Hospital, Northwest Ethiopia |
| Getu 2022 | 2016-2020 | Ethiopia | Addis Ababa (Zewditu) |
| Tiruneh 2019 | 200-2012 | Ethiopia | Nekemte Town, Western Ethiopia |
| Hanrahan 2010 | 2003-2008 | South Africa | Johannesburg |
| Van Rie 2011 | 2004-2008 | South Africa | Soweto |
| Cho 2022 | 2010-2017 | South Korea | National Database (general, **covered in** Park 2022) |
| Choi 2021 | 2011-2014 | South Korea | National Database (general, **covered in** Park 2022) |
| Jung 2016 | 2007-2009 | South Korea | Clinical Records |
| Kim 2018 | 2002-2006 | South Korea | National Database (general) |
| Park 2022 | 2009-2017 | South Korea | National Database (general) |
| Park 2023 | 2002-2017 | South Korea | National Database (gastrectomy, **covered in** Park 2022 and Kim 2018) |
| Yoo 2021a | 2009-2014 | South Korea | National Database (older adults) |
| Youn 2022 | 2002-2013 | South Korea | National Database (recurrence) |
| Baker 2012 | 2001-2004 | Taiwan, China | National Database (general, reported HR) |
| Lin 2018 - NIHS cohort | 2001-2013 | Taiwan, China | National Database (general, **covered in** Yen 2017) |
| Yen 2017 | 2001-2013 | Taiwan, China | National Database (general, reported RR) |
| Lin 2018 - NTC cohort | 2005-2013 | Taiwan, China | Population-based cohort |
| Li 2013 | 2004-2011 | Tanzania | Dar es Salaam (children) |
| Liu 2015 | 2004-2012 | Tanzania | Dar es Salaam (adults) |
| Maro 2010 | 2001-2005 | Tanzania | Dar es Salaam - adults (TB vaccine study) |
| Sabasaba 2019 | 2011-2014 | Tanzania | Dar es Salaam (adults) |
| Sudfeld 2013 | 2006-2009 | Tanzania | Dar es Salaam (vitamin supplementation study) |
| Moore 2007 | 2003-2005 | Uganda | Home-based AIDS Care Project - hazard ratios |
| Were 2009 | 2003-2005 | Uganda | Home-based AIDS Care Project - risk ratios |
| Worodria 2011 | N/A | Uganda | Infectious Disease Institute, Kampala |
