# Supplementary material for: Undernutrition as a risk factor for tuberculosis disease
Source: Cochrane Database Syst Rev. 2024 Jun 11;2024(6):CD015890. doi: 10.1002/14651858.CD015890.pub2 (PMC11165671; doi:10.1002/14651858.CD015890.pub2)
Supplement: Supplementary file 8 — Supplementary material 8 Sensitivity Analyses [file CD015890-SUP-08-other.html]

Sensitivity Analyses


# Supplementary material 8 to: Undernutrition as a risk factor for tuberculosis disease

Franco JVA, Bongaerts B, Metzendorf MI, Risso A, Guo Y, Peña Silva L, Boeckmann M, Schlesinger S, Damen JAAG, Richter B, Baddeley A, Bastard M, Carlqvist A, Garcia-Casal MN, Hemmingsen B, Mavhunga F, Manne-Goehler J, Viney K
  
https://doi.org/10.1002/14651858.CD015890.pub2

The material in this section has been supplied by the author(s) for publication under a Licence for Publication and the author(s) are solely responsible for the material. Cochrane has reviewed this material, but Cochrane has not copyedited, formatted or proofread. Cochrane accordingly gives no representations or warranties of any kind in relation to, and accepts no liability for any reliance on or use of, such material.

Back to top

# Sensitivity Analyses

| Analysis | Follow-up | HR | Lower CI | Upper CI | Lower PI | Upper PI | I2 |
| --- | --- | --- | --- | --- | --- | --- | --- |
| All | <10 years | 1.892 | 1.687 | 2.121 | 1.168 | 3.064 | 86.54 |
| Excluding high risk of bias studies | <10 years | 2.02 | 1.741 | 2.342 | 1.202 | 3.393 | 71.55 |
| ROB1 | <10 years | 1.909 | 1.482 | 2.457 | 0.884 | 4.122 | 88.11 |
| ROB2 | <10 years | 1.994 | 1.723 | 2.307 | 1.33 | 2.987 | 63.03 |
| ROB3 | <10 years | 1.841 | 1.635 | 2.074 | 1.141 | 2.971 | 85.75 |
| ROB4 | <10 years | 1.699 | 1.158 | 2.494 | 0.533 | 5.417 | 54.81 |
| ROB5 | <10 years | 1.894 | 0.889 | 4.032 | 0 | 12369.059 | 76.47 |
| ROB6 | <10 years | 1.831 | 1.628 | 2.06 | 1.208 | 2.775 | 83.67 |
| All | 10 or more | 9.011 | 1.611 | 50.398 | 0 | 2.59E+10 | 95.44 |
| Excluding high risk of bias studies | 10 or more | 12.43 | 5.742 | 26.91 | . | . | . |
| ROB1 | 10 or more | 4.679 | 0.749 | 29.226 | . | . | 95.56 |
| ROB2 | 10 or more | 1.913 | 1.852 | 1.977 | . | . | . |
| ROB3 | 10 or more | 20.157 | 6.884 | 59.024 | . | . | 62.06 |
| ROB4 | 10 or more | . | . | . | . | . | . |
| ROB5 | 10 or more | . | . | . | . | . | . |
| ROB6 | 10 or more | 9.011 | 1.611 | 50.398 | 0 | 2.59E+10 | 95.44 |
| All | Overall | 2.174 | 1.818 | 2.601 | 0.861 | 5.492 | 97.32 |
| Excluding high risk of bias studies | Overall | 2.229 | 1.827 | 2.719 | 0.984 | 5.049 | 86.16 |
| ROB1 | Overall | 2.254 | 1.48 | 3.434 | 0.535 | 9.495 | 98.67 |
| ROB2 | Overall | 1.99 | 1.772 | 2.234 | 1.441 | 2.747 | 71.85 |
| ROB3 | Overall | 2.2 | 1.771 | 2.732 | 0.743 | 6.513 | 96.76 |
| ROB4 | Overall | 1.699 | 1.158 | 2.494 | 0.533 | 5.417 | 54.81 |
| ROB5 | Overall | 1.894 | 0.889 | 4.032 | 0 | 12369.059 | 76.47 |
| ROB6 | Overall | 2.274 | 1.672 | 3.093 | 0.555 | 9.319 | 98.97 |
| **CI**: confidence interval; **HR**: hazard ratio; **PI**: prediction interval; **ROB**: risk of bias.    ROB1, ROB2, ROB3, ROB4, ROB5, and ROB6 are the analyses restricted to studies of low risk of bias in each domain. ROB1: Study Participation; ROB2: Study Attrition; ROB3: Prognostic Factor Measurement; ROB4: Outcome Measurement; ROB5: Study Confounding; ROB6: Statistical Analysis and Reporting. | | | | | | | |
